# Subcutaneous Lenacapavir in People With Multidrug-Resistant HIV-1: 156 Week Results of the CAPELLA Study

**DOI:** 10.1093/ofid/ofaf763

**Published:** 2025-12-19

**Authors:** Onyema Ogbuagu, Andrew Wiznia, Joseph P McGowan, Daniel S Berger, Catherine M Creticos, Debbie Hagins, Theo Hodge, Olayemi Osiyemi, James Sims, David A Wheeler, Hui Wang, Nicolas A Margot, Hadas Dvory-Sobol, Martin S Rhee, Sorana Segal-Maurer, Jean-Michel Molina

**Affiliations:** Department of Internal Medicine, Yale School of Medicine, New Haven, Connecticut, USA; Jacobi Medical Center, Albert Einstein College of Medicine, Bronx, New York, USA; Center for AIDS Research and Treatment, Northwell Health, Manhasset, New York, USA; Northstar Healthcare, Chicago, Illinois, USA; Howard Brown Health, Chicago, Illinois, USA; Coastal District Care Clinic, Savannah, Georgia, USA; Washington Health Institute, Washington, District of Columbia, USA; Triple O Research Institute PA, West Palm Beach, Florida, USA; St Hope Foundation, Houston, Texas, USA; Infectious Diseases Physicians, Inc., Annandale, Virginia, USA; Gilead Sciences, Inc., Foster City, California, USA; Gilead Sciences, Inc., Foster City, California, USA; Gilead Sciences, Inc., Foster City, California, USA; Gilead Sciences, Inc., Foster City, California, USA; Division of Infectious Diseases, New York-Presbyterian Queens, Flushing, New York, USA; Department of Infectious Diseases, Université de Paris Cité, Saint-Louis and Lariboisière Hospitals, APHP, Paris, France; INSERM 1342, Université Paris Cité, 1 Ave Claude Vellefaux, Paris 75010, France

**Keywords:** HIV I, HIV I - therapy, HIV/AIDS, HIV/AIDS - therapy, infections, viruses

## Abstract

**Background:**

Lenacapavir is a twice-yearly HIV-1 capsid inhibitor approved, in combination with other antiretrovirals, for the treatment of heavily treatment-experienced people with multidrug-resistant HIV, based on the Phase 2/3 CAPELLA study. Here, we report week 156 efficacy and safety results.

**Methods:**

In CAPELLA (NCT04150068), participants received 2-week oral lenacapavir lead-in doses, followed by subcutaneous lenacapavir every 6 months, combined with an investigator-selected optimized background regimen. Endpoints included virologic outcomes, CD4 cell count trends, adverse events, and treatment-emergent resistance.

**Results:**

CAPELLA enrolled 72 participants: 25% female; 38% Black; median age, 52 years; CD4 cell count <200 cells/μL, 64% (<50 cells/μL, 22%). At week 156, 61% (43/70) had HIV RNA <50 copies/mL by FDA Snapshot Algorithm, 16% (11/70) had ≥50 copies/mL, and 23% (16/70) had missing data; by missing = excluded analysis, 85% (44/52) had HIV RNA <50 copies/mL. Mean CD4 cell count increase from baseline to week 156 was 164 cells/μL (95% CI: 116–211). Through week 156, 14/72 participants developed emergent LEN resistance. Injection site reactions were mostly Grade 1/2, and frequency declined over time. Through week 156, two participants discontinued lenacapavir due to Grade 1 injection site nodules.

**Conclusions:**

Lenacapavir plus an optimized background antiretroviral regimen maintained a high rate of virologic suppression at week 156 with continued increases in CD4 counts. Lenacapavir was well tolerated with a favorable safety profile and very low rates of lenacapavir discontinuation. These data demonstrate longer-term efficacy and safety of lenacapavir for heavily treatment-experienced people with multidrug-resistant HIV.

## Teaser

Lenacapavir plus an optimized background regimen maintained high virologic suppression rates over 3 years in heavily treatment-experienced people with multidrug-resistant HIV-1 and limited treatment options, with ongoing robust immune recovery and a favorable safety profile.

Lenacapavir (LEN) is a first-in-class HIV-1 capsid inhibitor which targets viral nuclear import, virion assembly, and capsid core assembly, thereby inhibiting virion production and decreasing virion infectivity [1]. LEN is indicated for the treatment of HIV infection in heavily treatment-experienced (HTE) adults with multidrug-resistant (MDR) HIV infection whose current antiretroviral (ARV) regimen is failing due to resistance, intolerance, or safety considerations [2]. As LEN has a novel mechanism of action, it is an effective component of regimens for people with HIV with multiclass HIV drug resistance [2].

The approval of LEN was based on the results of the ongoing Phase 2/3 CAPELLA study (NCT04150068), in which LEN combined with an optimized background regimen (OBR) resulted in a high rate of virologic suppression in participants with MDR HIV [3]. LEN achieved its primary endpoint as 14-day functional monotherapy when added to a failing regimen: 88% of participants on LEN had a ≥ 0.5 log_10_ decline in HIV RNA versus 17% on placebo (*P* < .001) [3]. At week (W)26, virologic suppression (HIV-1 RNA <50 copies/mL) was achieved by 82% of participants by the US Food and Drug Administration (FDA) Snapshot Algorithm, with a mean increase from baseline in CD4 cells of 89 cells/μL [3]. At W52, virologic suppression was achieved by 78% of HTE participants by the FDA Snapshot Algorithm, with a mean increase in CD4 cells of 97 cells/μL from baseline [4]. After W52, the protocol was amended to allow longer follow-up, and at W104, 62% of participants had virologic suppression, with a mean increase from baseline in CD4 cells of 122 cells/μL [5]. When analyzed as missing = excluded (M = E), 82% of participants achieved virologic suppression at W104. The aim of this analysis was to assess the efficacy and safety of LEN in people with MDR HIV through W156.

## METHODS

### Study Design and Participants

CAPELLA is an ongoing, Phase 2/3 study in people with MDR HIV. Participants received 2-week oral LEN lead-in doses followed by twice-yearly subcutaneous (SC) LEN combined with an investigator-selected OBR of ARV agents. The full study design, including inclusion and exclusion criteria and procedures, has been described previously (see also Supplementary Appendix) [3, 4]. Briefly, key eligibility criteria included HIV RNA ≥400 copies/mL at screening, on a stable but failing ARV regimen for ≥8 weeks, resistance to ≥2 ARVs from ≥3 of the four main ARV drug classes (nucleos[t]ide reverse transcriptase inhibitor, non-nucleoside reverse transcriptase inhibitor, protease inhibitor, and integrase strand-transfer inhibitor). Participants were enrolled at 42 study sites in Canada, the Dominican Republic, France, Germany, Italy, Japan, South Africa, Spain, Taiwan, Thailand, and the USA. The trial was approved by the institutional review board or ethics committee at all sites and was carried out in compliance with international laws and guidelines. All participants provided written informed consent.

**Figure 1.**
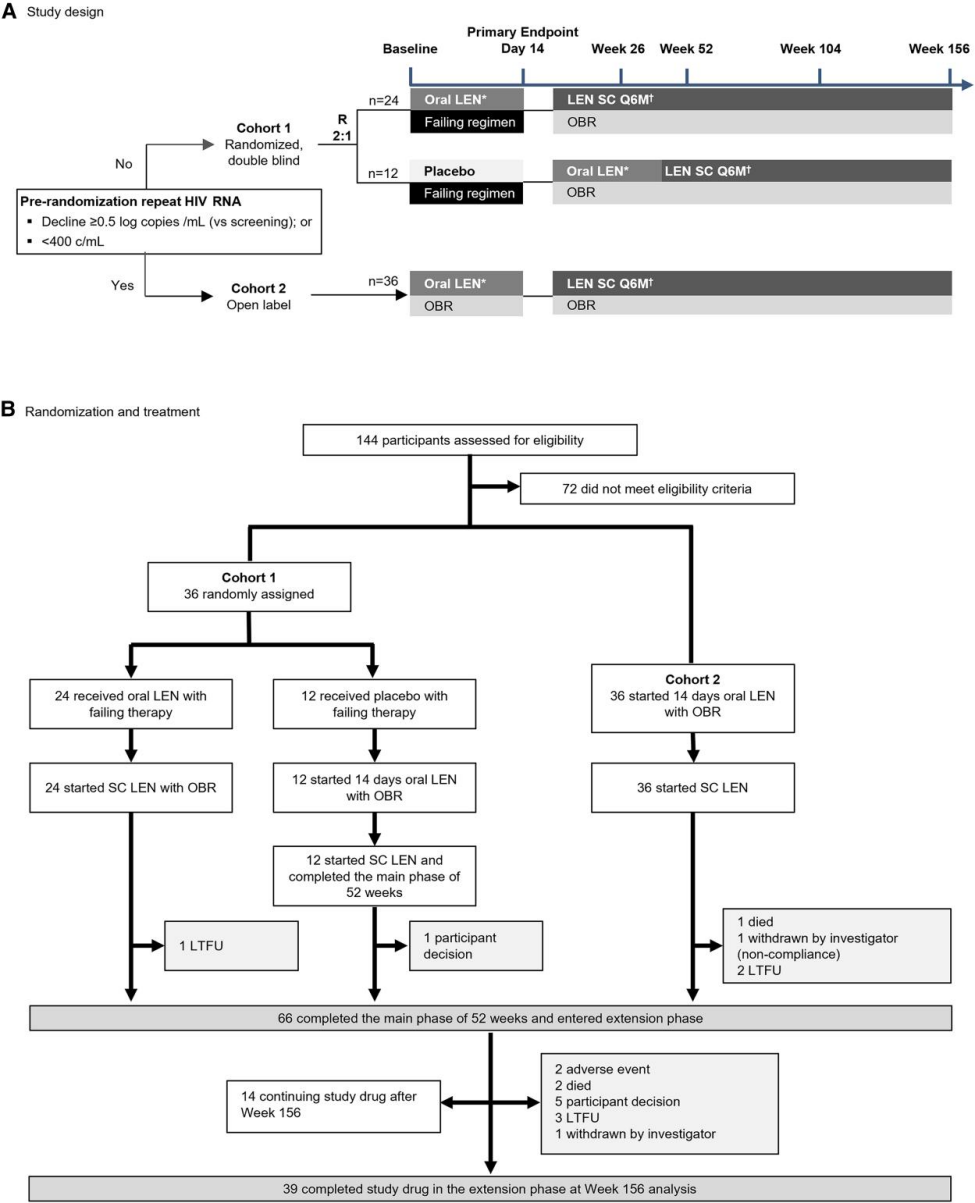
*A*, CAPELLA study design. *B*, Randomization and treatment. Overall, six participants were lost to follow-up, five discontinued due to participant decision, three died (one due to cancer on Day 90, one to respiratory failure on Day 568, and one with unknown cause on Day 551; none were considered related to study drug), two discontinued study drug because of injection site reactions (both subcutaneous nodule; Grade 1, related to study drug), one was withdrawn by the investigator at week 4 due to suboptimal adherence to their OBR, one completed study drug during the extension phase after rolling over to a lenacapavir access program, and one completed the study at week 52 but did not receive the week 52 lenacapavir injection or enter the extension phase due to participant decision. For participants with emergent LEN resistance, the decision to discontinue study participation was made jointly by the investigator and the participant. Participants with resistance at week 52 were permitted to continue in the study if both the investigator and the participant agreed that the potential benefit of continued participation outweighed the associated risks, regardless of whether the participant had resistance. *Days 1 and 2: 600 mg; Day 8, 300 mg. ^†^927 mg as two 1.5-mL injections in the abdomen on Day 15, then Q6M. ^‡^One participant had missing HIV RNA at the week 156 analysis. LEN, lenacapavir; LTFU, lost to follow-up; OBR, optimized background regimen; Q6M, every 6 months; R, randomized; SC, subcutaneous. Figure 1A has been repoduced with permission from Segal-Maurer S, et al. [3].

Full methods related to the sample size calculation and the randomization process have been reported previously [3, 4, 6], and the protocol and statistical analysis plan are provided in the Supplementary Appendix. The study design is depicted in Figure 1*A*. The randomization and treatment up to W156 is shown in Figure 1*B*. Participants could continue to receive study drug beyond 52 weeks until SC LEN became accessible through an access program or commercially available. Changes to the OBR were permitted at the clinician's discretion and with medical monitor approval.

### Outcomes

The primary efficacy endpoint for the CAPELLA study was the proportion of participants in the randomized cohort with a reduction of at least 0.5 log_10_ copies/mL in plasma HIV RNA by Day 15 of LEN added to a stable failing ARV regimen [3]. Secondary endpoints included efficacy and safety outcomes at W26, 52, 104, and 156. At W156, the proportion of participants with plasma HIV RNA <50 copies/mL and <200 copies/mL were evaluated based on the FDA-defined snapshot algorithm. Virological failure was defined as any of the following: a confirmed HIV RNA of ≥50 copies/mL and a decrease <1 log_10_ copies/mL at W4 after the initiation of oral LEN; a rebound in HIV RNA to ≥50 copies/mL after a previous measurement <50 copies/mL, confirmed at the following visit; or an increase from the nadir value of >1 log_10_ copies/mL, confirmed at the following visit.

Additional outcomes evaluated at W156, as prespecified in the statistical analysis plan, included proportion of participants with HIV RNA <50 copies/mL by M=E analysis, change in CD4 cell count from baseline to W156, resistance emergence, and treatment-emergent adverse events (TEAEs), including injection site reactions (ISRs).

Participants who met criteria for virologic failure, as described above, underwent resistance analysis. HIV capsid sequencing as well as protease, reverse transcriptase, and integrase resistance testing were performed as previously described using specimens collected at the initial or confirmed virologic failure visit [7]. Capsid inhibitor resistance-associated mutations were defined based on previous *in vitro* and/or *in vivo* studies: L56I, M66I, Q67H/K/N, K70H/N/R/S, N74D/H/K, A105S/T, and T107C/N [7–9].

### Statistical analysis

The FDA Snapshot Algorithm was used to assess the secondary endpoints of the percentage of participants with HIV RNA <50 and <200 copies/mL at W156 [10]. The virologic outcome was defined as the following categories: HIV RNA <50 copies/mL or <200 copies/mL (including participants with last available on-treatment HIV RNA <50 copies/mL or <200 copies/mL in the W156 analysis window); HIV RNA ≥50 copies/mL or ≥200 copies/mL; or no virologic data in the W156 analysis window. All efficacy data up to W156 were included in these analyses for both cohorts. All participants who had received at least one dose of LEN were assessed for safety. All adverse events data and laboratory findings were as of 29 January 2024 and 12 March 2024, respectively. Baseline and the change from baseline in HIV RNA (log_10_ copies/mL) and CD4 cell count (cells/µL) by visit were summarized using descriptive statistics. Resistance and safety data are described in summary form.

## RESULTS

Of the 72 participants originally enrolled in the CAPELLA study, 67 completed the main phase through W52, 66 of whom consented to enter the extension phase (Figure 1*B*). The W156 FDA Snapshot Analysis included 70/72 participants; participants who had missing HIV RNA at W156 and had completed the study before reaching the upper limit of the analysis window for W156 were excluded (*n* = 2). Of the 66 participants who entered the extension phase, 14 participants were excluded from the M=E analysis (due to discontinuation [*n* = 13] and missing HIV RNA [*n* = 1]), leaving 52 participants with HIV RNA data available. The median (interquartile range [IQR]) duration of follow-up on LEN was 165 (146–178) weeks.

Baseline demographics and clinical characteristics are shown in Supplementary Table 1 and have been reported in previous analyses [3–5]. The median age was 52 years and 75% of participants were male. Almost two-thirds of participants (63.9%) had baseline CD4 cell counts <200 cells/μL, with 22.2% < 50 cells/μL, and 19.4% had baseline HIV RNA >100 000 copies/mL. The median (IQR) time since HIV diagnosis was 24 (21–29) years and initiation of HIV treatment was 23 (18–27) years. The median (IQR) number of previous ARV medications was 11 (8–16). Nearly half of participants (45.8%) had resistance to ≥2 drugs from all four major classes of ARVs, and 12 of the 72 participants (16.7%) had no fully active ARVs in their OBR.

**Figure 2.**
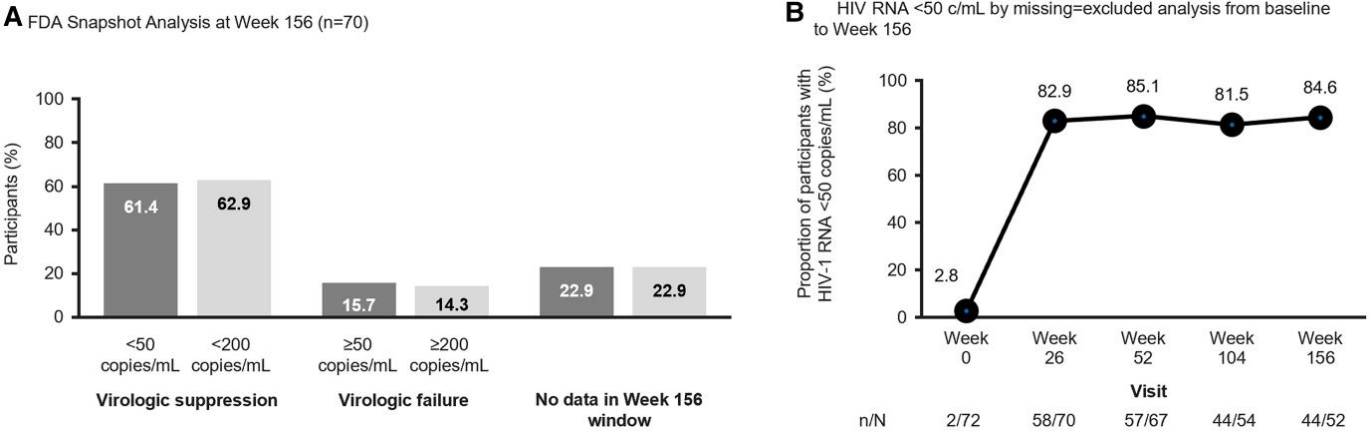
Virologic outcomes. *A*, FDA snapshot analysis at week 156 (*n* = 70). The week 156 window was Day 779 to Day 869 inclusive (*n* = 70); participants who had missing HIV RNA at week 156 and had completed the study before reaching the upper limit of the analysis window for week 156 were excluded (*n* = 2). *B*, HIV RNA <50 copies/mL by missing = excluded analysis from baseline to week 156. The denominator for percentages is the number of participants with nonmissing HIV RNA values at each time point. At week 156, there were 66 participants who completed the main phase and entered the extension phase, of whom 13 discontinued study drug in the extension phase prior to week 156 and one participant did not have a viral load result in the week 156 analysis window, leaving *n* = 52 for the missing = excluded analysis. Of these 52 participants, 21 were from Cohort 1 LEN + failing therapy arm, 7 from the Cohort 1 placebo + failing therapy arm, and 24 were from Cohort 2.

By FDA Snapshot Analysis, 43/70 (61.4%) participants had HIV RNA <50 copies/mL at W156 (Figure 2*A*) and 44/70 (62.9%) had HIV RNA <200 copies/mL. Data by cohort are depicted in Supplementary Figure 1. By M=E analysis, 44/52 (84.6%) participants had HIV RNA <50 copies/mL at W156 (Figure 2*B*). Overall, 98% of SC injections up to W156 were within ±14 days of the scheduled dose.

**Figure 3.**
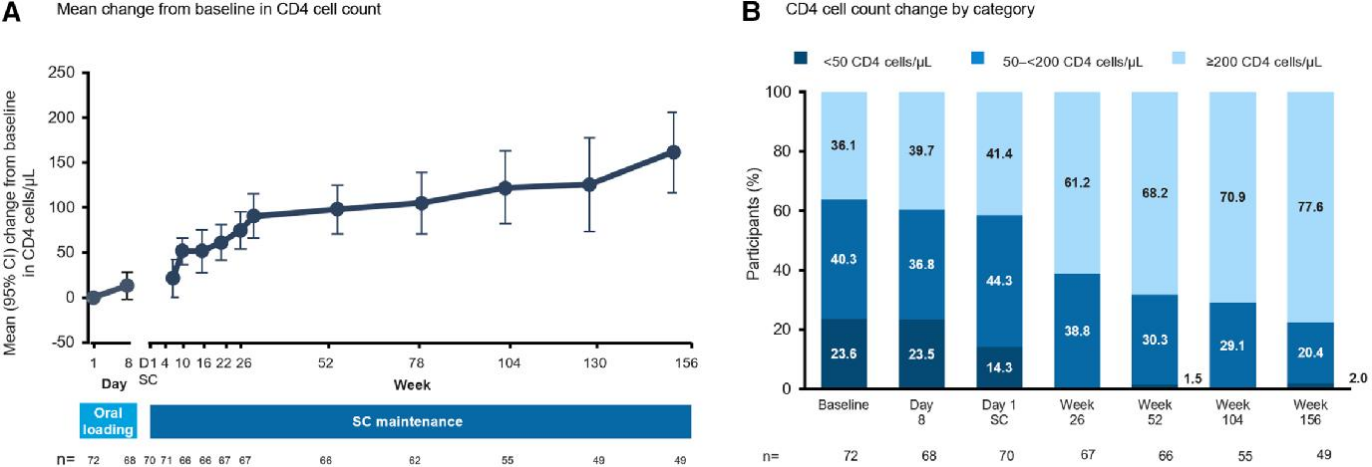
CD4 cell count changes. *A*, Mean change from baseline in CD4 cell count. *B*, CD4 cell count change by category. D1 SC: first day subcutaneous lenacapavir was administered.

The mean increase in CD4 count from baseline to W156 was 164 cells/µL (95% CI: 116–211) (Figure 3*A*; data by cohort are depicted in Supplementary Figure 2). From baseline to W156, the proportion of participants with CD4 counts <200 and <50 cells/µL decreased from 64% to 22% and 24% to 2%, respectively (Figure 3*B*).

Twenty-eight participants met protocol-defined criteria for resistance analysis up to W156. Overall, there were 14 cases of emergent LEN resistance in the CAPELLA study: eight before W26, one between W26 and 52, and five between W52 and 104, with no new cases between W104 and 156 [3–5]. Details of participants' viral load and resistance-associated mutations are provided in Supplementary Table 2.

Six participants had emergence of the M66I mutation along with other resistance-associated substitutions, four had emergence of Q67H + K70R with or without the secondary mutations A105T and/or T107N, one had emergence of K70N + N74K + T107T/N, one had emergence of N74D alone, one had emergence of Q67H alone, and one had emergence of Q67K + K70H.

Whilst no new cases of LEN resistance emerged between W104 and W156, two participants developed additional resistance-associated mutations between W104 and W156. Of these two participants, one had emergence of K70R + T107N with existing Q67H, and one had emergence of T107T/N with existing K70N + N74K.

All 14 participants with LEN resistance had either no fully active drugs in their OBR (*n* = 4) or inadequate OBR adherence based on the plasma concentrations of the OBR drugs (*n* = 10). Of the 14 participants with LEN resistance emergence, five resuppressed whilst remaining on LEN. Of those who did not resuppress after LEN resistance emergence, two returned to baseline viral load, three had a < 1 log_10_ reduction in HIV RNA, and four had a > 1 log_10_ reduction in HIV RNA. The participants with LEN resistance emergence had a mean increase in CD4 count of 193 cells/µL (95% CI: 74–313) from baseline to W156.

TEAEs are summarized in Table 1. The most common TEAEs, excluding ISRs and COVID-19, were diarrhea (15/72 [20.8%]), nausea (14/72 [19.4%]), cough (12/72 [16.7%]), and urinary tract infection (12/72 [16.7%]). Most were mild to moderate in severity and not considered related to LEN.

**Table 1.** Treatment-Emergent Adverse Events (TEAE) (*N* = 72)

*n* (%)	All	Treatment-related TEAEs
Any TEAE	71 (98.6)	57 (79.2)
Grade 3 or higher	31 (43.1)	6 (8.3)^a^
Serious	22 (30.6)	0 (0)
TEAEs, excluding injection site reactions, occurring in ≥10% of all participants		
COVID-19	18 (25.0)	0 (0)
Diarrhea	15 (20.8)	5 (6.9)
Nausea	14 (19.4)	4 (5.6)
Cough	12 (16.7)	0 (0)
Urinary tract infection	12 (16.7)	0 (0)
Constipation	10 (13.9)	0 (0)
Headache	10 (13.9)	2 (2.8)
Pyrexia	10 (13.9)	2 (2.8)
Abdominal distension	8 (11.1)	1 (1.4)
Arthralgia	8 (11.1)	0 (0)
Back pain	8 (11.1)	0 (0)
Grade 3 or higher occurring in >1 participant		
Pneumonia	4 (5.6)	0 (0)
Cellulitis	2 (2.8)	0 (0)
Dehydration	2 (2.8)	0 (0)
Hypotension	2 (2.8)	0 (0)
Pneumonia staphylococcal	2 (2.8)	0 (0)
Squamous cell carcinoma	2 (2.8)	0 (0)
Injection site reactions		
Injection site swelling	34 (47.2)	34 (47.2)
Injection site nodule	28 (38.9)	28 (38.9)
Injection site pain	28 (38.9)	25 (34.7)
Injection site erythema	26 (36.1)	25 (34.7)
Injection site induration	11 (15.3)	11 (15.3)
Grade 3 or higher occurring in >1 participant		
Injection site erythema	6 (8.3)	3 (4.2)
Injection site swelling	4 (5.6)	3 (4.2)
Injection site pain	3 (4.2)	2 (2.8)
Injection site edema	2 (2.8)	0 (0)

Abbreviation: TEAE, treatment-emergent adverse event.

^a^Injection site reaction (*n* = 4), immune reconstitution inflammatory syndrome (*n* = 1); abdominal abscess (*n* = 1); rash (*n* = 1).

Grade 3 TEAEs are included in Table 1, and treatment-emergent serious AEs are described in Supplementary Table 3. Most Grade 3 TEAEs that were related to LEN were ISRs. No participant experienced a treatment-related serious adverse event (AE) or a Grade 4 treatment-related AE.

**Figure 4.**
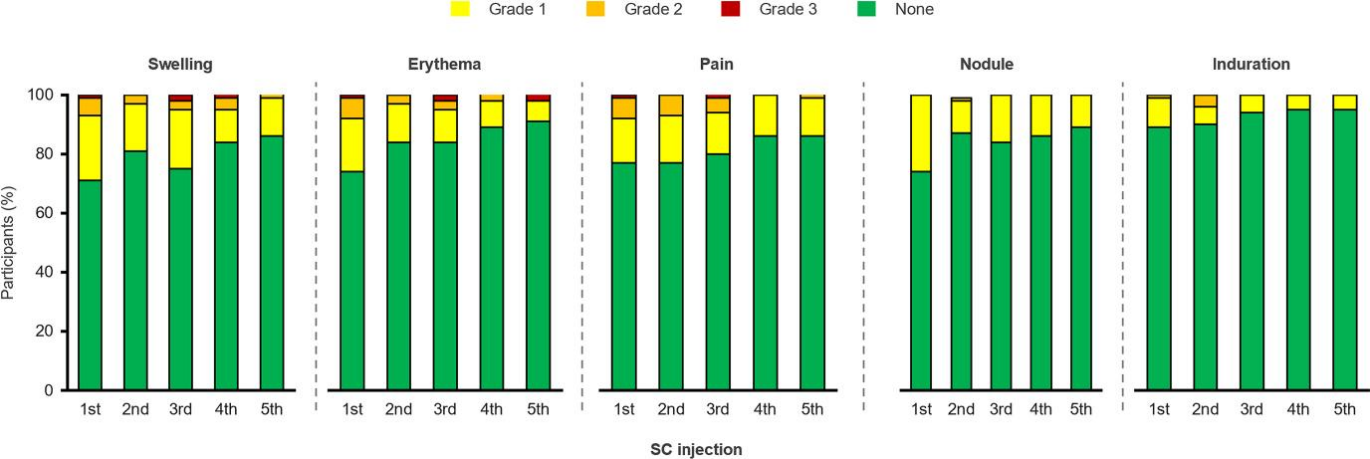
Incidence and severity of most common SC LEN-related ISRs. Details (percentages) for each ISR can be found in Supplementary Table 5. Percentage denominators are the number of participants who received an injection at that visit. Percentages may not total 100% due to rounding. ISR, injection site reaction; LEN, lenacapavir; SC, subcutaneous.

The most common SC LEN-related ISRs are shown in Figure 4. Most (97.2%) of the ISRs experienced by participants (383/394 individual events) were Grade 1 or 2. The percentages of participants with any LEN-related ISRs decreased in frequency over time (Supplementary Table 4). After SC LEN injections at day 15, W26, and W104, 44/72 (61.1%), 32/70 (45.7%), and 21/56 (37.5%) experienced ISRs, respectively. The median (IQR) duration of swelling, erythema, pain, nodules, and induration was 8 (4–15) days, 5 (3–8) days, 3 (2–5) days, 288 (155–548) days, and 190 (67–410) days, respectively.

Overall, 28 participants (38.9%) experienced LEN-related injection site nodule, and 11 participants (15.3%) experienced LEN-related injection site induration. All events were Grade 1 or 2. Of the individual nodule events that occurred after the first injection, 20/27 (74.1%) resolved with a median (IQR) duration of 191 (71–366) days and 7/27 (25.9%) are not fully resolved. Of the induration events that occurred after the first injection, 8/8 (100%) resolved with a median (IQR) duration of 113 (29–224) days. Two participants discontinued study drug due to grade 1 injection site nodules (*n* = 1 between W104 and W156).

Grade 3 or 4 laboratory abnormalities were noted in 29 (40.3%) of the 72 participants (Supplementary Table 5). Thirteen participants had grade 4 laboratory abnormalities: elevated creatinine (*n* = 5), low creatinine clearance (estimated glomerular filtration rate; *n* = 5), elevated aspartate aminotransferase (*n* = 3), elevated alanine aminotransferase (*n* = 1), fasting hyperglycemia (*n* = 1), nonfasting hyperglycemia (*n* = 1), elevated creatine kinase (*n* = 1), and elevated lipase (*n* = 1). High creatinine levels, low creatinine clearance, and hyperglycemia were transient, not considered clinically significant, and were possibly related to underlying conditions (eg, diabetes).

## DISCUSSION

LEN combined with an OBR continued to result in high and sustained rates of virologic suppression through W156 in HTE people with MDR HIV. Of those still on treatment and with available HIV RNA measurement at W156, 84.6% had HIV RNA <50 copies/mL, demonstrating strong durability of virologic suppression through W156.

Clinically relevant increases in CD4 cell count were observed from baseline to W156, with a mean increase of 164 cells/µL. Additionally, only 36% of participants had CD4 cell count >200 cells/µL at baseline, whereas at W156, 78% had CD4 cell count >200 cells/µL. Of note, CD4 cell counts continued to increase between W104 and 156, indicating a sustained benefit to maintaining a regimen of LEN + OBR for these participants. As the OBR was optimized, other agents may have also contributed to the CD4 cell count increase. Additionally, CD4 cell count increases were noted in some participants who did not achieve viral suppression; this indicates that LEN + OBR, in addition to the viral fitness cost that may come with drug resistance, may still provide immunologic benefit to some people with MDR HIV with no fully active ARV options by allowing CD4 recovery even without full viral suppression. This supports the goal of preserving immune function for HTE people with HIV even when no fully active OBR can be created [11].

Of the 14 participants who had emergent LEN resistance, resistance occurred before W52 for nine participants and between W52 and 104 for five participants, with no new cases occurring between W104 and 156 [5]. All 14 cases were due to LEN functional monotherapy, associated either with resistance to the OBR or nonadherence to the OBR [12], highlighting the importance of combining LEN with other fully active ARVs when possible and supporting adherence to the OBR. Including other clinic-administered long-acting agents in the OBR may address adherence challenges, and the development of novel ARVs with different mechanisms of action and similar dosing schedules to LEN would allow for a complete synchronized regimen with less frequent dosing.

LEN was well tolerated as most TEAEs were mild to moderate and self-limiting, and no participant experienced a treatment-related serious AE or a grade 4 treatment-related AE. The most common TEAEs of diarrhea and nausea are known AEs for many of the agents that were commonly included in the OBRs.

Although ISRs were common, they were mostly grade 1 or 2 and the frequency decreased over time, similar to observations of SC LEN in the CALIBRATE study (NCT04143594) in people with HIV starting a first ARV regimen and the PURPOSE 1 and PURPOSE 2 studies (NCT04994509 and NCT04925752) when used as HIV pre-exposure prophylaxis (PrEP) [13–15].

Subcutaneously injected LEN forms a drug depot which is usually not visible but may manifest as a palpable nodule. The size of the depot decreases over time, as the drug elutes [15–17]. Biopsy data indicate that a granulomatous or foreign body reaction to the depot may occur [18]. Up to W156, only 2 participants (2.8%) discontinued LEN due to ISR nodules.

The observed trend towards fewer ISRs over time might be attributable in part to improved injection technique, which may result in improved tolerability over time [19]. Contributing factors to the development of ISRs appear to be a superficial injection depth and incorrect injection angle resulting in drug delivery into the dermis instead of the SC tissue. Best practices to mitigate ISRs include ensuring that LEN is administered subcutaneously in the abdomen [2]. Strategies to ensure proper administration and minimize ISRs include gently pinching the skin to increase SC tissue available for injection, injecting at a 90° angle, and pausing briefly prior to needle withdrawal to avoid leakage [2].

In the PURPOSE 1 and PURPOSE 2 studies of LEN for PrEP, LEN SC was given to 2134 and 2179 people, respectively [13, 15]. Although the populations differed from CAPELLA, the safety results were similar and ISRs consistently decreased in frequency with subsequent SC doses across populations with low discontinuation rates due to ISRs.

Although adherence to LEN SC injections was high in the setting of the CAPELLA study, LEN can be administered both SC and orally, making it possible to bridge missed SC doses with oral doses. If a patient plans to miss a scheduled 6-month injection visit by more than 2 weeks, LEN 300 mg tablets once every 7 days may be taken for up to 6 months until injections resume [2, 18].

A limitation to this analysis was that study discontinuation over 3 years could have led to some selection bias in the data represented at W156. The main study phase was completed at W52 and while most participants chose to enter the extension phase, expected attrition occurred from the extension phase over time. Additionally, the lack of development of resistance to LEN from W104 to W156 may reflect selection bias for those participants who fully adhered to their OBR and LEN injection times.

The absence of a comparison group for AEs limits the safety analysis, and the small number of participants in this study limits subgroup analyses and the ability to detect low-frequency AEs. Less than 1% of people with HIV are considered to have MDR HIV with limited treatment options, making it difficult to find and enroll large numbers of HTE people with MDR HIV into research studies [20]. However, for this small proportion of people, ARV treatment options are a critical unmet need and the study participants enrolled represent this population well. Longer-term, real-world data and data on LEN for PrEP will continue to contribute to the safety profile of SC LEN.

Finally, the heterogeneity in OBRs limits any guidance on optimal combinations that include LEN for HTE people with MDR HIV based on this study, and there continue to be knowledge gaps with other therapies with less frequent dosing schedules that could be used in combination with LEN for this patient population. It is recommended that an ARV regimen preferably include at least two fully active drugs if at least one has a high resistance barrier [11].

## CONCLUSIONS

LEN combined with an OBR continued to result in high and sustained rates of virologic suppression with CD4 count recovery, and was well tolerated through W156 in the CAPELLA study. These longer-term data support the use of LEN for the treatment of HTE people with MDR HIV.

## Supplementary Material

ofaf763_Supplementary_Data

## Supplementary Data

Supplementary materials are available at *Open Forum Infectious Diseases* online. Consisting of data provided by the authors to benefit the reader, the posted materials are not copyedited and are the sole responsibility of the authors, so questions or comments should be addressed to the corresponding author.
