# Supplementary material for: Subcutaneous Lenacapavir in People With Multidrug-Resistant HIV-1: 156 Week Results of the CAPELLA Study
Source: Open Forum Infect Dis. 2025 Dec 19;13(1):ofaf763. doi: 10.1093/ofid/ofaf763 (PMC12740718; doi:10.1093/ofid/ofaf763)
Supplement: ofaf763_Supplementary_Data [file ofaf763_supplementary_data.zip › FINAL protocol-amd-4-GS-US-200-4625_DS_QCed-f-redact.pdf.pdf]

CLINICAL STUDY PROTOCOL

**Study Title:**	A Phase 2/3 Study to Evaluate the Safety and Efficacy of Long Acting Capsid Inhibitor GS-6207 in Combination With an Optimized Background Regimen in Heavily Treatment Experienced People Living With HIV-1 Infection With Multidrug Resistance
**Sponsor:**	Gilead Sciences, Inc.<br>333 Lakeside Drive<br>Foster City, CA 94404
IND Number:	136260
EU CT Number:	2019-003814-16
Clinical Trials.gov Identifier:	04150068
**Indication:**	HIV-1 Infection
**Protocol ID:**	GS-US-200-4625
**Contact Information:**	The medical monitor name and contact information will be provided on the Key Study Team Contact List.
**Protocol Version/Date:**	Amendment 4: 18 January 2023
**Amendment History:**	Original: 25 September 2019<br>Amendment 1: 18 December 2019<br>Amendment 2: 01 September 2020<br>Amendment 3: 01 February 2022<br>Amendment 4: 18 January 2023<br><br>High-level summaries of the history(ies) of amendment(s) are provided in Appendix 7.
**Country-specific Requirements:**	Country-specific requirements, as applicable, are listed in Appendix 6.

This study will be conducted under US Food & Drug Administration IND regulations (21 CFR Part 312); however, sites located in the European Economic Area and Switzerland are not included under the IND and are considered non-IND sites.

CONFIDENTIALITY STATEMENT

The information contained in this document, particularly unpublished data, is the property or under control of Gilead Sciences, Inc., and is provided to you in confidence as an investigator, potential investigator, or consultant, for review by you, your staff, and an applicable Institutional Review Board or Independent Ethics Committee. The information is only to be used by you in connection with authorized clinical studies of the investigational drug described in the protocol. You will not disclose any of the information to others without written authorization from Gilead Sciences, Inc., except to the extent necessary to obtain informed consent from those persons to whom the drug may be administered.

TABLE OF CONTENTS

TABLE OF CONTENTS 3
LIST OF APPENDICES 5
LIST OF IN-TEXT TABLES 6
LIST OF IN-TEXT FIGURES 6
PROTOCOL SYNOPSIS 7
GLOSSARY OF ABBREVIATIONS AND DEFINITION OF TERMS 17
1. INTRODUCTION 20
 1.1. Background 20
 1.2. GS-6207 20
  1.2.1. General Information 20
  1.2.2. Clinical Studies of GS-6207 21
 1.3. Rationale for This Study 41
 1.4. Rationale for Dose Selection of GS-6207 42
 1.5. Rationale for Oral Weekly Bridging of GS-6207 for Missed SC Injection 43
 1.6. Risk/Benefit Assessment for the Study 44
 1.7. Compliance 44
2. OBJECTIVES 45
3. STUDY DESIGN 46
 3.1. Endpoints 46
 3.2. Study Design 46
 3.3. Study Treatments 48
 3.4. Duration of Treatment 49
 3.5. Biomarker Testing 49
 CCI [redacted]
4. PARTICIPANT POPULATION 51
 4.1. Number of Participants and Participant Selection 51
 4.2. Inclusion Criteria 51
 4.3. Exclusion Criteria 52
5. INVESTIGATIONAL MEDICINAL PRODUCTS 54
 5.1. Randomization, Enrollment, Blinding, and Treatment Codes Access 54
  5.1.1. Randomization and Enrollment 54
  5.1.2. Blinding 54
  5.1.3. Procedures for Breaking Treatment Codes 55
 5.2. Description and Handling of GS-6207 (Lenacapavir) Injection, GS-6207 (Lenacapavir) Tablets and Placebo to Match GS-6207 (Lenacapavir) Tablets 55
  5.2.1. Formulation 55
  5.2.2. Packaging and Labeling 56
  5.2.3. Storage and Handling 56
 5.3. Dosage and Administration of GS-6207 56
 5.4. Dosing and Administration of Oral Weekly Bridging of GS-6207 57
 5.5. Prior and Concomitant Medications 59
 5.6. Accountability for Investigational Medicinal Product 61
  5.6.1. Investigational Medicinal Product Return or Disposal for GS-6207 and Placebo to Match GS-6207 Tablets 61

6. STUDY PROCEDURES 62
6.1. Participant Enrollment and Treatment Assignment 62
6.2. Pre-treatment Assessments 62
6.2.1. Screening Assessments 62
6.3. Treatment Assessments 63
6.3.1. Functional Monotherapy Period and Oral Lead-in Period Assessments 63
6.3.2. Maintenance Period Assessments 64
6.4. Post-Treatment Assessments 66
6.4.1. Early Termination Visit Assessments 66
6.4.2. 30-Day, 90-Day, and 180-Day Follow-Up Visits 68
6.5. Clinical Laboratory Assessments 68
6.5.1. Blood Samples 68
6.5.2. Blood and Urine Storage Samples 70
6.5.3. Urine Samples 71
6.6. Criteria for Discontinuation of Study Treatment 71
6.7. Management of Virologic Failure 71
6.7.1. Suboptimal Virologic Response (SVR) 72
6.7.2. Virologic Rebound (VR) 72
6.7.3. Viremia at Last Visit 72
6.8. End of Study 72
6.9. Post Study Care 73
7. ADVERSE EVENTS AND TOXICITY MANAGEMENT 74
7.1. Definitions of Adverse Events and Serious Adverse Events 74
7.1.1. Adverse Events 74
7.1.2. Serious Adverse Events 74
7.2. Assessment of Adverse Events and Serious Adverse Events 75
7.2.1. Assessment of Causality for Study Drugs and Procedures 75
7.2.2. Assessment of Severity 75
7.3. Investigator Requirements and Instructions for Reporting Adverse Events and Serious Adverse Events 76
7.3.1. Adverse Events 76
7.3.2. Serious Adverse Events 76
7.4. Gilead Reporting Requirements 77
7.5. Clinical Laboratory Abnormalities and Other Abnormal Assessments as Adverse Events or Serious Adverse Events 77
7.6. Toxicity Management 78
7.6.1. Grades 1 and 2 Laboratory Abnormality or Clinical Event 78
7.6.2. Grade 3 Laboratory Abnormality or Clinical Event 78
7.6.3. Grade 4 Laboratory Abnormality or Clinical Event 79
7.6.4. Management of Adverse Events of Injection Site Reactions of Grade 3 or Higher, or Persisting for More Than 26 Weeks 79
7.7. Special Situations Reports 80
7.7.1. Definitions of Special Situations 80
7.7.2. Instructions for Reporting Special Situations 80
7.8. Safety Monitoring Committee 82
8. STATISTICAL CONSIDERATIONS 83
8.1. Analysis Objectives and Endpoints 83
8.1.1. Analysis Objectives 83
8.1.2. Primary Endpoint 83
8.1.3. Secondary Endpoints 83
8.2. Planned Analyses 84

8.2.1. Interim Analysis 84
8.2.2. Primary Analysis 84
8.2.3. Year 2 Analysis 85
8.2.4. Year 3 Analysis 85
8.2.5. Final Analysis 85
8.3. Analysis Conventions 85
8.3.1. Analysis Sets 85
8.3.2. Data Handling Conventions 86
8.4. Demographic and Baseline Characteristics Analysis 86
8.5. Efficacy Analysis 86
8.5.1. Primary Analysis 86
8.5.2. Secondary Analyses 87
8.6. Safety Analysis 88
8.6.1. Extent of Exposure 88
8.6.2. Adverse Events 88
8.6.3. Laboratory Evaluations 89
8.6.4. Other Safety Evaluations 89
8.7. Adjustments for Multiplicity 89
8.8. Pharmacokinetic Analysis 89
8.9. Sample Size 89
8.10. Data Monitoring Committee 90

9. RESPONSIBILITIES 91

9.1. Investigator Responsibilities 91
9.1.1. Good Clinical Practice 91
9.1.2. Good Postmarketing Study Practices (Japan Only) 91
9.1.3. Financial Disclosure 91
9.1.4. Institutional Review Board/Independent Ethics Committee Review and Approval 91
9.1.5. Informed Consent 91
9.1.6. Confidentiality 92
9.1.7. Study Files and Retention of Records 92
9.1.8. Case Report Forms 94
9.1.9. Investigator Inspections 94
9.1.10. Protocol Compliance 94
9.2. Sponsor Responsibilities 94
9.2.1. Protocol Modifications 94
9.2.2. Study Reports and Publications 95
9.3. Joint Investigator/Sponsor Responsibilities 95
9.3.1. Payment Reporting 95
9.3.2. Access to Information for Monitoring 95
9.3.3. Access to Information for Auditing or Inspections 95
9.3.4. Study Discontinuation 95

10. REFERENCES 96

11. APPENDICES 97

LIST OF APPENDICES

Appendix 1. Investigator Signature Page 98
Appendix 2. Study Procedures Table 99
Appendix 3. Management of Clinical and Laboratory Adverse Events 102

Appendix 4. Pregnancy Precautions, Definition for Women of Childbearing Potential, and Contraceptive Requirements....103
Appendix 5. Pandemic Risk Assessment and Mitigation Plan 106
Appendix 6. Country-Specific Requirements 109
Appendix 7. Amendment History 110

LIST OF IN-TEXT TABLES

Table 1-1 GS-US-200-5709: Summary Statistics of GS-6207 Plasma Pharmacokinetic Parameters (Cohort 1) (GS-6207 PK Analysis Set) 23
Table 1-2 GS-US-200-5709: Summary Statistics of GS-6207 Plasma Pharmacokinetic Parameters (Cohort 2) (GS-6207 PK Analysis Set) 24
Table 1-3 GS-US-200-5709: Summary Statistics of GS-6207 Plasma Pharmacokinetic Parameters (Cohort 3) (GS-6207 PK Analysis Set) 25
Table 1-4 GS-US-200-4334: Study Treatments....33
Table 5-1. Oral Dosing of GS-6207 or Placebo to Match 57
Table 5-2. Dosing Schedule for Oral Weekly Bridging and Resumption of SC GS-6207 Injection 58
Table 5-3. Missed Dose Recommendations for GS-6207 During Oral Weekly Bridging....59
Table 5-4. List of Representative Medications that are Prohibited or To Be Used with Caution due to the Potential for Drug-Drug Interaction with GS-6207 60

LIST OF IN-TEXT FIGURES

Figure 1-1 GS-US-200-5709: Mean (SD) GS-6207 Plasma Concentration Versus Time (Cohort 1: Through Day 225) (GS-6207 PK Analysis Set) 22
Figure 1-2 GS-US-200-5709: Mean (SD) GS-6207 Plasma Concentration Versus Time (Cohort 2: Through Day 85) (GS-6207 PK Analysis Set) 24
Figure 1-3 GS-US-200-4334: Study Design 34
Figure 1-4. Simulated Pharmacokinetic Profile of Oral Weekly Bridging of GS-6207 (300 mg) (A) Prior to and (B) After Resuming SC Injection....43

PROTOCOL SYNOPSIS

Gilead Sciences, Inc.
333 Lakeside Drive
Foster City, CA 94404

**Study Title:**	A Phase 2/3 Study to Evaluate the Safety and Efficacy of Long-Acting Capsid Inhibitor GS-6207 in Combination With an Optimized Background Regimen in Heavily Treatment Experienced People Living With HIV-1 Infection With Multidrug Resistance
**IND Number:**<br>**EU CT Number:**<br>**Clinical Trials.gov Identifier:**	136260<br>2019-003814-16<br>04150068
**Study Centers Planned:**	Approximately 75 centers globally
**Objectives:**	The primary objective of this study is:<br>• To evaluate the antiviral activity of GS-6207 (Lenacapavir, LEN) administered as an add-on to a failing regimen (functional monotherapy) for people living with HIV (PLWH) with multidrug resistance as determined by the proportion of participants achieving at least 0.5 $\log_{10}$ reduction from baseline in HIV-1 RNA at the end of the Functional Monotherapy Period<br>The secondary objectives of this study are:<br>• To evaluate the safety and efficacy of GS-6207 in combination with an optimized background regimen (OBR) at Weeks 26 and 52<br>• To evaluate the safety and efficacy of GS-6207 in combination with an OBR at Weeks 104 and 156 (from the first subcutaneous [SC] dose of GS-6207)<br>CCI [redacted]<br>[redacted]<br>[redacted]

	• CCI [redacted]<br>CCI [redacted]
**Study Design:**	This is a global multicenter study of GS-6207 together with an OBR in people living with HIV with multidrug resistant infection.<br>Participants who complete a Screening visit will return to the clinic between 14 and 30 days after the Screening visit, for a Cohort Selection visit. HIV-1 RNA results from this Cohort Selection visit will be used to determine whether eligible participants will participate in Cohort 1 or Cohort 2.<br>**Cohort 1 (n=36)**<br>**Functional Monotherapy Period**<br>Eligible participants with a $< 0.5 \log_{10}$ HIV-1 RNA decline compared to the Screening visit and HIV-1 RNA ≥ 400 copies/mL at the Cohort Selection visit will be randomized, in a blinded fashion, in a 2:1 ratio to receive either oral GS-6207 or placebo to match GS-6207 for 14 days. Treatment assignment will be blinded to the Sponsor, participants, investigators, and study staff at the site. Functional Monotherapy will be assessed while participants continue their failing regimen.<br>After each participant completes the Functional Monotherapy Period, their treatment assignment will be unblinded.<br>**Maintenance Period**<br>Participants who were randomized to receive oral GS-6207 will receive SC GS-6207 and initiate their OBR on Day 1 SC (14 days after the first dose of oral GS-6207) **(Cohort 1A)**.<br>Participants who were randomized to receive placebo will receive oral GS-6207 and initiate their OBR on Day 15 **(Cohort 1B)**. They will receive SC GS-6207 at Day 1 SC (eg, 14 days after the first dose of oral GS-6207) while continuing their OBR.

	After the Day 1 SC visit, all Cohort 1 participants will continue with study visits at Weeks 4, 10, 16, 22, 26, 36, and 52 (study visits Week X are identified by the number of weeks that have elapsed since the Day 1 SC visit when GS-6207 is first administered by injection and excludes Oral Bridging Periods).<br><br>Participants will receive their subsequent SC GS-6207 injection at the Week 26 visit. At the Week 52 visit, participants will be given an option to receive SC GS-6207 injection and continue on the study to receive SC GS-6207 injections once every 6 months (26 weeks) ± 2 weeks from last injection.<br><br>**<u>Cohort 2 (n=64)</u>**<br><br>**Oral Lead-in Period**<br><br>Participants will be enrolled into Cohort 2 if Cohort 1 is fully enrolled or if they do not meet the criteria for randomization in Cohort 1 (ie, they had ≥ 0.5 $\log_{10}$ HIV-1 RNA decline compared to the Screening visit and/or HIV-1 RNA < 400 copies/mL at the Cohort Selection visit). All Cohort 2 participants will receive oral GS-6207 for 14 days starting at Day 1. Participants will initiate an OBR on Day 1.<br><br>**Maintenance Period**<br><br>At Day 1 SC (ie, 14 days after the first dose of oral GS-6207), participants will receive SC GS-6207 and will continue their OBR. After the Day 1 SC visit, participants will continue with study visits at Weeks 4, 10, 16, 22, 26, 36, and 52 (study visits Week X are identified by the number of weeks that have elapsed since the Day 1 SC visit when GS-6207 is first administered by injection and excludes Oral Bridging Periods). Participants will receive their subsequent SC GS-6207 injection at the Week 26 visit. At the Week 52 visit, participants will be given an option to receive SC GS-6207 injection and continue the study to receive SC GS-6207 injections once every 6 months (26 weeks) ± 2 weeks from last injection.
Number of Participants Planned:	Approximately 100 participants may be enrolled in this study.<br><br>36 participants will be enrolled in Cohort 1. Up to 64 participants may be enrolled in Cohort 2.

Target Population:	Heavily treatment experienced adults and adolescent PLWH with resistance to ≥ 2 antiretroviral medications from each of ≥ 3 of the 4 main classes of antiretroviral medication and plasma HIV-1 RNA ≥ 400 copies/mL at Screening while taking a failing regimen.<br>Adolescent participants will be enrolled only at sites in North America and Dominican Republic.
Duration of Treatment:	Duration of treatment is at least 54 weeks.<br>Following successful completion of Week 52 visit, participants will be given the option to attend visits at Week 62, 78, 88, 104, 114, 130 and will continue to alternate between every 10 weeks and every 16 weeks. Participants willing to continue the study beyond Week 52 will receive SC GS-6207 927 mg once every 6 months (26 weeks) ± 2 weeks from last injection starting at Week 52 visit, while continuing their OBR, until the product becomes accessible to participants through an access program, is commercially available, or until Gilead Sciences elects to discontinue the study in the country.<br>Participants who decide not to receive SC GS-6207 at Week 52 and not to continue the study will complete the study at Week 52 visit.<br>Participants who decide to discontinue SC GS-6207 early and do not wish to continue to attend study visits through the Week 52 visit or the next scheduled SC dosing visit will return to the clinic for 30-Day, 90-Day, and 180-Day Follow-Up visits after the Early Termination visit. The 180-Day Follow-Up may be conducted via a phone call per the investigator’s discretion.
Diagnosis and Main Eligibility Criteria:	People living with HIV who meet the following criteria:<br>• Adults and adolescents aged ≥ 12 and weighing ≥ 35 kg<br>• Are receiving a stable failing regimen for > 8 weeks before Screening and are willing to continue that regimen until Day 1. Cohort 1 participants should be willing to continue their failing regimen until Day 14<br>• Have HIV-1 RNA ≥ 400 copies/mL at Screening<br>• Have resistance to ≥ 2 antiretroviral medications from each of ≥ 3 of the 4 main classes of antiretroviral medications (NRTI, NNRTI, PI, INSTI). Resistance to FTC or 3TC associated with the presence of the M184V/I RT mutation cannot be used for the purpose of determining eligibility for this criterion

	• Have ≤ 2 fully active antiretroviral remaining from the 4 main classes that can be effectively combined to form a viable regimen in the opinion of the investigator based on resistance, tolerability, contraindication, safety, drug access, or acceptability to the participant<br>• Able and willing to receive an OBR together with GS-6207
Study Procedures/ Frequency:	At Screening, laboratory analyses (hematology, chemistry and urinalysis, and serum pregnancy test [for women]), HIV-1 RNA, CD4+ cell count, vital signs, electrocardiogram (ECG), complete physical examination and estimated glomerular filtration rate will be performed, and HBV and HCV serologies will be analyzed. Laboratory samples for resistance analysis will be collected. Analysis of the participant's HIV-1 resistance to support eligibility will be completed.<br>Participants who complete a Screening visit will return to the clinic between 14 and 30 days after the Screening visit for a Cohort Selection visit. HIV-1 RNA results from this Follow-Up visit will be used to randomize participants in Cohort 1 or enroll them in Cohort 2.<br>• Cohort 1 (n=36): Participants in Cohort 1 will be randomized to receive oral GS-6207 or placebo to match GS-6207 during the Functional Monotherapy Period while continuing their failing regimen. During the Maintenance Period, they will receive SC GS-6207 and an OBR.<br>• Cohort 2 (n=64): Participants in Cohort 2 will be enrolled to receive oral GS-6207 and an OBR in the Oral Lead-in Period. During the Maintenance Period they will receive SC GS-6207 and continue their OBR.<br>**Functional Monotherapy Period (Cohort 1 only)**<br>Participants with both < 0.5 $\log_{10}$ HIV-1 RNA decline compared to the Screening visit and HIV-1 RNA ≥ 400 copies/mL at the Cohort Selection visit will be randomized to receive oral GS-6207 or placebo to match GS-6207 while continuing their failing regimen. Sites, participants, and Sponsor will be blinded to the participant treatment assignment.

	Participants will visit the clinic on Days 1, 2, 5 (±1) (if possible) and 8. On Day 1 before dosing, baseline assessments will be conducted including adverse events (AEs), concomitant medications, laboratory assessments and physical examinations as detailed in the Study Procedures Table (Appendix 2). After completion of the assessments, the participant will receive oral GS-6207 600 mg, 600 mg, and 300 mg or placebo to match GS-6207 on Days 1, 2, and 8, respectively.<br>After each participant completes the Functional Monotherapy Period, their treatment assignment will be unblinded.<br>**Maintenance Period (Cohort 1A)**<br>Participants who received oral GS-6207 during the Functional Monotherapy Period will receive SC GS-6207 927 mg and initiate an OBR at the Day 1 SC visit (14 days after the first dose of oral GS-6207). They will continue with study visits at Weeks 4, 10, 16, 22, 26, 36, and 52. Participants will receive their subsequent SC GS-6207 injection at the Week 26 visit. At the Week 52 visit, participants will be given an option to receive SC GS-6207 injection and continue on the study to receive SC GS-6207 injections once every 6 months (26 weeks) ± 2 weeks from last injection.<br>**Maintenance Period (Cohort 1B)**<br>Participants who received placebo to match GS-6207 during the Functional Monotherapy Period (Day 1 to Day 14) will visit the clinic on Days 15, 16, 19 (±1) (if possible) and 22. They will receive oral GS-6207 600 mg and initiate their OBR on Day 15. After completing study visit assessments, participants will receive additional oral GS-6207 600 mg and 300 mg on Days 16 and Day 22, respectively.<br>Participants will receive SC GS-6207 927 mg at the Day 1 SC visit (14 days after the first dose of oral GS-6207) while continuing their OBR. They will continue with study visits at Weeks 4, 10, 16, 22, 26, 36, and 52. Participants will receive their subsequent SC GS-6207 927 mg injection at the Week 26 visit.<br>**Oral Lead-in (Cohort 2 only)**<br>Participants will be enrolled into Cohort 2 if Cohort 1 is fully enrolled or if they do not meet the criteria for randomization in Cohort 1 (ie, they had ≥ 0.5 $\log_{10}$ HIV-1 RNA decline compared to the Screening visit and/or HIV-1 RNA < 400 copies/mL at the Cohort Selection visit). Participants will be enrolled in Cohort 2 to initiate oral GS-6207. Participants will initiate their OBR on Day 1.

	Participants will visit the clinic on Days 1, 2, 5 (±1) and 8 during the Oral Lead-in Period.<br><br>On Day 1 before dosing, baseline assessments will be conducted including AEs, concomitant medications, laboratory tests and physical examinations as detailed in the Study Procedures Table (Appendix 2). After completion of the assessments, the participant will receive oral GS-6207 600 mg, 600 mg, and 300 mg on Days 1, 2, and 8, respectively.<br><br>**Maintenance Period (Cohort 2)**<br><br>Participants will receive SC GS-6207 927 mg at Day 1 SC visit (14 days after the first dose of oral GS-6207) while continuing their OBR. They will continue with study visits at Weeks 4, 10, 16, 22, 26, 36, and 52. Participants will receive their subsequent SC GS-6207 927 mg injection at the Week 26 visit.<br><br>**<u>Cohort 1 and Cohort 2</u>**<br><br>At the Week 52 visit, all participants (Cohorts 1 and 2) will be given an option to continue to complete study visits at Week 62, 78, 88, 104, 114, 130 and will continue to alternate between every 10 weeks and every 16 weeks. Participants willing to continue beyond Week 52 visit will receive SC GS-6207 927 mg once every 6 months (26 weeks) ± 2 weeks from last injection starting at Week 52 visit.<br><br>At each visit, AEs, concomitant medications, laboratory tests and physical examinations will be performed in accordance with the Study Procedures Table (Appendix 2).<br><br>Participants may require oral weekly bridging 300 mg if an SC injection of GS-6207 cannot be administered for any reason within the protocol visit window.<br><br>**Resistance Analysis:**<br><br>Plasma samples for genotypic and phenotypic testing of HIV-1 will be collected in accordance with the Study Procedures Table (Appendix 2). Screening results and/or historic HIV-1 resistance reports will be used to determine eligibility and to help construct the OBR.

	**Pharmacokinetic Assessments:**<br>PK sampling will occur relative to dosing of GS-6207 at the following time points for all participants:<br>• Day 1: Predose (within 30 minutes of dosing), 1, 2, 4, 6, and 8 hours post dose<br>• Day 2 and 8:<br>— Predose (within 30 minutes of dosing)<br>— A single timed PK sample between 1 and 6 hours post dose<br>• Day 5 (if visit occurs): A single anytime PK sample<br>• Day 15 (for Cohort 1B participants only): Predose (within 30 minutes of dosing), 1, 2, 4, 6 and 8 hours post dose<br>• Day 16 and 22 (for Cohort 1B participants only):<br>— Predose (within 30 minutes of dosing)<br>— A single timed PK sample between 1 and 6 hours post dose<br>• Day 19 (for Cohort 1B participants only, if visit occurs): A single anytime PK sample<br>• During the Maintenance Period, at all visits without SC GS-6207 injections, including the Oral Bridging visits, if applicable: A single anytime PK sample will be collected<br>• During the Maintenance Period, at all visits with SC GS-6207 injections, including when SC GS-6207 injection is resumed after the Oral Bridging visits, if applicable: A single predose (within 30 minutes of dosing) PK sample will be collected<br>**Patient reported outcomes (PROs):** Participants ≥ 18 years of age will complete the following, if available**:**<br>• Symptoms Distress Module, Short Form Health Survey (SF-36), EQ-5D-5L at Day 1, Weeks 4, 16, 26, and 52<br>• The Numeric Pain Rating Scale at Day 1 SC, Weeks 26 and 52
**Test Product, Dose, and Mode of Administration:**	Oral GS-6207 and SC GS-6207 injection. Study drug (oral and SC GS-6207) will be administered without regard to food.

**Reference Therapy, Dose, and Mode of Administration:**	Placebo to match GS-6207 (during the randomized Functional Monotherapy Period)<br>None (during the Maintenance Period)
**Criteria for Evaluation:**	
Safety:	Incidence of treatment-emergent AEs and clinical laboratory abnormalities
Efficacy:	The primary endpoint is:<br>• The proportion of participants in Cohort 1 achieving ≥ 0.5 log_{10} copies/ml reduction from baseline in HIV-1 RNA at the end of the Functional Monotherapy Period.<br>The secondary endpoints are:<br>• The proportion of participants in Cohort 1 with plasma HIV-1 RNA < 50 copies/mL and < 200 copies/mL at Weeks 26 and 52 visits based on the United States (US) Food and Drug Administration (FDA)-defined snapshot algorithm.<br>• The proportion of participants in combined Cohorts 1 and 2 with plasma HIV-1 RNA < 50 copies/mL and < 200 copies/mL at Weeks 104 and 156 from the first SC dose of GS-6207 based on the US FDA-defined snapshot algorithm.
**Statistical Methods:**	The primary efficacy analysis is to compare the proportion of participants in Cohort 1 achieving ≥ 0.5 log_{10} reduction from baseline in HIV-1 RNA at the end of the Functional Monotherapy Period. The p-value and 95% confidence interval for the difference in response rates between two treatment groups (GS-6207 in Cohort 1A and placebo in Cohort 1B) will be estimated and constructed based on an unconditional exact method using 2 invert 1-sided tests with an alpha level at 0.05.<br>The proportion of participants in Cohort 1 with HIV-1 RNA < 50 copies/mL and < 200 copies/mL at Weeks 26 and 52, and the proportion of participants in combined Cohorts 1 and 2 with HIV-1 RNA < 50 copies/mL and < 200 copies/mL at Weeks 104 and 156 (from the first SC dose of GS-6207) will be summarized using descriptive statistics based on the US FDA-defined snapshot algorithm.

	Incidence of treatment-emergent AEs and treatment-emergent laboratory abnormalities will be summarized.<br><br>A total of 36 participants in Cohort 1 will provide at least 90% power to detect a 60% difference in the proportion of participants achieving a $\geq 0.5 \log_{10}$ reduction from baseline at the end of the Functional Monotherapy Period between treatment groups (GS-6207 in Cohort 1A and placebo in Cohort 1B). In this sample size and power computation, it is assumed that 70% and 10% of participants achieve a $\geq 0.5 \log_{10}$ reduction from baseline in HIV-1 RNA in the GS-6207 group (Cohort 1A) and the placebo group (Cohort 1B) (based on data from Trogarzo Phase 3 TMB-301 study {Emu 2018}), respectively, and the Fisher exact test is conducted at 2-sided significant level of 0.05.<br><br>A total sample size of 36 participants from Cohort 1A and Cohort 1B will provide reasonable assessment of safety for at least 26 weeks of treatment in heavily treatment experienced participants.<br><br>The external multidisciplinary Data Monitoring Committee will review the progress, efficacy, and safety data after all participants in Cohort 1 have completed 14 days of assessment in the Functional Monotherapy Period or discontinued the study drug. Further enrollment will be stopped if 50% or more of the participants in the GS-6207 group fail to achieve at least 0.5 $\log_{10}$ reduction from baseline in HIV-1 RNA at the end of the Functional Monotherapy Period. The decision whether to continue with the study and the development of GS-6207 will be based on the magnitude of the HIV-1 RNA decline at the end of the Functional Monotherapy Period.

This study will be conducted in accordance with the guidelines of Good Clinical Practice (GCP) and Good Postmarketing Study Practice (GPSP) after approval of GS-6207 in Japan including archiving of essential documents.

“Clinical trial” shall read “postmarketing clinical trial” after approval of GS-6207 in Japan.

GLOSSARY OF ABBREVIATIONS AND DEFINITION OF TERMS

λ_z	terminal elimination rate constant, estimated by linear regression of the terminal elimination phase of the log concentration versus time curve of the drug
$\%AUC_{exp}$	percentage of AUC extrapolated between AUC_{last} and AUC_{inf}
%CV	percentage coefficient of variation
AE	adverse event
AIDS	acquired immunodeficiency syndrome
ALT	alanine aminotransferase
ART	antiretroviral therapy
ARV	Antiretroviral
AST	aspartate aminotransferase
AUC	area under the concentration versus time curve
AUC_{inf}	area under the concentration versus time curve extrapolated to infinite time, calculated as $AUC_{last} + (C_{last}/\lambda_z)$
AUC_{last}	area under the concentration versus time curve from time zero to the last quantifiable concentration
BCRP	breast cancer resistance protein
BUN	blood urea nitrogen
CAI	capsid inhibitor
CBC	complete blood count
CFR	Code of Federal Regulations
CI	confidence interval
CK	creatine kinase
CL/F	apparent oral clearance after administration of the drug:<br>$CL/F = Dose/AUC_{inf}$, where "Dose" is the dose of the drug
C_{last}	last observed quantifiable concentration of the drug
CL_{cr}	creatinine clearance
C_{max}	maximum observed concentration of drug
CPK	creatine phosphokinase
CRF	case report form
CRO	contract (or clinical) research organization
CSR	clinical study report
DMC	data monitoring committee
DNA	deoxyribonucleic acid
ECG	Electrocardiogram
EFV	Efavirenz
eCRF	electronic case report form
EDC	electronic data capture
EU CT	European Clinical Trials Database
eSAE	electronic serious adverse event

ET	early termination
EU	European Union
FDA	Food and Drug Administration
FSH	follicle-stimulating hormone
GCP	Good Clinical Practice
GFR	glomerular filtration rate
Gilead	Gilead Sciences, Inc.
GPSP	Good Postmarketing Study Practice
HBV	hepatitis B virus
HBsAb	hepatitis B surface antibody
HBSAg	hepatitis B surface antigen
HCV	hepatitis C virus
HDPE	high-density polyethylene
HIV, HIV-1	human immunodeficiency virus, type 1
IB	investigator’s brochure
ICF	informed consent form
ICH	International Council on Harmonisation (of Technical Requirements for Registration of Pharmaceuticals for Human Use)
IND	investigational new drug (application)
IQ	inhibitory quotient
IRB	institutional review board
IUD	intrauterine device
LEN	Lenacapavir
LLOQ	lower limit of quantitation
MedDRA	Medical Dictionary for Regulatory Activities
MDR	multi-drug resistance
NaS	Sodium salt
OBR	Optimized Background Regimen
PG	Pharmacogenomics
PI	principal investigator
PIT	Pitavastatin
PK	pharmacokinetic(s)
PLWH	people living with HIV
PRO	Patient Reported Outcomes
GLPS	Global Patient Safety (formerly Pharmacovigilance and Epidemiology (PVE))
QA	quality assurance
QD	Once Daily
RNA	ribonucleic acid
SADR	serious adverse drug reaction
SAE	serious adverse event

SAP	statistical analysis plan
SC	subcutaneous
SD	standard deviation
SOC	system organ class
SOP	standard operating procedure
SUSAR	suspected unexpected serious adverse reaction
SVR	suboptimal virologic response
TEAE	treatment-emergent adverse event
T_{last}	time (observed time point) of C_{last}
T_{max}	the time (observed time point) of C_{max}
$t_{1/2}$	estimate of the terminal elimination half-life of the drug, calculated by dividing the natural log of 2 by the terminal elimination rate constant (λ_z)
ULN	upper limit of normal
US	United States
VR	Virologic Rebound
WHO	World Health Organization

1. INTRODUCTION

1.1. Background

Human Immunodeficiency Virus (HIV)-1 infection is a life-threatening and serious disease of major public health significance, with approximately 37 million people living with HIV (PLWH) worldwide and approximately 16 million on antiretroviral (ARV) treatment {UNAIDS 2022}. Advances in combination antiretroviral therapy (ART) for HIV have led to significant improvements in morbidity and mortality by suppressing viral replication, preserving immunologic function, and averting disease progression to acquired immunodeficiency syndrome (AIDS). Standard-of-care for the treatment of HIV-1 infection involves the use of a combination of oral ARV drugs (eg, 2 NRTIs plus a third agent) to suppress viral replication to below detectable limits, increase CD4+ cell counts, and delay disease progression.

While combination ARV therapy for the treatment of HIV-1 infection is efficacious and well tolerated, these agents need to be taken every day and require near perfect adherence to minimize the emergence of drug resistant variants. In addition, "treatment fatigue" can occur, defined as "decreased desire and motivation to maintain vigilance in adhering to a treatment regimen" among PLWH prescribed chronic or life-long treatment {Claborn 2015}, which can lead to nonadherence and treatment failure. As such, there remains a significant medical need for ARVs that can be administered less frequently (eg, long-acting drug products), thereby providing an alternative treatment option for PLWH.

1.2. GS-6207

GS-6207 is a novel, first-in-class, selective inhibitor of HIV-1 capsid function, which has potent antiviral activity, low human clearance, and physicochemical properties well suited for extended-release parenteral or oral formulations. GS-6207 has been assigned the International Nonproprietary Name Lenacapavir (LEN).

1.2.1. General Information

For further information on GS-6207, please refer to the Investigator's Brochure (IB). Information in the IB includes:

- Nonclinical pharmacokinetic (PK) and in vitro metabolism
- Nonclinical pharmacology and toxicology
- Clinical experience

1.2.2. Clinical Studies of GS-6207

A summary of the relevant available data from 4 Phase 1 clinical studies in healthy volunteers (GS-US-200-4071, GS-US-200-4333, and GS-US-200-4538) and 1 study in PLWH (GS-US-200-4072) are presented in the IB. Participants in the GS-US-200-4072 study received injections of a subcutaneous (SC) GS-6207 suspension formulation whereas participants in the GS-US-200-4071 and GS-US-200-4538 studies used the tablet and SC solution formulations.

1.2.2.1. GS-US-200-5709

Study GS-US-200-5709 is an ongoing, Phase 1, open-label, multicohort, multidose oral and/or SC LEN study to evaluate the safety, tolerability, and PK of multiple-dose oral and SC LEN in healthy participants.

Herein is a summary from the 2 clinical study reports (CSRs): interim analysis conducted on final data from Cohort 3 and the CSR addendum of interim analysis with updated PK (through Day 225 PK assessments for Cohort 1 and through Day 85 PK assessments for Cohort 2) and updated safety analyses (through 25 June 2021) for the ongoing cohorts (Cohort 1 and 2) in the study.

Preliminary Pharmacokinetic Results

Interim PK data through Day 225 for Cohort 1 after administration of oral GS-6207 600 mg on Days 1 and 2, oral GS-6207 300 mg on Day 8, and SC GS-6207 927 mg on Day 15, are presented in Figure 1-1. Intensive PK sampling was conducted on Days 1, 2, 8, and 15, and single anytime PK samples were collected at prespecified study visits through Day 225. The GS-6207 PK parameters for Days 1, 2, 8, and 15 are summarized in Table 1-1. Mean GS-6207 plasma concentrations and their lower bound 90% CIs achieved IQ4 (4-fold higher than $paEC_{95}$) within 2 hours postdose on Day 2, and this was maintained through Week 30.

Figure 1-1 GS-US-200-5709: Mean (SD) GS-6207 Plasma Concentration Versus Time (Cohort 1: Through Day 225) (GS-6207 PK Analysis Set)

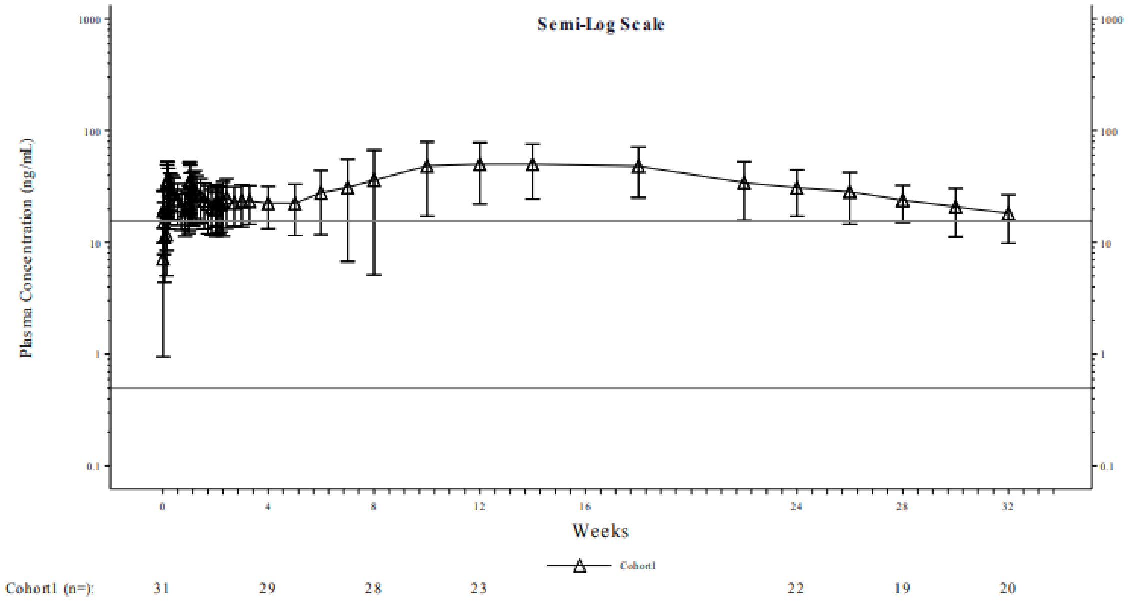

BLQ = below the limit of quantitation; IQ4 = inhibitory quotient of 4; GS-6207 = lenacapavir; LLOQ = lower limit of quantitation; PK = pharmacokinetic(s)
Cohort 1 dosing was oral GS-6207 600 mg on Days 1 and 2, oral GS-6207 300 mg on Day 8, and SC GS-6207 927 mg on Day 15.
Values BLQ were treated as 0 for predose and one-half the LLOQ for postdose summaries.
LLOQ was defined as 0.5 ng/mL for GS-6207.
Postdose concentration values ≤ LLOQ were not presented on the figure.
Figures on the semi-log scale were set to include all lower bar and mean/median values > 0 on the Y-axis.
Reference lines indicate IQ4 (15.5 ng/mL) and LLOQ (0.5 ng/mL)

Table 1-1 GS-US-200-5709: Summary Statistics of GS-6207 Plasma Pharmacokinetic Parameters (Cohort 1) (GS-6207 PK Analysis Set)

	GS-6207			
**PK Parameter Mean (%CV)**	**Day 1 600 mg oral GS-6207 (2 × 300 mg tablets) (N = 31)**	**Day 2 600 mg oral GS-6207 (2 × 300 mg tablets) (N = 31)**	**Day 8 300 mg oral GS-6207 (1 × 300 mg tablet) (N = 31)**	**Day 15 927 mg SC GS-6207 (2 × 1.5 mL 309 mg/mL NaS) (N = 30)**
C_{max} (ng/mL)	22.0 (45.5)	40.4 (43.4)	39.3 (44.7)	58.7 (58.1)
T_{max} (h)[a] [days]	4.00 (4.00, 6.00) [0.17]	6.00 (4.00, 8.00) [0.25]	6.00 (4.00, 8.00) [0.25]	2028.04 (1682.45, 2688.23) [84.5]
C_{last} (ng/mL)	11.2 (60.8)	19.2 (40.8)	20.6 (43.1)	25.0 (81.7)
T_{last} (h)[a] [days]	12.00 (12.00, 12.00) [0.5]	120.00 (120.00, 120.00) [5.0]	144.00 (144.00, 144.00) [6.0]	5037.95 (4032.00, 5042.33) [209.9]

%CV = percentage coefficient of variation; BLQ = below the limit of quantitation; GS-6207 = lenacapavir; LLOQ = lower limit of quantitation; NaS = sodium salt; PK = pharmacokinetics; Q1 = first quartile; Q3 = third quartile; SC = subcutaneous
Median (Q1, Q3).
Values BLQ were treated as 0 for predose and one-half of the LLOQ for postdose summaries.
LLOQ was defined as 0.5 ng/mL for GS-6207.

Interim PK data through Day 85 for Cohort 2 after administration of SC GS-6207 927 mg and oral GS-6207 600 mg on Day 1, followed by oral GS-6207 600 mg on Day 2, are presented in Figure 1-2. Intensive PK sampling was conducted on Days 1 and 2, and single anytime PK samples were collected at prespecified study visits through Day 85. The GS-6207 PK parameters for Days 1 and 2 are summarized in Table 1-2. Mean GS-6207 plasma concentrations and their lower bound 90% CIs achieved IQ4 within 2 hours postdose on Day 2, and this was maintained through Day 85. From Days 0 to 85, mean $AUC_{0\text{-}85d}$ (%CV) of 83,082.9 h•ng/mL (43.6%) was generally comparable to Cohort 1 (68,364 h•ng/mL [49.8%]).

Figure 1-2 GS-US-200-5709: Mean (SD) GS-6207 Plasma Concentration Versus Time (Cohort 2: Through Day 85) (GS-6207 PK Analysis Set)

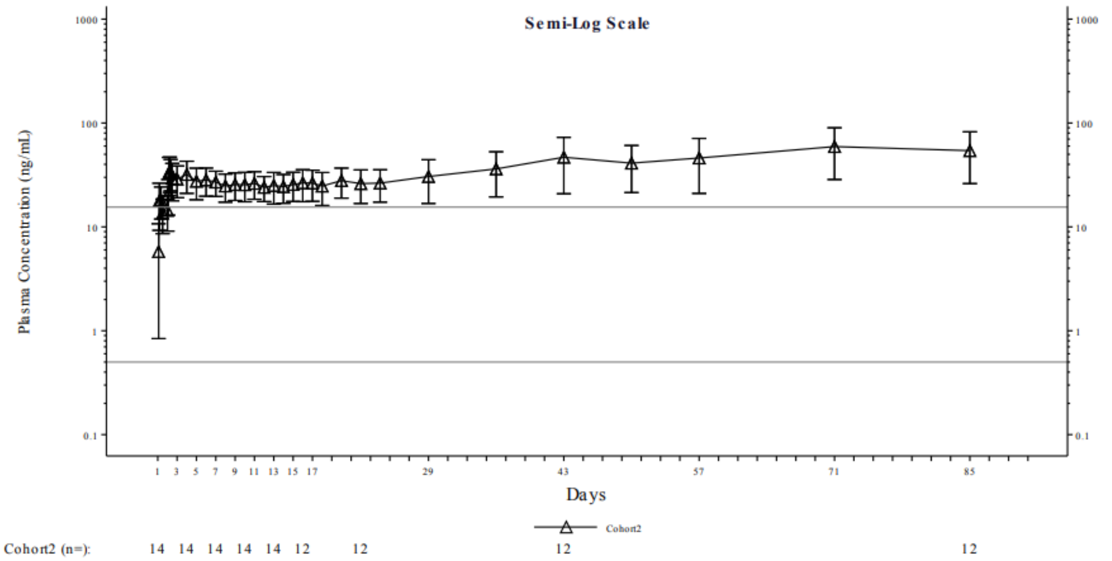

BLQ = below the limit of quantitation; IQ4 = inhibitory quotient of 4; GS-6207 = lenacapavir; LLOQ = lower limit of quantitation; PK = pharmacokinetic(s)
Cohort 2 dosing was SC GS-6207 927 mg and oral GS-6207 600 mg on Day 1 and oral GS-6207 600 mg on Day 2.
Values BLQ were treated as 0 for predose and one-half the LLOQ for postdose summaries.
LLOQ was defined as 0.5 ng/mL for GS-6207.
Postdose concentration values ≤ LLOQ were not presented on the figure.
Figures on the semi-log scale were set to include all lower bar and mean/median values > 0 on the Y-axis.
Reference lines indicate IQ4 (15.5 ng/mL) and LLOQ (0.5 ng/mL)

Table 1-2 GS-US-200-5709: Summary Statistics of GS-6207 Plasma Pharmacokinetic Parameters (Cohort 2) (GS-6207 PK Analysis Set)

	**GS-6207**	
**PK Parameter**<br>**Mean (%CV)**	**Day 1**<br>**927 mg SC GS-6207 (2 × 1.5 mL 309 mg/mL NaS)**<br>**600 mg Oral GS-6207 (2 × 300 mg Tablets)**<br>**(N = 14)**	**Day 2**<br>**600 mg Oral GS-6207**<br>**(2 × 300 mg Tablets)**<br>**(N = 14)**
C_{max} (ng/mL)	19.9 (35.5)	64.7 (45.0)
T_{max} (h)[a]<br>[days]	6.00 (4.00, 8.00)<br>[0.25]	1653.74 (12.00, 1653.98)<br>[68.9]
C_{last} (ng/mL)	13.6 (36.5)	53.8 (54.2)
T_{last} (h)[a]<br>[days]	12.0 (12.0, 12.0)<br>[0.50]	1991.16 (1990.70, 1991.18)<br>[83.0]

%CV = percentage coefficient of variation; BLQ = below the limit of quantitation; GS-6207 = lenacapavir; LLOQ = lower limit of quantitation; NaS = sodium salt; PK = pharmacokinetics; Q1 = first quartile; Q3 = third quartile; SC = subcutaneous
Median (Q1, Q3).
Values BLQ were treated as 0 for predose and one-half of the LLOQ for postdose summaries.
LLOQ was defined as 0.5 ng/mL for GS-6207.

Final PK data from Cohort 3 after administration of oral GS-6207 600 mg twice daily (Days 1 through 10), followed by an oral GS-6207 600 mg dose in the morning on Day 11, are presented in Table 1-3. Intensive PK sampling (from 0 to 12 hours) was conducted on Days 1, 2, and 11 with sparse sampling on Days 3, 4, 8, 9, and 10. As shown, GS-6207 exhibited significant accumulation over the course of 11 days of dosing, which is consistent with its long $t_{1/2}$.

Table 1-3 GS-US-200-5709: Summary Statistics of GS-6207 Plasma Pharmacokinetic Parameters (Cohort 3) (GS-6207 PK Analysis Set)

PK Parameter Mean (%CV)	GS-6207							
	600 mg oral GS-6207 (2 × 300 mg tablets; AM dose) and 600 mg oral GS-6207 (2 × 300 mg tablets; PM dose)							600 mg oral GS-6207 (2 × 300 mg tablets; AM dose)
	Day 1 (N = 15)	Day 2 (N = 15)	Day 3 (N = 15)	Day 4 (N = 15)	Day 8 (N = 15)	Day 9 (N = 15)	Day 10 (N = 15)	Day 11 (N = 15)
AUC_{0-12h} (h•ng/mL)	135.4 (30.7)	894.4 (47.2)	NC	NC	NC	NC	NC	9706.9 (28.2)
C_{max} (ng/mL)	19.0 (24.0)	96.4 (49.5)	NC	NC	NC	NC	NC	1012.1 (26.2)
T_{max} (h)[a]	5.00 (4.00, 5.00)	8.00 (5.00, 11.97)	NC	NC	NC	NC	NC	4.00 (4.00, 5.00)
C_{last} (ng/mL)	11.1 (39.9)	88.4 (45.2)	241.6 (41.2)	376.9 (31.7)	604.8 (32.6)	680.5 (28.2)	751.9 (25.7)	787.7 (31.3)
C_{last} (h)[a]	11.97 (11.97, 11.97)	11.97 (11.97, 11.97)	11.97 (11.97, 11.97)	11.97 (11.97, 11.97)	11.97 (11.97, 11.97)	11.97 (11.97, 11.97)	11.97 (11.97, 11.97)	11.97 (11.97, 11.97)
C_0 (ng/mL)	BLQ (BLQ)	42.0 (47.2)	152.6 (49.7)	342.4 (39.6)	558.5 (36.8)	699.9 (33.2)	693.1 (35.0)	788.8 (32.1)

BLQ = below the limit of quantitation; %CV = percentage coefficient of variation; GS-6207 = lenacapavir; LLOQ = lower limit of quantitation; NC = not calculated due to insufficient PK sampling; PK = pharmacokinetics; Q1 = first quartile; Q3 = third quartile
a Median (Q1, Q3).
C_0 represents concentration at time 0 hours.
Values BLQ were treated as 0 for predose and one-half the LLOQ for postdose summaries.
LLOQ was defined as 0.5 ng/mL for GS-6207.

Preliminary Safety Results

Interim safety data through 25 June 2021 for Cohorts 1 and 2 and final safety data for Cohort 3 are summarized below.

A total of 31 participants were enrolled into Cohort 1 and received at least 1 dose of study drug. Overall, GS-6207 was generally safe and well tolerated. No Grade 3 or 4 adverse events (AEs), SAEs, deaths, or pregnancies were reported. One participant (3.2%) prematurely discontinued study drug and the study due to a Grade 1 AE of SARS-CoV-2 test positive. Five participants (16.1%) had Grade 3 laboratory abnormalities (increased fasting triglycerides [2 participants] and increases in CK, fasting urine glucose, and fasting creatinine [1 participant each]), and 1 participant (3.2%) had a Grade 4 laboratory abnormality (increased fasting triglycerides).

A total of 14 participants were enrolled into Cohort 2 and received at least 1 dose of study drug. Overall, GS-6207 was generally safe and well tolerated. No Grade 3 or 4 AEs, SAEs, AEs leading to study drug or study discontinuation, deaths, or pregnancies were reported. No participant had Grade 4 laboratory abnormalities. Three participants (21.4%) had Grade 3 laboratory abnormalities (increased fasting cholesterol and LDL cholesterol [1 participant] and increases in CK and fasting triglycerides [1 participant each]).

No participant in Cohort 1 or 2 had notable changes in vital signs (systolic blood pressure, diastolic blood pressure, pulse, and temperature) or clinically significant electrocardiogram (ECG) abnormalities.

A total of 15 participants were enrolled in Cohort 3 and completed GS-6207 600 mg twice daily dosing for 11 days, with the last dose given in the morning on the 11th day. Overall, GS-6207 was generally safe and well tolerated at clinically relevant therapeutic and supratherapeutic exposures. Despite the supratherapeutic exposures observed, no Grade 3 or 4 AEs, SAEs, AEs leading to study drug or study discontinuation, deaths, or pregnancies were reported. One participant (6.7%) experienced an AE of dyspepsia (Grade 1), which was not considered related to study drug. No participant had Grade 4 laboratory abnormalities. One participant (6.7%) had Grade 3 increased fasting triglycerides. No participant had notable changes in vital signs (systolic blood pressure, diastolic blood pressure, pulse, and temperature) or clinically significant ECG abnormalities. Specifically, no participant had postbaseline QTcF exceeding 450 msec.

1.2.2.2. GS-US-200-4625

Study GS-US-200-4625 is a Phase 2/3, placebo-controlled, multicenter study of GS-6207 together with an OBR in HTE PWH with multidrug resistant infection.

Herein is a summary from the 2 clinical study reports of interim analysis at Week 26 (after all participants in Cohort 1 completed the Week 26 visit) and the Week 52 addendum (after all participants in Cohort 1 completed the Week 52 visit and all participants in Cohort 2 completed the Week 26 visit).

Participants in Cohort 1 were randomized to receive either oral GS-6207 or placebo, while also continuing their failing regimen during a 14-day Functional Monotherapy Period. After this period, participants who received oral GS-6207 switched to SC GS-6207 (972 mg; 309 mg/mL; 2 × 1.5 mL) and OBR, and participants who had received placebo then received oral GS-6207 and OBR for 14 days prior to switching to SC GS-6207 (972 mg; 309 mg/mL; 2 × 1.5 mL) and OBR. Participants in Cohort 2 received oral GS-6207 and OBR for 14 days before switching to SC GS-6207 and OBR. Cohort 2 includes participants that did not meet the criteria for randomization in Cohort 1 or who joined the study after Cohort 1 was fully enrolled.

Disposition and Baseline Characteristics

Overall, in Cohorts 1 and 2, 72 participants were enrolled in the study and were included in the Safety Analysis Set (Cohort 1: GS-6207, 24 participants; placebo, 12 participants; Cohort 2: GS-6207 + OBR, 36 participants). All 72 participants completed the Functional Monotherapy (Cohort 1, 36 participants) or Oral Lead-in Period (Cohort 2, 36 participants), and all received Day 1 SC GS-6207.

Of the 72 participants who received Day 1 SC GS-6207, 32 participants (all from Cohort 2) are continuing study drug in the Main Phase, and 37 participants completed study drug in the Main Phase (Week 52). Three participants discontinued study drug during the Main Phase. Reasons for premature discontinuation were lost to follow-up, investigator's discretion, and death (1 participant each, 1.4%). One participant who decided not to receive SC GS-6207 at Week 52 and not to continue the study completed the study at the Week 52 visit. Thirty-six participants entered into the Extension Phase (Cohort 1: 34 participants; Cohort 2: 2 participants). Of the 36 participants who entered the Extension Phase, 34 participants are continuing study drug. Two participants discontinued study drug during the Extension Phase. Reasons for premature discontinuation were AE and lost to follow-up (1 participant each, 2.8%).

Cohort 1

In Cohort 1, demographic and baseline characteristics were generally similar between the GS-6207 and placebo groups. The majority of participants were male (72.2%; 26 of 36 participants), White (45.7%, 16 participants) or Black (45.7%, 16 participants), and not Hispanic or Latino (71.4%, 25 participants). Median age was 54 years (range: 24 to 71 years).

Baseline disease characteristics were consistent with the profile of the HTE population, with a median (range) number of prior ARV medications of 9 (2, 24), and 75% of participants with CD4+ cell count < 200 cells/μL (a hallmark of severe immune suppression and the criterion to diagnose AIDS). Differences were seen between the GS-6207 and placebo groups in HIV-1 RNA ($\log_{10}$ copies/mL), HIV-1 RNA categories, and CD4+ cell counts and CD4+ percentage.

The most common prior ARV medications were as follows: INSTI (97.2%), NRTI (94.4%), NNRTI (88.9%), and PI (83.3%). Known resistance to ≥ 2 drugs in class was as follows: NRTI (97.2%), NNRTI (94.4%), PI (77.8%), and INSTI (75.0%).

The median number of ARVs in the failing regimen for Cohort 1 was 3 (range: 1 to 7). The composition of participants' failing regimens were characteristic of those of PWH with MDR, for example, PI (boosted darunavir twice daily), INSTI (dolutegravir twice daily), chemokine receptor 5 entry inhibitor (maraviroc), CD4+-directed post attachment (ibalizumab), attachment inhibitor (fostemsavir, which was investigational at the time of enrollment), and fusion inhibitor (enfuvirtide).

The median number of ARVs in the OBR was 4 (range: 2 to 7). The composition of participants' failing regimens and OBRs were similar, suggesting that they had few remaining treatment options prior to enrolling. Specifically, 6 out of 36 (16.7%) of participants continued their failing regimens as OBRs, suggesting there were no viable agents that could have further optimized the regimen.

The percentage of participants by number of fully active ARV agents in the OBR were as follows: 16.7% (0 fully active ARV agents), 38.9% (1 fully active ARV agent), 25.0% (2 fully active ARV agents), and 19.4% (≥ 3 fully active ARV agents).

Cohort 2

In Cohort 2, the majority of participants were male (77.8%; 28 of 36 participants), White (36.1%, 13 participants) or Asian (33.3%, 12 participants), and not Hispanic or Latino (86.1%, 31 participants). Median age was 49 years (range: 23 to 78 years).

The baseline disease characteristics, prior ARVs, failing regimens, OBR regimen, and resistance characteristics for Cohort 2 were consistent with the profile of the HTE population.

Efficacy Results:

Primary efficacy endpoint: Reduction in HIV 1 RNA of ≥ 0.5 $\log_{10}$ copies/mL

A significantly greater percentage of participants receiving GS-6207 had a reduction in HIV-1 RNA of ≥ 0.5 $\log_{10}$ copies/mL from baseline at the end of the Functional Monotherapy Period compared than those receiving placebo (87.5% vs 16.7%; $P < 0.0001$). To address the imbalance in baseline HIV-1 RNA between the GS-6207 and placebo groups, a post hoc analysis of the primary efficacy endpoint with adjustment for baseline HIV-1 RNA using rank analysis of covariance was conducted. Results from this post hoc analysis confirmed that the difference between the groups remained statistically significant: 87.5% versus 16.7%; $P = 0.0003$. To address the imbalance in baseline CD4+ cell count between the GS-6207 and placebo groups, post hoc analyses of the primary efficacy endpoint were conducted in participants with comparable or clinically relevant CD4+ cell counts. These analyses showed that the difference between groups remained statistically significant for the comparison between participants in the GS-6207 group with a low baseline CD4+ cell count (median: 98.5 cells/μL; n = 12) and participants in the placebo group (median: 84.5 cells/μL; n = 12) ($P = 0.0008$) and between participants in the GS-6207 and placebo groups with a baseline CD4+ cell count < 200 cells/μL ($P < 0.0001$).

Secondary Efficacy Endpoints

Week 26

The percentages of participants in Cohort 1 with HIV-1 RNA < 50 copies/mL and < 200 copies/mL at Week 26 using the United States (US) Food and Drug Administration (FDA)-defined snapshot algorithm were 80.6% (29 of 36 participants) and 88.9% (32 of 36 participants), respectively. The percentages of participants in Cohort 2 with HIV-1 RNA < 50 and < 200 copies/mL at Week 26 using the US FDA-defined snapshot algorithm were 66.7% (4 of 6 participants) and 66.7% (4 of 6 participants), respectively.

Week 52

The percentages of participants in Cohort 1 with HIV 1 RNA < 50 copies/mL and < 200 copies/mL at Week 52 using the US FDA defined snapshot algorithm were 83.3% (30 of 36 participants) and 86.1% (31 of 36 participants), respectively.

HIV-1 RNA < 50 Copies/mL and < 200 Copies/mL

At Week 26, the percentages of participants in Cohorts 1 and 2 with HIV-1 RNA < 50 copies/mL and < 200 copies/mL using the US FDA-defined snapshot algorithm were 80.6% (58 of 72 participants) and 87.5% (63 of 72 participants), respectively.

At Week 52, the percentages of participants in Cohorts 1 and 2 with HIV-1 RNA < 50 copies/mL and < 200 copies/mL using the US FDA-defined snapshot algorithm were 77.8% (35 of 45 participants) and 82.2% (37 of 45 participants), respectively.

Change From Baseline in HIV-1 RNA

Mean (SD) baseline HIV-1 RNA values of participants in Cohorts 1 and 2 was 4.12 (1.028) $\log_{10}$ copies/mL. The mean (SD) change from baseline in HIV-1 RNA at Weeks 26 and 52 was −2.53 (1.184) $\log_{10}$ copies/mL, and −2.50 (1.145) $\log_{10}$ copies/mL, respectively.

Change From Baseline in CD4+ Cell Count

Mean (SD) baseline CD4+ cell count value of participants in Cohorts 1 and 2 at baseline was 212 (226.2) cells/μL. At Week 26, mean (SD) change from baseline was 89 (106.7) cells/μL. At Week 52, mean (SD) change from baseline was 94 (121.5) cells/μL.

Preliminary Pharmacokinetic Results

The key findings of GS-6207 plasma PK parameters and concentration-time profiles based on interim analysis at Week 52 were:

- Mean GS-6207 concentrations and the lower bound 90% CI reached IQ4 (15.5 ng/mL) in approximately 1 hour postdose on Day 1 with majority of the participants (> 80%) achieving concentrations above IQ4 at 4 hours postdose on Day 1 and continued to be above IQ4 through the end of dosing intervals at Week 26 and Week 52.

- For the Oral Lead-in Period, mean [%CV] predose concentrations, and the lower bound 90% CI, on Day 2 (55.0 ng/mL [116%]; lower 90% CI: = 42.3 ng/mL), on Day 8 (61.0 ng/mL [70.9%]; lower 90% CI: = 52.5 ng/mL), and Day 1 SC (50.5 ng/mL [66.1%]; lower 90% CI: = 44.0 ng/mL) exceeded IQ4.

- For the maintenance period, mean [%CV] predose concentrations, and the lower bound 90% CI, at Week 26 (35.9 ng/mL [57.1%]; lower 90% CI: = 31.8 ng/mL) and at Week 52 (40.8 ng/mL [51.0%]; lower 90% CI: = 35.0 ng/mL) exceeded IQ4.

- C_{trough} at Week 52 was 1.14-fold higher compared with Week 26 indicating minimal accumulation over the dosing period of 52 weeks.

Adverse Events

Functional Monotherapy Period

During the Functional Monotherapy Period, the percentages of participants who experienced AEs were: GS-6207 37.5% (9 of 24 participants); placebo 25.0% (3 of 12 participants). Nausea was the only AE reported in > 1 participant (GS-6207 12.5%, 3 participants).

Adverse event considered related to study drugs that was reported in > 1 participant was as follows: nausea (8.3%, 2 participants; GS-6207 group).

No deaths, serious adverse events (SAEs), AEs leading to discontinuation of study drug, or Grade 3 or higher AEs, were reported in either the GS-6207 or placebo group.

All GS-6207 Analysis

The percentage of participants who received GS-6207 in Cohorts 1 and 2 and experienced AEs was 93.1% (67 of 72 participants). The 3 most commonly reported AEs were injection site pain (37.5%, 27 of 72 participants), injection site swelling (33.3%, 24 participants), and injection site erythema (27.8%, 20 participants). Some injection site reactions (ISRs) were attributed to enfuvirtide.

The majority of AEs were Grade 1 or 2 in severity. Grade 3 or higher AEs were reported for 16 participants (22.2%). Grade 3 or higher AEs that were reported for ≥ 2 participants were injection site erythema (5.6%, 4 participants), injection site edema, injection site pain, and injection site swelling (2.8%, 2 participants each). Four participants experienced Grade 3 or higher AEs that were considered related to study drug: rash and abdominal abscess, injection site swelling and injection site erythema, injection site pain, and immune reconstitution inflammatory syndrome (1 participant each). The AE of abdominal abscess was an abscess at the injection site due to secondary infection most likely because the participant scratched the injection site.

Overall, 66.7% (48 of 72 participants) experienced treatment-related AEs. The most commonly reported treatment-related AEs were injection site pain and injection site swelling (30.6%, 22 participants), injection site erythema (25.0%, 18 participants), and injection site nodule (23.6%, 17 participants)

Serious adverse events (SAEs) were reported for 11.1% (8 of 72 participants). The only SAE that was reported for more than 1 participant was COVID-19. None of these events led to discontinuation of study drug. No SAEs were considered related to study drug. One SAE of cancer resulted in death which was also reported as an AE leading to premature discontinuation from the study. This participant in Cohort 2 died on Study Day 90, and the cause of death was reported as cancer. The event was previously reported in the Week 26 analysis and was considered not related to study drug.

While no participant discontinued study drug due to AE at the Week 26 analysis, 1 participant (1.4%) in Cohort 1 experienced a Grade 1 AE of injection site nodule during the Extension Phase, leading to premature discontinuation from the study after receiving the Week 52 GS-6207 SC injection. The event was considered related to study drug.

Overall, 45 participants (62.5%) experienced a study drug-related ISR. All were Grade 1 or 2 with the exception of 2 participants (2.8%) who experienced a Grade 3 ISR which resolved after a few days. No Grade 3 or 4 ISRs were reported beyond Week 26. The median (Q1, Q3) total duration of any study drug-related ISR was 8 (3, 67) days. The most frequently reported study drug-related ISRs (reported in ≥ 10% of participants overall) and duration in median (Q1, Q3) days were as follows:

- Injection site swelling (30.6%, 22 participants), 12 (6, 30) days
- Injection site pain (30.6%, 22 participants), 3 (2, 7) days
- Injection site nodule (23.6%, 17 participants), 180 (111, 330) days
- Injection site erythema (25%, 18 participants), 6 (3, 8) days
- Injection site induration (15.3%, 11 participants), 118 (15, 182) days

A numerically lower percentage of participants had ISRs after the second GS-6207 injection compared with the first injection.

Overall, 17 participants experienced study drug-related injection site nodule, and 11 participants experienced study drug-related injection site induration. All events were Grade 1 or 2.

The injection site nodules and indurations reported due to Day 1 SC injection were as follows:

- Participant level analysis:
  - Nodules: Ongoing (9/16 [56.3%]); Resolved (7/16 [43.8%])
  - Indurations: Ongoing (1/8 [12.5%]); Resolved (7/8 [87.5%])
- Event level analysis:
  - Nodules: Ongoing (9/23 [39.1%]); Resolved (14/23 [60.9%])
  - Indurations: Ongoing (1/8 [12.5%]); Resolved (7/8 [87.5%])

The median (Q1, Q3) duration of resolved injection site nodule and injection site induration due to Day 1 SC injection was 107 (70, 227) days and 43 (15, 196) days, respectively.

The injection site nodules and indurations reported due to the second GS-6207 injection at Week 26 were as follows:

- Participant level analysis:
  - Nodules: Ongoing (6/8 [75.0%%]); Resolved (2/8 [25.0%])
  - Indurations: Ongoing (3/7 [42.9%]); Resolved (4/7 [57.1%])
- Event level analysis:
  - Nodules: Ongoing (12/14 [85.7%]); Resolved (2/14 [14.3%])
  - Indurations: Ongoing (3/7 [42.9%]); Resolved (4/7 [57.1%])

The median (Q1, Q3) duration of resolved injection site nodule and injection site induration due to the second GS-6207 injection at Week 26 was 92 (3, 180) Days and 38 (9, 123) Days, respectively.

CONCLUSIONS:

The conclusions from the Week 52 interim analysis of Study GS-US-200-4625 are as follows:

- Consistent with the data reported in the Study GS-US-200-4625 Interim Week 26 CSR, high rates of virologic suppression continued to be maintained through Week 52. These results were consistent even in participants who had suboptimal baseline OBR (eg, low OSS, no or 1 fully active agent, INSTI resistance, no dolutegravir or darunavir), demonstrating a clinically meaningful contribution of GS-6207 towards virologic suppression.
- Consistent with the data reported in the Study GS-US-200-4625 Interim Week 26 CSR, there were clinically meaningful increases in CD4+ cell count from baseline to Week 52.

GS-6207 remained generally safe and well tolerated. No participant experienced a study drug-related SAE. The majority of ISRs were Grade 1 or 2 in severity. One participant discontinued GS-6207 at Week 52 (after the Week 26 interim analysis) due to an AE of Grade 1 injection site nodule.

1.2.2.3. GS-US-200-4334

Study GS-US-200-4334 is an ongoing, Phase 2, randomized, open-label, active-controlled, multicenter study evaluating the safety and efficacy of GS-6207 in combination with other ARV agents in ARV-naive PWH. Participants were randomized in a 2:2:2:1 ratio to 1 of 4 treatment groups (Table 1-4, Figure 1-3).

Participants are treated for at least 80 weeks. Participants in Treatment Group 4 will complete the study at Week 80.

Herein is a summary from the 2 clinical study reports of interim analysis at Week 28 and the Week 54 addendum.

Table 1-4 GS-US-200-4334: Study Treatments

Treatment Group	Time Period	Study Treatments
1	Induction (Day 1 through Week 27)	• Oral GS-6207 600 mg (2 × 300 mg tablet) on Days 1 and 2; 300 mg (1 × 300 mg tablet) on Day 8<br>• Oral daily F/TAF (200/25 mg) from Day 1 onwards for a total of 28 weeks[a]<br>• SC GS-6207 927 mg (3 mL of 309 mg/mL) on Day 15
	Maintenance (Week 28 through Week 80)	• SC GS-6207 927 mg (3 mL of 309 mg/mL) at Week 28 and once every 26 weeks ± 2 weeks from last injection thereafter<br>• Oral daily TAF (25 mg)
2	Induction (Day 1 through Week 27)	• Oral GS-6207 600 mg (2 × 300 mg tablet) on Days 1 and 2; 300 mg (1 × 300 mg tablet) on Day 8<br>• Oral daily F/TAF (200/25 mg) from Day 1 onwards for a total of 28 weeks[b]<br>• SC GS-6207 927 mg (3 mL of 309 mg/mL) on Day 15
	Maintenance (Week 28 through Week 80)	• SC GS-6207 927 mg (3 mL of 309 mg/mL) at Week 28 and every 26 weeks thereafter<br>• Oral daily BIC (75 mg)
3	Day 1 through Week 80	• Oral GS-6207 600 mg (2 × 300 mg tablet) on Days 1 and 2; 50 mg (1 × 50 mg tablet) daily from Day 3 onwards<br>• Oral daily F/TAF (200/25 mg)
4	Day 1 through Week 80	• Oral daily B/F/TAF (50/200/25 mg)

BIC, B = bictegravir; F = emtricitabine; GS-6207 = lenacapavir; SC = subcutaneous; TAF = tenofovir alafenamide

a Treatment Group 1 participants with < 50 copies/mL of HIV-1 RNA at Week 16 and Week 22 stopped F/TAF at Week 28 and initiated oral daily TAF.

b Treatment Group 2 participants with < 50 copies/mL of HIV-1 RNA at Week 16 and Week 22 stopped F/TAF at Week 28 and initiated oral daily BIC.

Figure 1-3 GS-US-200-4334: Study Design

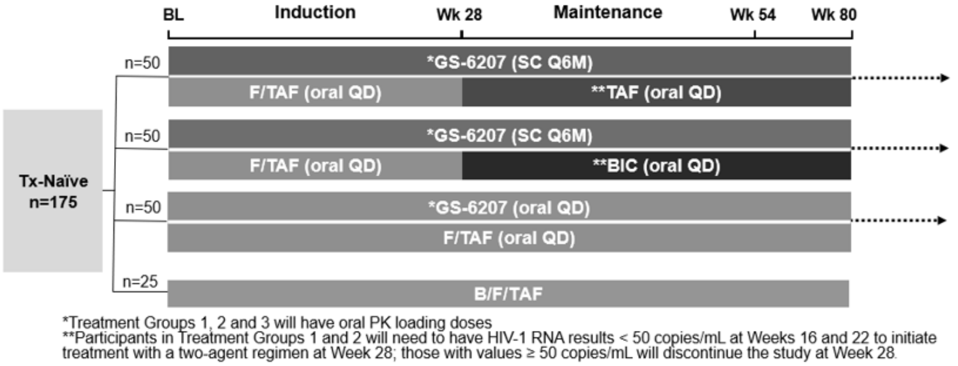

BIC, B = bictegravir; B/F/TAF = bictegravir/emtricitabine/tenofovir alafenamide (coformulated; Biktarvy®, BVY); BL = baseline; F/TAF = emtricitabine/tenofovir alafenamide (coformulated; Descovy®); GS-6207 = lenacapavir, LEN; PK = pharmacokinetic; QD = once daily; Q6M = every 6 months; SC = subcutaneous; TAF = tenofovir alafenamide; Tx-naive = treatment naive; Wk = week

Disposition and Baseline Characteristics

Of the 249 participants screened, 183 were randomized, and 182 received at least 1 dose of study drug. Of these 182 participants, 22 participants (12.1%) prematurely discontinued the study drug during the Main Phase (ie, data collected on or before Week 80): 17 of 105 participants (16.2%) in the SC GS-6207 total group, 4 of 52 participants (7.7%) in the oral GS-6207 group, and 1 of 25 participants (4.0%) in the BVY group. The reasons for premature study drug discontinuation in the SC GS-6207 total group were participant decision and lost to follow-up (each 4 participants [3.8%]), investigator's discretion, lack of efficacy, and AE (each 3 participants [2.9%]). The reasons for premature study drug discontinuation in the oral GS-6207 group were lost to follow-up (3 participants [5.8%]) and participant decision (1 participant [1.9%]) and in the BVY group it was participant decision (1 participant [4.0%]).

As of the Week 54 data cut date, 8 participants entered into the Extension Phase (SC GS-6207 total group, 5 participants; oral GS-6207 group, 3 participants) and were continuing study drug.

Demographics, general baseline characteristics, and baseline disease characteristics are described in the Interim Week 28 CSR.

Demographic and baseline characteristics were similar across the treatment groups. Most participants were male (93.4%), cisgender (92.3%), and gay (69.8%). Median age was 29 years (range: 19 to 72 years). Race was balanced between Black versus non-Black (52.2% vs 47.8%, respectively), as was ethnicity between Hispanic or Latino versus not Hispanic or Latino (45.1% vs 54.9%, respectively).

Baseline disease characteristics were similar across the treatment groups. Overall, the median (Q1, Q3) baseline HIV-1 RNA value was 4.37 (3.86, 4.74) $\log_{10}$ copies/mL, and the median (Q1, Q3) baseline CD4+ cell count was 437 (332, 599) cells/μL. Among the 182 participants, most had HIV-1 RNA ≤ 100,000 copies/mL (85.2%, 155 participants) and a CD4+ cell count range of ≥ 350 to < 500 cells/μL (31.9%, 58 participants) or ≥ 500 cells/μL (37.4%, 68 participants).

Efficacy Results:

Primary Efficacy Endpoint

HIV-1 RNA < 50 copies/mL at Week 54 Using the US FDA-Defined Snapshot Algorithm

The primary analysis at Week 54 demonstrated similar results in each treatment group, with no significant difference between each of the GS-6207-containing groups and the BVY group, as follows:

- GS-6207 total: 136 of 157 participants (86.6%)
- SC GS-6207 + (DVY → TAF): 47 of 52 participants (90.4%)
- SC GS-6207 + (DVY → BIC): 45 of 53 participants (84.9%)
- Oral GS-6207 + DVY: 44 of 52 participants (84.6%)
- BVY: 23 of 25 participants (92.0%)

For participants with HIV-1 RNA < 50 copies/mL at Week 28, the proportion with HIV-1 RNA < 50 copies/mL at Week 54 using the US FDA-defined snapshot algorithm were also similar in each treatment group, with no significant difference between each of the GS-6207-containing groups and the BVY group, as follows:

- GS-6207 total: 135 of 147 participants (91.8%)
- SC GS-6207 + (DVY → TAF): 46 of 49 participants (93.9%)
- SC GS-6207 + (DVY → BIC): 45 of 49 participants (91.8%)
- Oral GS-6207 + DVY: 44 of 49 participants (89.8%)
- BVY: 23 of 25 participants (92.0%)

Secondary Efficacy Endpoints

Week 28

Results from the secondary efficacy analysis of Week 28 data of Study GS-US-200-4334 demonstrated similar results in each treatment group, with no significant difference between each of the GS-6207-containing treatment groups and the BVY treatment group with respect to the proportion of participants with HIV-1 RNA < 50 copies/mL using the US FDA-defined snapshot algorithm, the change from baseline in $\log_{10}$ HIV-1 RNA, and the change from baseline in CD4+ cell count.

HIV-1 RNA < 50 copies/mL using the US FDA-defined snapshot algorithm

- GS-6207 total: 147 of 157 participants (93.6%)
- SC GS-6207 + (DVY → TAF): 49 of 52 participants (94.2%)
- SC GS-6207 + (DVY → BIC): 49 of 53 participants (92.5%)
- Oral GS-6207 + DVY: 49 of 52 participants (94.2%)
- BVY: 25 of 25 participants (100.0%)

Week 38

HIV-1 RNA < 50 copies/mL at Week 38 Using the US FDA-Defined Snapshot Algorithm

- GS-6207 total: 140 of 157 participants (89.2%)
- SC GS-6207 + (DVY → TAF): 47 of 52 participants (90.4%)
- SC GS-6207 + (DVY → BIC): 47 of 53 participants (88.7%)
- Oral GS-6207 + DVY: 46 of 52 participants (88.5%)
- BVY: 24 of 25 participants (96.0%)

Change from Baseline in HIV-1 RNA at Weeks 28, 38, and 54

Week 28

- GS-6207 total: −2.99 (0.666) $\log_{10}$ copies/mL
- SC GS-6207 + (DVY → TAF): −2.92 (0.649) $\log_{10}$ copies/mL
- SC GS-6207 + (DVY → BIC): −3.04 (0.638) $\log_{10}$ copies/mL
- Oral GS-6207 + DVY: −3.01 (0.716) $\log_{10}$ copies/mL

Week 38

- GS-6207 total: −3.01 (0.681) $\log_{10}$ copies/mL
- SC GS-6207 + (DVY → TAF): −2.96 (0.639) $\log_{10}$ copies/mL
- SC GS-6207 + (DVY → BIC): −3.06 (0.641) $\log_{10}$ copies/mL
- Oral GS-6207 + DVY: −3.02 (0.771) $\log_{10}$ copies/mL
- BVY: −3.04 (0.760) $\log_{10}$ copies/mL

Week 54

- GS-6207 total: −2.98 (0.700) $\log_{10}$ copies/mL
- SC GS-6207 + (DVY → TAF): −2.95 (0.636) $\log_{10}$ copies/mL
- SC GS-6207 + (DVY → BIC): −3.12 (0.589) $\log_{10}$ copies/mL
- Oral GS-6207 + DVY: −2.85 (0.840) $\log_{10}$ copies/mL
- BVY: −3.08 (0.787) $\log_{10}$ copies/mL

Change from Baseline in CD4+ Cell Count at Weeks 28, 38, and 54

Week 28

- GS-6207 total: 179 (166.0) cells/μL
- SC GS-6207 + (DVY → TAF): 172 (178.2) cells/μL
- SC GS-6207 + (DVY → BIC): 158 (164.1) cells/μL
- Oral GS-6207 + DVY: 206 (154.6) cells/μL
- BVY: 163 (157.7) cells/μL

Week 38

- GS-6207 total: 208 (172.7) cells/μL
- SC GS-6207 + (DVY → TAF): 195 (164.6) cells/μL
- SC GS-6207 + (DVY → BIC): 219 (187.4) cells/μL
- Oral GS-6207 + DVY: 210 (167.1) cells/μL
- BVY: 232 (209.3) cells/μL

Week 54

- GS-6207 total: 213 (182.1) cells/μL
- SC GS-6207 + (DVY → TAF): 206 (187.0) cells/μL
- SC GS-6207 + (DVY → BIC): 212 (187.1) cells/μL
- Oral GS-6207 + DVY: 220 (175.5) cells/μL
- BVY: 193 (191.1) cells/μL

Results from the secondary efficacy analysis at Weeks 38 and 54 demonstrated similar results in each treatment group, with no significant difference between each of the GS-6207-containing treatment groups and the BVY treatment group with respect to the proportion of participants with HIV 1 RNA < 50 copies/mL (Week 38), the change from baseline in $\log_{10}$ HIV 1 RNA, and the change from baseline in CD4+ cell count (Weeks 38 and 54).

Preliminary Pharmacokinetic Results

Based on the interim analysis, for both the SC LEN treatment groups (SC LEN + [DVY → TAF] and SC LEN + [DVY → BIC]), mean LEN concentrations and the lower bound 90% CI were consistently maintained above IQ4 (15.5 ng/mL) from Day 2 onwards to Week 54. For SC LEN, mean predose concentrations at Week 54 for Treatment Groups 1 and 2 were 1.22-fold and 1.15-fold higher, respectively compared to Week 28, indicating minimal accumulation over the dosing period of 54 weeks.

For oral LEN 50 mg once daily (oral LEN + DVY treatment group), mean LEN single anytime concentrations and the lower bound 90% CI reached IQ4 (15.5 ng/mL) by Day 2 and were maintained at Weeks 28 and 54 for this interim analysis. For oral LEN, accumulation was not apparent after Week 4 indicating steady-state is achieved by Week 4. Mean predose concentration at Week 54 was consistent with that at Week 28.

Safety Results:

Lenacapavir and BVY were well tolerated. By Week 54, 89.2% of participants in the GS-6207 total group (140 of 157 participants) and 96.0% of those in the BVY group (24 of 25 participants) had been exposed to study drug for at least 54 weeks

Adverse Events

Similar percentages of participants in the GS-6207 total (total of 157 participants) and BVY (total of 25 participants) groups had any AE by Week 54 (GS-6207 total 87.9%, 138 participants; BVY 84.0%, 21 participants).

The most commonly reported AEs were as follows:

- GS-6207 total (157 participants), excluding ISRs: headache and nausea (each 13.4%, 21 participants), COVID-19 (9.6%, 15 participants), and syphilis and lymphadenopathy (each 8.9%, 14 participants)
- SC GS-6207 total (105 participants), only ISRs: injection site erythema (31.4%, 33 participants), injection site swelling (27.6%, 29 participants), and injection site pain (23.8%, 25 participants)
- Oral GS-6207 + DVY (52 participants): headache (13.5%, 7 participants), nausea and lymphadenopathy (each 11.5%, 6 participants) and COVID-19, syphilis, diarrhea, and influenza (each 9.6%, 5 participants)
- BVY (25 participants): syphilis and arthralgia (each 16.0%, 4 participants) and headache, COVID-19, back pain, weight increased, upper respiratory tract infection, and insomnia (each 12.0%, 3 participants)

The majority of the AEs reported in the 4 treatment groups were Grade 1 or 2 in severity. The percentage of participants with AEs of Grade 3 or higher was 8.3% (13 of 157 participants) in the GS-6207 total group and 8.0% (2 of 25 participants) in the BVY group. No AEs of Grade 3 or higher were reported for > 1 participant in any treatment group.

Since the Week 28 analysis, 2 additional AEs of Grade 3 or higher were reported in the GS-6207 total group (SC GS-6207 + [DVY → TAF] group: 1 participant; SC GS-6207 + [DVY → BIC] group: 1 participant]) and 1 additional AE of Grade 3 or higher was reported in the BVY group (1 participant).

The percentages of participants with treatment-related AEs were as follows:

- GS-6207 total (157 participants): 43.9% (69 participants)
- SC GS-6207 total (105 participants): 58.1% (61 participants)
- Oral GS-6207 + DVY (52 participants): 15.4% (8 participants)
- BVY (25 participants): 16.0% (4 participants)

The higher frequency of treatment-related AEs in the SC GS-6207 total group was mainly because of ISRs, as reflected in the most common AEs considered related to study drugs:

- GS-6207 total group (157 participants), excluding ISRs: nausea (5.1%, 8 participants), diarrhea and headache (each 2.5%, 4 participants), and fatigue (1.9%, 3 participants)
- SC GS-6207 total group (105 participants), only ISRs: injection site erythema (26.7%, 28 participants), injection site swelling (22.9%, 24 participants), injection site pain (19.0%, 20 participants)
- Oral GS-6207 + DVY (52 participants): nausea and diarrhea (each 3.8%, 2 participants), fatigue, headache, dyspepsia, flatulence, vomiting, weight increased, influenza, overdose, and hot flush (each 1.9%, 1 participant)
- BVY (25 participants): dyspepsia, salivary hypersecretion, insomnia, and weight increased (4.0%, 1 participant)

Overall, 57 of 103 participants (55.3%) who received SC GS-6207 had a study drug-related ISR; all were Grade 1 or Grade 2, except for 1 participant with a Grade 3 injection site nodule. There were no new Grade 3 or higher ISRs reported since the Week 28 analysis. The most frequently reported ISRs (≥ 10% in SC GS-6207 total group) and their duration in median (Q1, Q3) days were as follows:

- Injection site erythema (27.2%, 28 participants), 5 (2, 11) days
- Injection site swelling (23.3%, 24 participants), 11 (6, 29) days

- Injection site pain (19.4%, 20 participants), 4 (1, 9) days
- Injection site nodule (14.6%, 15 participants), 195 (122, 301) days
- Injection site inflammation (13.6%, 14 participants), 3 (1, 8) days
- Injection site induration (12.6%, 13 participants), 202 (101, 361) days

Overall, 15 of 103 participants had an AE of injection site nodule and 13 of 103 participants had an AE of injection site induration. All were Grade 1 or Grade 2, except for 1 participant with a Grade 3 injection site nodule. Ongoing and resolved injection site indurations and nodules were as follows:

ISRs after the first SC GS-6207 injection at Day 15:

Participant-level analysis:

- Nodules: Ongoing (1 of 11 [9.1%]); Resolved (10 of 11 [90.9%])
- Indurations: Ongoing (6 of 9 [66.7%]); Resolved (3 of 9 [33.3%])

Event-level analysis:

- Nodules: Ongoing (1 of 13 [7.7%]); Resolved (12 of 13 [92.3%])
- Indurations: Ongoing (6 of 9 [66.7%]); Resolved (3 of 9 [33.3%])

The median (Q1, Q3) duration of resolved injection site nodule and injection site induration was 278 (136, 366) days and 202 (101, 213) days, respectively.

ISRs after the second SC GS-6207 injection at Week 28:

Participant-level analysis:

- Nodules: Ongoing (5 of 8 [62.5%]); Resolved (3 of 8 [37.5%])
- Indurations: Ongoing (3 of 6 [50.0%]); Resolved (3 of 6 [50.0%])

Event-level analysis:

- Nodules: Ongoing (5 of 8 [62.5%]); Resolved (3 of 8 [37.5%])
- Indurations: Ongoing (3 of 6 [50.0%]); Resolved (3 of 6 [50.0%])

The median (Q1, Q3) duration of resolved injection site nodule and injection site induration was 152 (123, 240) days and 145 (4, 258) days, respectively.

CONCLUSIONS:

The conclusions from the Week 54 interim analysis of Study GS-US-200-4334 are as follows:

- High rates of virologic suppression continued to be maintained through Week 54. For the primary efficacy endpoint, overall, 136 of 157 participants (86.6%) who received SC or oral GS-6207 and 23 of 25 participants (92.0%) who received BVY had HIV-1 RNA < 50 copies/mL at Week 54.
- For participants with HIV-1 RNA < 50 copies/mL at Week 28, high rates of virologic suppression continued to be maintained through Week 54.
- Subcutaneously and orally administered GS-6207 remained generally safe and well tolerated.

1.3. Rationale for This Study

Advances in ART have led to significant improvements in morbidity and mortality among people living with HIV by suppressing viral replication, preserving immunologic function, and averting disease progression to AIDS. While combination ARV therapy for the treatment of HIV-1 infection has been largely successful in reducing the morbidity and mortality associated with HIV disease, there remains a significant medical need for new well-tolerated therapies that take into consideration HIV genetic variability, ARV resistance, and new options for regimen simplification.

Some treatment-experienced (TE) PLWH eventually lose virologic, immunologic, or clinical benefit from their current regimens. People living with HIV with multiple prior regimen failures and significant drug resistance have limited treatment options and may be unable to achieve durable HIV viral suppression {Department of Health and Human Services (DHHS) 2013, Lundgren 2013, Thompson 2010, U. S. Department of Health and Human Services 2015, Williams 2014}. These challenges are also relevant for adolescents particularly those with perinatal HIV-1 transmission (https://www.ncbi.nlm.nih.gov/pmc/articles/PMC4901868/)

Developing safe and effective therapies for heavily TE PLWH with multidrug resistant (MDR) HIV remains a priority. For these individuals, newer treatments are needed to control viral replication, preserve immune function, and prevent clinical progression. In all PLWH, the ideal goal of therapy remains complete and durable viral suppression.

The current amendment has been enacted to comply with regulatory requirements in Japan, which stipulated that an ongoing clinical trial must be converted to a postmarketing clinical trial following approval of the product in Japan.

1.4. Rationale for Dose Selection of GS-6207

The dose selection for GS-6207 in this study is supported by antiviral activity, PK and safety data from the ongoing Phase 1b POC (Study GS-US-200-4072) in TN and TE but capsid inhibitor (CAI)-naïve PLWH, as well as PK and safety data from the two Phase 1 studies in healthy volunteers (Study GS-US-200-4538 and Study GS-US-200-4071).

In the ongoing Phase 1b POC study (GS-US-200-4072), potent antiviral activity of GS-6207 has been demonstrated; the mean maximum HIV-1 RNA decline over 10-day monotherapy after single SC doses of 50 to 450 mg was 1.8 to 2.2 $\log_{10}$ copies/mL. All participants achieved at least 1 $\log_{10}$ copies/mL decline in their HIV-1 RNA at Day 10. Day 10 antiviral activity was comparable across a dose range of single doses of 50 to 450 mg. At these doses, mean (% CV) GS-6207 concentrations on Day 10 were 1.1- to 9.9-fold higher (eg, IQ 1.1 – 9.9) than the protein adjusted (pa)EC_{95} for wild-type HIV-1 ($paEC_{95}$ = 3.87 ng/mL in MT-4 cells) (see the IB).

Phenotypic analyses of GS-6207 susceptibility in patient derived isolates indicate that isolates from TN and heavily TE PLWH have a similar in vitro profile, suggesting similar GS-6207 antiviral activity should be observed in the heavily TE patient population compared to the TN patient population.

Based on these data, a concentration of 15.5 ng/mL (corresponding to an IQ of 4 based on paEC95 from MT-4 cells), is anticipated to provide antiviral activity in the HTE population.

GS-6207 formulations and doses to be evaluated in this study are informed by PK and safety data from Phase 1 Studies GS-US-200-4071 and GS-US-200-4538 in healthy volunteers. The proposed regimen targets an exposure whereby the lower bound of the 90% confidence interval (90% CI) of the C_{trough} is 4-fold higher than the $paEC_{95}$ (ie, IQ4) within a few days of dosing initiation, at Day 14 (end of Functional Monotherapy Period), and at end of the dosing interval (once every 26 weeks).

As described in the IB, the GS-6207 SC solution formulation exhibits a slow initial release necessitating an oral PK load regimen prior to the first SC injection. In Study GS-US-200-4625, to achieve IQ4 within a few days of dosing and to maintain target concentrations through the Functional Monotherapy Period, participants will receive oral tablet doses of GS-6207 600 mg on Days 1 and 2 and an oral tablet dose of GS-6207 300 mg on Day 8.

Upon completion of the Functional Monotherapy Period, participants will receive GS-6207 927 mg SC (309 mg/mL), followed by SC doses of GS-6207 927 mg administered once every 6 months (26 weeks) ± 2 weeks from last injection, along with the optimized background regimen (OBR). This regimen is projected to achieve target exposures of GS-6207 within a few days of initiation and to maintain them through the 6 month (26 weeks) ± 2 weeks from last injection dosing interval.

Safety data from Studies GS-US-200-4071 and GS-US-200-4538 demonstrated favorable safety and tolerability profile of GS-6207 administered as single oral doses of up to 1800 mg, multiple oral daily doses of up to 100 mg or single SC doses of up to 927 mg. GS-6207 exposures in this study are predicted to be within the range of those shown to be safe and well tolerated; thereby, supporting further evaluation of this regimen in this study.

1.5. Rationale for Oral Weekly Bridging of GS-6207 for Missed SC Injection

Participants receiving SC GS-6207 may miss their SC injection window (within 26 to 28 weeks of the previous SC GS-6207 dose [Section 5.3]). If a missed dose is anticipated, participants may receive oral bridging until they can receive their next SC injection.

Oral bridging of GS-6207 is supported by antiviral activity, PK, and safety data from a Phase 1b proof-of-concept study (GS-US-200-4072) and 2 ongoing Phase 2 and 2/3 studies (GS-US-200-4334 and GS-US-200-4625), as well as PK and safety data from 2 Phase 1 studies in healthy volunteers (GS-US-200-4071 and GS-US-200-4333). Phenotypic analyses and PK/pharmacodynamic modeling indicate that a GS-6207 plasma concentration of 15.5 ng/mL, corresponding to IQ of 4 or higher, would provide near maximal antiviral activity (GS-US-200-4072).

The oral bridging dose of GS-6207 is 300 mg administered once weekly starting 26 to 28 weeks after the last GS-6207 SC injection. This oral weekly bridging dose, even when started as late as 28 weeks after the last GS-6207 SC injection, is predicted to immediately maintain the lower bound of the 90% CI of arithmetic mean for GS-6207 C_{tau} above IQ4 (ie, even before reaching steady state) (Figure 1-4A). As long as the oral weekly bridging is initiated between 26 to 28 weeks after the last GS-6207 SC injection, the PK profile upon resuming SC injection is predicted to be comparable with that of the prior SC dose and within the target range regardless of when SC injection is resumed (Figure 1-4B).

GS-6207 has been administered orally at doses up to 1800 mg (Study GS-US-200-4071). Safety data from all completed and ongoing clinical studies indicate that GS-6207 is generally safe and well tolerated at the intended exposures.

Figure 1-4. Simulated Pharmacokinetic Profile of Oral Weekly Bridging of GS-6207 (300 mg) (A) Prior to and (B) After Resuming SC Injection

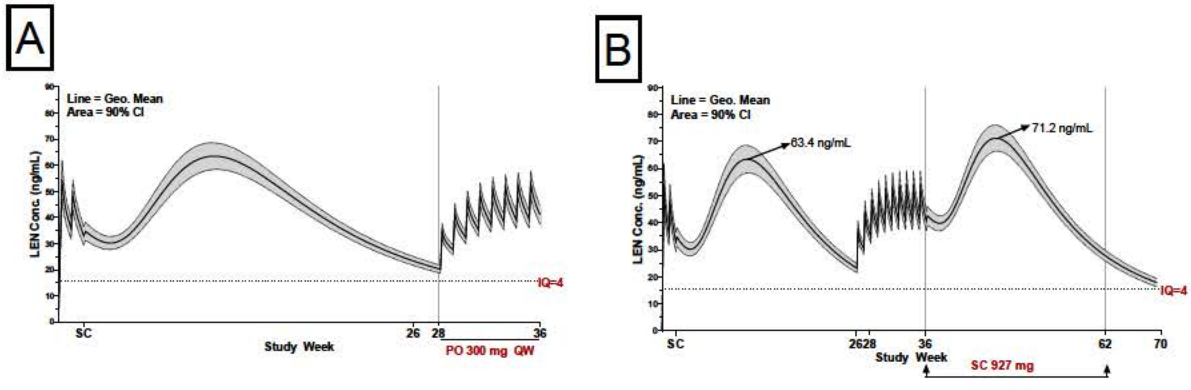

CI = confidence interval; IQ = inhibitory quotient; LEN = lenacapavir (GS-6207); PD = pharmacodynamic; PK = pharmacokinetic; PO = oral; SC = subcutaneous; QW = once weekly
The solid line and the shaded region correspond to the geometric mean and 90% CI, respectively. IQ is calculated as trough concentration/in vitro protein-adjusted EC95 (paEC95) against wild-type virus. Horizontal dashed lines correspond to target IQ values of 4 based on phenotypic analyses and PK/PD modeling.

1.6. Risk/Benefit Assessment for the Study

Potential risks associated with the study include unknown AEs, including ISRs, general risks associated with frequent clinic visits and laboratory blood draws, and the associated pain and discomfort of multiple phlebotomies. Although not specifically evaluated yet, adolescents living with HIV are expected to have similar safety profile as adult PLWH; no additional safety monitoring is required for adolescent participants and no dose adjustments are required. Strategies to mitigate any potential risks include close monitoring of laboratory values as well as AEs. Parameters for monitoring of AEs will be well defined and closely followed.

In addition, potential risks to PLWH include prolonged exposure to subtherapeutic concentrations of GS-6207 if dosing is stopped which could lead to HIV-1 developing resistance to GS-6207. Strategies to mitigate any potential risks include initiation of an OBR within 15 days of starting GS-6207 (Cohort 1) or co-initiation of GS-6207 and an OBR (Cohort 2).

PLWH are eligible for participation in this study if they are highly treatment experienced with limited therapeutic options from the 4 main classes of ARV agents and inability to construct a fully active ARV regimen. GS-6207 offers the potential benefit to construct a highly active regimen able to suppress HIV-1 replication, restoring or preserving immunologic function, and averting disease progression to AIDS. Given the above, the benefit-risk balance for this study is considered positive.

During the period of a pandemic (such as COVID-19), additional potential risks to participants may include interruptions to study visit schedule, inadequate study drug availability, and unrecognized safety issues due to interruptions in adherence to protocol-specified safety monitoring or laboratory assessments. Refer to Appendix 5 for further details on the risks and risk mitigation strategy.

1.7. Compliance

This study will be conducted in compliance with this protocol, Good Clinical Practice (GCP), Good Postmarketing Study Practice (GPSP), and all applicable regulatory requirements.

2. OBJECTIVES

The primary objective of this study is:

- To evaluate the antiviral activity of GS-6207 (Lenacapavir, LEN) administered as an add-on to a failing regimen (functional monotherapy) for PLWH with MDR as determined by the proportion of participants achieving at least 0.5 $\log_{10}$ reduction from baseline in HIV-1 RNA at the end of the Functional Monotherapy Period

The secondary objectives of this study are:

- To evaluate the safety and efficacy of GS-6207 in combination with an OBR at Weeks 26 and 52
- To evaluate the safety and efficacy of GS-6207 in combination with an OBR at Weeks 104 and 156 from the first SC dose of GS-6207

CCI [redacted]

[redacted]

[redacted]

[redacted]

[redacted]

3. STUDY DESIGN

3.1. Endpoints

The primary endpoint of this study is:

- The proportion of participants in Cohort 1 achieving ≥ 0.5 $\log_{10}$ copies/ml reduction from baseline in HIV-1 RNA at the end of Functional Monotherapy Period

The secondary endpoints of this study are:

- The proportion of participants in Cohort 1 with plasma HIV-1 RNA < 50 copies/mL and < 200 copies/mL at Weeks 26 and 52 visits based on the US FDA-defined snapshot algorithm
- The proportion of participants in combined Cohorts 1 and 2 with plasma HIV-1 RNA < 50 copies/mL and < 200 copies/mL at Weeks 104 and 156 from the first SC dose of GS-6207 based on the US FDA-defined snapshot algorithm

3.2. Study Design

This is a randomized and placebo-controlled multicenter study of GS-6207 in PLWH. Eligible participants will be enrolled in either of two cohorts.

Participants who complete a Screening visit will return to the clinic between 14 and 30 days after the Screening visit, for a Cohort Selection visit. HIV-1 RNA results from this Cohort Selection visit will be used to randomize the participant in Cohort 1 or enroll them in Cohort 2. Once enrollment in Cohort 1 is complete, the Cohort Selection visit will not be required.

<u>Cohort 1 (n=36)</u>

Functional Monotherapy Period

Eligible participants with both < 0.5 $\log_{10}$ HIV-1 RNA decline compared to the Screening visit and HIV-1 RNA ≥ 400 copies/mL at the Cohort Selection visit will be randomized, in a blinded fashion, in a 2:1 ratio to either receive oral GS-6207 or placebo to match GS-6207 for 14 days. The Sponsor, participants and site staff will be blinded to the treatment assignment. Functional monotherapy will be assessed while participants continue their failing regimen.

After each participant completes the Functional Monotherapy Period, their treatment assignment will be unblinded.

Maintenance Period

Participants who were randomized to receive oral GS-6207 will receive SC GS-6207 and initiate their OBR on Day 1 SC (14 days after the first dose of oral GS-6207) **(Cohort 1A)**.

Participants who were randomized to receive placebo to match GS-6207 will receive oral GS-6207 and initiate their OBR on Day 15 **(Cohort 1B)**. They will receive SC GS-6207 at Day 1 SC (14 days after the first dose of oral GS-6207) while continuing their OBR.

After the Day 1 SC visit, all Cohort 1 participants will continue with study visits at Weeks 4, 10, 16, 22, 26, 36, and 52 (study visits Week X are identified by the number of weeks that have elapsed since the Day 1 SC visit when GS-6207 is first administered by injection and excludes Oral Bridging Periods).

<u>Cohort 2 (n=64)</u>

Oral Lead-in Period

Participants will be enrolled into Cohort 2 if Cohort 1 is fully enrolled or if they do not meet the criteria for randomization in Cohort 1 (eg, they had ≥ 0.5 $\log_{10}$ HIV-1 RNA decline compared to the Screening visit and/or HIV-1 RNA < 400 copies/mL at the Cohort Selection visit). Participants will be enrolled in Cohort 2 to receive oral GS-6207 for 14 days. Participants will initiate an OBR on Day 1.

Maintenance Period

At Day 1 SC (14 days after the first dose of oral GS-6207), participants will receive SC GS-6207 and will continue their OBR. After the Day 1 SC visit, participants will continue with study visits at Weeks 4, 10, 16, 22, 26, 36, and 52 (study visits Week X are identified by the number of weeks that have elapsed since the Day 1 SC visit when GS-6207 is first administered by injection and excludes Oral Bridging Periods).

Study Design Schema

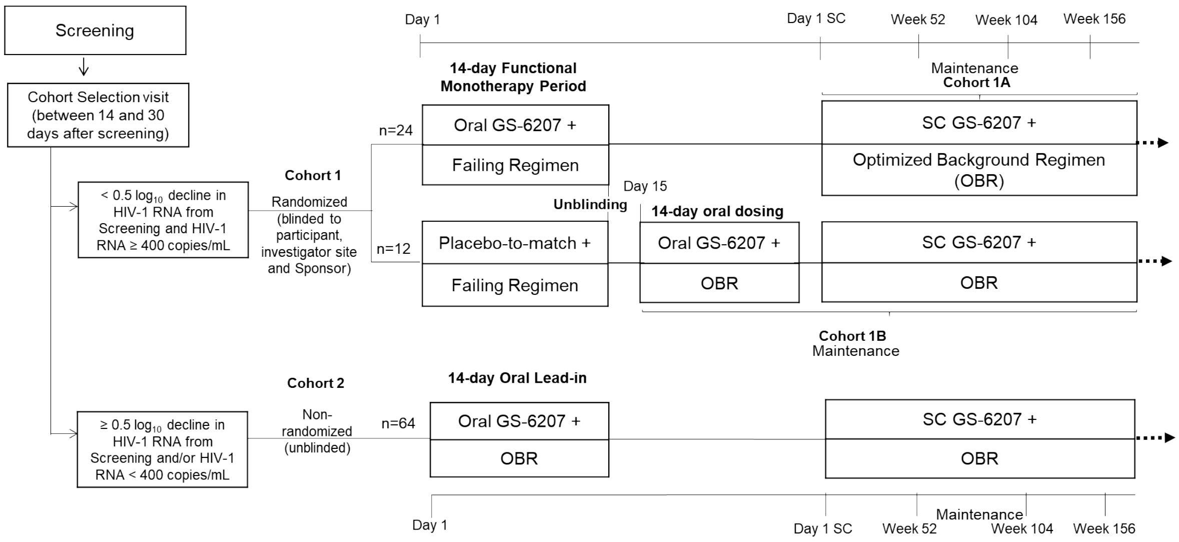

3.3. Study Treatments

Approximately 100 PLWH who meet all eligibility criteria may be enrolled in this study to receive GS-6207.

Cohort 1:

Functional Monotherapy Period: Participant will receive oral GS-6207 600 mg, 600 mg, and 300 mg or placebo to match GS-6207 on Days 1, 2, and 8 respectively while continuing their failing regimen; GS-6207 oral tablets can be administered without regards to food.

After each participant completes the Functional Monotherapy Period, their treatment assignment will be unblinded.

Maintenance Period: At Day 1 SC (14 days after the first dose of oral GS-6207), participants who were randomized to receive oral GS-6207 will receive SC GS-6207 927 mg and initiate an optimized background regimen (as prescribed by the investigator) (**Cohort 1A**). Participants will continue to receive SC GS-6207 927 mg once every 6 months (26 weeks) ± 2 weeks from last injection.

At Day 15, participants who were randomized to receive placebo to match GS-6207 will receive oral GS-6207 600 mg and initiate an optimized background regimen (as prescribed by the investigator). Participants will receive oral GS-6207 600 mg and 300 mg at Day 16 and Day 22, respectively. At Day 1 SC (14 days after the first dose of oral GS-6207), participants will receive SC GS-6207 927 mg while continuing their optimized background regimen (**Cohort 1B**). Participants will continue to receive SC GS-6207 927 mg once every 6 months (26 weeks) ± 2 weeks from last injection.

Cohort 2:

Oral Lead-in Period:

At Day 1, participants will receive oral GS-6207 600 mg and initiate an optimized background regimen (as prescribed by the investigator). Participants will receive oral GS-6207 600 mg and 300 mg at Day 2 and Day 8, respectively, while continuing their optimized background regimen; GS-6207 oral tablets can be administered without regards to food.

Maintenance Period:

At Day 1 SC, participants will receive SC GS-6207 927 mg while continuing their optimized background regimen. Participants will continue to receive SC GS-6207 927 mg once every 6 months (26 weeks) ± 2 weeks from last injection.

<u>**Cohort 1 and Cohort 2**</u>

Participants may require oral bridging if an SC injection of GS-6207 cannot be administered within the protocol visit window. In case of Oral Bridging initiation, participants will receive oral GS-6207 as specified in Section 5.4 and complete Oral Bridging visits. An Oral Bridging visit should be completed every 10 to 12 weeks. Additional visits during the oral weekly administration may be conducted per investigator's discretion. Upon restarting GS-6207 SC injection, an SC dosing visit will restart (eg, Week 52, Week 78), with follow-up visits in between occurring as specified in this protocol.

3.4. Duration of Treatment

Participants will be treated for at least 54 weeks. Following completion of the Functional Monotherapy Period (Cohort 1) or the oral lead-in Period (Cohort 2), participants will continue with study visits at Day 1 SC, Weeks 4, 10, 16, 22, 26, 36, and 52.

At the Week 52 visit, participants will be given the option to attend visits at Week 62, 78, 88, 104, 114, 130 and will continue to alternate between every 10 weeks and every 16 weeks. Participants willing to continue the study beyond Week 52 visit will receive SC GS-6207 every 6 months (26 weeks) ± 2 weeks from last injection starting at Week 52 visit, while continuing their OBR, until the product becomes accessible to participants through an access program, is commercially available, or until Gilead Sciences elects to discontinue the study in the country.

Participants who decide not to receive SC GS-6207 at Week 52 and not to continue the study will complete the study at Week 52 visit. Participants who decide to discontinue SC GS-6207 early and do not wish to continue to attend study visits through Week 52 visit or next scheduled SC dosing visit will return to the clinic for 30-Day, 90-Day and 180-Day Follow-Up visits after Early Termination visit. The 180-Day Follow-Up may be conducted via a phone call per the investigator's discretion.

3.5. Biomarker Testing

CCI [redacted]

[redacted].

CCI [redacted]

[redacted]

4. PARTICIPANT POPULATION

4.1. Number of Participants and Participant Selection

Approximately 100 participants may be enrolled in this study.

36 participants will be enrolled in Cohort 1, and up to 64 participants may be enrolled in Cohort 2.

4.2. Inclusion Criteria

Participants must meet all of the following inclusion criteria to be eligible for participation in this study:

1) Willing and able to provide written informed consent (participants ≥ 18 years of age) and assent (participants ≥ 12 and < 18 years of age) prior to performing study procedures. For participants ≥ 12 and < 18 years of age, parent or legal guardian willing and able to provide written informed consent prior to performing study procedures as required by local law

2) Adult aged ≥ 18 years (at all sites) or adolescent aged ≥ 12 and weighing ≥ 35 kg (at sites in North America and Dominican Republic)

3) Are receiving a stable failing ARV regimen for > 8 weeks before Screening and willing to continue the regimen until Day 1. Participants in Cohort 1 must also be willing to continue the failing regimen until completing the Functional Monotherapy Period (Day 1 to Day 14)

4) Have HIV-1 RNA ≥ 400 copies/mL at Screening

5) Have screening or available historical HIV resistance reports showing resistance to ≥ 2 antiretroviral medications from each of ≥ 3 of the 4 main classes of antiretroviral medications (NRTI, NNRTI, PI, INSTI). Resistance to FTC or 3TC associated with the presence of the M184V/I RT mutation cannot be used for the purpose of determining this eligibility criterion

6) Have ≤ 2 fully active ARV remaining from the 4 main classes that can be effectively combined to form a viable regimen in the opinion of the investigator based on resistance, tolerability, contraindication, safety, drug access, or acceptability to the participant. Refer to Table 5-4 for list of disallowed ARVs

7) Able and willing to receive an optimized background regimen together with GS-6207. Participants with an OBR without a fully active agent may be enrolled if the investigator considers that there is a favorable risk-benefit ratio for the participant. With prior approval from Gilead Sciences, components of the OBR may be investigational (ie, not-yet-approved)

8) A negative serum pregnancy test is required for all women at Screening

9) Participants of childbearing potential who engage in heterosexual intercourse must agree to use protocol specified method(s) of contraception as described in Appendix 4.

10) Lactating women must agree to discontinue nursing before administration of GS-6207

4.3. Exclusion Criteria

Participants who meet *any* of the following exclusion criteria are not to be enrolled in this study:

1) An opportunistic illness requiring acute therapy within the 30 days prior to screening

2) Active, serious infections (other than HIV-1 infection) requiring parenteral antibiotic or antifungal therapy within 30 days before screening

3) Active tuberculosis infection

4) Acute hepatitis within 30 days prior to Screening visit

5) Untreated or newly treated (< 3 months prior to screening) Hepatitis B Virus (HBV) infection. Participants may be enrolled regardless of the HBV serology criteria below if they are receiving treatment with anti-HBV activity and plan to continue the treatment during the study. Hepatitis B infection is defined as screening results showing either or both of:

    a. Positive HBV surface antigen

    b. Positive HBV core antibody and negative HBV surface antibody. Participants may be enrolled with these findings if they have HBV DNA < LLOQ

6) Hepatitis C virus (HCV) antibody positive and HCV RNA > LLOQ

7) A history of or current clinical decompensated liver cirrhosis (eg, ascites, encephalopathy, or variceal bleeding)

8) Treatment within three months prior to screening, or anticipated treatment during the study period with immunosuppressant therapies, hydroxyurea, foscarnet, radiation, or cytotoxic chemotherapeutic agents without prior approval from Sponsor prior to randomization. Agents disallowed in Table 5-4 may not be considered for approval.

9) Active malignancy requiring acute therapy (with the exception of local cutaneous Kaposi's sarcoma)

10) Current alcohol or substance use judged by the investigator to potentially interfere with the participant’s study compliance

11) Clinically significant abnormal ECG at the Screening visit

12) Any of the following laboratory values at screening:

    a. Estimated glomerular filtration rate (GFR) ≤ 50 mL/min using Cockcroft-Gault formula for participants ≥ 18 years of age {Cockcroft 1976} and Schwartz Formula for participants < 18 years of age for creatinine clearance

    b. ALT > 5 × upper limit of normal (ULN)

    c. Direct bilirubin > 1.5 × ULN

    d. Platelets < 50,000/mm^3

    e. Hemoglobin < 8.0 g/dL

13) Participation or planned participation in any other clinical trial (including observational trials) without prior approval from the sponsor throughout the study

14) Prior use of, or exposure to, GS-6207

15) Known hypersensitivity to the IMP, the metabolites, or formulation excipient

16) Use or planned use of exclusionary medications, refer to Section 5.4

17) Any other clinical condition or prior therapy that, in the opinion of the investigator, would make the participant unsuitable for the study or unable to comply with dosing requirements

5. INVESTIGATIONAL MEDICINAL PRODUCTS

5.1. Randomization, Enrollment, Blinding, and Treatment Codes Access

5.1.1. Randomization and Enrollment

Participants will be assigned a screening number using the Interactive Web Response System (IWRS) on the day of the Screening visit. Once eligibility has been confirmed and availability of OBR is confirmed, participants will be enrolled to either of the two cohorts as following:

- Cohort 1: Randomized in a 2:1 ratio to receive oral GS-6207 or placebo to match oral GS-6207 for 14 days starting on Day 1, while they continue their existing regimen

  OR

- Cohort 2: Enrolled to receive oral GS-6207 together with an OBR

Randomization or enrollment may occur approximately three days prior to Day 1 visit.

Each eligible participant will be assigned a unique participant number using IWRS. Once a participant number has been assigned, it will not be reassigned to another participant.

5.1.2. Blinding

During the Functional Monotherapy Period in Cohort 1, the investigational site(s), participants, and Gilead Sciences will remain blinded to treatment assignment and HIV-1 RNA results at Days 2 and 8. As each participant completes the Functional Monotherapy Period, their treatment assignment will be unblinded by investigational site using IWRS to determine their treatment regimen in the Maintenance Period. To mitigate the risks of inadvertently releasing the treatment information to participants who are still receiving functional monotherapy, Gilead staff will not receive the treatment codes from IWRS until all participants in Cohort 1 have completed the Functional Monotherapy Period. In the situation when Gilead staff is able to identify possible treatment assignment of participants still on blinded treatment, based on treatment information (eg, randomization block) from individual participants that have already been unblinded, they will maintain the confidentiality of the unblinded information, and will not communicate the information to blinded sites as specified in Gilead procedural documents.

Furthermore, specified personnel may be unblinded based on their study role. The Pharmacokinetics File Administrator, or designee, in Bioanalytical Operations and/or Clinical Data Management who facilitates the data transfer of PK files between Gilead and vendors will remain unblinded. Individuals in Clinical Packaging and Labeling or Clinical Supply Management who have an Unblinded Inventory Manager role in the IWRS for purposes of study drug inventory management will remain unblinded. Individuals in Gilead Patient Safety (formerly known as Pharmacovigilance and Epidemiology [PVE]) responsible for safety signal detection, IND safety reporting, and/or expedited reporting of suspected unexpected serious

adverse reactions (SUSARs) may be unblinded to individual case data and/or group-level summaries. External (ie, contract research organizations [CROs]) biostatisticians and programmers will be unblinded to support safety data review specified in Section 7.8 of the protocol. Regulatory Quality and Compliance personnel in Research and Development may also be unblinded for purposes of supporting Quality Assurance activities and/or regulatory agency inspections.

5.1.3. Procedures for Breaking Treatment Codes

In the event of a medical emergency where breaking the blind is required to provide medical care to the participant, the investigator may obtain treatment assignment directly from the IWRS system for that participant. Gilead recommends but does not require that the investigator contact the Gilead Medical Monitor before breaking the blind. Treatment assignment should remain blinded unless that knowledge is necessary to determine participant emergency medical care. The rationale for unblinding must be clearly explained in source documentation and on the electronic case report form (eCRF), along with the date on which the treatment assignment was obtained. The investigator is requested to contact the Gilead Medical Monitor promptly in case of any treatment unblinding.

All participants will be followed until study completion, unless consent to do so is specially withdrawn by the participant.

5.2. Description and Handling of GS-6207 (Lenacapavir) Injection, GS-6207 (Lenacapavir) Tablets and Placebo to Match GS-6207 (Lenacapavir) Tablets

5.2.1. Formulation

GS-6207 Injection, 309 mg/mL, is a clear, yellow to brown solution for SC injection. In addition to the active ingredient (GS-6207 sodium salt), GS-6207 Injection, 309 mg/mL contains the following inactive ingredients: polyethylene glycol 300 and water for injection.

GS-6207 tablets, 300 mg are capsule-shaped, film-coated beige tablets, debossed with “GSI” on one side of the tablet and “62L” on the other side of the tablet. Each tablet core contains the equivalent of 300 mg GS-6207 free acid in the form of GS-6207 sodium salt. In addition to the active ingredient, GS-6207 tablets, 300 mg contain the following inactive ingredients: microcrystalline cellulose, mannitol, poloxamer 407, copovidone, croscarmellose sodium, magnesium stearate, polyethylene glycol, polyvinyl alcohol, talc, titanium dioxide, iron oxide red, iron oxide black, and iron oxide yellow.

Placebo to match GS-6207 tablets, 300 mg, are capsule-shaped, film-coated beige tablets, debossed with “GSI” on one side of the tablet and “62L” on the other side of the tablet. Placebo to match GS-6207 tablets, 300 mg contain the following inactive ingredients: lactose, microcrystalline cellulose, croscarmellose sodium, magnesium stearate, polyethylene glycol, polyvinyl alcohol, talc and titanium dioxide, iron oxide red, iron oxide black, and iron oxide yellow.

5.2.2. Packaging and Labeling

GS-6207 injection, 309 mg/mL, is supplied as a sterile solution packaged in a single use, clear vial fitted with a rubber stopper and an aluminum flip-off seal.

GS-6207 tablets, 300 mg are packaged in white, high density polyethylene (HDPE) bottles. Each bottle contains 4 or 5 tablets, silica gel desiccant and polyester packing material. Each bottle is enclosed with a white, continuous thread, child-resistant polypropylene screw cap fitted with an induction-sealed, and aluminum-faced liner.

Placebo to match GS-6207 tablets, 300 mg are packaged in white, HDPE bottles. Each bottle contains 5 tablets, silica gel desiccant and polyester packing material. Each bottle is enclosed with a white, continuous thread, child-resistant polypropylene screw cap fitted with an induction-sealed, and aluminum-faced liner.

Study drugs to be distributed to centers in the US, EU and other participating countries shall be labeled to meet applicable requirements of the US FDA, EU Guideline to Good Manufacturing Practice - Annex 13 (Investigational Medicinal Products), the J-GCP (Ministerial Ordinance on Good Clinical Practice for Drugs) and/or other local regulations.

5.2.3. Storage and Handling

GS-6207 injection, 309 mg/mL should be stored below 30°C (86°F), protected from light. Storage conditions are specified on the label.

GS-6207 tablets, 300 mg should be stored below 30°C (86°F). Storage conditions are specified on the label. Placebo to match GS-6207 tablets, 300 mg should be stored below 30°C (86°F). Storage conditions are specified on the label.

Until dispensed to the participants, all study drugs should be stored in a securely locked area, accessible only to authorized site personnel. To ensure the stability and proper identification, study drugs should not be stored in a container other than the container in which they were supplied. Consideration should be given to handling, preparation, and disposal through measures that minimize drug contact with the body. Appropriate precautions should be followed to avoid direct eye contact or exposure when handling.

5.3. Dosage and Administration of GS-6207

Oral GS-6207 and GS-6207 injection for SC administration will be provided by Gilead Sciences. Participants will be responsible for continuing their pre-existing regimen. The OBR will be prescribed by the investigator, and the participant is responsible for obtaining the OBR prior to their Day 1 visit. Medications disallowed as components of the OBR for pharmacokinetic reasons are provided in Section 5.4. Investigators should confirm the availability of the OBR prior to randomizing or enrolling eligible participants in IWRS.

Oral GS-6207 or Placebo to Match GS-6207 will be administered on Days 1, 2, and 8. Participants who receive Placebo to Match GS-6207 on Days 1, 2, and 8 will receive oral GS-6207 on Day 15, 16, and 22. Oral GS-6207 will be administered without regard to food. Oral dosing is presented in Table 5-1.

Table 5-1. Oral Dosing of GS-6207 or Placebo to Match

Study Visits	Treatment
Days 1 or 15*	600 mg
Days 2 or 16*	600 mg
Days 8 or 22*	300 mg

* Days 15, 16 and 22 visits are applicable only to participants who receive placebo to match in the Functional Monotherapy Period at Days 1, 2, and 8.

GS-6207 927 mg injection, 309 mg/mL will be administered in the abdomen via SC injections on Day 1 SC, Weeks 26, 52, and every 6 months (26 weeks) ± 2 weeks from last injection thereafter. GS-6207 injections should be administered at different abdominal sites no more than 15 minutes apart when possible. Each SC GS-6207 dosing should occur 6 months (26 weeks) ± 2 weeks from the last injection.

The entire initial oral lead-in period and all subsequent SC GS-6207 administration will occur on-site (for Oral Bridging Period, see Section 5.4).

Study drug (oral and SC GS-6207) can be administered without regard to food.

If a participant is not dosed within the protocol visit windows, and the investigator believes that it is in the participant's medical interest to continue to receive GS-6207, Gilead should be contacted immediately, and the participant may continue to receive GS-6207 with the approval of the Gilead Medical Monitor. Additional oral GS-6207 doses may be needed prior to subsequent SC dosing (Section 5.4).

For all participants, the date and time of last meal prior to dosing will be collected.

5.4. Dosing and Administration of Oral Weekly Bridging of GS-6207

Participants may require oral weekly bridging if an SC injection of GS-6207 cannot be administered for any reason within the protocol visit window.

- If 26 to 28 weeks elapses since the last SC injection and if clinically appropriate to continue GS-6207, start oral GS-6207 300 mg (1 tablet) once a week at Oral Bridging visit(s) and continue weekly dosing on the same day of the week.
- If more than 28 weeks elapse since the last SC injection and if clinically appropriate to continue GS-6207, restart the oral lead-in at the Oral Bridging visits and continue oral weekly dosing on the same day of the week, until SC GS-6207 is administered (Table 5-2).

Table 5-2. Dosing Schedule for Oral Weekly Bridging and Resumption of SC GS-6207 Injection

26 to 28 weeks of elapse since the last SC injection	More than 28 weeks of elapse since the last SC injection	GS-6207 Dose
**Treatment time from oral start**	**Dose**	
NA	Day 1[a]	Oral 600 mg (2 × 300 mg tablets)
NA	Day 2	Oral 600 mg (2 × 300 mg tablets)
Day 1[a]	Day 8; continue oral weekly GS-6207 on the same day of the week as first dose until GS-6207 SC is administered	Oral 300 mg (1 × 300 mg tablet)
Day 8; continue oral weekly GS-6207 on the same day of the week as first dose until GS-6207 SC is administered	Not applicable	Oral 300 mg (1 × 300 mg tablet) once a week
SC administration restart (within 1 week of the oral weekly 300 mg dose [as early as after Day 1 dosing])	SC administration restart (within 1 week of the oral weekly 300 mg dose [as early as after Day 8 dosing])	SC 927 mg every 6 months[a]

NA = not applicable; SC = subcutaneous

a Day 1 oral dosing and SC GS-6207 will be administered on site, otherwise, all other oral dosing will be administered at home, but can be done on site at the investigator's discretion.

- For oral weekly bridging, the administration of the first dose will occur on-site and up to 2 bottles of GS-6207 will be given to the participant to take oral GS-6207 at home; further on-site oral dosing may occur at investigator's discretion. Up to 2 additional bottles may be given to the participant as needed upon completion of previously dispensed bottles.

- Participants may continue receiving oral weekly GS-6207 300 mg until they can receive their next SC GS-6207 injection.

- If oral weekly GS-6207 administration is expected to be needed beyond 12 consecutive weeks, Gilead should be contacted. Additional oral weekly doses can be provided after assessment unless other guidance is provided by Gilead.

- Monitoring of participants should continue according to the study protocol. Oral Bridging visit should be completed every 10 to 12 weeks. Additional visits during the oral weekly administration may be conducted per investigator's discretion

- Missed dose recommendations for oral weekly bridging are provided in Table 5-3. The scheduled dosing day of the week should not change due to the missed dose of GS-6207.

Table 5-3. Missed Dose Recommendations for GS-6207 During Oral Weekly Bridging

Number of days since initial missed schedule dose	Recommendation	Example
1 to 6 days<br>(1 missed dose)	Take 1 dose as soon as possible, then resume normal schedule, taking 1 dose on the next scheduled day	Participant forgets to take dose on Monday (scheduled) but remembers before the next scheduled dose day (ie, Tuesday-Sunday). Take 1 dose as soon as possible, then take 1 dose on the following Monday as scheduled.
7 to 14 days<br>(1-2 missed doses)	Take 2 doses as soon as possible, then resume normal schedule on scheduled day. If participant remembers on scheduled dosing day, then take 2 doses only. Never take 3 doses on the same day.	Participant forgets to take dose on Monday (scheduled) but remembers the following Monday. Take 2 doses on the second Monday and resume dosing schedule (1 dose on Mondays).<br>Participant forgets to take dose on 2 consecutive Mondays (scheduled) but remembers a few days later (ie, Tuesday-Sunday following second missed dose) before the third Monday. Take 2 doses as soon as possible, then take 1 dose the next Monday as scheduled.<br>Participant forgets to take dose on 2 consecutive Mondays (scheduled) but remembers on the third Monday. Take 2 doses on the third Monday and resume dosing schedule (1 dose on Mondays). Never take 3 doses on the same day.
More than 14 days<br>(3 or more missed doses)	Assess whether clinically appropriate to continue oral weekly bridging.<br>Consider checking HIV-1 RNA.	Participant forgets to take dose on 3 consecutive Mondays (scheduled). Clinical assessment needed.

5.5. Prior and Concomitant Medications

Clinical data indicate GS-6207 is a substrate of P-gp transporters, CYP3A and UGT1A1 enzymes. GS-6207 is a moderate inhibitor of CYP3A and a weak inhibitor of breast cancer resistance protein (BCRP), and P-gp. Concomitant use of GS-6207 with some medications or herbal/natural supplements that are inhibitors and inducers of CYP3A, UGT1A1 or P-gp may result in increased or decreased exposure of GS-6207, respectively.

Concomitant use of GS-6207 with some medications or herbal/natural supplements that are substrates of CYP3A, P-gp or BCRP may result in increased exposure of these medications.

Representative medications listed in Table 5-4 and herbal/natural supplements are currently excluded or should be used with caution while participating in this study; this table is not exhaustive. For medications that may be substrates of P-gp, CYP3A, UGT1A1 or BCRP, or those that may be inducers, the investigator should reach out to Gilead for guidance.

Participants should discontinue disallowed concomitant medications 30 days prior to initiation of study drug, unless otherwise specified.

Table 5-4. List of Representative Medications that are Prohibited or To Be Used with Caution due to the Potential for Drug-Drug Interaction with GS-6207

Medication Class	Disallowed Medications	Use Discouraged and To Be Used with Caution
Anti-coagulants		Dabigatran etexilate: monitoring and/or dose reduction may be needed for certain populations per prescribing information
Anti-convulsants	Carbamazepine, Oxcarbazepine, Phenobarbital, Phenytoin	
Anti-mycobacterials	Rifampin, Rifabutin, Rifapentine**	
Anti-retroviral agents*	ATV, ATV/co**, ATV/r, EFV, ETV, NVP, TPV**	
Digoxin		Digoxin: Concomitant use of oral GS-6207 may result in increased levels; use with caution and with appropriate monitoring of serum digoxin levels
Ergot derivatives	Ergotamine, Ergonovine, Dihydroergotamine, Methylergonovine, Ergometrine	
Herbal/Natural Supplements	St. John's Wort, Echinacea, Milk thistle (eg, silymarin), Chinese herb sho-saiko-to (or Xiao-Shai-Hu-Tang)	
HMG-CoA Reductase Inhibitors		Concentrations of statins may increase with GS-6207. Start with the lowest dose and titrate to clinical response. For each of the following statins, the maximum allowed dose is:<br>Simvastatin: 10 mg<br>Lovastatin**: 20 mg<br><br>Careful monitoring for signs and symptoms of muscle weakness or myopathy, including rhabdomyolysis
Phosphodiesterase-5 Inhibitors		Sildenafil, Vardenafil, Tadalafil: It is recommended that a single dose of Sildenafil no more than 25 mg in 48 hours, Vardenafil no more than 2.5 mg in 72 hours, or Tadalafil no more than 10 mg in 72 hours be coadministered.<br>Use of tadalafil for the treatment of pulmonary arterial hypertension is not recommended
Sedatives/Hypnotics		Midazolam, Triazolam
Systemic Corticosteroids	All agents, including dexamethasone	

* ATV, atazanavir; co, cobicistat; EFV, efavirenz; ETV, etravirine; NVP, nevirapine; r, ritonavir; TPV, tipranavir
** Not approved in Japan

Physicians should refer to the package insert for the OBR medications for guidance on concomitant medications.

Investigators should make every effort to maintain the initial OBR. However, if clinically indicated for efficacy, safety, or tolerability reasons, investigators may change the OBR after consulting with Gilead's Medical Monitor. The reason for any changes to the OBR will be documented in the participant's source documents and the eCRF.

Medications to treat disease conditions **excluded** from the protocol are not listed under this Concomitant Medication section and are disallowed in the study. Medications for malignancy are not included.

Should participants have a need to initiate treatment with any disallowed concomitant medication, the medical monitor must be consulted prior to initiation of the new medication. In instances where disallowed medication is initiated prior to discussion with the sponsor, the investigator must notify Gilead as soon as they are aware of the use of the medication.

5.6. Accountability for Investigational Medicinal Product

The investigator (or designee, eg, study center pharmacist) is responsible for ensuring adequate accountability of all used and unused study drug. This includes acknowledgment of receipt of each shipment of study drug (quantity and condition). Each study site must keep accountability records that capture:

- The date received and quantity of study drug kits
- The date, participant number, and the study drug kit number dispensed
- The date, quantity of used and unused study drug returned, along with the initials of the person recording the information

5.6.1. Investigational Medicinal Product Return or Disposal for GS-6207 and Placebo to Match GS-6207 Tablets

Gilead recommends that used and unused study drug supplies be destroyed at the site. If the site has an appropriate SOP for drug destruction as determined by Gilead, the site may destroy used (empty or partially empty) and unused study drug supplies in accordance with that site's approved SOP. A copy of the site's approved SOP will be obtained for eTMF. If study drug is destroyed on site, the investigator must maintain accurate records for all study drugs destroyed. Records must show the identification and quantity of each unit destroyed, the method of destruction, and the person who disposed of the study drug. Upon study completion, copies of the study drug accountability records must be filed at the site. Another copy will be returned to Gilead.

If the site does not have an appropriate standard operating procedure (SOP) for drug destruction, used and unused study drug supplies are to be sent to the designated disposal facility for destruction. The study monitor will provide instructions for return.

The study monitor will review study drug supplies and associated records at periodic intervals.

For both disposal options listed above, the study monitor must first perform drug accountability during an on-site monitoring visit, unless otherwise agreed with the sponsor and documented.

6. STUDY PROCEDURES

The study procedures to be conducted for each participant enrolled in the study are presented in tabular form in Appendix 2. and described in the text that follows.

The investigator must document any deviation from the protocol procedures and notify the Gilead or contract research organization (CRO).

6.1. Participant Enrollment and Treatment Assignment

Entry into screening does not guarantee enrollment into the study. In order to manage the total study enrollment, Gilead, at its sole discretion, may suspend screening and/or enrollment at any site or study-wide at any time.

6.2. Pre-treatment Assessments

6.2.1. Screening Assessments

Participants will be screened within 42 days prior to enrollment in the study. Each participant will be assigned a unique screening number using the IWRS.

The following will be performed and documented at Screening:

- Obtain written informed consent-related events, available historical genotype/phenotype reports, available HIV-1 treatment history, substance (ie, illicit drug) use and prior medications within 30 days of the Screening visit
- Complete physical examination (urogenital/anorectal examinations will be performed at the discretion of the investigator) including, vital signs, body weight, and height
- 12-lead ECG performed supine
- Obtain blood and urine samples as noted in Section 6.5
- Record any SAEs and all AEs related to protocol-mandated procedures occurring after signing of the consent form
- Select an OBR based on the screening and/or available historical HIV resistance reports.
  - Availability of selected OBR must be confirmed prior to randomization/enrollment and participant must be able to initiate OBR as specified in the protocol
- Return to the clinic between 14 and 30 days after the Screening visit, for a Cohort Selection visit. HIV-1 RNA results from this visit will be used to determine whether eligible participants will participate in Cohort 1 or Cohort 2.
  - Once enrollment in Cohort 1 is complete, Cohort Selection visit will not be required.

Participants meeting all the inclusion criteria and none of the exclusion criteria will return to the clinic within 42 days after screening for a Day 1 visit. Participants will be instructed to continue their failing regimen.

Participants not meeting one or more inclusion criteria and/or meeting one or more exclusion criteria may be rescreened on a case-by-case basis upon written approval from Gilead Medical Monitor or Study Director.

From the time of obtaining informed consent through the first administration of study drug, record all SAEs, as well as any AEs related to protocol-mandated procedures on the AEs electronic case report form (eCRF). All other untoward medical occurrences observed during the screening period, including exacerbation or changes in medical history are to be considered medical history. See Section 7 Adverse Events and Toxicity Management for additional details.

6.3. Treatment Assessments

6.3.1. Functional Monotherapy Period and Oral Lead-in Period Assessments

Cohort 1: Eligible participants will be randomized in Cohort 1 if they have a < 0.5 $\log_{10}$ HIV-1 RNA decline compared to the Screening visit and HIV-1 RNA ≥ 400 copies/mL at the Cohort Selection visit. Approximately three days prior to the Day 1 visit, investigator or designee may randomize the participant to receive oral GS-6207 or placebo to match GS-6207 and obtain a participant number using IWRS. Cohort 1 participants will have a Functional Monotherapy Period. Participants will continue to receive their failing regimen during this period.

Cohort 2: Eligible participants will be enrolled in Cohort 2 if Cohort 1 is fully enrolled or if they do not meet the criteria for randomization in Cohort 1 (eg, they had a ≥ 0.5 $\log_{10}$ HIV-1 RNA decline compared to the Screening visit and/or HIV-1 RNA < 400 copies/mL at the Cohort Selection visit). Approximately three days prior to the Day 1 visit, investigator or designee may enroll the participant using IWRS and obtain a participant number. Cohort 2 participants will have an oral lead-in period and will initiate an OBR (as prescribed by the investigator) on Day 1.

All participants will complete the following study visits: Days 1, 2, 5 (± 1 day) CCI [redacted] The following procedures are to be completed at the Day 1 visit, prior to study drug dosing and at Days 2, 5 (± 1 day) and 8, unless otherwise noted.

- Prior to completion of other study procedures, participants ≥ 18 years of age at the Day 1 visit will read the Patient Reported Outcomes, if available, by himself/herself and provide answers directly onto the questionnaires.
  - Symptoms Distress Module, Short Form Health Survey (SF-36), EQ-5D-5L at Day 1
- Review of AEs and changes in concomitant medications
- Complete physical examination (urogenital/anorectal examinations will be performed at the discretion of the investigator) (Day 1 only) or symptom directed physical examination as needed at Days 2 and 8

- Vital signs and body weight (except at Day 5)
- Obtain blood and urine samples collection as noted in Section 6.5
- After completion of study assessments, Cohort 1 participants will receive oral GS-6207 600 mg, 600 mg, and 300 mg or placebo to match on Days 1, 2, and 8 respectively.
  - Cohort 1 participants will continue on their failing regimen
- After completion of study assessments, Cohort 2 participants will receive oral GS-6207 600 mg, 600 mg, and 300 mg on Days 1, 2, and 8 respectively.
  - Cohort 2 participants will initiate an OBR (as prescribed by the investigator) at Day 1. Participants will take last dose of their failing regimen prior to Day 1 visit
- All study drug dosing will occur at the study center without regard to food
- Document study drug administration
- After each participant completes the Functional Monotherapy Period, their treatment assignment will be unblinded

6.3.2. Maintenance Period Assessments

All participants will complete a Maintenance Period. The Gilead Medical Monitor should be consulted prior to administration of SC GS-6207 in the Maintenance Period if a participant was not administered all doses of oral GS-6207 during the Functional Monotherapy Period in Cohort 1 or the Oral Lead-in Period in Cohort 2.

All participants will complete the following study visits: Day 1 SC (14 days after the first dose of oral GS-6207), Weeks 4, 10, 16, 22, 26, 36, and 52. At the Week 52 visit, participants will be given an option to continue receive SC GS-6207 927 mg and to complete study visits at Weeks 62, 78, 88, 104, 114, 130 and will continue to alternate between every 10 weeks and every 16 weeks. Participants willing to continue the study beyond Week 52 visit will receive SC GS-6207 every 6 months (26 weeks) ± 2 weeks from last injection starting at Week 52 visit, while continuing their OBR. Study visits Week X are identified by the number of weeks that have elapsed since the Day 1 SC visit when GS-6207 is first administered by injection and excludes Oral Bridging Periods.

Cohort 1B participants will complete the following study visits prior to receiving SC GS-6207: Days 15, 16, 19 (±1) CCI [redacted] and 22. They will receive oral GS-6207 600 mg, 600 mg, and 300 mg on Days 15, 16, and 22, respectively.

Participants may require oral bridging if an SC injection of GS-6207 cannot be administered within the protocol visit window. In case of Oral Bridging initiation, participants will receive oral GS-6207 as specified in Section 5.4 and complete Oral Bridging visits. An Oral Bridging visit should be completed every 10 to 12 weeks. Additional visits during the oral weekly

administration may be conducted per investigator's discretion. Upon restarting GS-6207 SC injection, an SC dosing visit will restart (eg, Week 52, Week 78), with follow-up visits in between occurring as specified in this protocol.

Week 4 visit through Week 52 visit are to be completed within ± 2 days of the protocol-specified visit date based on the Day 1 SC visit. Following the completion of Week 52 visit, visits are to be completed within ± 6 days of the protocol-specified visit date. Each SC GS-6207 dosing should occur within 26 and 28 weeks of the previous SC GS-6207 dosing.

The following procedures are to be completed at all visits unless otherwise noted.

- Participants ≥ 18 years of age at the Day 1 visit will read the Patient Reported Outcomes, if available, by himself/herself and provide answers directly onto the questionnaires.
  - Participants will complete Symptoms Distress Module, Short Form Health Survey (SF-36), EQ-5D-5L at Weeks 4, 16, 26 and 52 (these questionnaires should be completed prior to the completion of other study procedures).
  - Participants to complete the Numeric Pain Rating Scale at Day 1 SC, Weeks 26 and 52 (this questionnaire should be completed after the participant received their SC GS-6207 injections).
- Review of AEs and changes in concomitant medications.
- Complete physical examination (Day 15 [Cohort 1B only], Day 1 SC, Weeks 26 and 52) (urogenital/anorectal examinations will be performed at the discretion of the investigator) or symptom directed physical examination as needed (except at Days 5 and 19).
- Obtain vital signs and body weight (except at Days 5 and 19).
- Obtain blood and urine samples as noted in Section 6.5.
- Cohort 1 participants who received oral GS-6207 during randomized phase will receive SC GS-6207 927 mg and initiate their OBR on Day 1 SC visit (14 days after the first dose of oral GS-6207) **(Cohort 1A).** Participants will continue to receive SC GS-6207 927 mg every 6 months (26 weeks) ± 2 weeks from last injection thereafter while continuing their OBR.
- Cohort 1 participants who received placebo to match GS-6207 during randomized phase will receive oral GS-6207 600 mg and initiate their OBR on the Day 15 visit after completion of Day 15 assessments **(Cohort 1B).** Participants will receive oral GS-6207 600 mg and 300 mg on Days 16 and 22 respectively while continuing their OBR.
- Cohort 1B participants will receive SC GS-6207 927 mg at the Day 1 SC visit while continuing on their OBR (14 days after the first dose of oral GS-6207). Participants will continue to receive SC GS-6207 927 mg every 6 months (26 weeks) ± 2 weeks from last injection thereafter while continuing their OBR.

- Cohort 2 participants will receive SC GS-6207 927 mg at the Day 1 SC visit while continuing on their OBR (14 days after the first dose of oral GS-6207). Participants will continue to receive SC GS-6207 927 mg every 6 months (26 weeks) ± 2 weeks from last injection thereafter while continuing their OBR.
- SC study drug administration will occur at the study center without regard to food.
- Provide ISR assessment worksheet and instruct the participants to measure and report ISRs following the administration of the SC injections.
- For all participants on oral weekly bridging, the first dose of oral GS-6207 will be administered at the study center without regard to food and up to 2 bottles will be provided to the participant with instructions to take the remaining doses at home once a week (1 × 300 mg GS-6207) without regard to food on the same day of the week that the first dose was given.
- Document study drug administration.
- Document study drug accountability.
- Participants who meet the criteria for virologic failure will be managed according to the Management of Virologic Failure Section 6.7.

6.4. Post-Treatment Assessments

6.4.1. Early Termination Visit Assessments

6.4.1.1. Early Termination visit after Day 1 and prior to Day 1 SC

If a participant discontinues oral GS-6207 or decides not to initiate SC GS-6207 at Day 1 SC, an Early Termination visit should be performed within 72 hours of decision to discontinue the study drug. Participants in Cohort 1 will be asked to continue to attend study visits until completion of the Functional Monotherapy Period. Participant will be required to complete the 90-Day Follow-Up visit. Refer to Section 6.4.2

6.4.1.2. Early Termination visit after Day 1 SC and prior to Week 52

If a participant decides to discontinue SC GS-6207 prior to Week 52, an Early Termination visit should be performed within 72 hours of decision to discontinue study drug.

The participant will be asked to continue attending the scheduled study visits through the Week 52 visit.

- If the participant decides to discontinue SC GS-6207 and agrees to continue to attend study visits through Week 52, no follow-up visits are required.
- If the participant decides to discontinue SC GS-6207 and does not agree to continue to attend study visits through Week 52, the 30-Day, 90-Day, and 180-Day Follow-Up visits are required. Refer to Section 6.4.2. The 180-Day Follow-Up may be conducted via a phone call per the investigator's discretion.

6.4.1.3. Early Termination Visit After Week 52

If a participant continues the study after completing the Week 52 visit, but discontinues SC GS-6207 prior to study completion, an Early Termination visit should be performed.

- If the participant discontinues GS-6207 (SC and oral) and continues to attend study visits through their next SC dosing visit, all assessments, except SC dosing, will be performed at the last visit. No further follow-up visits are required.

- If the participant discontinues SC GS-6207 and does not continue to attend study visits through the participant's next SC dosing visit, the 30-Day, 90-Day and 180-Day Follow-Up visits are required. Refer to Section 6.4.2. The 180-Day Follow-Up may be conducted via a phone call per the investigator's discretion.

- If a participant discontinues oral GS-6207 during the Oral Bridging Period and does not continue to attend study visits through their next SC dosing visit, the participant will be required to complete the 90-Day Follow-Up visit (see Section 6.4.2).

If there are any abnormal laboratory results with a possible or probable causal relationship with the study drug, every attempt should be made to keep the participant in the study and repeat those laboratory tests weekly (or as often as deemed prudent by the investigator) until the abnormality is resolved, returns to baseline, or is otherwise explained. If this is not possible or acceptable to the participant or investigator, the participant may be withdrawn from the study.

If there are any AEs, every attempt should be made to keep the participant in the study and should be followed up until the AE is resolved or stable. Gilead may request that certain AEs be followed beyond the protocol-defined follow-up period. If this is not possible or acceptable to the participant or investigator, the participant may be withdrawn from the study.

The following evaluations should be performed at the Early Termination visit:

- Review of AEs and changes in concomitant medications

- Complete physical examination (urogenital/anorectal examinations will be performed at the discretion of the investigator)

- Vital signs measurement (blood pressure, pulse, respiration rate, and temperature)

- Weight

- Obtain blood and urine samples as noted in Section 6.5

- Counsel participant regarding the importance of continuing a complete ARV therapy in accordance to standard of care, and refer patient to an appropriate HIV treatment facility

6.4.2. 30-Day, 90-Day, and 180-Day Follow-Up Visits

The assessments below will be completed for participants who are required to complete 30-Day, 90-Day, and/or 180-Day Follow-Up visits as noted in Section 6.4.1. Follow-up visits will be scheduled based on the date of the Early Termination visit. No follow-up visits are required for those participants who do not receive SC GS-6207 at the Week 52 visit.

For scheduling the follow-up visits, a ± 6-day window may be used.

The following evaluations are to be completed at the follow-up visits:

- Review of AEs and changes in concomitant medications
- Symptom-directed physical examination
- Vital signs measurement (blood pressure, pulse, respiration rate, and temperature), including weight
- Obtain blood and urine samples as noted in Section 6.5

At the 30-Day, 90-Day or 180-Day Follow-Up visit, if there are any abnormal laboratory results indicating that there is a possible or probable causal relationship with the study drug, every attempt should be made to repeat those laboratory tests weekly (or as often as deemed prudent by the investigator) until the abnormality is resolved, returns to baseline, or is otherwise explained. If this is not possible or acceptable to the participant or investigator, the participant may be withdrawn from the study.

If there are any AEs, every attempt should be made to keep the participant in the study and should be followed up until resolution or until the AE is stable, if possible. Gilead may request that certain AEs be followed beyond the protocol-defined follow-up period.

6.5. Clinical Laboratory Assessments

Blood and urine samples will be collected throughout the study as outlined below and in Appendix 2. Study Procedures Table.

6.5.1. Blood Samples

Blood sample collection for the following laboratory analyses will be performed at every visit, unless specified:

- Serum pregnancy test for all women (Screening visit only)
- Serum follicle-stimulating hormone (FSH) test (FSH test is required for women who are < 54 years old and have stopped menstruating for ≥ 12 months but do not have documentation of ovarian hormonal failure) (Screening visit only)

- Chemistry profile: alkaline phosphatase, AST, ALT, GGT, total bilirubin, direct and indirect bilirubin, total protein, albumin, LDH, CPK, bicarbonate, BUN, calcium, chloride, creatinine, glucose, lipase, magnesium, phosphorus, potassium, sodium, uric acid (except at the Cohort Selection, Days 2, 5, 16 and 19 visits)

- Estimated GFR (except at the Cohort Selection, Days 2, 5, 16 and 19 visits) according to:

  — Cockcroft-Gault formula for Creatinine clearance for participants ≥ 18 years of age

Men:

$$\frac{(140 - \text{age in years}) \times (\text{wt in kg})}{72 \times (\text{serum creatinine in mg/dL})} = \text{CLcr (mL/min)}$$

Women:

$$\frac{(140 - \text{age in years}) \times (\text{wt in kg}) \times 0.85}{72 \times (\text{serum creatinine in mg/dL})} = \text{CLcr (mL/min)}$$

  — Schwartz Formula for participants < 18 years of age

Adolescent boys ≥ 12 years of age: $0.70 \times L / S_{Cr}$ (L is height in cm)

Adolescent girls ≥ 12 years of age: $0.55 \times L / S_{Cr}$ (L is height in cm)

- Hematology profile: complete blood count (CBC) with differential and platelet count (except at the Cohort Selection visit, Days 2, 5, 16 and 19 visits)

- CD4+ cell count (except at the Cohort Selection visit, Days 2, 5, 16 and 19 visits)

- Plasma HIV-1 RNA (except at Day 5 and 19)

- HIV-1 genotype and phenotype for PR, RT, IN, and capsid at Screening visit and any subsequent visit with virologic failure (described in Section 6.7).

- Hepatitis B virus (HBV) serologies (HBV surface antigen, HBV core antibody, HBV surface antibody) (Screening only)

- Hepatitis C virus (HCVAb) serology (Screening only)

- CCI [redacted]

[redacted]

PK Sampling

PK sampling will occur relative to dosing of GS-6207 at the following time points for all participants:

- Day 1: Predose (within 30 minutes of dosing), 1, 2, 4, 6 and 8 hours post dose
- Day 2 and 8:
  - Predose (within 30 minutes of dosing)
  - A single timed PK sample between 1 and 6 hours post dose
- Days 5 (if visit occurs): A single anytime PK sample
- Day 15 (for Cohort 1B participants only): Predose (within 30 minutes of dosing), 1, 2, 4, 6, and 8 hours post dose
- Day 16 and 22 (for Cohort 1B participants only):
  - Predose (within 30 minutes of dosing)
  - A single timed PK sample between 1 and 6 hours post dose
- Days 19 (for Cohort 1B participants only, if visit occurs): A single anytime PK sample
- During the Maintenance Period, at all visits without SC GS-6207 injections, including the Oral Bridging visits, if applicable: A single anytime PK sample will be collected
- During the Maintenance Period, at all visits with SC GS-6207 injections, including when SC GS-6207 injection is resumed after the Oral Bridging visits, if applicable: A single predose (within 30 minutes of dosing) PK sample will be collected (while participants are continuing to receive study drug)

6.5.2. Blood and Urine Storage Samples

Any residual blood (PK and virology samples) and urine samples collected at all visits (except the Screening and Early Termination visits) will be frozen and stored. These stored blood and urine samples may be used by the Sponsor or its research partners for HIV-1 genotyping/phenotyping assays or their development, for retesting the amount of HIV-1 in the blood, for measurement of antiviral drug levels in the blood, or for future testing to learn more about how the study drug has worked against HIV-1 or clinical laboratory testing to provide additional safety data. No human genetic testing will be performed using these samples without consent of study participants.

At the conclusion of this study, these samples may be retained in storage by Gilead Sciences, Inc. for a period up to 15 years.

6.5.3. Urine Samples

Urine samples will be collected for the following laboratory analyses at every study visit, unless otherwise specified:

- Urinalysis and urine chemistry: including color & clarity, specific gravity, pH, glucose, ketones, bilirubin, urobilinogen, blood, nitrite, leukocyte esterase and microscopic (if microscopic elements are seen), urine protein, albumin, creatinine, phosphate, calcium, magnesium and uric acid (except at Cohort Selection visit, Days 2, 5, 16 and 19 visits)
- Urine pregnancy test for women of childbearing potential (except at Screening, Cohort Selection visit, Days 2, 5, 16, and 19 visits)
  - If the test is positive, confirmatory serum test should be performed and study drug dosing should be delayed until results obtained.
- Urine storage for possible additional clinical testing (except at Screening, Cohort Selection, Day 5, Day 19, and at 30, 90, and 180 Day Follow-Up visits, as applicable)

6.6. Criteria for Discontinuation of Study Treatment

Study medication may be discontinued in the following instances:

- Intercurrent illness that would, in the judgment of the investigator, affect assessments of clinical status to a significant degree. Following resolution of intercurrent illness, the participant may resume study dosing at the discretion of the investigator.
- Unacceptable toxicity, or toxicity that, in the judgment of the investigator, compromises the ability to continue study-specific procedures or is considered to not be in the participant's best interest
- Lack of Efficacy; participants may continue to receive study medication if the investigator believes that there is medical benefit in doing so
- Participant request to discontinue for any reason
- Participant noncompliance
- Pregnancy during the study; refer to Appendix 4.
- Discontinuation of the study at the request of Gilead, a regulatory agency or an institutional review board (IRB) or independent ethics committee (IEC)

6.7. Management of Virologic Failure

Participants who experience suboptimal virologic response (SVR), virologic rebound (VR), or are viremic at their last visit, as defined below, will be considered to have virologic failure for the purposes of resistance analysis.

6.7.1. Suboptimal Virologic Response (SVR)

Suboptimal virology response is defined as:

- HIV-1 RNA ≥ 50 copies/mL **and** < 1 log_{10} HIV-1 RNA reduction from the start of oral GS-6207 (Day 1 for Cohort 1A and Cohort 2 participants, or Day 15 for Cohort 1B participants) at the Week 4 visit

Following the first instance of SVR at Week 4, participants will be asked to return to the clinic for a scheduled or unscheduled blood draw (2 to 3 weeks after first SVR visit) for confirmation of SVR. A plasma sample from either the first instance or the SVR confirmation visit will be tested for HIV-1 capsid genotypic and phenotypic resistance. In addition, if SVR is confirmed, a plasma sample from the SVR confirmation visit will be tested for HIV-1 PR, RT, and IN genotypic and phenotypic resistance.

6.7.2. Virologic Rebound (VR)

Virologic rebound is defined as:

- At any visit, after achieving HIV-1 RNA < 50 copies/mL, a rebound in HIV-1 RNA to ≥ 50 copies/mL, which is subsequently confirmed at the following scheduled or unscheduled visit; OR

- At any visit, a > 1 log_{10} increase in HIV-1 RNA from the nadir which is subsequently confirmed at the following scheduled or unscheduled visit.

At any visit after achieving HIV-1 RNA < 50 copies/mL, if the HIV-1 RNA is ≥ 50 copies/mL and < 200 copies/mL, a reflex HIV-1 RNA repeat test will be conducted on stored plasma samples, if available. If the repeat result is < 50 copies/mL, no further action is required. If the repeat result is ≥ 50 copies/mL participants will be asked to return to the clinic for a scheduled or unscheduled blood draw (2 to 3 weeks after the date of the original test that resulted in HIV-1 RNA VR) for confirmation of VR.

A plasma sample from either the first instance or the VR confirmation visit will be tested for HIV-1 capsid genotypic and phenotypic resistance. In addition, if VR is confirmed, a plasma sample from the VR confirmation visit (or from the next available visit) will be tested for HIV-1 PR, RT, and IN genotypic and phenotypic resistance.

6.7.3. Viremia at Last Visit

Participants with HIV-1 RNA ≥ 50 copies/mL at their last study visit (eg, not confirmable) will be analyzed for resistance (HIV-1 capsid, PR, RT, and IN resistance testing).

6.8. End of Study

The end of the study will be the last participant’s last observation (or visit).

6.9. Post Study Care

After the participant has completed/terminated their participation in the study, long-term care of the participant will remain the responsibility of their primary treating physician.

7. ADVERSE EVENTS AND TOXICITY MANAGEMENT

7.1. Definitions of Adverse Events and Serious Adverse Events

7.1.1. Adverse Events

An AE is any untoward medical occurrence in a clinical study participant administered an investigational product, which does not necessarily have a causal relationship with the treatment. An AE can therefore be any unfavorable and/or unintended sign, symptom, or disease temporally associated with the use of an investigational product, whether or not considered related to the investigational product. AEs may also include pre- or post-treatment complications that occur as a result of protocol specified procedures or special situations (Section 7.7). Preexisting events that increase in severity or change in nature during or as a consequence of participation in the clinical study will also be considered AEs.

An AE does not include the following:

- Medical or surgical procedures such as surgery, endoscopy, tooth extraction, and transfusion. The condition that led to the procedure may be an AE and must be reported.
- Preexisting diseases, conditions, or laboratory abnormalities present or detected before the screening visit that do not worsen
- Situations where an untoward medical occurrence has not occurred (eg, Hospitalization for elective surgery, social and/or convenience admissions)
- Overdose without clinical sequelae (Section 7.7.1)
- Any medical condition or clinically significant laboratory abnormality with an onset date before the consent form is signed and not related to a protocol-associated procedure is not an AE. It is considered to be preexisting and should be documented as medical history.

7.1.2. Serious Adverse Events

A SAE is defined as an event that, at any dose, results in the following:

- Death
- Life-threatening (Note: The term "life-threatening" in the definition of "serious" refers to an event in which the participant was at risk of death at the time of the event; it does not refer to an event that hypothetically might have caused death if it were more severe.)
- In-patient hospitalization or prolongation of existing hospitalization
- Persistent or significant disability/incapacity

- A congenital anomaly/birth defect

- A medically important event or reaction: such events may not be immediately life-threatening or result in death or hospitalization but may jeopardize the participant or may require intervention to prevent one of the other outcomes constituting SAEs. Medical and scientific judgment must be exercised to determine whether such an event is a reportable under expedited reporting rules. Examples of medically important events include intensive treatment in an emergency room or at home for allergic bronchospasm; blood dyscrasias or convulsions that do not result in hospitalization; and development of drug dependency or drug abuse

7.2. Assessment of Adverse Events and Serious Adverse Events

The investigator or qualified subinvestigator is responsible for assessing AEs and SAEs for causality and severity, and for final review and confirmation of accuracy of event information and assessments.

7.2.1. Assessment of Causality for Study Drugs and Procedures

The investigator or qualified subinvestigator is responsible for assessing the relationship to study drug using clinical judgment and the following considerations:

- **No**: Evidence exists that the AE has an etiology other than the study drug. For SAEs, an alternative causality must be provided (eg, preexisting condition, underlying disease, intercurrent illness, or concomitant medication).

- **Yes**: There is reasonable possibility that the event may have been caused by the study drug.

It should be emphasized that ineffective treatment should not be considered as causally related in the context of AE reporting.

The relationship to study procedures (eg, invasive procedures such as venipuncture or biopsy) should be assessed using the following considerations:

- **No:** Evidence exists that the AE has an etiology other than the study procedure.

- **Yes:** The AE occurred as a result of protocol procedures, (eg, venipuncture)

7.2.2. Assessment of Severity

The severity of AEs will be graded using the Division of AIDS (DAIDS) Table for Grading the Severity of Adult and Pediatric Adverse Events, Version 2.1 dated July 2017. For each episode, the highest grade attained should be reported as defined in the grading scale.

The DAIDS scale is available at the following location:
https://rsc.niaid.nih.gov/sites/default/files/daidsgradingcorrectedv21.pdf

7.3. Investigator Requirements and Instructions for Reporting Adverse Events and Serious Adverse Events

Requirements for Collection Prior to Study Drug Initiation:

After informed consent, but prior to initiation of study medication, the following types of events must be reported on the applicable electronic case report forms (eCRFs): all SAEs and AEs related to protocol-mandated procedures.

7.3.1. Adverse Events

Following initiation of study medication, all AEs, regardless of cause or relationship, throughout the duration of the study, including the protocol-defined follow-up visit, must be reported on the eCRFs as instructed.

All AEs should be followed up until resolution or until the AE is stable, if possible. Gilead may request that certain AEs be followed beyond the protocol-defined follow-up period.

7.3.2. Serious Adverse Events

All SAEs, regardless of cause or relationship, that occurs after the participant first consents to participate in the study (ie, signing the informed consent) and throughout the duration of the study, including the protocol-defined follow-up visit, must be reported on the applicable eCRFs and submitted to Gilead Patient Safety (formerly known as Pharmacovigilance and Epidemiology [PVE]) as instructed below in this section. This also includes any SAEs resulting from protocol-associated procedures performed after informed consent is signed.

Investigators are not obligated to actively seek SAEs after the protocol-defined follow-up period. However, if the investigator learns of any SAEs that occur after the protocol-defined follow-up period has concluded and the event is deemed relevant to the use of study drug, the investigator should promptly document and report the event to Gilead Patient Safety.

- All AEs and SAEs will be recorded in the eCRF database within the timelines outlined in the eCRF completion guidelines.

7.3.2.1. Electronic Serious Adverse Event (eSAE) Reporting Process

- Site personnel record all SAE data on the applicable eCRFs and from there transmit the SAE information to Gilead Patient Safety within 24 hours of the investigator's knowledge of the event. Detailed instructions can be found in the eCRF completion guidelines.

- If it is not possible to record and submit the SAE information electronically, because the eCRF database cannot be accessed or is not available (including at study start), record the SAE on the paper serious adverse event reporting form and submit within 24 hours to:

  Gilead Patient Safety: Gilead Patient Safety
  Email: Safety_FC@gilead.com or
  Fax: +1-650-522-5477

- As soon as it is possible to do so, any SAE reported via paper must be transcribed on the applicable eCRFs according to instructions and within the timelines outlined in the eCRF completion guidelines.

- If an SAE has been reported via a paper form because the eCRF database has been locked, no further action is necessary.

- For fatal or life-threatening events, copies of hospital case reports, autopsy reports, and other documents are also to be submitted by email or fax when requested and applicable. Transmission of such documents should occur without personal participant identification, maintaining the traceability of a document to the participant identifiers.

- Additional information may be requested to ensure the timely completion of accurate safety reports.

- Any medications necessary for treatment of the SAE must be recorded onto the concomitant medication section of the participant's eCRF and the event description of the SAE form.

7.4. Gilead Reporting Requirements

Depending on relevant local legislation or regulations, including the applicable United States (US) FDA Code of Federal Regulations, the European Union (EU) Clinical Trials Directive (2001/20/EC) and relevant updates, and other country-specific legislation or regulations, Gilead may be required to expedite to worldwide regulatory agencies reports of SAEs, which may be in the form of line-listings, serious adverse drug reactions (SADRs), or suspected unexpected serious adverse reactions (SUSARs). In accordance with the EU Clinical Trials Directive (2001/20/EC), Gilead or a specified designee will notify worldwide regulatory agencies and the relevant IEC in concerned Member States of applicable SUSARs as outlined in current regulations.

Assessment of expectedness for SAEs will be determined by Gilead using reference safety information specified in the investigator's brochure or relevant local label as applicable.

All investigators will receive a safety letter notifying them of relevant SUSAR reports associated with any study drug. The investigator should notify the IRB or IEC of SUSAR reports as soon as is practical, where this is required by local regulatory agencies, and in accordance with the local institutional policy.

7.5. Clinical Laboratory Abnormalities and Other Abnormal Assessments as Adverse Events or Serious Adverse Events

Laboratory abnormalities without clinical significance are not recorded as AEs or SAEs. However, laboratory abnormalities (eg, clinical chemistry, hematology, and urinalysis) that require medical or surgical intervention or lead to study drug interruption, modification, or discontinuation must be recorded as an AE, as well as an SAE, if applicable. In addition, laboratory or other abnormal assessments (eg, ECG, x-rays, vital signs) that are associated with

signs and/or symptoms must be recorded as an AE or SAE if they meet the definition of an AE or SAE as described in Sections 7.1.1 and 7.1.2. If the laboratory abnormality is part of a syndrome, record the syndrome or diagnosis (eg, anemia), not the laboratory result (eg, decreased hemoglobin).

Severity should be recorded and graded according to the DAIDS Table for Grading the Severity of Adult and Pediatric Adverse Events, Version 2.1 dated July 2017. For AEs associated with laboratory abnormalities, the event should be graded on the basis of the clinical severity in the context of the underlying conditions; this may or may not be in agreement with the grading of the laboratory abnormality

7.6. Toxicity Management

All clinical and clinically significant laboratory toxicities will be managed according to uniform guidelines detailed in Appendix 3 and as outlined below.

Grade 3 and 4 clinically significant laboratory abnormalities should be confirmed by repeat testing within 3 calendar days of receipt of results and before investigational medicinal product discontinuation, unless such a delay is not consistent with good medical practice. If repeat testing is not possible within 3 calendar days of receipt of results, it may be completed within 14 calendar days of receipt of results per the investigator’s discretion.

The Gilead Medical Monitor should be consulted prior to study drug discontinuation when medically feasible. Before discontinuation of study drug for AEs or laboratory abnormalities, an assessment of the participants’ medical situation including the potential for an alternative ARV regimen and the risk of resistance with subtherapeutic GS-6207 exposure should be made. Although routinely, participants with ≥ Grade 3 AEs or laboratory abnormalities that are considered related to the study drug should be discontinued, if alternative HIV therapies are not available, the participant may continue dosing with approval of the medical monitor following discussion with the investigator.

7.6.1. Grades 1 and 2 Laboratory Abnormality or Clinical Event

Continue study drug at the discretion of the investigator.

7.6.2. Grade 3 Laboratory Abnormality or Clinical Event

For a Grade 3 clinically significant laboratory abnormality or clinical event, study drug may be continued if the event is considered to be unrelated to study drug.

For a Grade 3 clinically significant laboratory abnormality or clinical event confirmed by repeat testing, that is considered to be related to study drug, consider withholding study drug until the toxicity returns to ≤ Grade 2.

If a clinically significant laboratory abnormality or clinical event recurs to ≥ Grade 3 following re-challenge with study drug and is considered to be related to study drug, consider permanent discontinuation of study drug. The participant should be managed according to local practice.

Recurrence of laboratory abnormalities considered unrelated to study drug may not require permanent discontinuation but requires discussion with the Gilead Medical Monitor.

7.6.3. Grade 4 Laboratory Abnormality or Clinical Event

For a Grade 4 clinically significant laboratory abnormality or clinical event confirmed by repeat testing, that is considered to be related to study drug, consider permanent discontinuation of study drug. The participant should be managed according to local practice. The participant should be followed as clinically indicated until the laboratory abnormality returns to baseline or is otherwise explained, whichever occurs first. A clinically significant Grade 4 laboratory abnormality that is not confirmed by repeat testing should be managed according to the algorithm for the new toxicity grade.

Study drug may be continued without dose interruption for a clinically non-significant Grade 4 laboratory abnormality (eg, Grade 4 CK elevation after strenuous exercise or triglyceride elevation that is nonfasting or that can be medically managed) or a clinical event considered unrelated to study drug.

Treatment-emergent toxicities will be noted by the investigator and brought to the attention of the Gilead Medical Monitor, and the appropriate course of action will be discussed and decided. Whether or not considered treatment-related, all participants experiencing AEs must be monitored periodically until symptoms subside, any abnormal laboratory values have resolved or returned to baseline levels or they are considered irreversible, or until there is a satisfactory explanation for the changes observed.

Any questions regarding toxicity management should be directed to the Gilead Medical Monitor.

7.6.4. Management of Adverse Events of Injection Site Reactions of Grade 3 or Higher, or Persisting for More Than 26 Weeks

In clinical studies of SC GS-6207, Grade 3 or higher AEs of injections site reactions (ISRs) were uncommon. Some participants experienced AEs of injection site nodule and induration, which decreased in size over 6 months or longer. For AEs of ISRs related to SC GS-6207 that are Grade 3 or higher, or persisting for more than 26 weeks, particularly nodule and/or induration, the investigator must contact the Medical Monitor and obtain consultation with an independent dermatologist to evaluate the ISRs.

If clinically indicated as determined by the independent dermatologist, a biopsy of the SC injection site may be performed. The histopathology should be reviewed by an independent dermatopathologist; if a dermatopathologist is not locally available, the histopathology may be reviewed by an otherwise medically qualified person.

Photographic documentation of the ISRs that meet the criteria above is recommended, if possible, but is not mandatory. If obtained, the documentation may be shared by the investigator with the independent dermatologist or dermatopathologist and may be requested by the sponsor.

7.7. Special Situations Reports

7.7.1. Definitions of Special Situations

Special situation reports include all reports of medication error, abuse, misuse, overdose, occupational exposure with AE, AE in an infant following exposure via breastfeeding, product complaints with AE, and pregnancy regardless of an associated AE.

Medication error is any unintentional error in the prescribing, dispensing, preparation for administration or administration of an investigational product while the medication is in the control of a health care professional, patient, or consumer. Medication errors may be classified as a medication error without an AE, which includes situations of missed dose; medication error with an AE; intercepted medication error; or potential medication error.

Abuse is defined as persistent or sporadic intentional excessive use of an investigational product by a participant.

Misuse is defined as any intentional and inappropriate use of an investigational product that is not in accordance with the protocol instructions or the local prescribing information.

An overdose is defined as an accidental or intentional administration of a quantity of an investigational product given per administration or cumulatively which is above the maximum recommended dose as per protocol or in the product labelling (as it applies to the daily dose of the participant in question). In cases of a discrepancy in drug accountability, overdose will be established only when it is clear that the participant has taken the excess dose(s). Overdose cannot be established when the participant cannot account for the discrepancy except in cases in which the investigator has reason to suspect that the participant has taken the additional dose(s).

Occupational exposure is defined as exposure to an investigational product as a result of one's professional or non-professional occupation.

Product complaint is defined as complaints arising from potential deviations in the manufacture, packaging, or distribution of the investigational product.

7.7.2. Instructions for Reporting Special Situations

7.7.2.1. Instructions for Reporting Pregnancies

The investigator should report pregnancies in female study participants and female partners of male study participants that are identified after initiation of study drug and throughout the study, including the protocol-defined follow-up period, to Gilead Patient Safety using the pregnancy report form within 24 hours of becoming aware of the pregnancy. Refer to Appendix 4 for further information regarding protocol-defined follow-up period.

If the investigator learns of any pregnancy or pregnancy outcomes that occur after the protocol-defined follow-up period has concluded but within 700 days following the last dose of SC GS-6207, or within 60 days of last oral dose of GS-6207, the investigator should promptly document and report the event to Gilead Patient Safety.

Refer to Section 7.3 and the eCRF completion guidelines for full instructions on the mechanism of pregnancy reporting.

The pregnancy itself is not considered an AE nor is an induced elective abortion to terminate a pregnancy without medical reasons.

Any premature termination of pregnancy (eg, a spontaneous abortion, an induced therapeutic abortion due to complications or other medical reasons) must be reported within 24 hours as an SAE. The underlying medical reason for this procedure should be recorded as the AE term.

A spontaneous abortion is always considered to be an SAE and will be reported as described in Section 7.3. Furthermore, any SAE occurring as an adverse pregnancy outcome post study must be reported to Gilead Patient Safety.

The participant should receive appropriate monitoring and care until the conclusion of the pregnancy. The outcome should be reported to Gilead Patient Safety using the pregnancy outcome report form. If the pregnancy/outcome occurs after the study has been completed, including the protocol-defined follow-up period, but within 700 days of the last dose of SC GS-6207, or within 60 days of last oral dose of GS-6207, the pregnancy/outcome should be reported directly to Gilead Patient Safety. Gilead Patient Safety contact information is as follows: email: Safety_FC@gilead.com and fax: +1-650-522-5477.

Pregnancies of female partners of male study participants exposed to Gilead or other study drugs during the study, including the protocol-defined follow-up period, must also be reported and relevant information should be submitted to Gilead Patient Safety using the pregnancy and pregnancy outcome forms within 24 hours. If the end of pregnancy occurs after the study has been completed, the outcome should be reported directly to Gilead Patient Safety, fax number +1-650-522-5477 or email Safety_FC@gilead.com.

Refer to Appendix 4. for Pregnancy Precautions, Definition for Women of Childbearing Potential, and Contraceptive Requirements.

7.7.2.2. Reporting Other Special Situations

All other special situation reports must be reported on the special situations report form and forwarded to Gilead Patient Safety within 24 hours of the investigator becoming aware of the situation. These reports must consist of situations that involve study drug and/or Gilead concomitant medications, but do not apply to non-Gilead concomitant medications.

Special situations involving non-Gilead concomitant medications do not need to be reported on the special situations report form; however, special situations that result in AEs due to a non-Gilead concomitant medication, must be reported as an AE.

Any inappropriate use of concomitant medications prohibited by this protocol should not be reported as "misuse," but may be more appropriately documented as a protocol deviation.

Refer to Section 7.3 and the eCRF completion guidelines for instructions on special situation reporting.

All clinical sequelae in relation to these special situation reports will be reported as AEs or SAEs at the same time using the AE eCRF and/or the SAE report form. Details of the symptoms and signs, clinical management, and outcome will be reported, when available.

7.8. Safety Monitoring Committee

Gilead teams periodically review accumulating safety data across completed and ongoing studies within the GS-6207 development program to detect unexpected suspected adverse reactions and clinically important increased rates of AEs, laboratory abnormalities, and previously recognized adverse reactions. If, during the periodic review of safety data from clinical studies, a significant numerical imbalance is observed for an AE, laboratory abnormality, and previously recognized adverse reaction across treatment groups based on predefined reporting thresholds, a safety monitoring committee will request review of unblinded safety data and, in collaboration with other safety committees (in line with Gilead's signal management process and unblinding process), determine if any actions are necessary to protect participants involved in the GS-6207 development program. If appropriate and available, the safety monitoring committee would be a data monitoring committee (DMC). If an appropriate DMC is not available, an internal Safety Assessment Committee (SAC), comprised of Gilead employees not involved in the conduct of the study, reviews the data. The membership, responsibilities, conduct, specific activities, and meeting schedule of the unblinded internal SAC will be described in a charter. The blind will be maintained for persons responsible for the ongoing conduct of the study and those responsible for data analysis and interpretation of results at the conclusion of the study.

8. STATISTICAL CONSIDERATIONS

8.1. Analysis Objectives and Endpoints

8.1.1. Analysis Objectives

The primary objective of this study is:

- To evaluate the antiviral activity of GS-6207 administered as an add-on to a failing regimen (functional monotherapy) for PLWH with MDR as determined by the proportion of participants achieving at least 0.5 $\log_{10}$ reduction from baseline in HIV-1 RNA at the end of the Functional Monotherapy Period

The secondary objectives of this study are:

- To evaluate the safety and efficacy of GS-6207 in combination with an optimized background regimen at Weeks 26 and 52
- To evaluate the safety and efficacy of GS-6207 in combination with an OBR at Weeks 104 and 156 (from the first SC dose of GS-6207)

CCI [redacted]

8.1.2. Primary Endpoint

The proportion of participants in Cohort 1 achieving $\geq$ 0.5 $\log_{10}$ copies/mL reduction from baseline in HIV-1 RNA at the end of Functional Monotherapy Period.

8.1.3. Secondary Endpoints

The secondary endpoints of this study are:

- The proportion of participants in Cohort 1 with plasma HIV-1 RNA < 50 copies/mL and < 200 copies/mL at Weeks 26 and 52 visits based on the US FDA-defined snapshot algorithm.
- The proportion of participants in combined Cohorts 1 and 2 with plasma HIV-1 RNA < 50 copies/mL and < 200 copies/mL at Weeks 104 and 156 from the first SC dose of GS-6207 based on the US FDA-defined snapshot algorithm.

8.2. Planned Analyses

8.2.1. Interim Analysis

Before the final analysis, interim analyses may be conducted, and the analyses may be submitted to regulatory agencies to seek guidance for the overall clinical development program.

8.2.1.1. Dose Escalation Analysis

Not applicable.

8.2.1.2. Planned Internal Analysis

Interim analyses after the primary analysis (see Section 8.2.2) will be performed after all participants in Cohort 1 have completed their Week 52 visit and/or after all participants in Cohort 2 have completed their Week 26 visit or have prematurely discontinued the study drug, outstanding data queries have been resolved or adjudicated as unresolvable, and the data have been cleaned and finalized.

8.2.1.3. DMC Analysis

There will be one planned DMC analysis of efficacy and safety. The DMC will convene after all participants in Cohort 1 have completed 14 days of assessment or discontinued the study drug in the Functional Monotherapy Period. Given that all participants have completed the Functional Monotherapy Period, treatment assignment will be unblinded for the DMC analysis.

No formal stopping rules will be used by the DMC for safety outcomes. Rather, a clinical assessment will be made to determine if the nature, frequency, and severity of AEs associated with a study regimen warrant the early termination of the study in the best interest of the participants.

Gilead does not have a prior intent to ask the DMC to consider early termination of the study even if there is an early evidence of favorable efficacy. However, Gilead will stop further enrollment if 50% or more of the participants in the GS-6207 group fail in Cohort 1 to achieve at least 0.5 $\log_{10}$ reduction from baseline in HIV-1 RNA at the end of the Functional Monotherapy Period. The decision whether to continue with the study and the development of GS-6207 will be based on the magnitude of the HIV-1 RNA decline at the end of the Functional Monotherapy Period.

8.2.2. Primary Analysis

The primary analysis of the primary endpoint will be conducted after all participants in Cohort 1 have completed the Week 26 visit or have prematurely discontinued the study drug, outstanding data queries have been resolved or adjudicated as unresolvable, and the data have been cleaned and finalized for the analysis. This analysis of the primary endpoint will serve as the final analysis for this endpoint. The data from this analysis will be used to support the GS-6207 regulatory filing for the indication in the heavily treatment experienced PLWH.

8.2.3. Year 2 Analysis

The Year 2 Analysis will be performed after all participants in both cohorts have had HIV-1 RNA data in the Week 104 analysis window (or reached the upper limit of the analysis window with missing HIV-1 RNA data), or have prematurely discontinued the study drug, outstanding data queries have been resolved or adjudicated as unresolvable, and the data have been cleaned and finalized.

8.2.4. Year 3 Analysis

The Year 3 Analysis will be performed after all participants in both cohorts have had HIV-1 RNA data in the Week 156 analysis window (or reached the upper limit of the analysis window with missing HIV-1 RNA data), or have prematurely discontinued the study drug, outstanding data queries have been resolved or adjudicated as unresolvable, and the data have been cleaned and finalized.

8.2.5. Final Analysis

The final analysis will be performed after all participants have completed the study, outstanding data queries have been resolved or adjudicated as unresolvable, and the data have been cleaned and finalized.

8.3. Analysis Conventions

8.3.1. Analysis Sets

8.3.1.1. Efficacy

The primary analysis set for efficacy analysis is defined as full analysis set (FAS). Two FASs are defined for this study: one for the primary efficacy endpoint (referred to as “FAS for the Functional Monotherapy Period analysis”) and the other for secondary efficacy endpoints (referred to as “FAS for the All GS-6207 analysis”). The FAS for the Functional Monotherapy Period includes all participants who are randomized and receive at least one dose of blinded study drug. In this analysis, participants will be grouped according to the treatment to which they are randomized. The FAS for the All GS-6207 analysis includes all participants who receive at least one dose of GS-6207.

8.3.1.2. Safety

The primary analysis set for safety analyses is defined as safety analysis set, which includes all participants who are randomized/enrolled and receive any dose of study drug. Participants who receive treatment other than that intended will be analyzed according to treatment received. All data collected during treatment will be included in the safety summaries.

8.3.1.3. Pharmacokinetics

The primary analysis set for PK analyses is defined for GS-6207, which includes all participants who are randomized/enrolled, receive any dose of study drug, and have at least 1 non-missing postbaseline concentration value for GS-6207.

8.3.2. Data Handling Conventions

Logarithm (base 10) transformation will be applied to HIV-1 RNA levels for efficacy analysis. Natural logarithm transformation for all PK parameters of GS-6207 will be applied for pharmacokinetic analysis.

For summary statistics, PK concentration values below the limit of quantitation (BLQ) will be treated as zero at predose and one-half of the lower limit of quantitation (LLOQ) for post dose time points.

Laboratory data that are continuous in nature but are less than the LLOQ or above the upper limit of quantitation will be imputed to the value of the lower or upper limit minus or plus 1 significant digit, respectively (eg, if the result of a continuous laboratory test is < 20, a value of 19 will be assigned; if the result of a continuous laboratory test is < 20.0, a value of 19.9 will be assigned).

8.4. Demographic and Baseline Characteristics Analysis

Demographic and baseline measurements will be summarized using standard descriptive methods by cohort and treatment.

Demographic summaries will include sex, race, ethnicity, age, sexual orientation, and gender identity.

Baseline data will include a summary of body weight, height, and body mass index.

For Cohort 1, the Cochran-Mantel-Haenszel (CMH) test will be used to compare the 2 treatment groups for categorical data, and the 2-sided Wilcoxon rank sum test will be used to compare the treatment groups for continuous data. No statistical comparisons will be made for Cohort 2.

8.5. Efficacy Analysis

8.5.1. Primary Analysis

The primary efficacy endpoint is the proportion of participants in Cohort 1 achieving $\geq 0.5 \log_{10}$ reduction from baseline in HIV-1 RNA at the end of the Functional Monotherapy Period. The primary analysis of the efficacy endpoint will be based on the FAS for the Functional Monotherapy Period.

The null hypothesis is that there is no difference in the proportion of participants achieving $\geq 0.5\ \log_{10}$ reduction from baseline at the end of the Functional Monotherapy Period (between the GS-6207 group and the Placebo group in Cohort 1); the alternative hypothesis is that there is a difference (GS-6207 – Placebo) in the proportion of participants achieving $\geq 0.5\ \log_{10}$ reduction from baseline at the end of the Functional Monotherapy Period between the two treatment groups in Cohort 1. For participants with missing HIV-1 RNA values at the end of the Functional Monotherapy Period the value will be imputed using the last observation carried forward method. The difference in proportions between two treatment groups will be compared using an unconditional exact method using 2 invert 1-sided tests (Chan and Zhang 1999) with an alpha level at 0.05 to evaluate superiority. The p-value and 95% confidence interval for the point estimate of treatment difference in proportions will be estimated and constructed using the above-mentioned method.

8.5.2. Secondary Analyses

The proportion of participants in Cohort 1 with HIV-1 RNA < 50 copies/mL at Week 26 will be summarized using the US FDA-defined snapshot algorithm.

The analysis window at Week 26 is defined as from Study Day 184 to Study Day 232, inclusive, where Study Day is calculated from the first dose of GS-6207. Virologic outcome will be defined as the following categories:

- **HIV-1 RNA < 50 copies/mL:** this includes participants who have the last available on-treatment HIV-1 RNA < 50 copies/mL in the Week 26 analysis window
- **HIV-1 RNA ≥ 50 copies/mL:** this includes participants
  - Who have the last available on-treatment HIV-1 RNA ≥ 50 copies/mL in the Week 26 analysis window, or
  - Who do not have on-treatment HIV-1 RNA data in the Week 26 analysis window and
    - Who discontinue study drug prior to or in the Week 26 analysis window due to lack of efficacy, or
    - Who discontinue study drug prior to or in the Week 26 analysis window due to reasons other than AE, death, or lack of efficacy and have the last available on-treatment HIV-1 RNA ≥ 50 copies/mL
- **No Virologic Data in the Week 26 analysis window:** this includes participants who do not have on-treatment HIV-1 RNA data in the Week 26 analysis window because of the following:
  - Discontinuation of study drug prior to or in the Week 26 analysis window due to AE or death (regardless of whether the last available on-treatment HIV-1 RNA < 50 copies/mL or not) or,

- Discontinuation of study drug prior to or in the Week 26 analysis window due to reasons other than AE, death, or lack of efficacy and the last available on-treatment HIV-1 RNA < 50 copies/mL or,
- Missing data during the window but on study drug.

The proportion of participants in Cohort 1 with HIV-1 RNA < 200 copies/mL at Week 26 will also be summarized using the US FDA-defined snapshot algorithm.

In addition, the proportion of participants in Cohort 1 with HIV-1 RNA < 50 copies/mL and < 200 copies/mL at Week 52, and the proportion of participants in combined Cohorts 1 and 2 with HIV-1 RNA < 50 copies/mL and < 200 copies/mL at Weeks 104 and 156 (from the first SC dose of GS-6207) will also be summarized using the US FDA-defined snapshot algorithm. The analysis window at Week 52 is defined as from Study Day 324 to Study Day 414, inclusive. The analysis window at Week 104 is defined as from Study Day 688 to Study Day 778, inclusive. The analysis window at Week 156 is defined as from Study Day 1052 to Study Day 1142, inclusive. The secondary endpoints will be based on the FAS for the All GS-6207 analysis. Analysis will be based on participants who receive at least one dose of SC GS-6207.

8.6. Safety Analysis

All safety data collected on or after the date that study drug was first dispensed will be summarized by cohort and treatment (according to the study drug received). For participants who discontinue from the oral GS-6207, only data collected up to 60 days (approximately 5 times the GS-6207 oral dose half-life) after the last dose of the oral tablet will be included. All data collected will be included in data listings.

8.6.1. Extent of Exposure

A participant's extent of exposure to study drug data will be generated from the study drug administration data. Exposure data will be listed.

8.6.2. Adverse Events

Clinical and laboratory AEs will be coded using the Medical Dictionary for Regulatory Activities (MedDRA). System Organ Class (SOC), High-Level Group Term (HLGT), High-Level Term (HLT), Preferred Term (PT), and Lower-Level Term (LLT) will be attached to the clinical database.

Events will be summarized on the basis of the date of onset for the event. A treatment-emergent AE will be defined as any AE that begins on or after the date of first dose of study drug. For participants who discontinue from the oral GS-6207, only AEs collected up to 60 days (approximately 5 times the GS-6207 oral dose half-life) after the last dose date will be considered treatment emergent.

Summaries (number and percentage of participants) of treatment-emergent AEs (TEAEs) (by SOC, and PT) will be provided by treatment group.

8.6.3. Laboratory Evaluations

Selected laboratory data (using units) will be summarized using only observed data. Data and change from baseline at all scheduled time points will be summarized.

Graded laboratory abnormalities will be defined using the grading scheme in the Division of AIDS (DAIDS) Table for Grading the Severity of Adult and Pediatric Adverse Events, Version 2.1 dated July 2017.

Incidence of treatment-emergent laboratory abnormalities, defined as values that increase at least 1 toxicity grade from baseline at any time post baseline (and up to 60 days after the last dose date if participants discontinue from oral GS-6207), will be summarized by cohort and treatment group. If baseline data are missing, any graded abnormality (eg, at least a Grade 1) will be considered treatment emergent. The post baseline maximum toxicity grade will be summarized by laboratory parameter.

8.6.4. Other Safety Evaluations

Vital sign and safety ECG data will be summarized and/or listed as appropriate.

8.7. Adjustments for Multiplicity

The primary efficacy endpoint will be evaluated at the time when all participates in Cohort 1 have completed the Functional Monotherapy Period or discontinued the study drug. There will be no interim analyses before the analysis of the primary efficacy endpoint; therefore, no alpha level adjustment will be applied to the primary efficacy endpoint.

8.8. Pharmacokinetic Analysis

Plasma concentrations of GS-6207 will be summarized by nominal sampling time using descriptive statistics, as appropriate (eg, sample size, arithmetic mean, 90% confidence intervals, geometric mean, % coefficient of variation, standard deviation, median, minimum, and maximum). The concentrations of the study drug in plasma over time will be plotted in semi-logarithmic and linear formats as mean ± standard deviation and median (Q1, Q3), respectively.

8.9. Sample Size

A total of 36 participants in Cohort 1 will provide at least 90% power to detect a 60% difference in the proportion of participants achieving a $\geq 0.5 \log_{10}$ reduction from baseline at Day 15 of the Functional Monotherapy Period between the treatment groups (GS-6207 in Cohort 1A and placebo in Cohort 1B).

In this sample size and power computation, it is assumed that 70% and 10% of participants achieve a $\geq 0.5 \log_{10}$ reduction from baseline in HIV-1 RNA in the GS-6207 treatment group (Cohort 1A) and the placebo group (Cohort 1B), respectively (based on data from Trogarzo Phase 3 TMB-301 study {Emu 2018}), and the Fisher exact test is conducted at 2-sided significant level of 0.05.

A total sample size of 36 participants from Cohort 1A and 1B will provide reasonable assessment of safety through at least 24 weeks of GS-6207 treatment in heavily treatment experienced participants.

8.10. Data Monitoring Committee

An external multidisciplinary data monitoring committee (DMC) will review the progress of the study and perform interim reviews of efficacy and safety data, and provide recommendation to Gilead whether the nature, frequency, and severity of adverse effects associated with study treatment warrant the early termination of the study in the best interests of the participants, whether the study should continue as planned, or the study should continue with modifications.

The DMC's specific activities will be defined by a mutually agreed charter, which will define the DMC's membership, conduct and meeting schedule.

While the DMC will be asked to advise Gilead regarding future conduct of the study, including possible early study termination, Gilead retains final decision-making authority on all aspects of the study.

9. RESPONSIBILITIES

9.1. Investigator Responsibilities

9.1.1. Good Clinical Practice

The investigator will ensure that this study is conducted in accordance with ICH E6(R2) addendum to its guideline for GCP and applicable laws and regulations.

9.1.2. Good Postmarketing Study Practices (Japan Only)

The investigator will ensure that this study is conducted in accordance with GPSP after approval of GS-6207 in Japan and adherence to the basic principles of GCP.

9.1.3. Financial Disclosure

The investigator and subinvestigators will provide prompt and accurate documentation of their financial interest or arrangements with Gilead, or proprietary interests in the investigational drug during the course of a clinical study. This documentation must be provided prior to the investigator's (and any subinvestigator's) participation in the study. The investigator and subinvestigator agree to notify Gilead of any change in reportable interests during the study and for 1 year following completion of the study. Study completion is defined as the date when the last participant completes the protocol-defined activities.

9.1.4. Institutional Review Board/Independent Ethics Committee Review and Approval

The investigator (or Gilead as appropriate according to local regulations) will submit this protocol, informed consent form, and any accompanying material to be provided to the participant (such as advertisements, participant information sheets, or descriptions of the study used to obtain informed consent) to an IRB/IEC. The investigator will not begin any study participant activities until approval from the IRB/IEC has been documented and provided as a letter to the investigator.

Before implementation, the investigator will submit to and receive documented approval from the IRB/IEC any modifications made to the protocol or any accompanying material to be provided to the participant after initial IRB/IEC approval, with the exception of those necessary to reduce immediate risk to study participants.

9.1.5. Informed Consent

The investigator is responsible for obtaining written informed consent from each participant after adequate explanation of the aims, methods, objectives, and potential hazards of the study before undertaking any study-related procedures. The investigator must use the most current IRB- or IEC-approved consent form for documenting written informed consent. Each informed consent

(or assent as applicable) will be appropriately signed and dated by the participant or the participant's legally authorized representative and the person conducting the consent discussion, and also by an impartial witness if required by IRB or IEC or local requirements.

A consent form will inform participants about pharmacogenomic testing and/or planned sample retention. CCI [redacted] The results of the tests done on the samples will not be given to the participant or the investigator.

9.1.6. Confidentiality

The investigator must assure that participants' anonymity will be strictly maintained and that their identities are protected from unauthorized parties. Only an identification code and any other unique identifier(s) as allowed by local law (such as year of birth) will be recorded on any form or biological sample submitted to Gilead, or laboratory. Laboratory specimens must be labeled in such a way as to protect participant identity while allowing the results to be recorded to the proper participant. Refer to specific laboratory instructions. NOTE: The investigator must keep a screening log with details for all participants screened and enrolled in the study, in accordance with the site procedures and regulations. Participant data will be processed in accordance with all applicable regulations.

The investigator agrees that all information received from Gilead, including but not limited to the investigator's brochure, this protocol, eCRF, the study drug, and any other study information, remain the sole and exclusive property of Gilead during the conduct of the study and thereafter. This information is not to be disclosed to any third party (except employees or agents directly involved in the conduct of the study or as required by law) without prior written consent from Gilead. The investigator further agrees to take all reasonable precautions to prevent the disclosure by any employee or agent of the study site to any third party or otherwise into the public domain.

9.1.7. Study Files and Retention of Records

The investigator must maintain adequate and accurate records to enable the conduct of the study to be fully documented and the study data to be subsequently verified. These documents should be classified into at least the following 2 categories: (1) investigator's study file, and (2) participant clinical source documents.

The investigator's study file will contain the protocol/amendments, eCRFs, and governmental approval with correspondence, informed consent, drug records, staff curriculum vitae and authorization forms, and other appropriate documents and correspondence.

The required source data should include sequential notes containing at least the following information for each participant:

- Participant identification;
- Documentation that participant meets eligibility criteria, ie, medical history, physical examination, and confirmation of diagnosis (to support inclusion and exclusion criteria);
- Documentation of the reason(s) a consented participant is not enrolled
- Participation in study (including study number);
- Study discussed and date of informed consent;
- Dates of all visits;
- Documentation that protocol-specific procedures were performed;
- Results of efficacy parameters, as required by the protocol;
- Start and end date (including dose regimen) of study drug, including dates of dispensing and return;
- Record of all AEs and other safety parameters (start and end date, and including causality and severity), and documentation that adequate medical care has been provided for any AE
- Concomitant medication (including start and end date, dose if relevant; dose changes);
- Date of study completion and reason for early discontinuation if it occurs.

All clinical study documents must be retained by the investigator until at least 2 years or according to local laws, whichever is longer, after the last approval of a marketing application in an ICH region (eg, United States, Europe, or Japan) and until there are no pending or planned marketing applications in an ICH region; or, if no application is filed or if the application is not approved for such indication, until 2 years after the investigation is discontinued and regulatory authorities have been notified. Investigators may be required to retain documents longer if specified by regulatory requirements, by local regulations, or by an agreement with Gilead. The investigator must notify Gilead before destroying any clinical study records.

Should the investigator wish to assign the study records to another party or move them to another location, Gilead must be notified in advance.

If the investigator cannot provide for this archiving requirement at the study site for any or all of the documents, special arrangements must be made between the investigator and Gilead to store these records securely away from the site so that they can be returned sealed to the investigator in case of an inspection. When source documents are required for the continued care of the participant, appropriate copies should be made for storage away from the site.

9.1.8. Case Report Forms

For each participant consented, an eCRF casebook will be completed by an authorized study staff member whose training for this function is completed in the EDC system. The eCRF casebook will only capture the data required per the protocol schedule of events and procedures. The Inclusion/Exclusion Criteria and Enrollment eCRFs should be completed only after all data related to eligibility have been received. Data entry should be performed in accordance with the eCRF Completion Guidelines (CCGs) provided by the Sponsor. After data entry, a study monitor will perform source data verification (SDV) within the EDC system. System-generated or manual queries will be issued in the EDC system as data discrepancies are identified by the monitor or Gilead staff, who routinely review the data for completeness, correctness, and consistency. The site investigator or site coordinator or other designee is responsible for responding to the queries in a timely manner, within the system, either by confirming the data as correct or updating the original entry, and providing the reason for the update (eg, data entry error). Original entries as well as any changes to data fields will be stored in the audit trail of the system. At a minimum, prior to any interim time points or database lock (as instructed by Gilead), the investigator will use his/her log in credentials to confirm that the forms have been reviewed, and that the entries accurately reflect the information in the source documents. At the conclusion of the study, Gilead will provide the site investigator with a read-only archive copy of the data entered by that site. This archive must be stored in accordance with the records retention requirements outlined in Section 9.1.7.

9.1.9. Investigator Inspections

The investigator will make available all source documents and other records for this study to Gilead’s appointed study monitors, to IRBs/IECs, or to regulatory authority or health authority inspectors.

9.1.10. Protocol Compliance

The investigator is responsible for ensuring the study is conducted in accordance with the procedures and evaluations described in this protocol.

9.2. Sponsor Responsibilities

9.2.1. Protocol Modifications

Protocol modifications, except those intended to reduce immediate risk to study participants, may be made only by Gilead. The investigator must submit all protocol modifications to the in accordance with local requirements and receive documented approval before modifications can be implemented.

9.2.2. Study Reports and Publications

A CSR will be prepared and provided to the regulatory agency(ies) when applicable and in accordance with local regulatory requirements. Gilead will ensure that the report meets the standards set out in the ICH Guideline for Structure and Content of Clinical Study Reports (ICH E3). Note that an abbreviated report may be prepared in certain cases. For studies with sites in countries following the Directive 2001/20/EC, a CSR will be submitted within 1 year (6 months for pediatric studies, in accordance with Regulation [EC] No. 1901/2006) after the global end of study (as defined in Section 6.8).

Investigators in this study may communicate, orally present, or publish in scientific journals or other scholarly media in accordance with the Gilead clinical trial agreement.

9.3. Joint Investigator/Sponsor Responsibilities

9.3.1. Payment Reporting

Investigators and their study staff may be asked to provide services performed under this protocol, eg, attendance at investigator meetings. If required under the applicable statutory and regulatory requirements, Gilead will capture and disclose to Federal and State agencies any expenses paid or reimbursed for such services, including any clinical study payments, meal, travel expenses or reimbursements, consulting fees, and any other transfer of value.

9.3.2. Access to Information for Monitoring

The monitor is responsible for routine review of the eCRF at regular intervals throughout the study to verify adherence to the protocol and the completeness, consistency, and accuracy of the data being entered on them. The monitor should have access to any participant records needed to verify the entries in the eCRF. The investigator agrees to cooperate with the monitor to ensure that any problems detected through any type of monitoring (central, on site) are resolved.

9.3.3. Access to Information for Auditing or Inspections

Representatives of regulatory authorities or of Gilead may conduct inspections or audits of the clinical study. If the investigator is notified of an inspection by a regulatory authority the investigator agrees to notify the Gilead Medical Monitor immediately. The investigator agrees to provide to representatives of a regulatory agency or Gilead access to records, facilities, and personnel for the effective conduct of any inspection or audit.

9.3.4. Study Discontinuation

Both Gilead and the investigator reserve the right to terminate the study at any time. Should this be necessary, both parties will arrange discontinuation procedures and notify the participants, appropriate regulatory authorities, IRBs, and IECs. In terminating the study, Gilead and the investigator will assure that adequate consideration is given to the protection of the participants' interests.

10. REFERENCES

Claborn KR, Meier E, Miller MB, Leffingwell TR. A Systematic Review Of Treatment Fatigue Among HIV-Infected Patients Prescribed Antiretroviral Therapy. Psychology, health & medicine 2015;20 (3):1-11.

Cockcroft DW, Gault MH. Prediction of creatinine clearance from serum creatinine. Nephron 1976;16:31-41.

Department of Health and Human Services (DHHS). Guidelines for the Use of Antiretroviral Agents in HIV-1-Infected Adults and Adolescents. Developed by the HHS Panel on Antiretroviral Guidelines for Adults and Adolescents – A Working Group of the Office of AIDS Research Advisory Council (OARAC). Revised 12 February 2013. Available at: http://aidsinfo.nih.gov/guidelines. Accessed: 2013 February 12. 2013.

Emu B, Fessel J, Schrader S, Kumar P, Richmond G, Win S, et al. Phase 3 Study of Ibalizumab for Multidrug-Resistant HIV-1. N Engl J Med 2018;379 (7):645-54.

Lundgren JD, Clumeck N, Rockstroh J, Ryom L. European AIDS Clinical Society (EACS) Guidelines. Version 7.0 October. 2013:

Thompson MA, Aberg JA, Cahn P, Montaner JS, Rizzardini G, Telenti A, et al. Antiretroviral treatment of adult HIV infection: 2010 recommendations of the International AIDS Society-USA panel. JAMA 2010;304 (3):321-33.

U. S. Department of Health and Human Services, Food and Drug Administration (FDA), Center for Drug Evaluation and Research (CDER). Human Immunodeficiency Virus-1 Infection: Developing Antiretroviral Drugs for Treatment. Guidance for Industry. Silver Spring, MD. November, 2015.

UNAIDS. Fact Sheet 2022. Global HIV statistics. 2022:

Williams I, Churchill D, Anderson J, Boffito M, Bower M, Cairns G, et al. British HIV Association guidelines for the treatment of HIV-1-positive adults with antiretroviral therapy 2012 (Updated November 2013. All changed text is cast in yellow highlight.). HIV Med 2014;15 Suppl 1:1-85.

11. APPENDICES

Appendix 1. Investigator Signature Page
Appendix 2. Study Procedures Table
Appendix 3. Management of Clinical and Laboratory Adverse Events
Appendix 4. Pregnancy Precautions, Definition for Women of Childbearing Potential, and Contraceptive Requirements
Appendix 5. Pandemic Risk Assessment and Mitigation Plan
Appendix 6. Country-Specific Requirements
Appendix 7. Amendment History

Appendix 1. Investigator Signature Page

GILEAD SCIENCES, INC.
333 LAKESIDE DRIVE
FOSTER CITY, CA 94404

STUDY ACKNOWLEDGMENT

A Phase 2/3 Study to Evaluate the Safety and Efficacy of Long Acting Capsid Inhibitor GS-6207 in Combination With an Optimized Background Regimen in Heavily Treatment Experienced People Living With HIV-1 Infection With Multidrug Resistance

GS-US-200-4625, Amendment 4 18 January 2023

This protocol has been approved by Gilead Sciences, Inc. The following signature documents this approval.

PPD
Executive Director, Clinical Development

[See appended electronic signature]
Signature

[See appended electronic signature]
Date

INVESTIGATOR STATEMENT

I have read the protocol, including all appendices, and I agree that it contains all necessary details for me and my staff to conduct this study as described. I will conduct this study as outlined herein and will make a reasonable effort to complete the study within the time designated.

I will provide all study personnel under my supervision copies of the protocol and access to all information provided by Gilead Sciences, Inc. I will discuss this material with them to ensure that they are fully informed about the drugs and the study.

Principal Investigator Name (Printed)

Signature

Date

Site Number

Appendix 2. Study Procedures Table

	Screening[a]	Cohort Selection[b]	Day 1[c]	Day 2	Day 5	Day 8	Day 15[d]	Day 16[d]	Day 19[d]	Day 22[d]	Day 1 SC	Weeks 4, 10, 16, 22, 26, 36	Week 52 and Visits Thereafter[e]	Oral Bridging Visits[f]	30, 90, and 180 Day Follow-Up[g]	Early Termination[h]
Written Informed Consent/Assent/Parental Consent	X															
Medical History	X															
Demographic Information	X															
Complete Physical Examination	X		X				X				X	X[i]	X[i]			X
Symptom Directed Physical Examination				X		X		X		X		X[i]	X[i]	X	X	
Vital Signs[j](include weight)	X		X	X		X	X	X		X	X	X	X	X	X	X
12-lead ECG (supine)	X															
Height	X															
Hematology[k], Chemistry[l], Estimated GFR, Urinalysis[m], Urine Chemistry[m], CD4+ Cell Count	X		X			X	X			X	X	X	X	X	X	X
Urine Storage Sample			X	X		X	X	X		X	X	X	X	X		X
Serum Pregnancy Test[n]	X															
Serum FSH[o]	X															
Urine Pregnancy Test[n]			X			X	X			X	X	X	X	X	X	X
HBV, HCV Testing	X															
HIV-1 Genotyping/Phenotyping	X															

	Screening[a]	Cohort Selection[b]	Day 1[c]	Day 2	Day 5	Day 8	Day 15[d]	Day 16[d]	Day 19[d]	Day 22[d]	Day 1 SC	Weeks 4, 10, 16, 22, 26, 36	Week 52 and Visits Thereafter[e]	Oral Bridging Visits[f]	30, 90, and 180 Day Follow-Up[g]	Early Termination[h]
Plasma HIV-1 RNA	X	X	X	X		X	X	X		X	X	X	X	X	X	X
Plasma Storage Sample	X	X	X	X		X	X	X		X	X	X	X	X	X	X
Intensive PK Plasma Collection[p]			X				X									
Single Timed, Single Anytime and/or Predose PK Plasma Sample[q]				X	X	X		X	X	X	X	X[r,s]	X	X		
[redacted]																
Begin Optimized Background Regimen[v]			X				X				X					
SC GS-6207 Administration[w]											X	X	X			
Symptoms Distress Module, SF-36, EQ-5D-5L[x]			X									X	X			
Numeric Pain Scale[y]											X	X	X			
Injection Site Reaction Worksheet[z]											X	X	X			
Adverse Events/ Concomitant Meds	X	X	X	X	X	X	X	X	X	X	X	X	X	X	X	X

a Screening evaluations must be completed within 42 days prior to Day 1.
b Cohort Selection visit to be completed 14 to 30 days after the Screening visit until Cohort 1 is fully enrolled.
c Day 1 tests and procedures must be completed prior to study drug administration.
d Day 15, 16, 19, and 22 visits will be completed by Cohort 1B participants only.
e At the Week 52 visit, participants will be given an option to attend visits at Weeks 62, 78, 88, 104, 114, 130, and will continue to alternate between every 10 weeks and every 16 weeks. Participants will receive SC GS-6207 every 6 months (26 weeks) ± 2 weeks from last injection starting at Week 52 visit, while continuing their OBR, until the product becomes accessible to participants through an access program, is commercially available, or until Gilead Sciences elects to discontinue the study in the country.
f Only applicable to participants who require oral weekly bridging if an SC injection of GS-6207 cannot be administered for any reason within the protocol visit window.
g Participants may be required to return to the clinic for a 30-, 90-, and 180-Day Follow-Up visit after Early Termination visit as noted in Section 6.4.1.
h Early Termination visit to be completed, if participant decides to discontinue study drug prior to completing Week 52 visit or prior to study completion. Investigators should counsel participant regarding the importance of continuing a complete ARV therapy in accordance to standard of care, and refer participant to an appropriate HIV treatment facility.
i Complete physical examination to be completed at Weeks 26 and 52, symptom-directed physical examination to be completed at all other visits.
j Vital signs – blood pressure, pulse, respiration rate, and temperature, weight.
k Hematology: CBC with differential and platelet count.
l Chemistries: alkaline phosphatase, AST, ALT, GGT, total bilirubin, direct and indirect bilirubin, total protein, albumin, LDH, CPK, bicarbonate, BUN, calcium, chloride, creatinine, glucose, lipase, magnesium, phosphorus, potassium, sodium, uric acid (except at Cohort Selection, Days 2, 5, 16, and 19 visits).
m Urinalysis and Urine Chemistry: including color & clarity, specific gravity, pH, glucose, ketones, bilirubin, urobilinogen, blood, nitrite, leukocyte esterase and microscopic (if microscopic elements are seen), urine protein, albumin, creatinine, phosphate, calcium, magnesium and uric acid (except at Cohort Selection, Days 2, 5, 16, and 19 visits).
n All women will have a serum test performed at Screening. Urine pregnancy test will be performed at all subsequent visits for women of childbearing potential (except at Days 2, 5, 16 and 19). Positive urine pregnancy tests will be confirmed with a serum test.
o FSH test is required for women who are < 54 years old and have stopped menstruating for ≥ 12 months but do not have documentation of ovarian hormonal failure.
p Day 1 (for all participants) and Day 15 (for Cohort 1B participants only): 0 (predose, ≤ 30 min before dose), 1, 2, 4, 6, and 8 hours post dose.
q Days 2, 8, 16, and 22: A predose sample (within 30 minutes of dosing) and a single timed PK samples between 1 and 6 hours post dose. Days 5 and 19 (if the visits are completed): a single anytime PK sample.
r During the Maintenance Period, at all visits without SC GS-6207 injections, including the Oral Bridging visits, if applicable: A single anytime PK sample will be collected.
s During the Maintenance Period, at all visits with SC GS-6207 injections: A single predose (within 30 minutes of dosing) PK sample will be collected (while participants are continuing to receive study drug).
[redacted]
v Cohort 1 participants will begin an OBR on Day 15 (Cohort 1B) or Day 1 SC (Cohort 1A). Cohort 2 participants will begin an OBR on Day 1. An OBR should be selected based on the screening and/or available historical HIV resistance reports.
w All participants will be administered SC GS-6207 at Day 1 SC (14 days after the first dose of oral GS-6207) and will continue to receive SC GS-6207 every 6 months (26 weeks) ± 2 weeks from last injection. Each SC GS-6207 dosing should occur 6 months (26 weeks) ± 2 weeks from last injection.
x Participants ≥ 18 years of age at Day 1 visit will complete Symptoms Distress Module, SF-36, EQ-5D-5L at Day 1, Weeks 4, 16, 26, and 52, if available (before completing other study procedures).
y Participants ≥ 18 years of age at Day 1 visit will complete the Numeric Pain Rating Scale at Day 1 SC, Weeks 26 and 52 (after they receive SC GS-6207 injections).
z Provide injection site reaction assessment worksheet and instruct the participants to measure and report injection site reactions following the administration of the SC injections.

Appendix 3. Management of Clinical and Laboratory Adverse Events

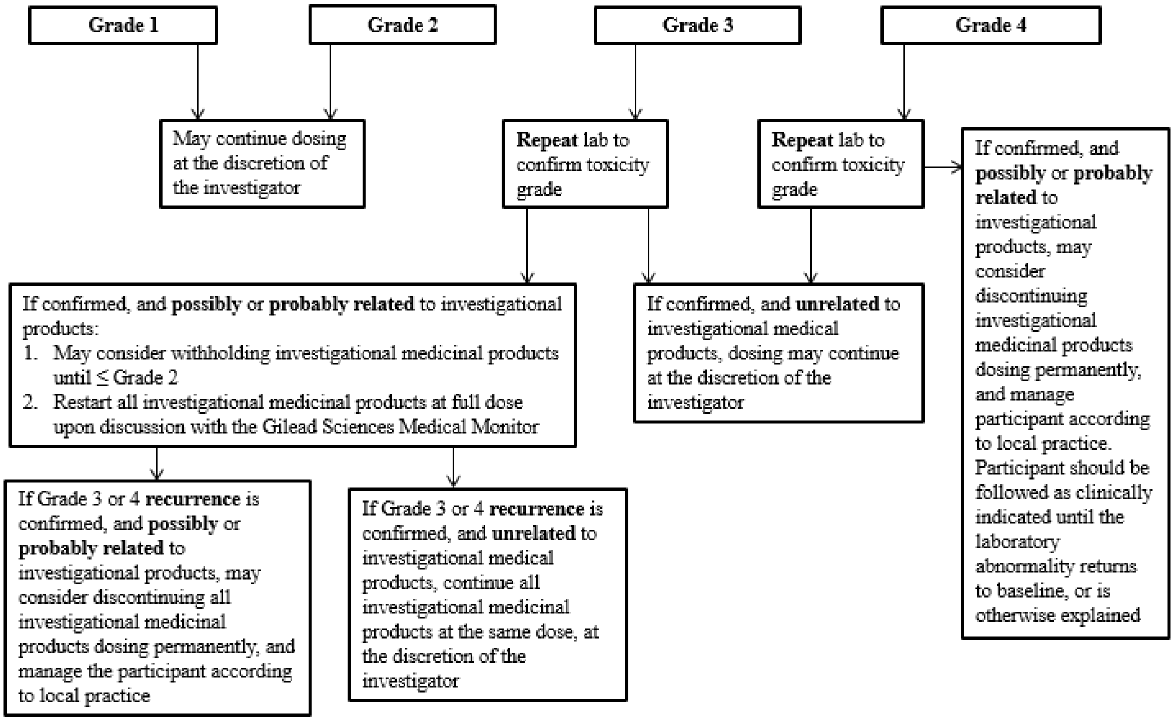

Appendix 4. Pregnancy Precautions, Definition for Women of Childbearing Potential, and Contraceptive Requirements

1) Definitions

a. Definition of Childbearing Potential

For the purposes of this study, a female born participant is considered a woman of childbearing potential following the initiation of puberty (Tanner stage 2) until becoming post-menopausal, unless permanently sterile or with medically documented ovarian failure. For participants deemed by the investigator to have initiated puberty, no documentation of Tanner stage will be required.

Female born participants are considered to be in a postmenopausal state when they are ≥ 54 years of age with cessation of previously occurring menses for ≥ 12 months without an alternative cause. In addition, female born participants < 54 years of age with amenorrhea of ≥ 12 months may also be considered postmenopausal if their follicle stimulating hormone (FSH) level is in the postmenopausal range and they are not using hormonal contraception or hormonal replacement therapy.

For the purposes of this study, permanent sterilization is hysterectomy, bilateral oophorectomy, or bilateral salpingectomy in a female born participant of any age. Tubal ligation is not considered permanent sterilization.

b. Definition of Male Fertility

For the purposes of this study, a male born participant is considered fertile after the initiation of puberty unless permanently sterile by bilateral orchidectomy or medical documentation.

2) Contraception Requirements for Female Born Participants

a. Study Drug Effects on Pregnancy and Hormonal Contraception

Nonclinical toxicity studies of GS-6207 have demonstrated no adverse effect on fertility or embryo-fetal development. However, there are no clinical studies of GS-6207 in pregnant women. Based on in vitro and in vivo drug–drug interaction liability assessment, a clinically significant drug-drug interaction with GS-6207 and hormonal contraceptives is not expected; an oral contraception drug-drug interaction study was not done.

b. Contraception Requirements for Female Born Participants of Childbearing Potential

The inclusion of female born participants of childbearing potential requires using at least an acceptable effective contraceptive. They must have a negative serum pregnancy test at screening and a negative pregnancy test at the Day 1 visit prior to the dose of study drug. Pregnancy tests will be performed as defined by the Study Procedures Table (Appendix 2). In the event of a delayed menstrual period (over one month between menstruations), a pregnancy test must be performed to rule out pregnancy. This is also applicable for female born participants of childbearing potential with infrequent or irregular periods.

Duration of contraception for female born participants of childbearing potential enrolled in this clinical trial should start from screening visit until 60 days after the last dose of oral study drug or 700 days following the last dose of SC study drug, whichever is later, as applicable.

Female born participants of childbearing potential must agree to 1 of the following contraceptive methods:

- Complete abstinence from intercourse of reproductive potential. Abstinence is an acceptable method of contraception only when it is in line with the participant's preferred and usual lifestyle.

Or

- Consistent and correct use of 1 of the following methods of birth control listed below:
  - Hormonal and non-hormonal intrauterine device (IUD)
  - Bilateral tubal occlusion sterilization (upon medical assessment of surgical success)
  - Vasectomy in the male born partner (upon medical assessment of surgical success)

Or

Female born participants who initiate use of a hormonal contraceptive > 7 days after onset of menses as one of their birth control methods should use additional back-up contraception (e.g. condoms) or avoid sexual intercourse for 7 days. Hormonally-based contraceptives and barrier methods permitted for use in this protocol are as follows:

- Hormonal Methods
  - Oral contraceptives (either combined or progesterone only)
  - Injectable progesterone*
  - Subdermal contraceptive implant*
  - Transdermal contraceptive patch*
  - Contraceptive vaginal ring*
- Barrier methods
  - Male condom (with or without spermicide)
  - Female condom (with or without spermicide)
  - Diaphragm with spermicide*
  - Cervical cap with spermicide*
  - Sponge with spermicide

* Not approved in Japan.

Inclusion of methods of contraception in this list of permitted methods does not imply that the method is approved in any country or region. Methods should only be used if locally approved.

Female born participants must also refrain from egg donation and in vitro fertilization during treatment and until the end of contraception requirement.

3) Contraception Requirements for Male Born Participants

No contraception measures are needed.

Condoms should be used for all sexual activity including oral, vaginal, and anal sexual contact to decrease the risk of transmission of HIV and other sexually transmitted diseases.

4) Unacceptable Birth Control Methods

Birth control methods that are unacceptable include periodic abstinence (eg, calendar, ovulation, symptothermal, post-ovulation methods), withdrawal (coitus interruptus), spermicides only, and lactational amenorrhea method (LAM). A female condom and a male condom should not be used together.

5) Procedures to be Followed in the Event of Pregnancy

Female born participants who are pregnant or suspect they are pregnant will be instructed to notify the investigator at any time during the study and within 700 days following the last dose of SC GS-6207, or 60 days following the last oral dose of GS-6207. Male participants whose partner has become pregnant or suspects she is pregnant during the study, including the protocol-defined post-treatment follow-up period, must report the information to the investigator. Female born participants who become pregnant or who suspect that they are pregnant during the study should discontinue study drug immediately. Instructions for reporting pregnancy, partner pregnancy, and pregnancy outcome are outlined in Section 7.7.2.1.

Appendix 5. Pandemic Risk Assessment and Mitigation Plan

In the event of an ongoing pandemic (such as COVID-19), potential risks associated with participants being unable to attend study visits have been identified for this study.

These risks can be summarized as follows:

- Completion of study visits which require on-site study drug administration:
  - Oral and SC study drug administration must occur at the study site. Participants may be unable to return to the site, or the site may be unable to accept any participant visits. If a dosing visit is missed, the participant would not be able to complete dosing per protocol.

    Mitigation plan: Prior to initiation of study drug dosing, investigator or designee should ensure investigator or designee and participant availability to complete dosing visits from Day 1 through Day 1 SC visit. If oral or SC dosing (at Day 1 SC and subsequent) is not completed per protocol, sites should inform Sponsor immediately upon becoming aware of the issue and obtain Sponsor's input for participant's dosing management.

- Study drug shortage at the sites:
  - Delayed or missed study drug shipment to the sites will cause a shortage of study drug at the sites.

    Mitigation plan: The investigator or designee should closely monitor their study drug inventory. Site staff should notify the sponsor or delegate if they foresee shortage in study drug inventory or if there is any interruption in local shipping service. The sponsor will continue to monitor inventory at the study drug depot and study sites. Manual shipments will be triggered as necessary.

- Participant safety monitoring and follow-up:
  - Participants cannot come to the study site for their scheduled study visits as required per protocol.

    Mitigation plan: For participants who cannot come to the study site for their scheduled study visits as required per protocol, the principal investigator (PI) or qualified delegate will conduct a virtual study visit, via phone or video conferencing, to assess the participant within target visit window date whenever possible. During the virtual study visit, the following information at minimum will be reviewed:

    - Confirm if participant has experienced any AEs/SAEs and follow-up on any unresolved AE/SAEs.

    - Review current list of concomitant medications and document any new concomitant medications.

  - ■ Review any changes in medical history
  - ■ Remind participant to continue to administer other ARVs (failing regimen or optimized background regimen)

- — Safety blood draws & central laboratory analysis cannot be done.

  Mitigation plan: Local labs may be utilized as appropriate to monitor participant safety until the participant can return to the site for their regular follow-up per protocol per PI discretion. Any laboratory assessments conducted at a local laboratory due to the pandemic will be documented accordingly.

- — Participants are unable to attend the study visit to sign an updated informed consent form (ICF) version.

  Mitigation plan: The site staff will follow their approved consent process and remain in compliance with local EC/IRB and national laws and regulations. Remote consent will be allowed if approved by the local EC/IRB. The consent process will be documented and confirmed by normal consent procedure at the earliest opportunity.

- Protocol and monitoring compliance:

  - — Protocol deviations in case scheduled visits cannot occur as planned per protocol.

    Mitigation plan: If it is not possible to complete a required procedure at scheduled visit, the visit should be conducted as soon as possible when conditions allow. The situation should be recorded and explained as a protocol deviation. Any missed participant visits or deviation to the protocol due to the pandemic must be reported in the e-case report form (CRF) and described in the clinical study report. Any virtual study visits that are conducted in lieu of clinic visits due to the pandemic will be documented as a protocol deviation related to the pandemic.

  - — Onsite monitoring visit is not feasible.

    Monitors may be unable to carry out source data review (SDR) or source data verification (SDV), study drug accountability, or protocol and GCP/GPSP compliance. This may lead to delays in SDV, an increase in protocol deviations, or under reporting of AEs.

    Mitigation plan: The study monitor is to remain in close communication with the site to ensure data entry and query resolution (remote SDV not allowed). The study monitor is to reference the Study Monitoring Plan for guidance on how to conduct a remote monitoring visit. The study staff is to save and document all relevant communication in the study files. The status of sites that cannot accept monitoring visits and/or participants on site, must be tracked centrally and updated on a regular basis.

- Missing data and data integrity:

  — Increased number of missing data due to participants missing visits/assessments. This could have an impact on the analysis and the interpretation of clinical trial data.

    Mitigation plan: Implications of a pandemic on methodological aspects for the study will be thoroughly assessed and documented, and relevant actions will be taken as appropriate (ie, modification of the statistical analysis plan) and in compliance with Regulatory Authorities' guidance. Overall, the CSR will describe the impact of the pandemic on the interpretability of study data.

Virtual visits should be documented in the participant's source documents. For any completed virtual visits, associated data will be entered in EDC and a general comment will be added noting that the visit was completed virtually due to pandemic.

Risks will be assessed continuously, and temporary measures will be implemented to mitigate these risks as part of a mitigation plan, as described above. These measures will be communicated to the relevant stakeholders as appropriate and are intended to provide alternate methods that will ensure the evaluation and assessment of the safety of participants who are enrolled in this study.

Since these potential risks would be considered mitigated with the implementation of these measures, the expected benefit risk assessment of study drug(s) in study participants remains unchanged.

Appendix 6. Country-Specific Requirements

Not applicable.

Appendix 7. Amendment History

A high-level summary of the history of this study's amendment is provided in tabular form in the subsection below, with changes listed in each table in order of importance. Minor changes such as the correction of typographic errors, grammar, or formatting are not detailed.

Earlier separate summaries of changes are available upon request.

A separate tracked change (red-lined) document comparing Amendment 3 to this amendment will be made available upon the publication of this protocol.

Amendment 4 (18 January 2023)

Rationale for Key Changes Included in Amendment 4	Affected Sections
Long-term data collection were added to support that lenacapavir in combination with optimized background regimen at Weeks 104 and 156 (from the first subcutaneous [SC] dose of GS-6207) have sustained durable virologic responses, clinically meaningful improvement in CD4+, and was well-tolerated in people living with human immunodeficiency virus with multidrug resistance and limited options. Secondary objectives and endpoints have been added to reflect this long-term data collection. To reflect the long-term data collection window, Year 2 and 3 analyses have been added to the Week 104 and 156 analysis window.	Synopsis, Study Design Schema, Sections 2, 3, 5.5, 8, 8.2.3, and 8.2.4
Since worldwide marketing authorization applications are upcoming, ongoing or approved for lenacapavir participants willing to continue the study beyond Week 52 visit are given the option to also receive SC GS-6207 until the product becomes commercially available.	Synopsis, Section 3, Appendix 2
Clinical studies that are completed and summarized in the Investigator's Brochure were removed from the Background.	Section 1
Business critical update to the language in Section 9.2.2 has been streamlined to avoid misalignment with the publications language in the clinical trial agreement.	Section 9.2.2
Minor changes to correct typographic errors.	Throughout, as needed

Prot-GS-US-200-4625 amd-4

ELECTRONIC SIGNATURES

Signed by	Meaning of Signature	Server Date (dd-MMM-yyyy hh:mm:ss)
PPD	Clinical Research eSigned	19-Jan-2023 19:30:26
