# Supplementary material for: Subcutaneous Lenacapavir in People With Multidrug-Resistant HIV-1: 156 Week Results of the CAPELLA Study
Source: Open Forum Infect Dis. 2025 Dec 19;13(1):ofaf763. doi: 10.1093/ofid/ofaf763 (PMC12740718; doi:10.1093/ofid/ofaf763)
Supplement: ofaf763_Supplementary_Data [file ofaf763_supplementary_data.zip › GS-US-200-4625-Year 2 & 3-SAP_f-redact.pdf]

STATISTICAL ANALYSIS PLAN

**Study Title:**	A Phase 2/3 Study to Evaluate the Safety and Efficacy of Long Acting Capsid Inhibitor GS-6207 in Combination with an Optimized Background Regimen in Heavily Treatment Experienced People Living with HIV-1 Infection with Multidrug Resistance
**Name of Test Drug:**	Lenacapavir (LEN; GS-6207)
**Study Number:**	GS-US-200-4625
**Protocol Version (Date):**	Amendment 4 (18 January 2023)
**Analysis Type:**	Year 2 and Year 3 Interim Analyses
**Analysis Plan Version:**	Version 1.0
**Analysis Plan Date:**	11 April 2023
**Analysis Plan Author(s):**	PPD

CONFIDENTIAL AND PROPRIETARY INFORMATION

TABLE OF CONTENTS

TABLE OF CONTENTS 2
LIST OF IN-TEXT TABLES 3
LIST OF ABBREVIATIONS 4
1. INTRODUCTION 6
1.1. Study Objectives 6
1.2. Study Design 7
1.3. Sample Size and Power 8
2. TYPE OF PLANNED ANALYSIS 9
2.1. Interim Analyses 9
2.1.1. Data Monitoring Committee Analysis/Primary Efficacy Analysis 9
2.1.2. Primary (Week 26) Analysis 9
2.1.3. Week 52 Analysis 10
2.1.4. Oral Bridging Interim Analysis 10
2.1.5. Year 2 Analysis 10
2.1.6. Year 3 Analysis 10
2.2. Final Analysis 10
3. GENERAL CONSIDERATIONS FOR DATA ANALYSES 11
3.1. Analysis Sets 11
3.1.1. All Enrolled Analysis Set 11
3.1.2. Full Analysis Set (All LEN Analysis) 11
3.1.3. Safety Analysis Set (All LEN Analysis) 12
3.2. Participant Grouping 12
3.3. Strata and Covariates 12
3.4. Examination of Participant Subgroups 12
3.4.1. Participant Subgroups for Efficacy Analyses 12
3.4.2. Participant Subgroups for Safety Analyses 13
3.5. Multiple Comparisons 13
3.6. Missing Data and Outliers 13
3.6.1. Missing Data 13
3.6.2. Outliers 13
3.7. Data Handling Conventions and Transformations 13
3.8. Analysis Visit Windows 15
3.8.1. Definition of Key Dates and Study Day 15
3.8.2. Analysis Visit Windows 16
3.8.3. Selection of Data in the Event of Multiple Records in an Analysis Visit Window 17
4. PARTICIPANT DISPOSITION 18
4.1. Participant Enrollment and Disposition 18
4.2. Study Drug Administration and Adherence 19
4.3. Study Duration 20
4.4. Protocol Deviations 20
5. BASELINE CHARACTERISTICS 22
5.1. Demographics and Baseline Characteristics 22
5.2. Other Baseline Characteristics 22
5.3. Medical History 24

6. EFFICACY ANALYSES 25
6.1. Primary Efficacy Endpoint 25
6.2. Secondary Efficacy Endpoints 25
6.2.1. US FDA-defined Snapshot Algorithm 25
6.3. Other Efficacy Endpoints 27
6.3.1. Definition of Other Efficacy Endpoints 27
6.3.2. Analysis of Other Efficacy Endpoints 27
6.4. Subgroup Analysis 28
6.5. Changes from Protocol-Specified Efficacy Analyses 28
7. SAFETY ANALYSES 29
7.1. Adverse Events and Deaths 29
7.1.1. Adverse Event Dictionary 29
7.1.2. Adverse Event Severity 29
7.1.3. Relationship of Adverse Events to Study Drug 29
7.1.4. Serious Adverse Events 29
7.1.5. Treatment-Emergent Adverse Events 29
7.1.6. Summaries of Adverse Events and Deaths 30
7.1.7. Additional Analysis of Adverse Events 31
7.2. Laboratory Evaluations 32
7.2.1. Summaries of Numeric Laboratory Results 33
7.2.2. Graded Laboratory Values 34
7.3. Body Weight and Vital Signs 34
7.4. Antiretroviral Medications and Concomitant Medications 35
7.4.1. Antiretroviral Medications 35
7.4.2. Concomitant Medications 36
7.5. Electrocardiogram Results 37
7.5.1. Investigator Electrocardiogram Assessment 37
7.6. Other Safety Measures 37
7.7. Participant Subgroup for Safety Endpoints 37
7.8. Changes from Protocol-Specified Safety Analyses 37
8. PHARMACOKINETIC (PK) ANALYSES 38
9. REFERENCES 39
10. SOFTWARE 40
11. SAP REVISION 41
12. APPENDICES 42

LIST OF IN-TEXT TABLES

Table 3-1. Analysis Windows for All LEN Analysis 16

LIST OF ABBREVIATIONS

AE	adverse event
ARV	antiretroviral
BLQ	below the limit of quantitation
BID	twice daily
BMI	body mass index
CI	confidence interval
CMH	Cochran-Mantel-Haenszel
COBI	cobicistat
CSR	clinical study report
CV	coefficient of variation
DAIDS	Division of AIDS
DMC	data monitoring committee
ECG	electrocardiogram
eCRF	electronic case report form
FAS	Full Analysis Set
FDA	Food and Drug Administration
GSS	genotypic sensitivity score
HLT	high-level term
ID	identification
INSTI	integrase strand-transfer inhibitor
ISR	injection site reaction
LEN	lenacapavir; GS-6207
LOQ	limit of quantitation
M = E	missing = excluded
M = F	missing = failure
MDR	multidrug resistance
MedDRA	Medical Dictionary for Regulatory Activities
NNRTI	non-nucleoside reverse transcriptase inhibitors
NRTI	nucleoside reverse transcriptase inhibitors
OBR	optimized background regimen
OSS	overall susceptibility score
PI	protease inhibitor
PK	pharmacokinetics
PWH	people with HIV
PP	per protocol
PSS	phenotypic sensitivity score
PT	preferred term
RTV	ritonavir

Q1, Q3	first quartile, third quartile
SAE	serious adverse event
SAP	statistical analysis plan
SC	subcutaneous
SD	standard deviation
SE	standard error
SOC	system organ class
TEAE	treatment-emergent adverse event
TFLs	tables, figures, and listings
US	United States
WHO	World Health Organization
yrs	years

1. INTRODUCTION

This statistical analysis plan (SAP) describes the statistical analysis methods and data presentations to be used in tables, figures, and listings (TFLs) of the Year 2 and Year 3 analyses for Study GS-US-200-4625. The Year 2 and Year 3 analysis will be performed when all participants in both cohorts have had HIV-1 RNA data in the Week 104 and Week 156 analysis windows, respectively (or reached the upper limit of the corresponding analysis window with missing HIV-1 RNA data), or prematurely discontinued or completed the study drug.

This SAP is based on the study protocol amendment 4 dated 18 January 2023 and the electronic case report form (eCRF). The SAP will be finalized prior to data finalization for the Year 2 analysis.

1.1. Study Objectives

The primary objective of this study is as follows:

- To evaluate the antiviral activity of LEN administered as an add-on to a failing regimen (functional monotherapy) for PWH with multidrug resistance (MDR) as determined by the proportion of participants achieving at least 0.5 $\log_{10}$ reduction from baseline in HIV-1 RNA at the end of the Functional Monotherapy Period

The secondary objective of this study is as follows:

- To evaluate the safety and efficacy of LEN in combination with an optimized background regimen (OBR) at Weeks 26 and 52
- To evaluate the safety and efficacy of GS-6207 in combination with an OBR at Weeks 104 and 156 (from the first subcutaneous [SC] dose of GS-6207)

The exploratory objectives of this study are:

- To evaluate the emergence of capsid inhibitor resistance
- To evaluate the plasma pharmacokinetics (PK) of LEN
- To identify or validate genetic markers that may be predictive of the natural history of disease, response to therapy, and/or tolerability of medical therapies through genetic discovery research (eg, pharmacogenomics), in participants who provide their specific consent
- To assess the effect of treatment on health related quality of life

1.2. Study Design

This is a Phase 2/3 global multicenter study of LEN together with an OBR in PWH with MDR infection. Participants who complete a Screening visit will return to the clinic between 14 and 30 days after the Screening visit, for a Cohort Selection visit. HIV-1 RNA results from the Cohort Selection visit will be used to determine whether eligible participants will participate in Cohort 1 or Cohort 2.

Cohort 1 (n=36)

Functional Monotherapy Period

Eligible participants with a < 0.5 $\log_{10}$ HIV-1 RNA decline compared to the Screening visit and HIV-1 RNA ≥ 400 copies/mL at the Cohort Selection visit will be randomized in a 2:1 ratio to receive either oral LEN or placebo to match LEN for 14 days. Treatment assignment will be blinded to the sponsor, participants, investigators and study staff at the site. Functional monotherapy will be assessed while participants continue their failing regimen. After each participant completes the Functional Monotherapy Period, their treatment assignment will be unblinded.

Maintenance Period

Participants who were randomized to receive oral LEN will receive subcutaneous (SC) LEN and initiate their OBR on Day 1 SC (14 days after the first dose of oral LEN) **(Cohort 1A)**.

Participants who were randomized to receive placebo will receive oral LEN and initiate their OBR on Day 15 **(Cohort 1B)**. They will receive SC LEN at Day 1 SC (14 days after the first dose of oral LEN) while continuing their OBR.

After the Day 1 SC visit, all Cohort 1 participants will continue with study visits and receive their subsequent SC LEN injection at the Week 26 visit. At the Week 52 visit, participants will be given an option to receive SC LEN injection and continue on the study to receive SC LEN injections every 6 months (26 weeks).

Cohort 2 (n=64)

Oral Lead-in Period

Participants will be enrolled into Cohort 2 if Cohort 1 is fully enrolled or if they do not meet the criteria for randomization in Cohort 1 (ie, they had ≥ 0.5 $\log_{10}$ HIV-1 RNA decline compared to the Screening visit and/or HIV-1 RNA < 400 copies/mL at the Cohort Selection visit). All Cohort 2 participants will receive oral LEN for 14 days starting at Day 1. Participants will initiate an OBR on Day 1.

Maintenance Period

At Day 1 SC (14 days after the first dose of oral LEN), participants will receive SC LEN and will continue their OBR. After the Day 1 SC visit, participants will continue with study visits and receive their subsequent SC LEN injection at the Week 26 visit. At the Week 52 visit, participants will be given an option to receive SC LEN injection and continue the study to receive SC LEN injections every 6 months (26 weeks).

For both Cohorts 1 and 2, data collected through Week 52 (52 weeks from the first dose of SC LEN) refer to as the “Main Phase” of the study. Data collected post Week 52 refer to as the “Extension Phase” of the study.

Schedule of Assessments is provided in Section 12 (Appendix 1).

1.3. Sample Size and Power

A total of 36 participants in Cohort 1 will provide at least 90% power to detect a 60% difference in the proportion of participants achieving a $\geq 0.5 \log_{10}$ reduction from baseline at Day 15 of the Functional Monotherapy Period between the treatment groups (LEN in Cohort 1A and placebo in Cohort 1B).

In this sample size and power computation, it is assumed that 70% and 10% of participants achieve a $\geq 0.5 \log_{10}$ reduction from baseline in HIV-1 RNA in the LEN group (Cohort 1A) and the placebo group (Cohort 1B), respectively (based on data from Trogarzo Phase 3 TMB-301 study {Emu 2018}), and the Fisher exact test is conducted at 2-sided significant level of 0.05.

A total sample size of 36 participants from Cohorts 1A and 1B will provide reasonable assessment of safety through at least 26 weeks of LEN treatment in heavily treatment experienced participants.

2. TYPE OF PLANNED ANALYSIS

2.1. Interim Analyses

2.1.1. Data Monitoring Committee Analysis/Primary Efficacy Analysis

One external multidisciplinary Data Monitoring Committee (DMC) analysis was conducted to review the progress of the study and to perform interim reviews of the efficacy and safety data. The DMC convened after all participants in Cohort 1 had completed or discontinued the study drug in the Functional Monotherapy Period. Treatment assignment was unblinded for the DMC analysis.

No formal stopping rules were used by the DMC for safety outcomes. Rather, a clinical assessment was made to determine if the nature, frequency, and severity of adverse events (AEs) associated with a study regimen warranted the early termination of the study in the best interest of the participants.

Gilead did not have a prior intent to ask the DMC to consider early termination of the study even if there is an early evidence of favorable efficacy. However, Gilead would stop further enrollment if 50% or more of the participants in the LEN group fail in Cohort 1 to achieve at least 0.5 log_{10} reduction from baseline in HIV-1 RNA at the end of the Functional Monotherapy Period. The decision was to continue with the study and the development of LEN based on the magnitude of the HIV-1 RNA decline at the end of the Functional Monotherapy Period.

The DMC's role and responsibilities and the scope of analysis to be provided to the DMC are defined in a mutually agreed upon charter, which defines the DMC membership, meeting logistics, and meeting frequency, and in the DMC SAP.

2.1.2. Primary (Week 26) Analysis

Analysis at Week 26 was conducted after all participants in Cohort 1 had completed the Week 26 visit (ie, 26 weeks after the first dose of SC LEN) or had prematurely discontinued the study drug, outstanding data queries had been resolved or adjudicated as unresolvable, and the data had been cleaned and finalized for the analysis. The data from Week 26 analysis were used to support the LEN regulatory filing for the indication in the heavily treatment experienced PWH.

2.1.3. Week 52 Analysis

Analysis at Week 52 were conducted after all participants in Cohort 1 have completed the Week 52 visit (ie, 52 weeks after the first dose of SC LEN) and after all participants in Cohort 2 have completed the Week 26 visit (ie, 26 weeks after the first dose of SC LEN) or have prematurely discontinued the study drug, outstanding data queries have been resolved or adjudicated as unresolvable, and the data had been cleaned and finalized for the analysis. The data from Week 52 analysis were used to support the LEN regulatory filing for the indication in the heavily treatment experienced PWH.

2.1.4. Oral Bridging Interim Analysis

Oral Bridging Interim Analysis was an adhoc analysis. It was conducted after all participants who received oral bridging of LEN had resumed their SC injection of LEN or had prematurely discontinued the study drug, outstanding data queries have been resolved or adjudicated as unresolvable, and the data had been cleaned and finalized for the analysis. The data from Oral Bridging Interim Analysis may be used to support the regulatory filing for oral bridging with once-weekly 300 mg LEN for planned missed doses of SC LEN injection in heavily treatment experienced PWH.

2.1.5. Year 2 Analysis

The Year 2 Analysis will be performed after all participants in both cohorts have had HIV-1 RNA data in the Week 104 analysis window (or reached the upper limit of the analysis window with missing HIV-1 RNA data), or have prematurely discontinued or completed the study drug, outstanding data queries have been resolved or adjudicated as unresolvable, and the data have been cleaned and finalized.

This SAP describes the analysis plan for the Year 2 Analysis.

2.1.6. Year 3 Analysis

The Year 3 Analysis will be performed after all participants in both cohorts have had HIV-1 RNA data in the Week 156 analysis window (or reached the upper limit of the analysis window with missing HIV-1 RNA data), or have prematurely discontinued or completed the study drug, outstanding data queries have been resolved or adjudicated as unresolvable, and the data have been cleaned and finalized.

This SAP describes the analysis plan for the Year 3 Analysis.

2.2. Final Analysis

After all participants have completed the study, outstanding data queries have been resolved or adjudicated as unresolvable, and the data have been cleaned and finalized, final analysis of the data will be performed.

3. GENERAL CONSIDERATIONS FOR DATA ANALYSES

Year 2 and Year 3 Analyses will be based on All LEN Analysis.

All LEN Analysis include data collected from participants who receive at least one dose of LEN (ie, oral LEN [blinded or open-label] or SC LEN). Data collected on and after the first dose of LEN are included in this analysis. Data included in this analysis will be used to assess efficacy, and safety LEN. Results will be summarized by cohort and total, unless specified otherwise.

General Analysis Approaches

Analysis results will be presented using descriptive statistics. For categorical variables, the number and percentage of participants in each category will be presented; for continuous variables, the number of participants (n), mean, standard deviation (SD) or standard error (SE), median, first quartile (Q1), third quartile (Q3), minimum, and maximum will be presented.

By-participant listings will be presented for all participants in the All Enrolled Analysis Set and sorted by cohort, participant identification (ID) number, visit date, and time (if applicable). Data collected on log forms, such as AEs, will be presented in chronological order within the participant. The treatment group to which participants were enrolled will be used in the listings. Age, sex at birth, race, and ethnicity will be included in the listings, as space permits.

3.1. Analysis Sets

Analysis sets define the participants to be included in an analysis. Analysis sets and their definitions are provided in this section. The analysis set will be identified and included as a subtitle of each TFLs.

For each analysis set, the number and percentage of participants will be summarized by cohort based on the All Enrolled Analysis Set.

A listing of reasons for exclusion from analysis sets will be provided by participant.

3.1.1. All Enrolled Analysis Set

All Enrolled Analysis Set includes all participants who were randomized into Cohort 1 or enrolled into Cohort 2. This is the primary analysis set for by-participant listings.

3.1.2. Full Analysis Set (All LEN Analysis)

The Full Analysis Set (FAS) for the All LEN Analysis includes all participants who are enrolled into the study and receive at least one dose of any LEN.

3.1.3. Safety Analysis Set (All LEN Analysis)

The Safety Analysis Set includes all participants who are enrolled and receive at least one dose of any LEN. This is the primary analysis set for safety analyses. This analysis set will be used to describe baseline information as well as safety summary.

3.2. Participant Grouping

For analyses based on the FAS, participants will be grouped according to the treatment to which they were enrolled. For analyses based on the Safety, and PK Analysis Sets, participants will be grouped according to the actual treatment received.

3.3. Strata and Covariates

This study does not use a stratified randomization schedule when enrolling participants. No covariates will be included in efficacy analyses.

3.4. Examination of Participant Subgroups

3.4.1. Participant Subgroups for Efficacy Analyses

The proportion of participants with HIV-1 RNA < 50 copies/mL at Week 104 as determined by the United States (US) Food and Drug Administration (FDA)-defined snapshot algorithm {U. S. Department of Health and Human Services 2015} will be analyzed for the following participant subgroups based on FAS for the All LEN Analysis:

- Age (years): (a) < 50 and (b) ≥ 50
- Sex: (a) male and (b) female
- Race: (a) black and (b) nonblack
- Region: (a) US and (b) Ex-US
- Baseline CD4 cell count categories (/µL): (a) < 200 and (b) ≥ 200
- Baseline viral load categories (copies/mL): (a) ≤ 100,000 and (b) > 100,000
- Baseline INSTI resistance categories: (a) with INSTI resistance and (b) without INSTI resistance
- Genotypic sensitivity score (GSS) based on baseline OBR: eg, 0, 1, 2, etc.
- Phenotypic sensitivity score (PSS) based on baseline OBR: eg, 0, 1, 2, etc.
- Overall susceptibility score (OSS) based on baseline OBR: eg, 0, 1, 2, etc.
- Use of dolutegravir (DTG) and/or darunavir (DRV) in baseline OBR: (a) With DTG and DRV (b) With DTG, without DRV (c) Without DTG, with DRV (d) Without DTG or DRV

- Use of ibalizumab (IMAB) and/or fostemsavir (FTR) in baseline OBR: (a) With IMAB and FTR (b) With IMAB, without FTR (c) Without IMAB, with FTR (d) Without IMAB or FTR (e) With either IMAB or FTR (f) With IMAB, regardless of FTR (g) Without IMAB, regardless of FTR (h) Regardless of IMAB, with FTR (i) Regardless of IMAB, without FTR
- Number of fully active ARV agents from baseline OBR: 0, 1, 2, 2 or more, 3 or more

3.4.2. Participant Subgroups for Safety Analyses

Incidence of all treatment-emergent AEs (TEAEs) will be analyzed for the following participant subgroups using the Safety Analysis Set for All LEN Analysis

- Age (years): (a) < 50 and (b) ≥ 50
- Sex: (a) male and (b) female
- Race: (a) black and (b) nonblack
- Region: (a) US and (b) Ex-US

3.5. Multiple Comparisons

No alpha level adjustment is needed as the primary efficacy endpoint was analyzed at the DMC analysis and has been included in the Week 26 CSR.

3.6. Missing Data and Outliers

3.6.1. Missing Data

In general, missing data will not be imputed unless methods for handling missing data are specified. Exceptions are presented in this document.

For missing last study date, imputation rules are described in Section 4.3. The handling of missing or incomplete dates for AE onset is described in Section 7.1.5.2, and for antiretroviral medications and concomitant medications in Section 7.4.

3.6.2. Outliers

Outliers will be identified during the data management and data analysis process, but no sensitivity analyses will be conducted. All data will be included in the data analysis.

3.7. Data Handling Conventions and Transformations

Only year of birth is collected in this study. The following conventions will be used for the imputation of full date of birth:

- If year of birth is collected, "01 July" will be imputed as the day and month of birth
- If year of birth is missing, date of birth will not be imputed

In general, age collected at baseline (in years) will be used for analyses and presented in listings. If age at baseline is not available for a participant, age derived based on date of birth and the baseline visit date will be used instead. If an enrolled participant was not dosed with any study drug, the randomization/enrollment date will be used instead of the baseline visit date. For screen failures, the date the first informed consent was signed will be used for the age derivation. Age required for longitudinal and temporal calculations and analyses (eg, estimates of creatinine clearance) will be based on age derived from date of birth and the date of the measurement or event, unless otherwise specified.

Non-PK data that are continuous in nature but are less than the lower limit of quantitation (LOQ) or above the upper LOQ will be imputed as follows:

- A value that is 1 unit less than the LOQ will be used to calculate descriptive statistics if the datum is reported in the form of "< x" (where x is considered the LOQ). For example, if the values are reported as < 50 and < 5.0, values of 49 and 4.9, respectively, will be used to calculate summary statistics. An exception to this rule is any value reported as < 1 or < 0.1, etc. For values reported as < 1 or < 0.1, a value of 0.9 or 0.09, respectively, will be used to calculate summary statistics.
- A value that is 1 unit above the LOQ will be used to calculate descriptive statistics if the datum is reported in the form of "> x" (where x is considered the LOQ). Values with decimal points will follow the same logic as above.
- The LOQ will be used to calculate descriptive statistics if the datum is reported in the form of "≤ x" or "≥ x" (where x is considered the LOQ).

Logarithmic (base 10) transformations will be applied to HIV-1 RNA data for efficacy analysis. HIV-1 RNA results of 'No HIV-1 RNA detected' and "<20 cp/mL HIV-1 RNA Detected" will be imputed as 19 copies/mL for analysis purposes.

Natural logarithm transformation will be used for analyzing concentrations and PK parameters. Concentration and PK parameter values that are below the limit of quantitation (BLQ) will be presented as "BLQ" in the data listing. Values that are BLQ will be treated as 0 at predose time points, and one-half the value of the LOQ at postdose time points for summary purposes or statistical model fitting.

The following conventions will be used for the presentation of summary and order statistics for PK concentrations:

- If at least 1 participant has a concentration value of BLQ for the time point, the minimum value will be displayed as "BLQ".
- If more than 25% of the participants have a concentration data value of BLQ for a given time point, the minimum and Q1 values will be displayed as "BLQ".
- If more than 50% of the participants have a concentration data value of BLQ for a given time point, the minimum, Q1, and median values will be displayed as "BLQ".

- If more than 75% of the participants have a concentration data value of BLQ for a given time point, the minimum, Q1, median, and Q3 values will be displayed as "BLQ".
- If all participants have concentration data values of BLQ for a given time point, all order statistics (minimum, Q1, median, Q3, and maximum) will be displayed as "BLQ".

3.8. Analysis Visit Windows

3.8.1. Definition of Key Dates and Study Day

First Dose Date for All LEN Analysis is defined as the earliest dose date of either blinded or open-label oral LEN, as recorded on the Study Drug Administration eCRF form.

Last Dose Date for All LEN Analysis is defined for participants who prematurely discontinue study drug in any phase (ie, main phase or extension phase) of the study or who complete study drug as the latest dose date of either blinded or open-label oral LEN or SC LEN.

First Dose Date for the study is defined as the day when the first dose of blinded study drug (LEN or Placebo) or open-label oral LEN was taken, as recorded on the Study Drug Administration eCRF form.

Last Dose Date for the study is defined for participants who prematurely discontinue study drug in any phase of the study or who complete study drug as the latest dose date of any study drug including blinded study drug, open-label oral LEN, and SC LEN.

First Dose Date of Oral Bridging is defined for participants who received oral bridging of LEN as the earliest dose date of oral bridging of LEN, as recorded on the Study Drug Administration - Oral Bridging eCRF form.

Last Dose Date of Oral Bridging is defined for participants who received oral bridging of LEN, as the latest dose date of oral bridging of LEN before the First Dose Date of Resumed SC Injection, as recorded on the Study Drug Administration - Oral Bridging eCRF form.

First Dose Date of Resumed SC Injection is defined for participants who received oral bridging of LEN and resumed the SC injection, as the earliest dose date of SC LEN following the lifting of the Clinical Hold on SC LEN, as recorded on the Study Drug Administration – SC eCRF form.

Last Study Date is defined as the latest date of non-missing clinic visits and/or laboratory visits, and/or AE onset and end dates without any imputation, including the 30, 90, and 180-day follow-up visit dates for participants who prematurely discontinue or complete the study.

Study Day will be calculated from the First Dose Date, as defined separately above for All LEN Analysis and for the study, and will be derived as follows:

- For postdose study days: Assessment Date – First Dose Date + 1
- For study days prior to the first dose: Assessment Date – First Dose Date

3.8.2. Analysis Visit Windows

For All LEN Analysis, participant's visits might not occur on protocol-specified visits. Therefore, for the purpose of analysis, observations will be assigned to analysis windows (see Table 3-1).

Table 3-1. Analysis Windows for All LEN Analysis

Analysis Visit	Nominal Study Day	Analysis Window Study Day Range	
		HIV-1 RNA and Vital Signs	Hematology, (Urine) Chemistry, Urinalysis, CD4 Cell Count, and CD4%
Baseline		≤ First Dose Date, if time not available<br>≤ First Dose Date/Time, if time available	≤ First Dose Date, if time not available<br>≤ First Dose Date/Time, if time available
Day 1 (Postdose)[a]	1	> First Dose Date/Time, 1	> First Dose Date/Time, 1
Day 2	2	2, 5	NP
Day 8	8	6, 11	2, 11
Day 1 SC	15	12, 29	12, 29
Week 4	43	30, 64	30, 64
Week 10	85	65, 106	65, 106
Week 16	127	107, 148	107, 148
Week 22	169	149, 183	149, 183
Week 26	197	184, 232	184, 232
Week 36	267	233, 323	233, 323
Week 52	379	324, 414	324, 414
Week 62	449	415, 505	415, 505
Week 78	561	506, 596	506, 596
Week 88	631	597, 687	597, 687
Week 104	743	688, 778	688, 778
Week 114	813	779, 869	779, 869
Week 130	925	870, 960	870, 960
Week 140	995	961, 1051	961, 1051
Week 156	1107	1052, 1142	1052, 1142
Week 166	1177	1143, 1233	1143, 1233
Week 182	1289	1234, 1324	1234, 1324
Week 192	1359	1325, 1415	1325, 1415
Week 208	1471	1416, 1506	1416, 1506

NP = Not planned by the protocol

a Day 1 postdose will be used to label the records collected on Day 1 with collection time after the Day 1 dosing time. The Day 1 postdose will be used in listings to identify such records. Day 1 postdose visit will not be included in by-visit table summary

3.8.3. Selection of Data in the Event of Multiple Records in an Analysis Visit Window

Depending on the statistical analysis method, single values may be required for each analysis window. For example, change from baseline by visit usually requires a single value, whereas a time-to-event analysis would not require 1 value per analysis window.

If multiple valid, nonmissing measurements exist in an analysis window, records will be chosen based on the following rules if a single value is needed:

- For baseline, the last nonmissing value with collection date (and time, if available) on or prior to the first dose date (and time) of study drug will be selected, unless specified differently. If there are multiple records with the same time or no time recorded on the same day, the baseline value will be the average of the measurements for continuous data (except for HIV-1 RNA, see below), or the measurement with the lowest severity (eg, normal will be selected over abnormal for safety electrocardiogram [ECG] findings) for categorical data.

- For postbaseline values (ie, data with collection date [and time, if available] after the first dose date [and time] of study drug):

  - The record closest to the nominal day for that visit will be selected with the exception of CD4 cell counts and CD4% in which the latest record will be selected and HIV-1 RNA level (see below).

  - If there are 2 records that are equidistant from the nominal day, the later record will be selected.

  - If there is more than 1 record on the selected day, the average will be taken for continuous data (except for HIV-1 RNA, see below) and the worse severity will be taken for categorical data, unless otherwise specified.

- For baseline HIV-1 RNA, the latest (considering both date and time) record(s) on or prior to the first dose date and time of study drug will be selected. For postbaseline HIV-1 RNA, the latest record(s) on the window will be selected. For both baseline and postbaseline HIV-1 RNA, if both “HIV RNA Taqman 2.0” and “HIV RNA Taqman 2.0 Repeat” or both “HIV RNA COBAS 6800” and “HIV RNA COBAS 6800 Repeat” are available with the same collection time, the results from the “HIV RNA Taqman Repeat” or “HIV RNA COBAS 6800 Repeat” will be selected for analysis purposes; otherwise, if there are multiple “HIV RNA Taqman 2.0” or “HIV RNA COBAS 6800” records with the same collection time, the geometric mean will be taken for analysis purposes.

4. PARTICIPANT DISPOSITION

4.1. Participant Enrollment and Disposition

Key study dates for the first participant screened, first participant enrolled, last participant enrolled, last participant last visit for the primary efficacy endpoint, and last participant last visit for the Year 2 or Year 3 intierm analysis will be provided.

A summary of participant enrollment will be provided by cohort, treatment group, and total. The number and percentage of participants enrolled in each country, investigator within a country, and overall will be summarized. For each column, the denominator for the percentage calculation will be the total number of participants analyzed for that column.

A summary of participant disposition will be provided by cohort, treatment group, and total based on all screened participants. This summary will present the number of participants screened, the number of participants who met all eligibility criteria but were not enrolled with reasons participants not enrolled, the number of participants enrolled, the number of participants enrolled but never treated (if applicable), the number of participants in the Safety Analysis Set, and the number of participants in each of the categories listed below:

Participant Disposition on Study Drug by Phase

- Main Phase (Up to Week 52 visit)
  - Oral lead-in before the first SC injection
    - Completed study drug
    - Prematurely discontinued study drug with reasons (if applicable)
  - After the first SC injection
    - Received Day 1 SC injection
    - Continuing study drug
    - Completed study drug
    - Prematurely discontinued study drug with reasons (if applicable)

- Extension Phase (Post Week 52 visit)
  - Treated in the extension phase
  - Completed study drug
  - Continuing study drug
  - Prematurely discontinued study drug with reasons (if applicable)

Participant Disposition throughout the Study

- Continuing study
- Completed study
- Prematurely discontinued study with reasons (if applicable)

The denominator for the percentage calculation for the main phase categories and for study disposition categories will be the total number of participants in the Safety Analysis Set corresponding to that column. The denominator for the percentage calculation for the extension phase categories will be the total number of participants treated in the extension phase corresponding to that column. In addition, a flowchart will be provided to depict participant disposition on study drug.

The following by-participant listings will be provided by participant ID number in ascending order to support the above summary tables:

- Reasons for premature study drug for each phase/period or study discontinuation throughout the study
- Reasons for screen failure (will be provided by screening ID number in ascending order)

4.2. Study Drug Administration and Adherence

Study drug administration and study drug dispensing information will be collected in the Study Drug Administration and Study Drug Accountability eCRFs. Number of participants receiving oral lead-in LEN and SC LEN at each protocol specified visit (Day 1, Day 2, Day 8, Day 15, Day 16, and Day 22 for oral LEN and Day 1 SC, Week 26, Week 52, Week 78, Week 104, and etc. for SC LEN) will be summarized. Number of participants receiving oral reload before the first SC injection due to missed scheduled oral dosing, and number of participants receiving oral bridging after the first SC injection will also be provided.

Study drug administration and dispensing information including lot number and kit ID (if applicable) will be listed.

The adherence to SC LEN will be assessed by adherence to the projected injection interval, which is 26 weeks (182 days) between 2 adjacent injection visits. The number of days from the projected injection visit date will be calculated for each injection visit (excluding the 1st injection visit) as the injection visit date minus the previous injection visit date minus 182 days minus oral bridging period (ie, First Dose Date of Resumed SC Injection - First Dose Date of Oral Bridging) if any occurred between these 2 adjacent injection visits. The number of days from the projected injection visit date will be classified into the following categories:

- < - 14 days
- -14 to -8 days
- -7 to -3 days
- ± 2 days
- 3 to 7 days
- 8 to 14 days
- > 14 days

The number and percentage of participants in each category will be summarized by cohort and treatment group and overall for the injection visits of interest.

4.3. Study Duration

Due to long-acting feature of the study drug, study duration will be calculated for All LEN Analysis using the Safety Analysis set.

All LEN Analysis Study duration is calculated as the last study date minus the first dose date of LEN plus 1. For participants who are still on study at the time of the Year 2 or Year 3 interim analysis, the data cut date will be used to impute the last study day.

Study duration will be expressed in days and will be summarized using descriptive statistics and using the number and percentage of participants that stayed through the following applicable time periods: 1 day, 2 days, 8 days, 15 days, 43 days, 85 days, 127 days, 169 days, 197 days, etc.

4.4. Protocol Deviations

Participants who did not meet the eligibility criteria for study entry but enrolled in the study will be summarized. The summary will present the number and percentage of participants who did not meet at least 1 eligibility criterion and the number of participants who did not meet specific criteria by cohort, treatment group, and total for the Safety Analysis Set. A by-participant listing will be provided for those participants who did not meet at least 1 eligibility (inclusion or exclusion) criterion. The listing will present the eligibility criterion (or criteria if more than 1 deviation) that participants did not meet and related comments, if collected.

Protocol deviations occurring after participants entered the study are documented during routine monitoring. The number and percentage of participants with important protocol deviations and number of important protocol deviations by deviation category (eg, eligibility criteria, informed consent) will be summarized by cohort and treatment group for the Safety Analysis Set. A by-participant listing will be provided for those participants with important protocol deviation.

5. BASELINE CHARACTERISTICS

5.1. Demographics and Baseline Characteristics

Participant demographic variables (ie, age, sex at birth, gender identity, sexual orientation, race, and ethnicity) and baseline characteristics (body weight [in kg], height [in cm], body mass index [BMI; in kg/m^2]) will be summarized by cohort, treatment group, and total using descriptive statistics for continuous variables and using number and percentage of participants for categorical variables. The summary of demographic data will be provided for the Safety Analysis Set.

For Cohort 1, the Cochran-Mantel-Haenszel (CMH) test will be used to compare the 2 treatment groups (LEN vs. Placebo) for categorical data. The 2-sided Wilcoxon rank sum test will be used to compare the treatment groups (LEN vs. Placebo) for continuous data.

A by-participant demographic and baseline characteristics listing, including the informed consent date, will be provided by participant ID number in ascending order.

5.2. Other Baseline Characteristics

Other baseline characteristics include disease characteristics, HIV and ARV medication history (prior ARV drugs), failing regimen and resistance, and OBR and resistance at baseline:

Disease characteristics will include the following:

- HIV-1 RNA (log_{10} copies/mL)
- HIV-1 RNA categories (copies/mL): (a) ≤ 100,000, (b) > 100,000
- CD4 cell counts (/µL)
- CD4 cell counts categories (/uL): (a) < 50, (b) ≥ 50 to < 200, (c) ≥ 200 to < 350, (d) ≥ 350 to < 500, and (e) ≥ 500
- CD4 percentage (%)

HIV and ARV medication history will include the following:

- Number of years since HIV diagnosis
- Number of years since first started HIV treatment
- Number of prior ARV medications (see definition in Section 7.4.1.1 excluding cobicistat [COBI] and ritonavir [RTV] used as a boosting agent)

- Prior ARV medications drug class exposure: (a) nucleoside reverse transcriptase inhibitors (NRTI), (b) non-nucleoside reverse transcriptase inhibitors (NNRTI), (c) protease inhibitor (PI), (d) integrase strand-transfer inhibitor (INSTI), (e) fusion inhibitor (eg, enfuvirtide), (f) CCR5 entry inhibitor (eg, maraviroc), (g) CD4-directed post-attachment inhibitor (eg, ibalizumab) , (h) attachment inhibitor (eg, fostemsavir), (i) Other, if applicable

- Number of participants with known resistance to ≥ 2 drugs in class

Failing regimen and resistance will include the following:

- Number of failing regimen (see definition in Section 7.4.1.2 excluding COBI and RTV used as a boosting agent)

- Failing regimen class exposure (a) NRTI, (b) NNRTI, (c) PI, (d) INSTI, (e) fusion inhibitor, (f) CCR5 entry inhibitor, (g) CD4-directed post-attachment inhibitor (eg, ibalizumab), (h) attachment inhibitor (eg, fostemsavir), (i) Other, if applicable

- OSS based on failing regimen

- GSS based on failing regimen

- PSS based on failing regimen

- Number of fully active ARV agents from failing regimen

- Use of DTG twice daily (BID) and/or DRV BID in failing regimen: (a) With DTG BID and DRV BID, (b) With DTG BID, without DRV BID, (c) Without DTG BID, with DRV BID, (d) Without DTG BID or DRV BID

OBR and resistance will include the following:

- Number of ARV medications in the baseline OBR

- Baseline OBR drug class exposure (a) NRTI, (b) NNRTI, (c) PI, (d) INSTI, (e) fusion inhibitor, (f) CCR5 entry inhibitor, (g) CD4-directed post-attachment inhibitor (eg, ibalizumab), (h) attachment inhibitor (eg, fostemsavir), (i) Other, if applicable

- OSS based on baseline OBR (as continuous variable and categorical variable: 0, 0.5, 1, 1.5, 2, > 2);

- GSS based on baseline OBR (as continuous variable and categorical variable: 0, 0.5, 1, 1.5, 2, > 2);

- PSS based on baseline OBR (as continuous variable and categorical variable: 0, 0.5, 1, 1.5, 2, > 2);

- Number of fully active ARV agents from baseline OBR
- Use of DTG and/or darunavir DRV in baseline OBR: (a) With DTG and DRV (b) With DTG, without DRV (c) Without DTG, with DRV (d) Without DTG or DRV
- Use of IMAB and/or FTR in baseline OBR: (a) With IMAB and FTR (b) With IMAB, without FTR (c) Without IMAB, with FTR (d) Without IMAB or FTR

Other baseline characteristics will be summarized by cohort and treatment group and overall using descriptive statistics for continuous variables and using number and percentage of participants for categorical variables. The summary of other baseline characteristics will be provided for the Safety Analysis Set.

For Cohort 1, the CMH test will be used to compare the 2 treatment groups for categorical data and the 2-sided Wilcoxon rank sum test will be used to compare the treatment groups for continuous data.

A by-participant listing of other baseline characteristics will be provided by participant ID number in ascending order.

5.3. Medical History

Medical history will be collected at screening including HIV-1 related disease events. Medical history data will be coded and listed.

6. EFFICACY ANALYSES

6.1. Primary Efficacy Endpoint

The primary efficacy endpoint is the proportion of participants in Cohort 1 achieving ≥ 0.5 $\log_{10}$ reduction from baseline in HIV-1 RNA at the end of the Functional Monotherapy Period.

The statistical analysis methods for the primary efficacy endpoint were described in the Week 26 SAP, and the analysis was performed in the Week 26 analysis.

6.2. Secondary Efficacy Endpoints

The secondary efficacy endpoints are:

- The proportion of participants in Cohort 1 with plasma HIV-1 RNA < 50 copies/mL and < 200 copies/mL at Weeks 26 and 52 visits based on the United States (US) Food and Drug Administration (FDA)-defined snapshot algorithm.

- The proportion of participants in combined Cohorts 1 and 2 with plasma HIV-1 RNA < 50 copies/mL and < 200 copies/mL at Weeks 104 and 156 from the first SC dose of GS-6207 based on the US FDA-defined snapshot algorithm.

Analysis will be based on the FAS for All LEN Analysis. This SAP describes the statistical analysis methods for the proportion of participants in combined Cohorts 1 and 2 with plasma HIV-1 RNA < 50 copies/mL and < 200 copies/mL at Weeks 104 and 156 from the first SC dose of GS-6207 based on the US FDA-defined snapshot algorithm.

6.2.1. US FDA-defined Snapshot Algorithm

The analysis window at Week 104 is defined as from Study Day 688 to Study Day 778, inclusive, where Study Day is calculated from the first dose of oral LEN. Participants who have missing HIV-1 RNA at Week 104 and have completed the study before reaching the upper limit of the analysis window for Week 104 will be excluded. All HIV-1 RNA data collected on-treatment will be used in the snapshot algorithm.

HIV-1 RNA on-SC-LEN-treatment is defined as HIV-1 RNA collected up to 28 weeks from the last SC LEN, and on or prior to the first dose of oral bridging of LEN or up to 28 weeks from any resumed SC LEN after oral bridging for participants who received oral bridging of LEN.

HIV-1 RNA on-oral-LEN-treatment is defined as HIV-1 RNA collected after the First Dose Date of Oral Bridging or oral reload after the first SC LEN, and up to 7 days from the Last Dose Date of Oral Bridging for participants who discontinued from the oral bridging of LEN, or up to minimal (Last Dose Date of Oral Bridging +7, First Dose Date of Resumed SC Injection) for participants who resumed SC LEN.

For the snapshot algorithm, HIV-1 RNA on-SC-LEN-treatment will be used. If there is no such result, HIV-1 RNA on-oral-LEN-treatment will be used.

Virologic outcome will be defined as the following categories:

- HIV-1 RNA < 50 copies/mL: this includes participants who have the last available on-treatment HIV-1 RNA < 50 copies/mL in the Week 104 analysis window
- HIV-1 RNA ≥ 50 copies/mL: this includes participants
  - Who have the last available on-treatment HIV-1 RNA ≥ 50 copies/mL in the Week 104 analysis window, or
  - Who do not have on-treatment HIV-1 RNA data in the Week 104 analysis window and
    - Who discontinue study drug prior to or in the Week 104 analysis window due to lack of efficacy, or
    - Who discontinue study drug prior to or in the Week 104 analysis window due to reasons other than AE, death, or lack of efficacy and have the last available on-treatment HIV-1 RNA ≥ 50 copies/mL
- No Virologic Data in the Week 104 analysis window: this includes participants who do not have on-treatment HIV-1 RNA data in the Week 104 analysis window because of the following:
  - Discontinuation of study drug prior to or in the Week 104 analysis window due to AE or death (regardless of whether the last available on-treatment HIV-1 RNA < 50 copies/mL or not) or,
  - Discontinuation of study drug prior to or in the Week 104 analysis window due to reasons other than AE, death, or lack of efficacy and the last available on-treatment HIV-1 RNA < 50 copies/mL or,
  - Missing data during the window but on study drug.

The number and percentage of participants with HIV-1 RNA < 50 copies/mL, HIV-1 RNA ≥ 50 copies/mL, and reasons for no virologic data at Week 104 will be summarized. The 95% CI for the percentage of participants with HIV-1 RNA < 50 copies/mL will be constructed using the Exact method.

The proportion of participants with HIV-1 RNA < 200 copies/mL at Week 104, and the proportion of participants with HIV-1 RNA < 50 copies/mL and < 200 copies/mL at Week 156 will also be summarized in the same manner using the US FDA-defined snapshot algorithm. The analysis window at Week 104 is defined as from Study Day 779 to Study Day 869, inclusive.

The flowchart of the US FDA-defined snapshot algorithm is provided in Section 12 (Appendix 1).

6.3. Other Efficacy Endpoints

6.3.1. Definition of Other Efficacy Endpoints

Other efficacy endpoints that will be performed for All LEN Analysis are provided below.

- Change from baseline in HIV 1 RNA (copies/mL) by visit
- Change from baseline in CD4 cell count (/uL) by visit
- Proportion of participants with HIV 1 RNA < 50 copies/mL by visit based on Missing = Failure and Missing = Excluded analyses

6.3.2. Analysis of Other Efficacy Endpoints

Baseline and the change from baseline in HIV-1 RNA ($\log_{10}$ copies/mL) and CD4 cell count (/µL) by visit for All LEN Analysis will be summarized using descriptive statistics.

Mean ± 95% CI of the change from baseline in HIV-1 RNA ($\log_{10}$ copies/mL) will be plotted by visit.

Number and percentage of participants with HIV-1 RNA < 50 copies/mL by visit will be analyzed using the following analyses:

- Missing = Failure (M = F):

  In this approach, missing data will be treated as virologic failure and summarized into the "missing" category (see list of HIV-RNA categories below). The denominator for percentages at a visit is the number of participants in the FAS, excluding ongoing participants who have missing HIV-1 RNA at a visit and have not reached the upper limit of the analysis window for the corresponding visit and participants who have missing HIV-1 RNA at a visit and have completed the study before reaching the upper limit of the analysis window for the corresponding visit.

- Missing = Excluded (M = E):

  In this approach, missing data will be excluded in the computation of the percentages (ie, missing data points will be excluded from both the numerator and denominator in the computation). The denominator for percentages at a visit is the number of participants in the FAS with nonmissing HIV-1 RNA value at that visit.

No statistical testing is planned. The number and percentage of participants with HIV-1 RNA in the following categories will be summarized:

- < 50 copies/mL
  - < 20 copies/mL
    - < 20 copies/mL Not Detectable
    - < 20 copies/mL Detectable
  - 20 to < 50 copies/mL
- 50 to < 200 copies/mL
- 200 to < 400 copies/mL
- 400 to < 1000 copies/mL
- ≥ 1000 copies/mL
- Missing (only applicable to M = F analysis)

Analysis of the other efficacy endpoints will be based on the FAS.

6.4. Subgroup Analysis

Analysis of subgroups (defined in Section 3.4.1) will be performed for the proportion of participants with HIV-1 RNA < 50 copies/mL at Weeks 104 as determined by the US FDA-defined snapshot algorithm. Subgroup analysis will be performed on FAS for All LEN Analysis set for participants who receive at least 1 dose of SC injection. Results will be descriptive without statistical testing. The 95% CIs of the proportions will be provided using the exact method.

6.5. Changes from Protocol-Specified Efficacy Analyses

No change from protocol-specified efficacy analysis is planned.

7. SAFETY ANALYSES

For participants who discontinue from the oral bridging of LEN, only data collected up to 60 days after Last Dose Date of Oral Bridging will be included. All data collected will be included in data listings.

7.1. Adverse Events and Deaths

7.1.1. Adverse Event Dictionary

Clinical and laboratory AEs will be coded using the current version of the Medical Dictionary for Regulatory Activities (MedDRA). System organ class (SOC), high-level group term, high-level term (HLT), preferred term (PT), and lower-level term will be provided in the AE dataset.

7.1.2. Adverse Event Severity

Adverse events are graded by the investigator as Grade 1, 2, 3, 4, or 5 according to toxicity criteria specified in the protocol. The severity grade of events for which the investigator did not record severity will be categorized as "missing" for tabular summaries and data listings. The missing category will be listed last in summary presentation.

7.1.3. Relationship of Adverse Events to Study Drug

Related AEs are those for which the investigator selected "Related" on the AE eCRF to the question of "Related to Study Treatment." Relatedness will always represent the investigator's choice, not that of the medical monitor. Events for which the investigator did not record relationship to study drug will be considered related to study drug for summary purposes. However, by-participant data listings will show the relationship as missing.

7.1.4. Serious Adverse Events

Serious adverse events (SAEs) will be identified and captured as SAEs if the AEs met the definitions of SAEs that were specified in the study protocol. SAEs captured and stored in the clinical database will be reconciled with the SAE database from the Global Patient Safety (formerly known as Pharmacovigilance and Epidemiology) before data finalization.

7.1.5. Treatment-Emergent Adverse Events

7.1.5.1. Definition of Treatment-Emergent Adverse Events

The TEAEs are defined as any AEs that begin on or after the date of first dose of study drug or any AEs leading to premature discontinuation of study drug. For participants who discontinue from the oral bridging of LEN, only AEs collected up to 60 days after the Last Dose Date of Oral Bridging will be considered treatment emergent.

7.1.5.2. Incomplete Dates

If the onset date of the AE is incomplete and the AE stop date is not prior to the first dose date of study drug, then the month and year (or year alone if month is not recorded) of onset determine whether an AE is treatment emergent. The event is considered treatment emergent if the AE onset is the same as or after the month and year (or year) of the first dose date of study drug.

An AE with completely missing onset and stop dates, or with the onset date missing and a stop date later than the first dose date of study drug, will be treatment emergent. In addition, an AE with the onset date missing and incomplete stop date with the same or later month and year (or year alone if month is not recorded) as the first dose date of study drug will be considered treatment emergent.

When calculating the duration of event or time to onset, the following imputation rule will be used:

Missing start month/day: Jan 1/first day of the month will be used unless this is before the start date of study drug; in this case the study drug start date will be used;

Missing stop month/day: Dec 31/last day of the month will be used, unless this is after the last study date; in this case the last study date will be used.

Completely missing start or end dates will remain missing, with no imputation applied.

7.1.6. Summaries of Adverse Events and Deaths

Treatment-emergent AEs will be summarized based on the Safety Analysis Set.

7.1.6.1. Summaries of AE Incidence

A brief, high-level summary of the number and percentage of participants who experienced at least 1 TEAE in the categories described below will be provided for All LEN Analysis. All deaths observed in the study will also be included in this summary.

The number and percentage of participants who experienced at least 1 TEAE will be provided and summarized by SOC and PT and by PT only, unless specified otherwise, for the following AE categories:

- TEAEs
- TEAEs (by severity)
- TEAEs with Grade 3 or higher (by severity)
- TEAEs with Grade 2 or higher
- TE treatment-related AEs (by severity)
- TE treatment-related AEs with Grade 3 or higher (by severity)
- TE treatment-related AEs with Grade 2 or higher

- TE SAEs
- TE treatment-related SAEs
- TEAEs leading to death (by SOC and PT only)
- TEAEs leading to premature discontinuation of study drug
- TEAEs leading to premature discontinuation of study

Multiple events will be counted only once per participant in each summary. For summaries by SOC and PT, AEs will be summarized and listed first in alphabetic order of SOC and then by PT in descending order of total frequency within each SOC. For summaries by PT, AEs will be summarized and listed by PT in descending order of total frequency. For summaries by severity grade, the most severe grade will be used for those AEs that occurred more than once in an individual participant during the study.

In addition, data listings will be provided for the following:

- All AEs
- All SAEs
- All Deaths
- All AEs with severity of Grade 3 or higher
- All AEs leading to discontinuation of study drug
- All AEs leading to discontinuation of study

For each listing, whether the event is treatment emergent will be indicated.

7.1.7. Additional Analysis of Adverse Events

7.1.7.1. Study Drug Related Injection Site Reactions

Additional analysis of AEs will be performed for injection site reaction (ISR) related to study drug, which is defined as an AE related to study drug with HLT of "Injection Site Reactions". The following summaries will be provided for each SC injection (eg, Day 1 SC, Week 26, and Week 52) and the overall using All LEN Analysis.

- Number of participants that received SC injection
- Number and percentage of participants with study drug related ISRs
- Number and percentage of participants with study drug related ISRs by grade
- Number and percentage of participants with study drug related ISRs by PT term

The denominator for the percentage calculation for the by visit summary and the overall summary will be based on the total number of participants who receive at least 1 SC injection at the visit of interest and the total number of participants who receive at leave 1 SC injection at any injection visit, respectively.

Duration of the ISR will also be calculated and summarized. Duration for a given event is defined as the ISR stop date minus the ISR onset date plus 1 day. For ISR with ongoing stop date, stop date will be imputed as last study date or data cut date, whichever is the earliest. Duration of ISR events in days will be summarized using descriptive statistics.

A by-participant listing for study drug related ISRs and the corresponding duration will be provided.

7.1.7.2. Selected Study Drug Related ISRs

Selected study drug related ISRs include study drug related injection site induration, nodule, swelling, erythema, and pain.

The number and percentage of participants who experienced at least 1 selected study drug related ISR will be provided and summarized by SOC, PT and serverity for each SC injection and the overall.

For each PT term of the study drug related ISRs, percentage of ongoing and resolved events will be summarized for each SC injection (Day 1 SC, Week 26, Week 52, etc.) and the overall at both participant-level and event-level.

For the participant-level summary, if a participant had more than one events of the same PT term, the participant will be counted in the “Ongoing” category unless all events of that same PT term have been resolved.

For the event-level summary, duration of the resolved events will also be summarized using descriptive statistics.

A by-participant listing for study drug related injection site induration and nodules and the corresponding duration will be provided.

7.2. Laboratory Evaluations

Laboratory data collected during the study will be analyzed and summarized using both quantitative and qualitative methods. Summaries of laboratory data will be provided for the Safety Analysis Set and will include all available data at the time of the database snapshot for the interim analysis. The analysis will be based on values reported in conventional units. When values are below the LOQ, they will be listed as such, and the closest imputed value will be used for the purpose of calculating summary statistics as specified in Section 3.7 Hemolyzed test results will not be included in the analysis, but they will be listed in by-participant laboratory listings.

Calcium Corrected for Albumin

Calcium corrected for albumin will be calculated and summarized for the study. The following formula will be used when both serum calcium and albumin results for a given blood drawn are available and serum albumin value is < 4.0 g/dL.

Calcium corrected for albumin (mg/dL) = serum calcium (mg/dL) + 0.8 × (4.0 – albumin (g/dL))

Toxicity grading for calcium will be applied based on the corrected values.

Estimated Glomerular Filtration Rate

The following formulae will be used to calculate the estimated glomerular filtration rate using Cockcroft-Gault formula ($eGFR_{CG}$):

$eGFR_{CG}$ (mL/min) = [(140 – age (yrs)) × weight (kg) × (0.85 if female)] / (SCr (mg/dL) × 72),

where weight is total body mass in kilograms and SCr is serum creatinine.

A by-participant listing for laboratory test results will be provided by participant ID number and visit in chronological order for hematology, (urine) chemistry, and urinalysis separately. Values falling outside of the relevant reference range and/or having a severity grade of 1 or higher based on Division of AIDS (DAIDS) Table for Grading the Severity of Adult and Pediatric Adverse Events, Version 2.1 dated July 2017, will be flagged in the data listings, as appropriate.

No formal statistical testing is planned.

7.2.1. Summaries of Numeric Laboratory Results

Descriptive statistics will be provided by treatment group for white blood cells, neutrophils (absolute result and %), lymphocytes (absolute result and %), creatinine, estimated glomerular filtration rate (eGFR) by CG, albumin, alkaline phosphatase, alanine amino transferase (ALT), aspartate amino transferase (AST), total bilirubin as follows:

- Baseline values
- Values at each postbaseline visit
- Change from baseline at each postbaseline visit

A baseline laboratory value will be defined as the last measurement obtained on or prior to the date of first dose of study drug. Change from baseline to a postbaseline visit will be defined as the visit value minus the baseline value. The mean, median, Q1, Q3, minimum, and maximum values will be displayed to the reported number of digits; SD values will be displayed to the reported number of digits plus 1.

Median (Q1, Q3) of the change from baseline values for the above laboratory tests (except for % neutrophils and lymphocytes) will be plotted using a line plot by cohort and treatment group and visit.

In the case of multiple values in an analysis window, data will be selected for analysis as described in Section 3.8.3.

7.2.2. Graded Laboratory Values

The DAIDS Table for Grading the Severity of Adult and Pediatric Adverse Events, Version 2.1 dated July 2017, will be used to assign toxicity grades (0 to 4) to laboratory results for analysis. Grade 0 includes all values that do not meet the criteria for an abnormality of at least Grade 1. For laboratory tests with criteria for both increased and decreased levels, analyses for each direction (ie, increased, decreased) will be presented separately.

7.2.2.1. Treatment-Emergent Laboratory Abnormalities

Treatment-emergent laboratory abnormalities are defined as values that increase at least 1 toxicity grade from baseline at any postbaseline visit (and up to 60 days after Last Dose Date of Oral Bridging if participants discontinue from the oral bridging of LEN). If the relevant baseline laboratory value is missing, any abnormality of at least Grade 1 observed at any postbaseline visit will be considered treatment emergent.

7.2.2.2. Summaries of Laboratory Abnormalities

The summaries (number and percentage of participants) for treatment-emergent laboratory abnormalities and Grade 3 or 4 laboratory abnormalities will be provided by lab test; participants will be categorized according to the most severe postbaseline abnormality grade for a given lab test.

Graded laboratory abnormalities and Grade 3 or 4 laboratory abnormality will be summarized for All LEN Analysis. For all summaries of laboratory abnormalities, the denominator is the number of participants with nonmissing postbaseline values.

A by-participant listing of treatment-emergent laboratory abnormalities and treatment-emergent Grade 3 or 4 laboratory abnormalities will be provided by participant ID number and visit in chronological order. This listing will include all test results that were collected throughout the study for the lab test of interest, with all applicable severity grades displayed.

7.3. Body Weight and Vital Signs

Descriptive statistics will be provided for All LEN Analysis for body weight, BMI, and vital signs as follows:

- Baseline value
- Values at each postbaseline visit
- Change from baseline at each postbaseline visit

A baseline value will be defined as the last available value collected on or prior to the date of first dose of study drug. Change from baseline to a postbaseline visit will be defined as the postbaseline value minus the baseline value.

In the case of multiple values in an analysis window, data will be selected for analysis as described in Section 3.8.3. No formal statistical testing is planned.

A by-participant listing of vital signs will be provided by participant ID number and visit in chronological order. Body weight, height, and BMI will be included in the vital signs listing, if space permits. If not, they will be provided separately.

7.4. Antiretroviral Medications and Concomitant Medications

7.4.1. Antiretroviral Medications

The ARV medications are defined as nonstudy drug ARV medications used prior to, during, or after the study (if collected). The ARV medications are recorded on the ARV eCRF and will be coded using the World Health Organization (WHO) Drug Dictionary for ARV medications. The WHO preferred name and drug code will be attached to the clinical database. All ARV medications recorded on the ARV eCRF will be listed.

7.4.1.1. Prior Antiretroviral Medications

Prior ARV medications are defined as any nonstudy drug ARV medications with a start date prior to the first dose date of study drug regardless of when the stop date is. If a partial start date is entered, the medication will be considered prior unless the month and year (if day is missing) or year (if day and month are missing) of the start date are after the first dose date. Medications with a completely missing start date will be included in the prior medication summary, unless otherwise specified.

Prior ARV medications by individual agent will be summarized by drug class and preferred name (based on the WHO coding terms) using the number and percentage of participants for each cohort and treatment group. A participant reporting the same medication more than once will be counted only once within each drug class when calculating the number and percentage of participants who received that medication. The summary will be ordered alphabetically by drug class and then by preferred term in descending order of the total frequency. For drugs with the same frequency, sorting will be done alphabetically.

In addition, number of prior ARVs by individual (excluding COBI and RTV used as a boosting agents) and prior ARV drug class exposure will be summarized in the baseline characteristics table mentioned in Section 5.2.

7.4.1.2. Antiretroviral Medications as Failing Regimen

The ARV medications as failing regimen is defined as any nonstudy drug ARV medications that are used on Day -1, the day before the first dose date of study drug. The failing regimen will be summarized in the same manner as the prior ARV medications by drug class and preferred term. In addition, the number of failing regimens by individual agent and failing regimens by drug class (excluding COBI and RTV used as a boosting agents) will be included in the baseline characteristics table mentioned in Section 5.2.

The GSS, PSS, and OSS will be calculated for failing regimen by summing up the available sensitivity/susceptibility scores from all drugs in the failing regimen based on resistance assessments from genotypic (GSS) testing and phenotypic (PSS) testing, and the overall assessments from both tests (OSS). The details of the calculation are available in the programming specification (Appendix 1).

7.4.1.3. Optimized Background Regimen at Baseline

The OBR at baseline is defined as any nonstudy drug ARV medications that were started on or prior to Study Day 28 from the first dose date of open-label LEN and were used for a minimum duration of 28 days on or after the first dose date of open-label LEN. The ARV medications taken on and after the open-label first dose date with temporary interruption will be considered as the same ARV. The earliest start date and the latest end date of the same ARV will be used in the calculation to define baseline OBR. The OBR by individual agent will be summarized in the same manner as the prior ARV medications by drug class and preferred term. The number of OBR by individual agent and OBR by drug class (excluding COBI and RTV used as a boosting agents) will be summarized for All LEN Analysis.

A listing of participants with OBR change during the study will be provided. The OBR change during the study includes:

1) Added new ARV that is not part of the baseline OBR;

2) Stopped any ARV that is part of the OBR;

3) Any interruption of ARV that is part of the OBR during the study

The listing format will be the same as the listing of non-study drug ARV medication, with only the participants with OBR change included.

In addition, the number of OBR by individual agent and OBR by drug class (excluding COBI and RTV used as a boosting agents) will be included in the baseline characteristics table mentioned in Section 5.2.

The GSS, PSS, and OSS will be calculated for OBR at baseline by summing up the available sensitivity/susceptibility scores from all drugs in the baseline OBR based on resistance assessments from genotypic (GSS) testing and phenotypic (PSS) testing, and the overall assessments from both tests (OSS). The details of the calculation are available in the programming specification (Appendix 1).

7.4.2. Concomitant Medications

Concomitant medications are defined as non-ARV medications taken on and after first dose date of study drug. Use of concomitant medications will be summarized by preferred name using the number and percentage of participants and cohort based on the Safety Analysis Set in All LEN Analysis. A participant reporting the same medication more than once will be counted only once when calculating the number and percentage of participants who received that medication. The summary will be ordered by preferred term in descending order of the overall frequency. For drugs with the same frequency, sorting will be done alphabetically.

For the purposes of analysis, any medications with a start date prior to or on the first dose date of study drug and continued to be taken after the first dose date, or started after the first dose date will be considered concomitant medications. Medications started and stopped on the same day as the first dose date will also be considered concomitant. Medications with a stop date prior to the date of first dose date of study drug will be excluded from the concomitant medication summary. If a partial stop date is entered, any medication with the month and year (if day is missing) or year (if day and month are missing) prior to the date of first study drug administration will be excluded from the concomitant medication summary. If a partial start date is entered, any medication with the month and year (if day is missing) or year (if day and month are missing) after the study drug stop date will be excluded from the concomitant medication summary. Medications with completely missing start and stop dates will be included in the concomitant medication summary, unless otherwise specified.

A by-participant listing for all concomitant medications will be listed.

7.5. Electrocardiogram Results

7.5.1. Investigator Electrocardiogram Assessment

The investigators' assessment of ECG results (normal; abnormal, not clinically significant; abnormal, clinically significant) are collected at screening only. A by-participant listing for ECG assessment results will be provided by participant ID number.

7.6. Other Safety Measures

A data listing will be provided for participants experiencing pregnancy and participants using/misusing any substances (eg, illicit drug) during the study, respectively. Physical examination was not collected in the eCRF. Therefore, it will not be included in the analysis.

7.7. Participant Subgroup for Safety Endpoints

Incidence of all treatment-emergent AEs will be repeated within each subgroup defined in Section 3.4.2 using the Safety Analysis set for All LEN Analysis. Due to small number of participants available in each subgroup, results will be descriptive. No formal statistical testing is planned.

7.8. Changes from Protocol-Specified Safety Analyses

No change from protocol-specified safety analyses is planned.

8. PHARMACOKINETIC (PK) ANALYSES

No PK analysis is planned.

9. REFERENCES

Emu B, Fessel J, Schrader S, Kumar P, Richmond G, Win S, et al. Phase 3 Study of Ibalizumab for Multidrug-Resistant HIV-1. N Engl J Med 2018;379 (7):645-54.

U. S. Department of Health and Human Services, Food and Drug Administration (FDA), Center for Drug Evaluation and Research (CDER). Human Immunodeficiency Virus-1 Infection: Developing Antiretroviral Drugs for Treatment. Guidance for Industry. Silver Spring, MD. November, 2015.

10. SOFTWARE

SAS® Software Version 9.4. SAS Institute Inc., Cary, NC, USA.

nQuery Advisor® Version 7.0. Statistical Solutions, Cork, Ireland.

Phoenix WinNonlin® 7.0 Pharsigh Corporation, Princeton, NJ, USA.

11. SAP REVISION

Revision Date (DD MMM YYYY)	Section	Summary of Revision	Reason for Revision

12. APPENDICES

Appendix 1. Schedule of Assessments
Appendix 2. Flowchart of US FDA-defined Snapshot Algorithm
Appendix 3. Programming Specifications

Appendix 1. Schedule of Assessments

	Screening[a]	Cohort Selection[b]	Day 1[c]	Day 2	Day 5	Day 8	Day 15[d]	Day 16[d]	Day 19[d]	Day 22[d]	Day 1 SC	Weeks 4, 10, 16, 22, 26, 36	Week 52 and Visits Thereafter[e]	Oral Bridging Visits[f]	30, 90, and 180 Day Follow-Up[g]	Early Termination[h]
Written Informed Consent/Assent/Parental Consent	X															
Medical History	X															
Demographic Information	X															
Complete Physical Examination	X		X				X				X	X[i]	X[i]			X
Symptom Directed Physical Examination				X		X		X		X		X[i]	X[i]	X	X	
Vital Signs[j](include weight)	X		X	X		X	X	X		X	X	X	X	X	X	X
12-lead ECG (supine)	X															
Height	X															
Hematology[k], Chemistry[l], Estimated GFR, Urinalysis[m], Urine Chemistry[m], CD4+ Cell Count	X		X			X	X			X	X	X	X	X	X	X
Urine Storage Sample			X	X		X	X	X		X	X	X	X	X		X
Serum Pregnancy Test[n]	X															
Serum FSH[o]	X															
Urine Pregnancy Test[n]			X			X	X			X	X	X	X	X	X	X
HBV, HCV Testing	X															
HIV-1 Genotyping/Phenotyping	X															

	Screening[a]	Cohort Selection[b]	Day 1[c]	Day 2	Day 5	Day 8	Day 15[d]	Day 16[d]	Day 19[d]	Day 22[d]	Day 1 SC	Weeks 4, 10, 16, 22, 26, 36	Week 52 and Visits Thereafter[e]	Oral Bridging Visits[f]	30, 90, and 180 Day Follow-Up[g]	Early Termination[h]
Plasma HIV-1 RNA	X	X	X	X		X	X	X		X	X	X	X	X	X	X
Plasma Storage Sample	X	X	X	X		X	X	X		X	X	X	X	X	X	X
Intensive PK Plasma Collection[p]			X				X									
Single Timed, Single Anytime and/or Pre-Dose PK Plasma Sample[q]				X	X	X		X	X	X	X	X[r,s]	X	X		
Optional Whole Blood Sample for PG[t]			X													
Oral GS-6207 Administration[u]			X	X		X	X	X		X				X		
Begin Optimized Background Regimen[v]			X				X				X					
SC GS-6207 Administration[w]											X	X	X			
Symptoms Distress Module, SF-36, EQ-5D-5L[x]			X									X	X			
Numeric Pain Scale[y]											X	X	X			
Injection Site Reaction Worksheet[z]											X	X	X			
Adverse Events/ Concomitant Meds	X	X	X	X	X	X	X	X	X	X	X	X	X	X	X	X

	Screening[a]	Cohort Selection[b]	Day 1[c]	Day 2	Day 5	Day 8	Day 15[d]	Day 16[d]	Day 19[d]	Day 22[d]	Day 1 SC	Weeks 4, 10, 16, 22, 26, 36	Week 52 and Visits Thereafter[e]	Oral Bridging Visits[f]	30, 90, and 180 Day Follow-Up[g]	Early Termination[h]
Written Informed Consent/Assent/Parental Consent	X															
Medical History	X															
Demographic Information	X															
Complete Physical Examination	X		X				X				X	X[i]	X[i]			X
Symptom Directed Physical Examination				X		X		X		X		X[i]	X[i]	X	X	
Vital Signs[j](include weight)	X		X	X		X	X	X		X	X	X	X	X	X	X
12-lead ECG (supine)	X															
Height	X															
Hematology[k], Chemistry[l], Estimated GFR, Urinalysis[m], Urine Chemistry[m], CD4+ Cell Count	X		X			X	X			X	X	X	X	X	X	X
Urine Storage Sample			X	X		X	X	X		X	X	X	X	X		X
Serum Pregnancy Test[n]	X															
Serum FSH[o]	X															
Urine Pregnancy Test[n]			X			X	X			X	X	X	X	X	X	X
HBV, HCV Testing	X															
HIV-1 Genotyping/Phenotyping	X															
Plasma HIV-1 RNA	X	X	X	X		X	X	X		X	X	X	X	X	X	X
Plasma Storage Sample	X	X	X	X		X	X	X		X	X	X	X	X	X	X

	Screening[a]	Cohort Selection[b]	Day 1[c]	Day 2	Day 5	Day 8	Day 15[d]	Day 16[d]	Day 19[d]	Day 22[d]	Day 1 SC	Weeks 4, 10, 16, 22, 26, 36	Week 52 and Visits Thereafter[e]	Oral Bridging Visits[f]	30, 90, and 180 Day Follow-Up[g]	Early Termination[h]
Intensive PK Plasma Collection[p]			X				X									
Single Timed, Single Anytime and/or Pre-Dose PK Plasma Sample[q]				X	X	X		X	X	X	X	X[r,s]	X	X		
Optional Whole Blood Sample for PG[t]			X													
Oral GS-6207 Administration[u]			X	X		X	X	X		X				X		
Begin Optimized Background Regimen[v]			X				X				X					
SC GS-6207 Administration[w]											X	X	X			
Symptoms Distress Module, SF-36, EQ-5D-5L[x]			X									X	X			
Numeric Pain Scale[y]											X	X	X			
Injection Site Reaction Worksheet[z]											X	X	X			
Adverse Events/ Concomitant Meds	X	X	X	X	X	X	X	X	**X**	X	X	X	X	X	X	X

a Screening evaluations must be completed within 42 days prior to Day 1.
b Cohort Selection visit to be completed 14 to 30 days after the Screening visit until Cohort 1 is fully enrolled.
c Day 1 tests and procedures must be completed prior to study drug administration.
d Day 15, 16, 19 and 22 visits will be completed by Cohort 1B participants only.
e At the Week 52 visit, participants will be given an option to attend visits at Week 62, 78, 88, 104, 114, 130 and will continue to alternate between every 10 weeks and every 16 weeks. Participants will receive SC GS-6207 every 6 months (26 weeks) starting at Week 52 visit, while continuing their OBR, until the product becomes accessible to participants through an access program or until Gilead Sciences elects to discontinue the study in the country.
f Only applicable to participants who require oral weekly bridging if an SC injection of GS-6207 cannot be administered for any reason within the protocol visit window.
g Participants may be required to return to the clinic for a 30, 90 and 180-Day Follow-Up visit after Early Termination visit as noted in section 6.4.1.

h Early Termination visit to be completed, if participant decides to discontinue study drug prior to completing Week 52 visit or prior to study completion. Investigators should counsel participant regarding the importance of continuing a complete ARV therapy in accordance to standard of care, and refer participant to an appropriate HIV treatment facility.
i Complete physical examination to be completed at Weeks 26 and 52, symptom-directed physical examination to be completed at all other visits.
j Vital signs – blood pressure, pulse, respiration rate, and temperature, weight.
k Hematology: CBC with differential and platelet count.
l Chemistries: alkaline phosphatase, AST, ALT, GGT, total bilirubin, direct and indirect bilirubin, total protein, albumin, LDH, CPK, bicarbonate, BUN, calcium, chloride, creatinine, glucose, lipase, magnesium, phosphorus, potassium, sodium, uric acid (except at Cohort Selection, Days 2, 5, 16 and 19 visits).
m Urinalysis and Urine Chemistry: including color & clarity, specific gravity, pH, glucose, ketones, bilirubin, urobilinogen, blood, nitrite, leukocyte esterase and microscopic (if microscopic elements are seen), urine protein, albumin, creatinine, phosphate, calcium, magnesium and uric acid (except at Cohort Selection, Days 2, 5, 16 and 19 visits).
n All women will have a serum test performed at Screening. Urine pregnancy test will be performed at all subsequent visits for women of childbearing potential (except at Days 2, 5, 16 and 19). Positive urine pregnancy tests will be confirmed with a serum test.
o FSH test is required for women who are < 54 years old and have stopped menstruating for ≥ 12 months but do not have documentation of ovarian hormonal failure.
p Day 1 (for all participants) and Day 15 (for Cohort 1B participants only): 0 (predose, ≤ 30 min before dose), 1, 2, 4, 6 and 8 hours post dose.
q Days 2, 8, 16, and 22: A pre-dose sample (within 30 minutes of dosing) and a single timed PK samples between 1 and 6 hours post dose. Days 5 and 19 (if the visits are completed): a single anytime PK sample.
r During the Maintenance Period, at all visits without SC GS-6207 injections, including the Oral Bridging visits, if applicable: A single anytime PK sample will be collected.
s During the Maintenance Period, at all visits with SC GS-6207 injections: A single pre-dose (within 30 minutes of dosing) PK sample will be collected (while participants are continuing to receive study drug).
t If consent for pharmacogenomic testing is obtained, then a sample will be collected for Optional Pharmacogenomic testing. This sample should be collected at the Day 1 visit, but may be collected at any time during the study or at a separate post study visit, if necessary.
u Cohort 1 participants will be administered oral GS-6207 or placebo to match GS-6207 at Days, 1, 2 and 8. Participants who receive placebo to match GS-6207 at Days 1, 2 and 8 will receive oral GS-6207 at Days 15, 16 and 22. Cohort 2 participants will be administered oral GS-6207 at Days 1, 2 and 8. Participants requiring oral bridging will be administered oral GS-6207 per Section 5.4, as applicable.
v Cohort 1 participants will begin an OBR on Day 15 (Cohort 1B) or Day 1 SC (Cohort 1A). Cohort 2 participants will begin an OBR on Day 1. An OBR should be selected based on the screening and/or available historical HIV resistance reports.
w All participants will be administered SC GS-6207 at Day 1 SC (14 days after the first dose of oral GS-6207) and will continue to receive SC GS-6207 every 6 months (26 weeks). Each SC GS-6207 dosing should occur within 26 and 28 weeks of the previous SC GS-6207 dosing.
x Participants ≥ 18 years of age at Day 1 visit will complete Symptoms Distress Module, SF-36, EQ-5D-5L at Day 1, Weeks 4, 16, 26 and 52, if available (before completing other study procedures).
y Participants ≥ 18 years of age at Day 1 visit will complete the Numeric Pain Rating Scale at Day 1 SC, Weeks 26 and 52 (after they receive SC GS-6207 injections).
z Provide injection site reaction assessment worksheet and instruct the participants to measure and report injection site reactions following the administration of the SC injections.

Appendix 2. Flowchart of US FDA-defined Snapshot Algorithm

The following flowchart for US FDA-defined snapshot algorithm is based on the US FDA Guidance on Human Immunodeficiency Virus-1 Infection: Developing Antiretroviral Drugs for Treatment {U. S. Department of Health and Human Services 2015}

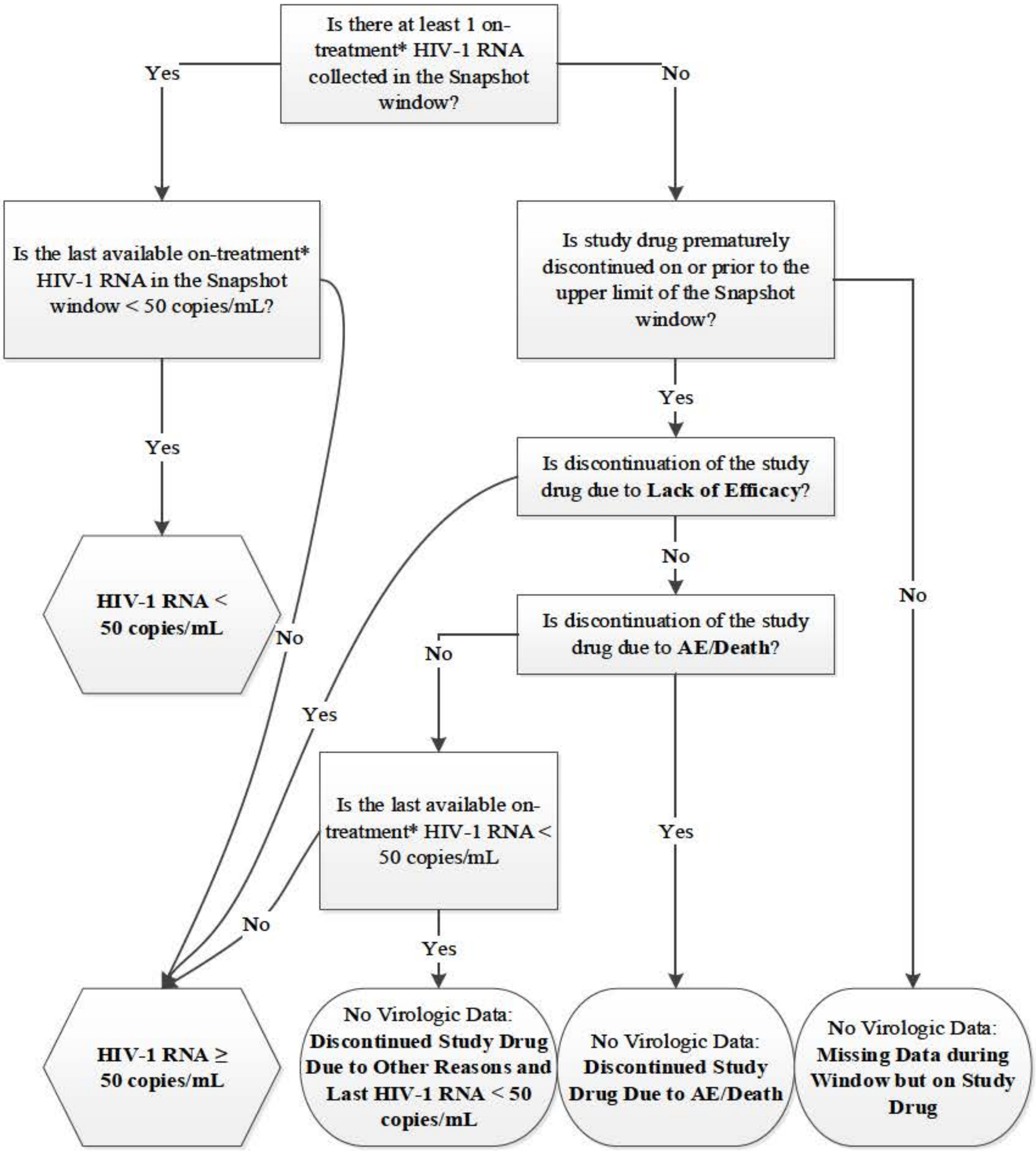

* The definition of on-treatment HIV-1 RNA is specified in Section 6.2.1.

Appendix 3. Programming Specifications

1) Study drug disposition in Main Phase

   — Oral lead-in before the first SC injection

     - Completed study drug: participants who received SC LEN injection on Day 1 SC.
     - Continuing study drug: participants who did not receive SC LEN on Day 1 SC and Study Drug Completion eCRF for the Study Phase = “Treatment” has not been filled out.
     - Prematurely discontinued study drug with reasons: participants who did not receive SC LEN injection on Day 1 SC and whose Study Drug Completion eCRF for the Study Phase = “Treatment” was marked as “No”.

   — After the first SC injection

     - Received Day 1 SC injection.
     - Continuing study drug: participants who received Day 1 SC injection and Study Drug Completion eCRF for the Study Phase = “Treatment” has not been filled out.
     - Completed study drug: participants who received Day 1 SC injection and whose Study Drug Completion eCRF for the Study Phase = “Treatment” was marked as “Yes”.
     - Prematurely discontinued study drug with reasons: participants who received Day 1 SC injection and whose Study Drug Completion eCRF for the Study Phase = “Treatment” was marked as “No”.

2) Number of years since HIV diagnosis is calculated as year of the first dose date minus year of HIV diagnosis. If number of years is great than age, reset number of years = age. Number of years since first started HIV treatment is calculated similarly.

3) Drug class and abbreviation of the generic name is tabulated below:

Drug Class	Generic Name	Generic Name Abbreviation
NRTI	Abacavir	ABC
	Zidovudine	AZT or ZDV
	Didanosine	ddI
	Stavudine	d4T
	Emtricitabine	FTC
	Tenofovir alafenamide	TAF
	Tenofovir Disoproxil Fumarate	TDF
	Zalcitabine	ddC
	Lamivudine	3TC

Drug Class	Generic Name	Generic Name Abbreviation
NNRTI	Delavirdine	DLV
	Doravirine	DOR
	Efavirenz	EFV
	Etravirine	ETR
	Nevirapine	NVP
	Rilpivirine	RPV
PI	Amprenavir	APV
	Atazanavir	ATV
	Darunavir	DRV
	Indinavir	IDV
	Fosamprenavir	FPV
	Lopinavir	LPV
	Nelfinavir	NFV
	Ritonavir	RTV or /r when used with other PI [1]
	Saquinavir	SQV
	Tipranavir	TPV
INSTI	Bictegravir	BIC
	Dolutegravir	DTG
	Elvitegravir	EVG
	Raltegravir	RAL
Fusion Inhibitor	Enfuvirtide	T20
CCR5 co-receptor Antagonist	Maraviroc	MVC
CD4-Directed Post Attachment	Ibalizumab	IMAB
Attachment Inhibitor (AI)	Fostemsavir	FTR
Pharmacokinetic Enhancers	Cobicistat	COBI or /c when used with PI[2]
Other	for any ARVs not meeting any of above drug classes	

1. RTV is not considered as an ARV when used as a boosting agent, defined as RTV used as 100 mg QD or BID or as 200 mg QD or BID.
2. COBI is not an ARV.

4) Study drug administration: Oral reload will be recorded as an “Unscheduled” visit in the Study Drug Administration eCRF. participants with oral reload prior to the 1st dose of SC have “Unscheduled” dosing visits occurred prior to Day 1 SC visit. Participants who received a total of 5 tablets of LEN (300 mg) (ie, 2 x 300 mg LEN tablets each for the first 2 days and 1 x 300 mg LEN tablet on the 8th day) prior to “Unscheduled” dosing visits followed by Day 1 SC visit will be classified as oral reload due to missing scheduled SC dosing. Otherwise, participants will be classified as oral reload due to missing scheduled oral dosing visit.

5) GSS, PSS, and OSS calculation for the baseline OBR:

   For participants enrolled based on historical genotypic reports, only GSS data for individual drugs are available, and the OSS is the same as the GSS. For participants enrolled based on the Screening report, GSS, PSS, and OSS data for individual drugs are provided in the Screening report. On occasions, drug scores for some participants combined scores from both historical report and Screening report. GSS, PSS, and OSS for the failing regimen and baseline OBR are calculated by summing up the scores from individual drugs in the regimens. GSS, PSS, and OSS data are stored in the Virology Database (GVD, Gilead Virology Database).

6) ARV definitions:

   a) Prior ARV: For an ARV with ARV.CMSCAT = "Prior ARV", if the end date of the ARV is missing and not marked as ongoing, the ARV is considered ended prior to the 1st dose date.

7) Injection site reaction:

   a) To summarize ISRs by injection visit, each study drug related ISR will be associated with one injection visit (eg, Day 1 SC injection, Week 26 SC Injection, Week 52 SC injection) based on the start date of the ISR. If the start date of the ISR is on or after a given injection visit date and prior to the next injection visit date, if available, the ISR will be associated with that injection visit.

   b) For ISR summarized by PT:

      i) For the overall summary (ie, participants received at least one injection), multiple ISRs with the same PT will only be counted once per participant for each PT.

      ii) For by visit summary (ie, participants received injection for a given injection visit), multiple ISRs associated with the injection visit of interest with the same PT will only be counted once per participant for each PT.

   c) For ISR summarized by grade:

      i) For the overall summary (ie, participants received at least one injection), the most severe grade based on all ISRs will be used.

      ii) For by visit summary (participants received injection for a given injection visit), the most severe grade from all ISRs associated with the injection visit of interest will be used.

8) For the proportion of participants with HIV-1 RNA < 50 and < 200 copies/mL using the US FDA-defined snapshot algorithm and its subgroup analysis, and for HIV-1 RNA Missing = Failure, 'have not reached the upper limit of that analysis window for the corresponding visit' refers to: data finalization date – First Dose Date (for All LEN Analysis) + 1 < the upper limit of the analysis window for a visit.

9) For completely or partially missing Last Dose Date (for All LEN Analysis, for the study, or of Oral Bridging), the latest date among the study drug start date and end date, clinical visit date, laboratory sample collection date, and vital signs assessment date that occurred during the oral bridging period will be used for calculating the study day on listings. If month and year of the last dose are known, and the last study drug dosing date imputed above is different from the month collected, the last date of that month will be used. If only year of the last dose is known, and the last study drug dosing date imputed above is after the year collected, the last date of that year will be used; if the last study drug dosing date imputed above is before the year collected, the first date of that year will be used.

GS-US-200-4625-Year 2 & 3-SAP

ELECTRONIC SIGNATURES

Signed by	Meaning of Signature	Server Date (dd-MMM-yyyy hh:mm:ss)
PPD	Clinical Development eSigned	12-Apr-2023 22:22:28
PPD	Biostatistics eSigned	13-Apr-2023 18:53:35
