# Supplementary material for: Subcutaneous Lenacapavir in People With Multidrug-Resistant HIV-1: 156 Week Results of the CAPELLA Study
Source: Open Forum Infect Dis. 2025 Dec 19;13(1):ofaf763. doi: 10.1093/ofid/ofaf763 (PMC12740718; doi:10.1093/ofid/ofaf763)
Supplement: ofaf763_Supplementary_Data [file ofaf763_supplementary_data.zip › Ogbuagu_CAPELLA 3 yr analysis appendix.docx]

**Supplementary Appendix**

Ogbuagu O, et al. Subcutaneous Lenacapavir in People with Multidrug-Resistant HIV-1: 156 Week Results of the CAPELLA Study

**GS-US-400-4265 Investigators**

**United States**

Edwin DeJesus

Gary J Richmond

Mezgebe Berhe

Peter J Ruane

Gary I Sinclair

Kenneth Lichtenstein

Moti N Ramgopal

Andrew Wiznia

Kimberly Workowski

William Sanchez

Cynthia Brinson

Joseph P McGowan

Catherine M Creticos

Daniel S Berger

David A Wheeler

Debbie Hagins

Gordon E Crofoot

James Sims

Olayemi Osiyemi

Theo Hodge

Christine Zurawski

Onyema Ogbuagu

Sorana Segal-Maurer

**Thailand**

Winai Ratanasuwan

Anchalee Avihingsanon

Krittaecho Siripassorn

Ploenchan Chetchotisakd

**Italy**

Antonella Castagna

Andrea Antinori

Francesco Castelli

**France**

Sylvie Ronot-Bregigeon

Jean-Michel Molina

Yazdan Yazdanpanah

**Canada**

Benoit Trottier

Jason Brunetta

**Japan**

Takuma Shirasaka

Yoshiyuki Yokomaku

**Dominican Republic**

Ellen Koenig

**Spain**

Josep Mallolas

**Germany**

Hans-Jurgen Stellbrink

**Taiwan**

Chien-Ching Hung

**South Africa**

Mohammed Rassool

# Inclusion and Exclusion Criteria

***To be eligible for study participation, all of the following inclusion criteria had to be met:***

1) Willing and able to provide written informed consent (participants ≥18 years of age) and assent (participants ≥12 and <18 years of age) prior to performing study procedures. For participants ≥12 and <18 years of age, parent or legal guardian willing and able to provide written informed consent prior to performing study procedures as required by local law

2) Adult aged ≥18 years (at all sites) or adolescent aged ≥12 years and weighing ≥35 kg (at sites in North America and Dominican Republic)

3) Are receiving a stable failing antiretroviral (ARV) regimen for >8 weeks before screening and willing to continue the regimen until Day 1. Participants in Cohort 1 must also be willing to continue the failing regimen until completing the Functional Monotherapy Period (Day 1 to Day 14)

4) Have HIV-1 RNA ≥400 copies/mL at screening

5) Have screening or available historical HIV-1 resistance reports showing resistance to

≥2 ARV medications from each of ≥3 of the four main classes of antiretroviral medications (nucleoside reverse transcriptase inhibitors, non-nucleoside reverse transcriptase inhibitors, protease inhibitors, integrase strand transfer inhibitor). Resistance to emtricitabine or lamivudine associated with the presence of the M184V/I reverse transcriptase mutation cannot be used for the purpose of determining this eligibility criterion

6) Have ≤2 fully active ARV(s) remaining from the four main classes that can be effectively

combined to form a viable regimen in the opinion of the investigator based on resistance,

tolerability, contraindication, safety, drug access, or acceptability to the participant

7) Able and willing to receive an optimized background regimen (OBR) together with lenacapavir. Participants with an OBR without a fully active agent may be enrolled if the investigator considers that there is a favorable risk-benefit ratio for the participant. With prior approval from Gilead Sciences, Inc., components of the OBR may be investigational (i.e., not yet approved)

8) A negative serum pregnancy test is required for all women at screening

9) Participants of childbearing potential who engage in heterosexual intercourse must agree to use protocol-specified method(s) of contraception

10) Lactating women must agree to discontinue nursing before administration of lenacapavir

***Individuals who meet any of the following exclusion criteria were not to be enrolled:***

1) An opportunistic illness requiring acute therapy within the 30 days prior to screening

2) Active, serious infections (other than HIV-1 infection) requiring parenteral antibiotic or antifungal therapy within 30 days before screening

3) Active tuberculosis infection

4) Acute hepatitis within 30 days prior to screening visit

5) Untreated or newly treated (<3 months prior to screening) hepatitis B virus (HBV)

infection. Participants may be enrolled regardless of the HBV serology criteria below if they are receiving treatment with anti-HBV activity and plan to continue the treatment during the study. HBV infection is defined as screening results showing either or both of:

a. Positive HBV surface antigen

b. Positive HBV core antibody and negative HBV surface antibody. Participants may be enrolled with these findings if they have HBV DNA < lower limit of quantification

6) Hepatitis C virus (HCV) antibody positive and HCV RNA > lower limit of quantification

7) A history of or current clinical decompensated liver cirrhosis (e.g., ascites, encephalopathy, or variceal bleeding)

8) Treatment within three months prior to screening, or anticipated treatment during the study period, with immunosuppressant therapies, hydroxyurea, foscarnet, radiation, or cytotoxic chemotherapeutic agents without prior approval from Sponsor prior to randomization

9) Active malignancy requiring acute therapy (with the exception of local cutaneous Kaposi's sarcoma)

10) Current alcohol or substance use judged by the investigator to potentially interfere with the participant’s study compliance

11) Clinically significant abnormal electrocardiogram at the screening visit

12) Any of the following laboratory values at screening:

a. Estimated glomerular filtration rate ≤50 mL/min using Cockcroft-Gault formula for participants ≥18 years of age and Schwartz Formula for participants <18 years of age for creatinine clearance

b. Alanine transaminase >5 x upper limit of normal

c. Direct bilirubin >1.5 x upper limit of normal

d. Platelets <50,000/mm^3^

e. Hemoglobin <8.0 g/dL

13) Participation or planned participation in any other clinical trial (including observational trials) without prior approval from the Sponsor throughout the study

14) Prior use of, or exposure to, lenacapavir

15) Known hypersensitivity to the investigational medicinal product, the metabolites, or formulation excipient

16) Use or planned use of exclusionary medications

17) Any other clinical condition or prior therapy that, in the opinion of the investigator, would make the participant unsuitable for the study or unable to comply with dosing requirement

# Supplementary Table 1. Participant Demographics and Baseline Characteristics

|  | **Cohort 1** | | | **Cohort 2** | **Total  (n=72)** |
| --- | --- | --- | --- | --- | --- |
|  | **Lenacapavir (n=24)** | **Placebo* (n=12)** | **Total  (n=36)** | **Lenacapavir (n=36)** |  |
| Median (IQR) age, years | 55 (50–61) | 54 (49–55) | 54 (49–59) | 49 (38–60) | 52 (45–59) |
| Sex at birth | | | | | |
| Male | 17 (71) | 9 (75) | 26 (72) | 28 (78) | 54 (75) |
| Female | 7 (29) | 3 (25) | 10 (28) | 8 (22) | 18 (25) |
| Gender identity | | | | | |
| Cisgender | 24 (100) | 10 (83) | 34 (94) | 33 (92) | 67 (93) |
| Transgender | 0 | 0 | 0 | 1 (3) | 1 (1) |
| Non-binary or third gender | 0 | 1 (8) | 1 (3) | 0 | 1 (1) |
| Other | 0 | 0 | 0 | 1 (3) | 1 (1) |
| Prefer not to disclose | 0 | 1 (8) | 1 (3) | 1 (3) | 2 (3) |
| Race | | | | | |
| Black | 10 (42) | 6 (55) | 16 (46) | 11 (31) | 27/71 (38) |
| White | 12 (50) | 4 (36) | 16 (46) | 13 (36) | 29/71 (41) |
| Asian | 2 (8) | 1 (9) | 3 (9) | 12 (33) | 15/71 (21) |
| Not permitted | 0 | 1 (9) | 1 (3) | 0 | 1 (1) |
| Ethnicity, Hispanic or Latine | 6 (25) | 4 (36) | 10 (29) | 5 (14) | 15/71 (21) |
| HIV-1 viral load, log_10_ copies/mL^†^ | | | | | |
| Mean (SD) | 4.0 (0.92) | 4.9 (0.39) | 4.3 (0.89) | 4.1 (1.16) | 4.2 (1.03) |
| Median (IQR) | 4.2 (3.2–4.6) | 4.9 (4.5–5.3) | 4.5 (4.1–4.9) | 4.5 (3.3–4.9) | 4.5 (3.5–4.9) |
| Participants with HIV-1 RNA >100,000 copies/mL | 1 (4) | 6 (50) | 7 (19) | 7 (19) | 14 (19) |
| CD4 count, cells/μL | | | | | |
| Mean (SD) | 199 (166.1) | 85 (62.9) | 161 (149.5) | 258 (273.4) | 210 (224.2) |
| Median (IQR) | 172 (99–248) | 85 (39–109) | 127 (79–201) | 195 (56–392) | 150 (76–286) |
| CD4 count distribution, n (%) | | | | | |
| <50 cells/μL | 3 (13) | 4 (33) | 7 (19) | 9 (25) | 16 (22) |
| ≥50 to <200 cells/μL | 13 (54) | 7 (58) | 20 (56) | 10 (28) | 30 (42) |
| ≥200 to <500 cells/μL | 7 (29) | 1 (8) | 8 (22) | 12 (33) | 20 (28) |
| ≥500 cells/μL | 1 (4) | 0 | 1 (3) | 5 (14) | 6 (8) |
| Time since HIV diagnosis, years | | | | | |
| Mean (SD) | 27 (6.1) | 24 (5.5) | 26 (6.0) | 23 (7.1) | 25 (6.7) |
| Median (IQR) | 27 (23–32) | 26 (20–27) | 26 (22–31) | 23 (19–28) | 24 (21–29) |
| Time since first HIV-1 treatment, years | | | | | |
| Mean (SD) | 25 (4.9) | 22 (6.3) | 24 (5.5) | 19 (8.2) | 22 (7.2) |
| Median (IQR) | 25 (22–28) | 25 (19–26) | 25 (21–28) | 20 (13–26) | 23 (18–27) |
| Number of prior antiretroviral medications | | | | | |
| Mean (SD) | 11 (6.3) | 10 (6.0) | 11 (6.1) | 13 (5.6) | 12 (6.0) |
| Median (IQR) | 9 (8–16) | 9 (5–13) | 9 (7–14) | 13 (10–17) | 11 (8–16) |
| Resistance to two or more drugs in major class | | | | | |
| Nucleoside reverse transcriptase inhibitor | 23 (96) | 12 (100) | 35 (97) | 36 (100) | 71 (99) |
| Non-nucleoside reverse transcriptase inhibitor | 22 (92) | 12 (100) | 34 (94) | 36 (100) | 70 (97) |
| Protease inhibitor | 20 (83) | 8 (67) | 28 (78) | 30 (83) | 58 (81) |
| Integrase strand transfer inhibitor | 20 (83) | 7 (58) | 27 (75) | 23 (64) | 50 (69) |
| All four major classes | 14 (58) | 3 (25) | 17 (47) | 16 (44) | 33 (46) |
| Resistance to entry inhibitors^‡^ | | | | | |
| Enfuvirtide | 2/23 (9) | 3/10 (30) | 5/33 (15) | 0/25 | 5/58 (9) |
| Fostemsavir | 5/23 (22) | 5/10 (50) | 10/33 (30) | 7/21 (33) | 17/54 (31) |
| Ibalizumab | 8/23 (35) | 3/10 (30) | 11/33 (33) | 6/25 (24) | 17/58 (29) |
| Maraviroc^§^ | 19/24 (79) | 8/11 (73) | 27/35 (77) | 14/26 (54) | 41/61 (67) |
| Composition of OBR | | | | | |
| Nucleoside reverse transcriptase inhibitor | 23 (96) | 9 (75) | 32 (89) | 29 (81) | 61 (85) |
| Integrase strand transfer inhibitor^¶^ | 16 (67) | 9 (75) | 25 (69) | 21 (58) | 46 (64) |
| Protease inhibitor^¶^ | 12 (50) | 9 (75) | 21 (58) | 24 (67) | 45 (63) |
| Non-nucleoside reverse transcriptase inhibitor | 6 (25) | 5 (42) | 11 (31) | 14 (39) | 25 (35) |
| Ibalizumab | 9 (38) | 3 (25) | 12 (33) | 5 (14) | 17 (24) |
| Maraviroc | 2 (8) | 4 (33) | 6 (17) | 4 (11) | 10 (14) |
| Fostemsavir | 3 (13) | 0 | 3 (8) | 5 (14) | 8 (11) |
| Enfuvirtide | 1 (4.2) | 2 (17) | 3 (8) | 2 (6) | 5 (7) |
| Median overall susceptibility score of OBR^#^ | 2.0 | 1.5 | 2.0 | 2.0 | 2.0 |
| Number of fully active agents in the OBR | | | | | |
| 0 | 4 (17) | 2 (17) | 6 (17) | 6 (17) | 12 (17) |
| 1 | 7 (29) | 6 (50) | 13 (36) | 13 (36) | 26 (36) |
| ≥2 | 13 (54) | 4 (33) | 17 (47) | 17 (47) | 34 (47) |

Data are n (%) unless stated otherwise. Not permitted=local regulators did not allow collection of data on race or ethnicity. Percentages may not add up to 100 because of rounding.
*In Cohort 1, participants randomly assigned to placebo received it from Day 1 to 14; on Day 15, oral lenacapavir was initiated for two weeks, followed by subcutaneous injection on Day 29.

^†^Two participants in Cohort 2 had HIV-1 RNA >400 copies/mL at screening but <50 copies/mL at baseline.

^‡^All 72 participants were tested for entry inhibitor sensitivity; denominators refer to participants with available data for these drugs.

^§^Susceptibility to maraviroc was assessed using the Trofile® co-receptor tropism assay (Monogram Biosciences, San Francisco, CA, USA), which indicates whether the activity of maraviroc is expected based on the co-receptor usage (CCR5, CXCR4, or dual mix).

^¶^24 (33%) of 72 participants were taking dolutegravir twice a day and 22 (31%) of 72 boosted darunavir twice a day.

^#^Drug susceptibility scores (genotypic, phenotypic, and overall; 1 for full, 0.5 for partial, or 0 for no susceptibility) were determined based on a proprietary algorithm. For historical resistance reports, they were derived from data provided by investigators. The overall susceptibility score of the OBR was the sum of the individual scores.
IQR, interquartile range; OBR, optimized background regimen.

**Supplementary Table 2.** **Details of Participants in the Week 156 Resistance Analysis Population**

| **Participant ID** | **VF Visit*** | **HIV‑1 RNA (copies/mL)** | **Emergent Resistance-Associated Mutations** | | | |
| --- | --- | --- | --- | --- | --- | --- |
|  |  |  | **CA^†^** | **PR** | **RT** | **IN** |
| 1 | Oral Bridging | 2060 | Q67H | None | None | None |
|  | Week 88-R | 462 | ND | None | None | None |
|  | Week 130 | 69700 | Q67H K70R T107N | None | None | None |
| 2 | Week 26 | 561 | M66I | None | None | None |
|  | Week 52-R | 11400 | M66I N74D A105T | None | None | None |
| 3 | Week 10 | 2870 | M66I N74D A105T | None | None | None |
|  | Week 10-R | 5440 | M66I N74D A105T | None | None | None |
|  | Week 16 | 7510 | M66I N74D A105T | None | None | None |
|  | Week 22 | 206 | M66I Q67Q/H N74D A105T | None | None | None |
| 4 | Week 4 | 263 | AF | ND | ND | ND |
|  | Week 4-R | 293 | AF | None | None | None |
|  | Week 26 | 342 | None | AF | AF | None |
|  | Week 52 | 574 | AF | AF | AF | AF |
|  | Week 104 | 170 | AF | AF | AF | AF |
| 5 | Week 4 | 8380 | None | ND | ND | ND |
|  | Week 4-R | 6380 | None | None | None | AF |
|  | Week 22 | 4950 | None | ND | ND | ND |
|  | Week 52 | 3340 | None | None | None | None |
|  | Week 88 | 2090 | None | ND | ND | ND |
|  | Oral Bridging | 2640 | None | M46M/I I47I/V V82V/A | None | None |
|  | Week 156 | 654 | None | None | None | None |
| 6 | Week 4 | 137 | None | None | None | None |
|  | Week 4‑R | 111 | None | None | None | None |
|  | Week 26 | 164 | None | None | None | AF |
|  | Week 52 | 67 | None | I54I/L | None | AF |
|  | Week 156 | 347 | None | AF | AF | AF |
| 7 | Oral Bridging | 406000 | Q67H | M46I | None | None |
|  | Oral Bridging | 317 | Q67H | M46I | None | None |
|  | Week 88 | 220000 | Q67H K70R A105A/T | M46I | None | None |
| 8 | Week 22 | 97 | None | ND | ND | ND |
|  | Week 22‑R | 157 | None | AF | AF | AF |
| 9 | Oral Bridging | 895 | K70N N74K | None | None | None |
|  | Week 156 | 226 | K70N N74K T107T/N | None | None | None |
| 10 | Week 4 | 2150 | M66M/I | None | None | None |
|  | Week 26 | 2420 | M66M/I A105A/T | None | None | None |
|  | Week 52 | 2970 | M66I A105T | None | None | None |
|  | Week 104 | 7750 | M66I A105T | None | None | None |
| 11 | Week 4 | 186 | None | None | None | None |
|  | Week 26 | 81 | None | None | None | AF |
| 12 | Week 4 | 156 | None | AF | AF | AF |
| 13 | Week 4 | 260 | None | None | None | None |
|  | Week 26 | 91 | T107A | AF | AF | AF |
| 14 | Week 4 | 39000 | M66M/I | ND | ND | ND |
|  | Week 4-R | 25200 | M66M/I K70K/N/R/S | ND | ND | ND |
|  | Week 10 | 28900 | K70N/S N74N/H | None | None | None |
| 15 | Week 4 | 702 | None | None | None | None |
|  | Week 4-R | 1280 | None | None | None | None |
|  | Week 26 | 2350 | None | None | None | None |
|  | Week 52-R | 2630 | None | None | None | None |
|  | Oral Bridging | 1350 | AF | None | None | AF |
| 16 | Week 4 | 424 | None | None | K103N/S M184M/I/V | AF |
| 17 | Week 4 | 101000 | M66M/I Q67Q/H/K/N | AF | AF | None |
|  | Week 4-R | 68100 | M66M/I Q67Q/H/K/N K70K/R T107T/C | None | None | AF |
| 18 | Week 36 | 137 | AF | AF | AF | AF |
|  | Week 52 | 209 | T107S | None | K65K/R V106V/M | None |
|  | Week 62 | 50 | AF | AF | AF | AF |
|  | Week 88 | 39 | AF | AF | AF | AF |
|  | Week 104 | 89 | AF | AF | AF | None |
|  | Week 156 | 131 | AF | AF | AF | None |
| 19 | Oral Bridging | 2450 | N74D | ND | ND | ND |
|  | Week 62-R | 920 | ND | AF | AF | None |
|  | Week 156 | 473 | N74D | None | None | None |
| 20 | Week 10 | 10500 | K70H A105A/S/T T107T/N | None | None | None |
|  | Week 52 | 24100 | Q67K K70H | None | None | None |
|  | Week 88 | 15400 | Q67K K70H | None | None | None |
| 21 | Week 130 | 277 | None | None | L74I | None |
| 22 | Week 26 | 216 | None | ND | ND | ND |
|  | Week 26-R | 108 | None | None | None | None |
|  | Week 52 | 112 | AF | ND | ND | ND |
|  | Week 140 | 288 | None | AF | AF | None |
| 23 | Week 4 | 11000 | M66I T107A | ND | ND | None |
|  | Week 4-R | 6220 | M66I T107A | None | None | None |
|  | Week 10-R | 9310 | M66I T107A | None | None | None |
| 24 | Oral Bridging | 41300 | Q67H K70R T107T/N | None | None | None |
| 25 | Week 16 | 217 | AF | AF | AF | AF |
|  | Week 16-R | 5410 | None | None | None | None |
| 26 | Week 26-R | 116 | None | AF | AF | AF |
| 27 | Oral Bridging | 4050 | Q67Q/H | None | None | ND |
|  | Week 36 | 4480 | Q67Q/H | None | None | None |
| 28 | Week 4 | 75500 | Q67H K70R | None | None | AF |

*“-R” in the VF visit description indicates “retest”. ^†^Emergence of T107A or T107S polymorphisms alone is not associated with LEN resistance (Participants 13 and 18).

AF, assay failure; CA, capsid; IN, integrase; ND, not determined; PR, protease; RT, reverse transcriptase; VF, virologic failure.

#

# Supplementary Table 3. Treatment-Emergent Serious Adverse Events

| **Cohort/treatment** | **Serious adverse event*** | **Study day onset/resolution** | **Related to study drug** | **Serious adverse event outcome** |
| --- | --- | --- | --- | --- |
| Cohort 1 /LEN | Proctalgia | 288 to 289 | No | Recovered/resolved |
|  | Impaired Healing | 351 to 358 | No | Recovered/resolved |
|  | Anal cancer | 479 to 483 | No | Recovered/resolved |
|  | Anal squamous cell carcinoma | 257 to 259^†^ | No | Recovered/resolved |
| Cohort 1/ Placebo | Pancreatic Mass | 36, ongoing | No | Not recovered/not resolved |
|  | Abdominal Pain | 77 to 115 | No | Unknown |
|  | Clostridium difficile infection | 240 to 242 | No | Recovered/resolved |
|  | Angina pectoris | 367 to 368 | No | Recovered/resolved |
|  | Pneumonia | 970 to 979 | No | Recovered/resolved |
|  | Angina unstable | 985 to 997 | No | Recovered/resolved |
| Cohort 1/ LEN | COVID-19 | 266 to 275 | No | Recovered/resolved |
| Cohort 1 / Placebo | Pneumonia | 465 to 467 | No | Recovered/resolved |
| Cohort 1 / Placebo | COVID-19 | 403 to 423 | No | Recovered/resolved |
| Cohort 1 / LEN | Genital herpes simplex | 467 to 505 | No | Unknown |
| Cohort 1 / LEN | Pneumonia | 811, missing | No | Recovered/resolved |
| Cohort 1 / LEN | Angina pectoris | 1087, ongoing | No | Recovering/resolving |
| Cohort 1 / LEN | Cellulitis | 1221 to 1241 | No | Recovered/resolved |
| Cohort 1 / Placebo | Dengue fever | 1208 to 1214 | No | Recovered/resolved |
| Cohort 1 / LEN | Ejection fraction decreased | 1213, ongoing | No | Not recovered/not resolved |
| Cohort 2 / LEN | Dizziness | 38 to 43 | No | Recovered/resolved |
|  | Malignant Neoplasm | 80, ongoing | No | Fatal |
| Cohort 2 / LEN | Femoral Neck Fracture | 83, ongoing | No | Recovering/resolving |
| Cohort 2 / LEN | Septic Shock | 177 to 197 | No | Recovered/resolved |
|  | Renal Impairment | 221 to 236 | No | Recovered/resolved |
|  | Shock | 221 to 236 | No | Recovered/resolved |
|  | Urinary tract infection | 533 to 543 | No | Recovered/resolved |
|  | Pneumonitis | 1147, ongoing | No | Recovering/resolving |
| Cohort 2 / LEN | Mycoplasma pneumonia | 514 to 543 | No | Recovered/resolved |
|  | Pancytopenia | 528 to 543 | No | Recovered/resolved |
|  | Dehydration | 543, ongoing | No | Not recovered/not resolved |
|  | Acute respiratory failure | 553, ongoing | No | Fatal |
| Cohort 2 / LEN | Death (cause unknown)^‡^ | 551, 551 | No | Fatal |
| Cohort 2 / LEN | Pneumonia staphylococcal | 843 to 983 | No | Recovered/resolved |
| Cohort 2 / LEN | Congestive heart failure | 623 to 625 | No | Recovering/resolved with sequelae |
|  | Respiratory syncytial virus infection | 623 to 632 | No | Recovered/resolved |
| Cohort 2 / LEN | Prostate cancer | 487, ongoing | No | Not recovered/not resolved |
|  | Urinary retention | 515, ongoing | No | Not recovered/not resolved |
|  | Squamous cell carcinoma | 633, ongoing | No | Not recovered/not resolved |
|  | Acute kidney injury | 767 to 828 | No | Recovered/resolved |
|  | Hypotension | 767 to 828 | No | Recovered/resolved |
|  | E. coli bacteremia | 810 to 822 | No | Recovered/resolved |
| Cohort 2 / LEN | Inguinal hernia | 856 to 863 | No | Recovered/resolved |
|  | Influenza | 1066 to 1076 | No | Recovered/resolved |
|  | Pneumonia | 1078 to 1090 | No | Recovered/resolved |
|  | Cellulitis | 1078 to 1111 | No | Recovered/resolved |
| Cohort 2 / LEN | Dehydration | 1000 to 1001 | No | Recovered/resolved |
| Cohort 2 / LEN | Lower gastrointestinal hemorrhage | 944 to 948 | No | Recovered/resolved |

*Including only treatment-emergent adverse events, defined as those that began on or after the first dose of LEN.

^†^The investigator considered “getting better” as adverse event recovered/resolved.

^‡^Participant had alcoholic liver disease and depression prior to death.

LEN, lenacapavir.

**Supplementary Table 4. Lenacapavir-Related Injection Site Reactions**

| % | **Any** | **Grade 1** | **Grade 2** | **Grade 3** | **None** |
| --- | --- | --- | --- | --- | --- |
| **SC injection 1 (Day 1 SC, n=72)** | | | | | |
| Any | 61.1 | 47.2 | 11.1 | 2.8 | 38.9 |
| Swelling | 29.2 | 22.2 | 5.6 | 1.4 | 70.8 |
| Erythema | 26.4 | 18.1 | 6.9 | 1.4 | 73.6 |
| Pain | 23.6 | 15.3 | 6.9 | 1.4 | 76.4 |
| Nodule | 27.8 | 27.8 | 0.0 | 0.0 | 72.2 |
| Induration | 11.1 | 9.7 | 1.4 | 0.0 | 88.9 |
| **SC injection 2 (Week 26, n=70)** | | | | | |
| Any | 45.7 | 34.3 | 11.2 | 0.0 | 54.3 |
| Swelling | 18.6 | 15.7 | 2.9 | 0.0 | 81.4 |
| Erythema | 15.7 | 12.9 | 2.9 | 0.0 | 84.3 |
| Pain | 22.9 | 15.7 | 7.1 | 0.0 | 77.1 |
| Nodule | 15.7 | 14.3 | 1.4 | 0.0 | 84.3 |
| Induration | 10.0 | 5.7 | 4.3 | 0.0 | 90.0 |
| **SC injection 3 (Week 52, n=64)** | | | | | |
| Any | 53.1 | 43.8 | 7.8 | 1.6 | 46.9 |
| Swelling | 25.0 | 20.3 | 3.1 | 1.6 | 75.0 |
| Erythema | 15.6 | 10.9 | 3.1 | 1.6 | 84.4 |
| Pain | 20.3 | 14.1 | 4.7 | 1.6 | 79.7 |
| Nodule | 18.8 | 18.8 | 0.0 | 0.0 | 81.2 |
| Induration | 6.3 | 6.3 | 0.0 | 0.0 | 93.7 |
| **SC injection 4 (Week 78, n=57)** | | | | | |
| Any | 42.1 | 36.8 | 3.5 | 1.8 | 57.9 |
| Swelling | 15.8 | 10.5 | 3.5 | 1.8 | 84.2 |
| Erythema | 10.5 | 8.8 | 1.8 | 0.0 | 89.5 |
| Pain | 14.0 | 14.0 | 0.0 | 0.0 | 86.0 |
| Nodule | 14.0 | 14.0 | 0.0 | 0.0 | 86.0 |
| Induration | 5.3 | 5.3 | 0.0 | 0.0 | 94.7 |
| **SC injection 5 (Week 104, n=56)** | | | | | |
| Any | 37.5 | 33.9 | 1.8 | 1.8 | 62.5 |
| Swelling | 14.3 | 12.5 | 1.8 | 0.0 | 85.7 |
| Erythema | 8.9 | 7.1 | 0.0 | 1.8 | 91.1 |
| Pain | 14.3 | 12.5 | 1.8 | 0.0 | 85.7 |
| Nodule | 10.7 | 10.7 | 0.0 | 0.0 | 89.3 |
| Induration | 5.4 | 5.4 | 0.0 | 0.0 | 94.6 |
| **SC injection 6 (Week 130, n=40)** | | | | | |
| Any | 30.0 | 30.0 | 0.0 | 0.0 | 70.0 |
| Swelling | 10.0 | 10.0 | 0.0 | 0.0 | 90.0 |
| Erythema | 10.0 | 10.0 | 0.0 | 0.0 | 90.0 |
| Pain | 12.5 | 12.5 | 0.0 | 0.0 | 87.5 |
| Nodule | 7.5 | 7.5 | 0.0 | 0.0 | 92.5 |
| Induration | 5.0 | 5.0 | 0.0 | 0.0 | 95.0 |

SC, subcutaneous.

# Supplementary Table 5. Grade 3 or 4 Laboratory Abnormalities

| **n (%)** | **Cohort 1 (n=36)** | **Cohort 2 (n=36)** | **Total (N=72)** |
| --- | --- | --- | --- |
| Low creatinine clearance | 9 (25.) | 10 (27.8) | 19 (26.4) |
| High creatinine | 8 (22.2) | 6 (16.7) | 14 (19.4) |
| Glycosuria | 3 (8.3) | 3 (8.3) | 6 (8.3) |
| Hyperglycemia (non-fasting)* | 3 (8.6) | 2 (6.3) | 5 (7.5) |
| Proteinuria | 2 (5.6) | 3 (8.3) | 5 (6.9) |
| Hyperglycemia (fasting)^†^ | 3 (9.4) | 1 (3.0) | 4 (6.2) |
| High alanine aminotransferase | 0 | 4 (11.1) | 4 (5.6) |
| High aspartate aminotransferase | 1 (2.8) | 3 (8.3) | 4 (5.6) |
| High direct bilirubin | 0 | 3 (8.3) | 3 (4.2) |
| Low hemoglobin | 1 (2.8) | 2 (5.6) | 3 (4.2) |

Abnormalities in >2 participants in either cohort are shown. Low creatinine clearance (Grade 3: <60 to 30 ml/min, Grade 4: <30 ml/min) or high creatinine (Grade 3: >1.8 to <3.5 x the upper limit of normal or increase to 1.5 to <2.0 x participant’s baseline, Grade 4: ≥3.5 x the upper limit of normal or increase to ≥2.0 x participant’s baseline) were transient. Severity grades were defined by Division of AIDS (DAIDS) Table for Grading the Severity of Adult and Pediatric Adverse Events. Hyperglycemia or glycosuria were transient or related to underlying diabetes.

*Cohort 1, n=35; Cohort 2, n=32; Total, n=67.

^†^Cohort 1, n=32, Cohort 2, n=33; Total, n=65.

# Supplementary Figure 1. Virologic Outcomes (FDA Snapshot Algorithm) According to Cohort at Week 156


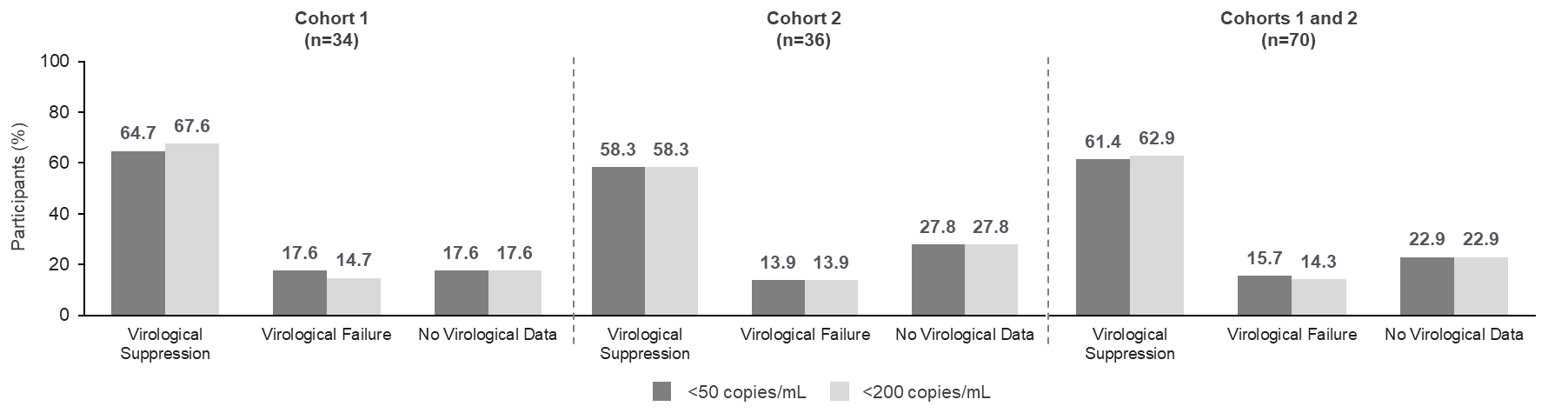


FDA, US Food and Drug Administration.

# Supplementary Figure 2. CD4 Cell Count Changes According to Cohort


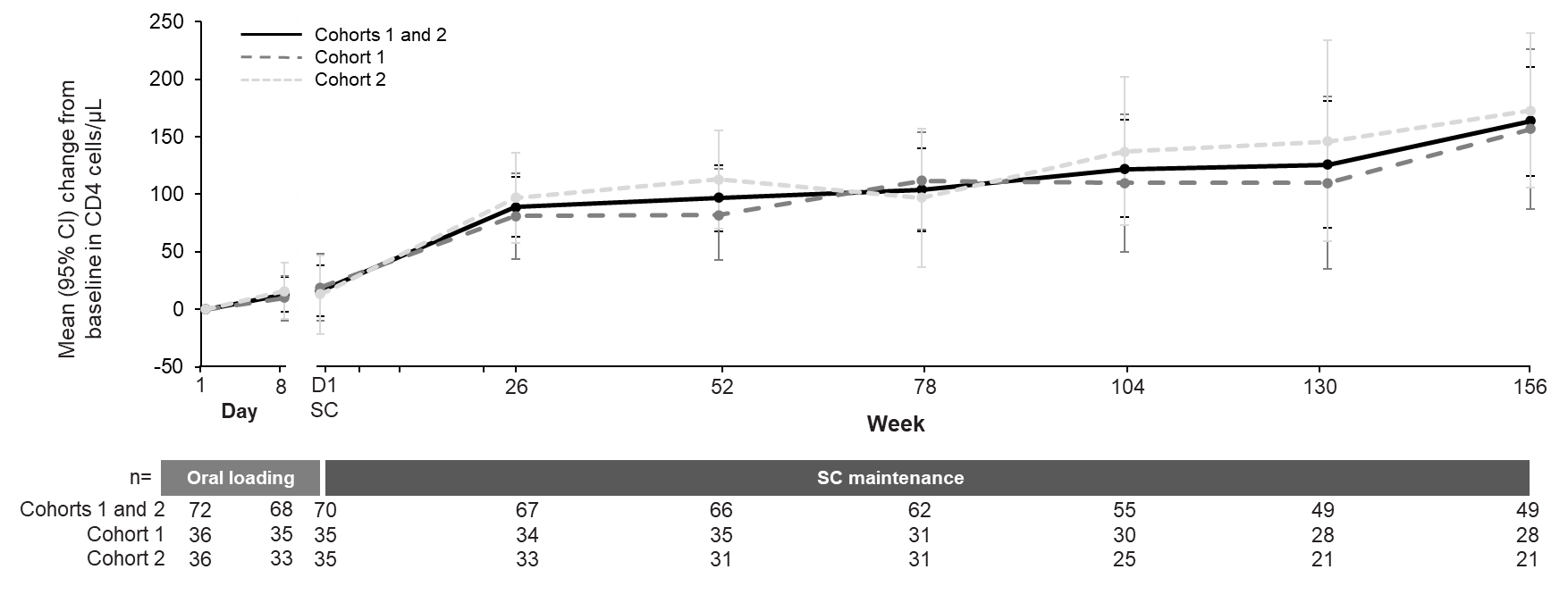


D, Day; SC, subcutaneous.
