# Supplementary material for: Subcutaneous Lenacapavir in People With Multidrug-Resistant HIV-1: 156 Week Results of the CAPELLA Study
Source: Open Forum Infect Dis. 2025 Dec 19;13(1):ofaf763. doi: 10.1093/ofid/ofaf763 (PMC12740718; doi:10.1093/ofid/ofaf763)
Supplement: ofaf763_Supplementary_Data [file ofaf763_supplementary_data.zip › Prot GS-US-200-4625 amd-5_DS_QCed-f-redact.pdf.pdf]

CLINICAL STUDY PROTOCOL

**Study Title:**	A Phase 2/3 Study to Evaluate the Safety and Efficacy of Long-Acting Capsid Inhibitor GS-6207 in Combination With an Optimized Background Regimen in Heavily Treatment Experienced People Living With HIV-1 Infection With Multidrug Resistance
**Sponsor:**	Gilead Sciences, Inc.<br>333 Lakeside Drive<br>Foster City, CA 94404
**IND Number:**	136260
**EU CT Number:**	2019-003814-16
**Clinical Trials.gov Identifier:**	04150068
**Indication:**	HIV-1 Infection
**Protocol ID:**	GS-US-200-4625
**Contact Information:**	The medical monitor name and contact information will be provided on the Key Study Team Contact List.
**Protocol Version/Date:**	Amendment 5: 22 May 2024
**Amendment History:**	Original: 25 September 2019<br>Amendment 1: 18 December 2019<br>Amendment 2: 01 September 2020<br>Amendment 3: 01 February 2022<br>Amendment 4: 18 January 2023<br><br>A high-level summary of the changes in each amendment is provided in Appendix 7.
**Country-specific Requirements:**	Country-specific requirements, as applicable, are listed in Appendix 6.

This study will be conducted under US Food & Drug Administration IND regulations (21 CFR Part 312); however, sites located in the European Economic Area and Switzerland are not included under the IND and are considered non-IND sites.

CONFIDENTIALITY STATEMENT
The information contained in this document, particularly unpublished data, is the property or under control of Gilead Sciences, Inc., and is provided to you in confidence as an investigator, potential investigator, or consultant, for review by you, your staff, and an applicable Institutional Review Board or Independent Ethics Committee. The information is only to be used by you in connection with authorized clinical studies of the investigational drug described in the protocol. You will not disclose any of the information to others without written authorization from Gilead Sciences, Inc., except to the extent necessary to obtain informed consent from those persons to whom the drug may be administered.

TABLE OF CONTENTS

TABLE OF CONTENTS 3
LIST OF IN-TEXT TABLES 5
LIST OF IN-TEXT FIGURES 6
PROTOCOL SYNOPSIS 7
GLOSSARY OF ABBREVIATIONS AND DEFINITION OF TERMS 18
1. INTRODUCTION 21
  1.1. Background 21
  1.2. Lenacapavir (GS-6207) 21
    1.2.1. General Information 21
    1.2.2. Clinical Studies of Lenacapavir 22
  1.3. Rationale for This Study 25
  1.4. Rationale for Dose Selection of Lenacapavir 26
  1.5. Rationale for Oral Weekly Bridging of Lenacapavir for Missed SC Injection 27
  1.6. Risk/Benefit Assessment for the Study 28
  1.7. Compliance 28
2. OBJECTIVES 29
3. STUDY DESIGN 30
  3.1. Endpoints 30
  3.2. Study Design 30
  3.3. Study Treatments 32
  3.4. Duration of Treatment 33
  3.5. Biomarker Testing 34
  CCI [redacted]
4. PARTICIPANT POPULATION 35
  4.1. Number of Participants and Participant Selection 35
  4.2. Inclusion Criteria 35
  4.3. Exclusion Criteria 36
5. INVESTIGATIONAL MEDICINAL PRODUCTS 38
  5.1. Randomization, Enrollment, Blinding, and Treatment Codes Access 38
    5.1.1. Randomization and Enrollment 38
    5.1.2. Blinding 38
    5.1.3. Procedures for Breaking Treatment Codes 39
  5.2. Description and Handling of Lenacapavir Injection, Lenacapavir Tablets, and Placebo to Match Lenacapavir Tablets 39
    5.2.1. Formulation 39
    5.2.2. Packaging and Labeling 40
    5.2.3. Storage and Handling 40
  5.3. Dosage and Administration of Lenacapavir 40
  5.4. Dosing and Administration of Oral Weekly Bridging of Lenacapavir 41
  5.5. Prior and Concomitant Medications 43
  5.6. Accountability for Study Drug 45
    5.6.1. Study Drug Return or Disposal for Lenacapavir and Placebo to Match Lenacapavir Tablets 46
6. STUDY PROCEDURES 47

6.1. Participant Enrollment and Treatment Assignment 47
6.2. Pretreatment Assessments 47
6.2.1. Screening Assessments....47
6.3. Treatment Assessments 48
6.3.1. Functional Monotherapy Period and Oral Lead-in Period Assessments....48
6.3.2. Maintenance Period Assessments....49
6.4. Posttreatment Assessments....52
6.4.1. Early Termination Visit Assessments 52
6.4.2. 30-Day, 90-Day, and 180-Day Follow-Up Visits 54
6.5. Clinical Laboratory Assessments 55
6.5.1. Blood Samples....55
6.5.2. Blood and Urine Storage Samples....57
6.5.3. Urine Samples 57
6.6. Criteria for Discontinuation of Study Treatment....58
6.7. Management of Virologic Failure 58
6.7.1. Suboptimal Virologic Response 58
6.7.2. Virologic Rebound 59
6.7.3. Viremia at Last Visit 59
6.8. End of Study....59
6.9. Poststudy Care 59

7. ADVERSE EVENTS AND TOXICITY MANAGEMENT 60

7.1. Definitions of Adverse Events and Serious Adverse Events....60
7.1.1. Adverse Events....60
7.1.2. Serious Adverse Events....60
7.2. Assessment of Adverse Events and Serious Adverse Events....61
7.2.1. Assessment of Causality for Study Drugs and Procedures 61
7.2.2. Assessment of Severity....61
7.3. Investigator Requirements and Instructions for Reporting Adverse Events and Serious Adverse Events....62
7.3.1. Adverse Events....62
7.3.2. Serious Adverse Events....62
7.4. Gilead Reporting Requirements 63
7.5. Clinical Laboratory Abnormalities and Other Abnormal Assessments as Adverse Events or Serious Adverse Events....64
7.6. Toxicity Management....64
7.6.1. Grades 1 and 2 Laboratory Abnormality or Clinical Event 65
7.6.2. Grade 3 Laboratory Abnormality or Clinical Event....65
7.6.3. Grade 4 Laboratory Abnormality or Clinical Event....65
7.7. Special Situations Reports....66
7.7.1. Definitions of Special Situations....66
7.7.2. Instructions for Reporting Special Situations....66

8. STATISTICAL CONSIDERATIONS 69

8.1. Analysis Objectives and Endpoints....69
8.1.1. Analysis Objectives....69
8.1.2. Primary Endpoint....69
8.1.3. Secondary Endpoints....69
8.2. Planned Analyses....70
8.2.1. Interim Analysis 70
8.2.2. Primary Analysis 70
8.2.3. Year 2 Analysis 71
8.2.4. Year 3 Analysis 71
8.2.5. Final Analysis....71

8.3. Analysis Conventions 71
8.3.1. Analysis Sets 71
8.3.2. Data Handling Conventions 72
8.4. Demographic and Baseline Characteristics Analysis 72
8.5. Efficacy Analysis....72
8.5.1. Primary Analysis 72
8.5.2. Secondary Analyses....73
8.6. Safety Analysis....74
8.6.1. Extent of Exposure 74
8.6.2. Adverse Events....74
8.6.3. Laboratory Evaluations 75
8.6.4. Other Safety Evaluations....75
8.7. Adjustments for Multiplicity 75
8.8. Pharmacokinetic Analysis 75
8.9. Sample Size 75
8.10. Data Monitoring Committee....76
9. RESPONSIBILITIES....77
9.1. Investigator Responsibilities 77
9.1.1. Good Clinical Practice....77
9.1.2. Good Postmarketing Study Practices (Japan Only)....77
9.1.3. Financial Disclosure 77
9.1.4. Institutional Review Board/Independent Ethics Committee Review and Approval....77
9.1.5. Informed Consent 77
9.1.6. Confidentiality....78
9.1.7. Study Files and Retention of Records 78
9.1.8. Case Report Forms 80
9.1.9. Investigator Inspections....80
9.1.10. Protocol Compliance 80
9.2. Sponsor Responsibilities 80
9.2.1. Protocol Modifications 80
9.2.2. Study Reports and Publications....81
9.3. Joint Investigator/Sponsor Responsibilities 81
9.3.1. Payment Reporting 81
9.3.2. Access to Information for Monitoring....81
9.3.3. Access to Information for Auditing or Inspections 81
9.3.4. Study Discontinuation 82
9.3.5. Data Protection 82
10. REFERENCES....83
11. APPENDICES....84

LIST OF IN-TEXT TABLES

Table 1. Oral Dosing of Lenacapavir or Placebo to Match 41
Table 2. Dosing Schedule for Oral Weekly Bridging and Resumption of SC Lenacapavir Injection 42
Table 3. Missed Dose Recommendations for Lenacapavir During Oral Weekly Bridging 43
Table 4. List of Representative Medications That are Prohibited or To Be Used With Caution Due to the Potential for Drug-Drug Interaction With Lenacapavir....44

LIST OF IN-TEXT FIGURES

Figure 1. Simulated Pharmacokinetic Profile of Oral Weekly Bridging of Lenacapavir (300 mg) (A) Prior to and (B) After Resuming SC Injection 27
Figure 2. Study Design Schema 31

PROTOCOL SYNOPSIS

Gilead Sciences, Inc.
333 Lakeside Drive
Foster City, CA 94404

**Study Title:**	A Phase 2/3 Study to Evaluate the Safety and Efficacy of Long-Acting Capsid Inhibitor GS-6207 in Combination With an Optimized Background Regimen in Heavily Treatment Experienced People Living With HIV-1 Infection With Multidrug Resistance
**IND Number:**<br>**EU CT Number:**<br>**Clinical Trials.gov Identifier:**	136260<br>2019-003814-16<br>04150068
**Study Centers Planned:**	Approximately 75 centers globally
**Objectives:**	The primary objective of this study is:<br>• To evaluate the antiviral activity of lenacapavir (LEN; GS-6207) administered as an add-on to a failing regimen (functional monotherapy) for people with HIV (PWH) with multidrug resistance as determined by the proportion of participants achieving at least 0.5 $\log_{10}$ reduction from baseline in HIV-1 RNA at the end of the Functional Monotherapy Period.<br>The secondary objectives of this study are:<br>• To evaluate the safety and efficacy of LEN in combination with an Optimized Background Regimen (OBR) at Weeks 26 and 52.<br>• To evaluate the safety and efficacy of LEN in combination with an OBR at Weeks 104 and 156 (from the first subcutaneous [SC] dose of LEN).

	CCI [redacted]
**Study Design:**	This is a global multicenter study of LEN together with an OBR in PWH with multidrug–resistant infection.<br>Participants who complete a screening visit will return to the clinic between 14 and 30 days after the screening visit, for a Cohort Selection visit. HIV-1 RNA results from this Cohort Selection visit will be used to determine whether eligible participants will participate in Cohort 1 or Cohort 2.<br>**<u>Cohort 1 (n = 36)</u>**<br>**Functional Monotherapy Period**<br>Eligible participants with a < 0.5 $\log_{10}$ HIV-1 RNA decline compared to the screening visit and HIV-1 RNA ≥ 400 copies/mL at the Cohort Selection visit will be randomized, in a blinded fashion, in a 2:1 ratio to receive either oral LEN or placebo to match LEN for 14 days. Treatment assignment will be blinded to the sponsor, participants, investigators, and study staff at the site. Functional Monotherapy will be assessed while participants continue their failing regimen.<br>After each participant completes the Functional Monotherapy Period, their treatment assignment will be unblinded.<br>**Maintenance Period**<br>Participants who were randomized to receive oral LEN will receive SC LEN and initiate their OBR on Day 1 SC (14 days after the first dose of oral LEN) (**Cohort 1A**).<br>Participants who were randomized to receive placebo will receive oral LEN and initiate their OBR on Day 15 (**Cohort 1B**). They will receive SC LEN at Day 1 SC

	(eg, 14 days after the first dose of oral LEN) while continuing their OBR.<br>After the Day 1 SC visit, all Cohort 1 participants will continue with study visits at Weeks 4, 10, 16, 22, 26, 36, and 52 (study visits Week X are identified by the number of weeks that have elapsed since the Day 1 SC visit when LEN is first administered by injection and excludes Oral Bridging Periods).<br>Participants will receive their subsequent SC LEN injection at the Week 26 visit. At the Week 52 visit, participants will be given an option to receive SC LEN injection and continue on the study to receive SC LEN injections once every 6 months (26 weeks) ± 2 weeks from last injection.<br>**Cohort 2 (n = 64)**<br>**Oral Lead-in Period**<br>Participants will be enrolled into Cohort 2 if Cohort 1 is fully enrolled or if they do not meet the criteria for randomization in Cohort 1 (ie, they had ≥ 0.5 $\log_{10}$ HIV-1 RNA decline compared to the screening visit and/or HIV-1 RNA < 400 copies/mL at the Cohort Selection visit). All Cohort 2 participants will receive oral LEN for 14 days starting at Day 1. Participants will initiate an OBR on Day 1.<br>**Maintenance Period**<br>At Day 1 SC (ie, 14 days after the first dose of oral LEN), participants will receive SC LEN and will continue their OBR. After the Day 1 SC visit, participants will continue with study visits at Weeks 4, 10, 16, 22, 26, 36, and 52 (study visits Week X are identified by the number of weeks that have elapsed since the Day 1 SC visit when LEN is first administered by injection and excludes Oral Bridging Periods). Participants will receive their subsequent SC LEN injection at the Week 26 visit. At the Week 52 visit, participants will be given an option to receive SC LEN injection and continue the study to receive SC LEN injections once every 6 months (26 weeks) ± 2 weeks from last injection. At the Week 156 visit, participants will be given an option to receive SC LEN injection and continue the study to receive SC LEN injections once every 6 months (26 weeks) ± 2 weeks from last injection.
Number of Participants Planned:	Approximately 100 participants may be enrolled in this study.<br>36 participants will be enrolled in Cohort 1. Up to 64 participants may be enrolled in Cohort 2.

Target Population:	Heavily treatment-experienced adults and adolescent PWH with resistance to ≥ 2 antiretroviral (ARV) medications from each of ≥ 3 of the 4 main classes of ARV medication and plasma HIV-1 RNA ≥ 400 copies/mL at screening while taking a failing regimen.<br><br>Adolescent participants will be enrolled only at sites in North America and Dominican Republic.
Duration of Treatment:	Duration of treatment is at least 54 weeks.<br><br>Following successful completion of Week 52 visit, participants will be given the option to attend visits at Week 62, 78, 88, 104, 114, 130 and will continue to alternate between every 10 weeks and every 16 weeks. Participants willing to continue the study beyond Week 52 will receive SC LEN 927 mg once every 6 months (26 weeks) ± 2 weeks from last injection starting at Week 52 visit, while continuing their OBR, until the product becomes accessible to participants through an access program, is commercially available, or until Gilead elects to discontinue the study in the country.<br><br>Participants who decide not to receive SC LEN at Week 52 and not to continue the study will complete the study at Week 52 visit.<br><br>At Week 156 and thereafter, upon regulatory and/or institutional review board (IRB)/independent ethics committee (IEC) approval of the protocol and when participants have provided written informed consent of the most current IRB-approved consent form, participants will continue to receive study drug every 26 weeks, and continue to attend visits every 26 weeks (± 2 weeks). Participants will receive SC LEN every 6 months (26 weeks) ± 2 weeks from the last injection starting at the Week 156 visit, while continuing their OBR, until the product becomes accessible to participants through an access program, is commercially available, or until Gilead elects to discontinue the study in the country.<br><br>Participants who decide to discontinue SC LEN early and do not wish to continue to attend study visits through the Week 156 visit or the next scheduled SC dosing visit will return to the clinic for 30-day, 90-day, and 180-day follow-up visits after the early termination visit. The 180-day follow-up may be conducted via a phone call per the investigator's discretion.

Diagnosis and Main Eligibility Criteria:	People living with HIV who meet the following criteria:<br>• Adults and adolescents aged ≥ 12 and weighing ≥ 35 kg.<br>• Are receiving a stable failing regimen for > 8 weeks before screening and are willing to continue that regimen until Day 1. Cohort 1 participants should be willing to continue their failing regimen until Day 14.<br>• Have HIV-1 RNA ≥ 400 copies/mL at screening.<br>• Have resistance to ≥ 2 ARV medications from each of ≥ 3 of the 4 main classes of ARV medications (nucleoside reverse transcriptase inhibitor [NRTI], nonnucleoside reverse transcriptase inhibitor [NNRTI], protease inhibitor [PI], integrase strand-transfer inhibitor [INSTI]). Resistance to emtricitabine (FTC) or lamivudine (3TC) associated with the presence of the M184V/I reverse transcriptase (RT) mutation cannot be used for the purpose of determining eligibility for this criterion.<br>• Have ≤ 2 fully active ARV remaining from the 4 main classes that can be effectively combined to form a viable regimen in the opinion of the investigator based on resistance, tolerability, contraindication, safety, drug access, or acceptability to the participant.<br>• Able and willing to receive an OBR together with LEN.
Study Procedures/ Frequency:	At screening, laboratory analyses (hematology, chemistry and urinalysis, and serum pregnancy test [for women]), HIV-1 RNA, CD4+ cell count, vital signs, electrocardiogram, complete physical examination, and estimated glomerular filtration rate will be performed, and hepatitis B virus and hepatitis C virus serologies will be analyzed.<br>Laboratory samples for resistance analysis will be collected. Analysis of the participant's HIV-1 resistance to support eligibility will be completed.<br>Participants who complete a screening visit will return to the clinic between 14 and 30 days after the screening visit for a Cohort Selection visit. HIV-1 RNA results from this follow-up visit will be used to randomize participants in Cohort 1 or enroll them in Cohort 2.<br>• Cohort 1 (n = 36): Participants in Cohort 1 will be randomized to receive oral LEN or placebo to match LEN during the Functional Monotherapy Period while

	continuing their failing regimen. During the Maintenance Period, they will receive SC LEN and an OBR.<br>• Cohort 2 (n = 64): Participants in Cohort 2 will be enrolled to receive oral LEN and an OBR in the Oral Lead-in Period. During the Maintenance Period they will receive SC LEN and continue their OBR.<br>**Functional Monotherapy Period (Cohort 1 only)**<br>Participants with both < 0.5 $\log_{10}$ HIV-1 RNA decline compared to the screening visit and HIV-1 RNA ≥ 400 copies/mL at the Cohort Selection visit will be randomized to receive oral LEN or placebo to match LEN while continuing their failing regimen. Sites, participants, and sponsor will be blinded to the participant treatment assignment.<br>Participants will visit the clinic on Days 1, 2, 5 (± 1) (if possible) and 8. On Day 1 before dosing, baseline assessments will be conducted including adverse events (AEs), concomitant medications, laboratory assessments and physical examinations as detailed in the Study Procedures Table (Appendix 2). After completion of the assessments, the participant will receive oral LEN 600 mg, 600 mg, and 300 mg or placebo to match LEN on Days 1, 2, and 8, respectively.<br>After each participant completes the Functional Monotherapy Period, their treatment assignment will be unblinded.<br>**Maintenance Period (Cohort 1A)**<br>Participants who received oral LEN during the Functional Monotherapy Period will receive SC LEN 927 mg and initiate an OBR at the Day 1 SC visit (14 days after the first dose of oral LEN). They will continue with study visits at Weeks 4, 10, 16, 22, 26, 36, and 52. Participants will receive their subsequent SC LEN injection at the Week 26 visit. At the Week 52 visit, participants will be given an option to receive SC LEN injection and continue on the study to receive SC LEN injections once every 6 months (26 weeks) ± 2 weeks from last injection.<br>**Maintenance Period (Cohort 1B)**<br>Participants who received placebo to match LEN during the Functional Monotherapy Period (Day 1 to Day 14) will visit the clinic on Days 15, 16, 19 (± 1) (if possible) and 22. They will receive oral LEN 600 mg and initiate their OBR on Day 15. After completing study visit assessments, participants

	will receive additional oral LEN 600 mg and 300 mg on Days 16 and Day 22, respectively.<br><br>Participants will receive SC LEN 927 mg at the Day 1 SC visit (14 days after the first dose of oral LEN) while continuing their OBR. They will continue with study visits at Weeks 4, 10, 16, 22, 26, 36, and 52. Participants will receive their subsequent SC LEN 927 mg injection at the Week 26 visit.<br><br>**Oral Lead-in (Cohort 2 only)**<br><br>Participants will be enrolled into Cohort 2 if Cohort 1 is fully enrolled or if they do not meet the criteria for randomization in Cohort 1 (ie, they had ≥ 0.5 $\log_{10}$ HIV-1 RNA decline compared to the screening visit and/or HIV-1 RNA < 400 copies/mL at the Cohort Selection visit). Participants will be enrolled in Cohort 2 to initiate oral LEN. Participants will initiate their OBR on Day 1.<br><br>Participants will visit the clinic on Days 1, 2, 5 (± 1) and 8 during the Oral Lead-in Period.<br><br>On Day 1 before dosing, baseline assessments will be conducted including AEs, concomitant medications, laboratory tests and physical examinations as detailed in the Study Procedures Table (Appendix 2). After completion of the assessments, the participant will receive oral LEN 600 mg, 600 mg, and 300 mg on Days 1, 2, and 8, respectively.<br><br>**Maintenance Period (Cohort 2)**<br><br>Participants will receive SC LEN 927 mg at Day 1 SC visit (14 days after the first dose of oral LEN) while continuing their OBR. They will continue with study visits at Weeks 4, 10, 16, 22, 26, 36, and 52. Participants will receive their subsequent SC LEN 927 mg injection at the Week 26 visit.<br><br>**<u>Cohort 1 and Cohort 2</u>**<br><br>At the Week 52 visit, all participants (Cohorts 1 and 2) will be given an option to continue to complete study visits at Week 62, 78, 88, 104, 114, 130 and will continue to alternate between every 10 weeks and every 16 weeks. Participants willing to continue beyond Week 52 visit will receive SC LEN 927 mg once every 6 months (26 weeks) ± 2 weeks from last injection starting at Week 52 visit.<br><br>At Week 156 and thereafter, upon regulatory and/or IRB/IEC approval of the protocol and when participants have provided written informed consent of the most current IRB-approved

	consent form, participants will continue to receive study drug every 26 weeks, and continue to attend visits every 26 weeks (± 2 weeks). Participants will receive SC LEN every 6 months (26 weeks) ± 2 weeks from last injection starting at Week 156 visit, while continuing their OBR, until the product becomes accessible to participants through an access program, is commercially available, or until Gilead elects to discontinue the study in the country.<br><br>At each visit, AEs, concomitant medications, laboratory tests and physical examinations will be performed in accordance with the Study Procedures Table (Appendix 2).<br><br>Participants may require oral weekly bridging 300 mg if an SC injection of LEN cannot be administered for any reason within the protocol visit window.<br><br>**Resistance Analysis:**<br><br>Plasma samples for genotypic and phenotypic testing of HIV-1 will be collected in accordance with the Study Procedures Table (Appendix 2). Screening results and/or historic HIV-1 resistance reports will be used to determine eligibility and to help construct the OBR.<br><br>**Pharmacokinetic Assessments:**<br><br>PK sampling will occur relative to dosing of LEN at the following time points for all participants:<br><br>• Day 1: Predose (within 30 minutes of dosing), 1, 2, 4, 6, and 8 hours postdose.<br><br>• Day 2 and 8:<br>— Predose (within 30 minutes of dosing).<br>— A single timed PK sample between 1 and 6 hours postdose.<br><br>• Day 5 (if visit occurs): A single anytime PK sample.<br><br>• Day 15 (for Cohort 1B participants only): Predose (within 30 minutes of dosing), 1, 2, 4, 6, and 8 hours postdose.<br><br>• Day 16 and 22 (for Cohort 1B participants only):<br>— Predose (within 30 minutes of dosing).<br>— A single timed PK sample between 1 and 6 hours postdose.

	• Day 19 (for Cohort 1B participants only, if visit occurs): A single anytime PK sample.<br>• During the Maintenance Period, at all visits without SC LEN injections, including the Oral Bridging visits, if applicable: A single anytime PK sample will be collected.<br>• During the Maintenance Period, at all visits with SC LEN injections, including when SC LEN injection is resumed after the Oral Bridging visits, if applicable: A single predose (within 30 minutes of dosing) PK sample will be collected.<br>At Week 156 and thereafter, upon regulatory and/or IRB/IEC approval of the protocol and when participants have provided written informed consent of the most current IRB-approved consent form, participants will continue to receive study drug every 26 weeks, and continue to attend visits every 26 weeks (± 2 weeks). Participants will not be required to provide PK samples for these visits.<br>**Patient reported outcomes:** Participants ≥ 18 years of age will complete the following, if available:<br>• Symptoms Distress Module, Short Form Health Survey (SF-36), EuroQol (5 dimensions, 5 levels) (EQ-5D-5L) at Day 1, Weeks 4, 16, 26, and 52.<br>• The Numeric Pain Rating Scale at Day 1 SC, Weeks 26 and 52.
**Test Product, Dose, and Mode of Administration:**	Oral LEN and SC LEN injection. Study drug (oral and SC LEN) will be administered without regard to food.
**Reference Therapy, Dose, and Mode of Administration:**	Placebo to match LEN (during the randomized Functional Monotherapy Period)<br>None (during the Maintenance Period)

**Criteria for Evaluation:**	
Safety:	Incidence of treatment-emergent AEs and clinical laboratory abnormalities
Efficacy:	The primary endpoint is:<br>• The proportion of participants in Cohort 1 achieving $\geq 0.5 \log_{10}$ copies/mL reduction from baseline in HIV-1 RNA at the end of the Functional Monotherapy Period.<br>The secondary endpoints are:<br>• The proportion of participants in Cohort 1 with plasma HIV-1 RNA < 50 copies/mL and < 200 copies/mL at Weeks 26 and 52 visits based on the United States (US) Food and Drug Administration (FDA)–defined snapshot algorithm.<br>• The proportion of participants in combined Cohorts 1 and 2 with plasma HIV-1 RNA < 50 copies/mL and < 200 copies/mL at Weeks 104 and 156 from the first SC dose of LEN based on the US FDA–defined snapshot algorithm.
**Statistical Methods:**	The primary efficacy analysis is to compare the proportion of participants in Cohort 1 achieving $\geq 0.5 \log_{10}$ reduction from baseline in HIV-1 RNA at the end of the Functional Monotherapy Period. The *P* value and 95% CI for the difference in response rates between 2 treatment groups (LEN in Cohort 1A and placebo in Cohort 1B) will be estimated and constructed based on an unconditional exact method using 2 invert 1-sided tests with an alpha level at 0.05.<br>The proportion of participants in Cohort 1 with HIV-1 RNA < 50 copies/mL and < 200 copies/mL at Weeks 26 and 52, and the proportion of participants in combined Cohorts 1 and 2 with HIV-1 RNA < 50 copies/mL and < 200 copies/mL at Weeks 104 and 156 (from the first SC dose of LEN) will be summarized using descriptive statistics based on the US FDA-defined snapshot algorithm.<br>Incidence of treatment-emergent AEs and treatment-emergent laboratory abnormalities will be summarized.<br>A total of 36 participants in Cohort 1 will provide at least 90% power to detect a 60% difference in the proportion of participants achieving a $\geq 0.5 \log_{10}$ reduction from baseline at

	the end of the Functional Monotherapy Period between treatment groups (LEN in Cohort 1A and placebo in Cohort 1B). In this sample size and power computation, it is assumed that 70% and 10% of participants achieve a $\geq$ 0.5 log_{10} reduction from baseline in HIV-1 RNA in the LEN group (Cohort 1A) and the placebo group (Cohort 1B) (based on data from Trogarzo Phase 3 TMB-301 study), respectively, and the Fisher exact test is conducted at 2-sided significant level of 0.05.<br><br>A total sample size of 36 participants from Cohort 1A and Cohort 1B will provide reasonable assessment of safety for at least 26 weeks of treatment in heavily treatment-experienced participants.<br><br>The external multidisciplinary Data Monitoring Committee will review the progress, efficacy, and safety data after all participants in Cohort 1 have completed 14 days of assessment in the Functional Monotherapy Period or discontinued the study drug. Further enrollment will be stopped if 50% or more of the participants in the LEN group fail to achieve at least 0.5 log_{10} reduction from baseline in HIV-1 RNA at the end of the Functional Monotherapy Period. The decision whether to continue with the study and the development of LEN will be based on the magnitude of the HIV-1 RNA decline at the end of the Functional Monotherapy Period.

This study will be conducted in accordance with the guidelines of Good Clinical Practice (GCP) and Good Postmarketing Study Practice (GPSP) after approval of Lenacapavir in Japan including archiving of essential documents.

“Clinical trial” shall read “postmarketing clinical trial” after approval of Lenacapavir in Japan.

GLOSSARY OF ABBREVIATIONS AND DEFINITION OF TERMS

%CV	percentage coefficient of variation
3TC	lamivudine
AE	adverse event
AIDS	acquired immunodeficiency syndrome
ALT	alanine aminotransferase
ART	antiretroviral therapy
ARV	antiretroviral
$AUC_{x\text{-}xx}$	partial area under the concentration versus time curve from time "x" to time "xx"
BCRP	breast cancer resistance protein
BVY	bictegravir/emtricitabine/tenofovir alafenamide (coformulated; Biktarvy®)
CAI	capsid inhibitor
CD4	clusters of differentiation 4
CFR	Code of Federal Regulations
CI	confidence interval
CK	creatine kinase
CL_{cr}	creatinine clearance
C_{max}	maximum observed concentration of drug
COVID-19	coronavirus disease 2019
CRO	contract research organization
CSR	clinical study report
C_{trough}	concentration at the end of the dosing interval
CYP	cytochrome P450
DAIDS	Division of AIDS
DMC	data monitoring committee
DNA	deoxyribonucleic acid
DVY	emtricitabine/tenofovir alafenamide (coformulated; Descovy®)
ECG	electrocardiogram
eCRF	electronic case report form
EDC	electronic data capture
EU	European Union
EU CT	European Union Clinical Trials Database
EQ-5D-5L	EuroQol (5 dimensions, 5 levels) questionnaire
FAS	Full Analysis Set
FDA	Food and Drug Administration
FSH	follicle-stimulating hormone
FTC	emtricitabine
GCP	Good Clinical Practice
GFR	glomerular filtration rate

Gilead	Gilead Sciences, Inc.
GPSP	Good Postmarketing Study Practice
HBV	hepatitis B virus
HCV	hepatitis C virus
HDPE	high-density polyethylene
HIV	human immunodeficiency virus
HIV-1	human immunodeficiency virus type 1
HTE	heavily treatment experienced
IB	investigator's brochure
ICH	International Council for Harmonisation (of Technical Requirements for Pharmaceuticals for Human Use)
IEC	independent ethics committee
IN	integrase
IND	investigational new drug (application)
INSTI	integrase strand-transfer inhibitor
IQ	inhibitory quotient
IRB	institutional review board
ISR	injection site reaction
IWRS	interactive web response system
LEN	lenacapavir; GS-6207
LLOQ	lower limit of quantitation
MDR	multidrug resistant
MedDRA	Medical Dictionary for Regulatory Activities
NNRTI	nonnucleoside reverse transcriptase inhibitor
NRTI	nucleoside reverse transcriptase inhibitor
OBR	Optimized Background Regimen
$paEC_{95}$	protein-adjusted effective concentration at 95%
P-gp	P-glycoprotein
PI	protease inhibitor
PK	pharmacokinetic(s)
POC	proof of concept
PR	protease
PS	Patient Safety
PT	preferred term
PWH	people with HIV
Q1	first quartile
Q3	third quartile
RNA	ribonucleic acid
RT	reverse transcriptase
SAE	serious adverse event

SC	subcutaneous
SOC	system organ class
SOP	standard operating procedure
SUSAR	suspected unexpected serious adverse reaction
SVR	suboptimal virologic response
TE	treatment experienced
TEAE	treatment-emergent adverse event
T_{max}	the time (observed time point) of C_{max}
TN	treatment-naive
UGT1A1	uridine diphosphate glucuronosyltransferase 1A1
ULN	upper limit of normal
US, USA	United States, United States of America
VR	virologic rebound

1. INTRODUCTION

1.1. Background

Human immunodeficiency virus (HIV) type 1 (HIV-1) infection is a life-threatening and serious disease of major public health significance, with approximately 37 million people with HIV (PWH) worldwide and approximately 16 million on antiretroviral (ARV) treatment {UNAIDS 2022}. Advances in combination antiretroviral therapy (ART) for HIV have led to significant improvements in morbidity and mortality by suppressing viral replication, preserving immunologic function, and averting disease progression to AIDS. Standard-of-care for the treatment of HIV-1 infection involves the use of a combination of oral ARV drugs (eg, 2 nucleoside reverse transcriptase inhibitors [NRTIs] plus a third agent) to suppress viral replication to below detectable limits, increase CD4+ cell counts, and delay disease progression.

While combination ART for the treatment of HIV-1 infection is efficacious and well tolerated, these agents need to be taken every day and require near perfect adherence to minimize the emergence of drug resistant variants. In addition, "treatment fatigue" can occur, defined as "decreased desire and motivation to maintain vigilance in adhering to a treatment regimen" among PWH prescribed chronic or life-long treatment {Claborn 2015}, which can lead to nonadherence and treatment failure. As such, there remains a significant medical need for ARVs that can be administered less frequently (eg, long-acting drug products), thereby providing an alternative treatment option for PWH.

1.2. Lenacapavir (GS-6207)

Lenacapavir (LEN; GS-6207) is a novel, first-in-class, selective inhibitor of HIV-1 capsid function, which has potent antiviral activity, low human clearance, and physicochemical properties well suited for extended-release parenteral or oral formulations. Lenacapavir has been assigned the International Nonproprietary Name Lenacapavir (LEN).

1.2.1. General Information

For further information on LEN, please refer to the investigator's brochure (IB). Information in the IB includes:

- Nonclinical pharmacokinetic (PK) and in vitro metabolism.
- Nonclinical pharmacology and toxicology.
- Clinical experience.

1.2.2. Clinical Studies of Lenacapavir

A summary of the relevant available data from clinical studies in healthy volunteers and PWH are presented in the current edition of the IB.

1.2.2.1. GS-US-200-5709

Study GS-US-200-5709 was a Phase 1, open-label, multicohort, multidose oral and/or subcutaneous (SC) LEN study to evaluate the safety, tolerability, and PK of multiple-dose oral and SC LEN in healthy participants. A total of 76 participants were enrolled in 3 cohorts as follows:

- Cohort 1: oral LEN 600 mg (2 × 300-mg tablets) given on Days 1 and 2, oral LEN 300 mg (1 × 300-mg tablet) on Day 8, and SC LEN 927 mg on Day 15 (2 × 1.5 mL of LEN injection, 309 mg/mL sodium salt). All doses were administered under fed conditions.
- Cohort 2: SC LEN 927 mg (2 × 1.5 mL of LEN injection, 309 mg/mL sodium salt) and oral LEN 600 mg (2 × 300-mg tablets) administered on Day 1 followed by oral LEN 600 mg (2 × 300-mg tablets) on Day 2, was evaluated. All doses were administered under fed conditions.
- Cohort 3: Oral LEN 600 mg (2 × 300-mg tablets) was given twice daily for 10 days with last dose given in the morning on Day 11.

The conclusions from this study were as follows:

- Lenacapavir administered as an SC injection or as tablets was generally safe and well tolerated. All injection site reactions (ISRs) were Grade 1 or 2 in severity.
- For Cohorts 1 and 2, mean LEN concentrations and their lower bound 90% CIs exceeded inhibitory quotient (IQ) 1 (protein-adjusted effective concentration at 95% [paEC$_{95}$] from MT-4 cells; 3.87 ng/mL) within a few hours of dosing on Day 1, achieved IQ4 (15.5 ng/mL) within 2 hours after dosing on Day 2, and maintained above IQ4 through the desired dosing interval of 26 weeks after the SC dose.
- The C_{max} was achieved approximately 12 weeks and 10 weeks (median T_{max}) after the SC dose for Cohorts 1 and 2, respectively. Mean LEN C_{max} (%CV) was 58.7 ng/mL (58.1%) and 62.7 ng/mL (39.2%) for Cohorts 1 and 2, respectively.
- For the dosing interval of 26 weeks, mean estimate of $AUC_{Days\ 1\text{-}197}$ for Cohort 1 and $AUC_{Days\ 1\text{-}183}$ for Cohort 2 were 166,024 ng•h/mL and 159,097 ng•h/mL, respectively.

- In Cohort 3, LEN exhibited significant accumulation over the course of 10 days of twice daily oral dosing; C_{max} and $AUC_{0\text{-}12h}$ were 53-fold and 72-fold higher, respectively, on Day 11 compared with Day 1.

For further information, refer to the current edition of the IB.

1.2.2.2. GS-US-200-4625 (Week 52)

The study design for Study GS-US-200-4625 is described in Section 3.2. A total of 72 participants are enrolled in 2 cohorts as follows:

- Cohort 1: LEN or placebo to match 600 mg oral on Days 1 or 15 and Days 2 or 16; LEN or placebo to match 300 mg oral on Days 8 or 22.
- Cohort 2: LEN or placebo to match 600 mg oral on Days 1 or 15 and Days 2 or 16; LEN or placebo to match 300 mg oral on Days 8 or 22 in combination with Optimized Background Regimen (OBR).

Week 52 data are available from this study and the main conclusions from this study are as follows:

- Consistent with the data reported in the Week 26 interim analysis, high rates of virologic suppression continued to be maintained through Week 52. These results were consistent even in participants who had suboptimal baseline OBR (eg, low overall susceptibility score, no or 1 fully active agent, integrase strand-transfer inhibitor [INSTI] resistance, no dolutegravir or darunavir), demonstrating a clinically meaningful contribution of LEN towards virologic suppression.
- Consistent with the data reported in the Week 26 interim analysis, there were clinically meaningful increases in CD4 cell count from baseline to Week 52.
- LEN remained generally safe and well tolerated and no new safety information was identified.

For further information, refer to the current edition of the IB.

1.2.2.3. GS-US-200-4334

Study GS-US-200-4334 is an ongoing, Phase 2, randomized, open-label, active-controlled, multicenter study evaluating the safety and efficacy of LEN in combination with other ARV

agents in ARV-naive PWH. A total of 183 participants were enrolled in 1 of 4 treatment groups as follows:

- Treatment Groups 1 and 2:
  - Induction Period (Day 1 through Week 27):
    - LEN 600 mg oral (2 × 300-mg tablet) on Days 1 and 2; LEN 300 mg oral (1 × 300-mg tablet) on Day 8
    - Oral daily Descovy® (emtricitabine/tenofovir alafenamide (DVY; F/TAF 200/25 mg) from Day 1 onwards for a total of 28 weeks
    - LEN 927 mg SC (309 ng/mL; 2 × 1.5 mL) on Day 15
  - Maintenance Period (Week 28 through Week 80):
    - LEN 927 mg SC (309 ng/mL; 2 × 1.5 mL) at Week 28 and every 6 months (26 weeks) thereafter
    - Treatment Group 1: Oral daily TAF (25 mg)
    - Treatment Group 2: Oral daily bictegravir (75 mg)
- Treatment Group 3 (Day 1 through Week 80):
  - LEN 600 mg oral (2 × 300-mg tablet) on Days 1 and 2; LEN 50 mg oral (1 × 50-mg tablet) daily on Day 3 and onwards.
  - Oral daily DVY (F/TAF 200/25 mg)
- Treatment Group 4 (Day 1 through Week 80): bictegravir/emtricitabine (FTC)/tenofovir alafenamide (coformulated; Biktarvy® [BVY]) 50/200/25 mg orally daily at approximately the same time.

The conclusions from this study were as follows:

- For the primary efficacy endpoint, overall, 136 of 157 participants (86.6%) who received SC or oral LEN and 23 of 25 participants (92.0%) who received BVY had HIV-1 RNA < 50 copies/mL at Week 54.
- High rates of virologic suppression continued to be maintained through the end of the Main Phase (up to Week 80) and the Extension Phase. At Week 80, 130 of 157 participants (82.8%) who received SC or oral LEN and 23 of 25 participants (92.0%) who received BVY had HIV-1 RNA < 50 copies/mL.

- At Week 80 using the Missing = Excluded analysis, 134 of 136 participants (98.5%) who received SC or oral LEN and 24 of 24 participants (100.0%) who received BVY had HIV-1 RNA < 50 copies/mL.

- The increase from baseline in CD4 cell count continued to be maintained through the end of the Main Phase (up to Week 80) and the Extension Phase across treatment groups.

- Mean LEN concentrations and the lower bound 90% CI were consistently maintained above IQ4 (15.5 ng/mL) at steady state in both the SC LEN treatment groups (SC LEN + [DVY → TAF] and SC LEN + [DVY → BIC]) and the oral LEN + DVY treatment group.

- The safety profile of LEN was consistent with previous analyses and LEN remained generally safe and well tolerated. No participant experienced a study drug–related serious adverse event (SAE).

- All but 1 ISR were Grade 1 or 2 in severity (Grade 3 injection site nodule). There was a numerical trend towards fewer study drug–related injection site nodules and indurations over time.

For further information, refer to the current edition of the IB.

1.3. Rationale for This Study

Advances in ART have led to significant improvements in morbidity and mortality among PWH by suppressing viral replication, preserving immunologic function, and averting disease progression to AIDS. While combination ART for the treatment of HIV-1 infection has been largely successful in reducing the morbidity and mortality associated with HIV disease, there remains a significant medical need for new well-tolerated therapies that take into consideration HIV genetic variability, ARV resistance, and new options for regimen simplification.

Some treatment-experienced (TE) PWH eventually lose virologic, immunologic, or clinical benefit from their current regimens. People living with HIV with multiple prior regimen failures and significant drug resistance have limited treatment options and may be unable to achieve durable HIV viral suppression {Department of Health and Human Services (DHHS) 2013, Lundgren 2013, Thompson 2010, U. S. Department of Health and Human Services 2015, Williams 2014}. These challenges are also relevant for adolescents particularly those with perinatal HIV-1 transmission (https://www.ncbi.nlm.nih.gov/pmc/articles/PMC4901868/)

Developing safe and effective therapies for heavily treatment-experienced (HTE) PWH with multidrug–resistant (MDR) HIV remains a priority. For these individuals, newer treatments are needed to control viral replication, preserve immune function, and prevent clinical progression. In all PWH, the ideal goal of therapy remains complete and durable viral suppression.

1.4. Rationale for Dose Selection of Lenacapavir

The dose selection for LEN in this study is supported by antiviral activity, PK, and safety data from the completed Phase 1b proof-of-concept (POC) Study (GS-US-200-4072) in treatment-naive (TN) and TE but capsid inhibitor (CAI)–naive PWH, as well as PK and safety data from the 2 Phase 1 studies in healthy volunteers (Study GS-US-200-4538 and Study GS-US-200-4071).

In the completed Phase 1b POC study (GS-US-200-4072), potent antiviral activity of LEN has been demonstrated; the mean maximum HIV-1 RNA decline over 10-day monotherapy after single SC doses of 50 to 450 mg was 1.8 to 2.2 $\log_{10}$ copies/mL. All participants achieved at least 1 $\log_{10}$ copies/mL decline in their HIV-1 RNA at Day 10. Day 10 antiviral activity was comparable across a dose range of single doses of 50 to 450 mg. At these doses, mean (%CV) LEN concentrations on Day 10 were 1.1- to 9.9-fold higher (eg, IQ: 1.1-9.9) than the $paEC_{95}$ for wild-type HIV-1 ($paEC_{95}$ = 3.87 ng/mL in MT-4 cells) (see the current edition of the IB).

Phenotypic analyses of LEN susceptibility in patient derived isolates indicate that isolates from TN and HTE PWH have a similar in vitro profile, suggesting similar LEN antiviral activity should be observed in the HTE patient population compared to the TN patient population.

Based on these data, a concentration of 15.5 ng/mL (corresponding to an IQ of 4 based on $paEC_{95}$ from MT-4 cells), is anticipated to provide antiviral activity in the HTE population.

Lenacapavir formulations and doses to be evaluated in this study are informed by PK and safety data from Phase 1 Studies GS-US-200-4071 and GS-US-200-4538 in healthy volunteers. The proposed regimen targets an exposure whereby the lower bound of the 90% CI of the C_{trough} is 4-fold higher than the $paEC_{95}$ (ie, IQ4) within a few days of dosing initiation, at Day 14 (end of Functional Monotherapy Period), and at end of the dosing interval (once every 26 weeks).

As described in the IB, the LEN SC solution formulation exhibits a slow initial release necessitating an oral PK load regimen prior to the first SC injection (GS-US-200-4538 clinical study report [CSR]). In Study GS-US-200-4625, to achieve IQ4 within a few days of dosing and to maintain target concentrations through the Functional Monotherapy Period, participants will receive oral tablet doses of LEN 600 mg on Days 1 and 2 and an oral tablet dose of LEN 300 mg on Day 8.

Upon completion of the Functional Monotherapy Period, participants will receive LEN 927 mg SC (309 mg/mL), followed by SC doses of LEN 927 mg administered once every 6 months (26 weeks) ± 2 weeks from last injection, along with the OBR. This regimen is projected to achieve target exposures of LEN within a few days of initiation and to maintain them through the 6 month (26 weeks) ± 2 weeks from last injection dosing interval.

Safety data from Studies GS-US-200-4071 and GS-US-200-4538 demonstrated favorable safety and tolerability profile of LEN administered as single oral doses of up to 1800 mg, multiple oral daily doses of up to 100 mg or single SC doses of up to 927 mg. Lenacapavir exposures in this study are predicted to be within the range of those shown to be safe and well tolerated; thereby, supporting further evaluation of this regimen in this study.

1.5. Rationale for Oral Weekly Bridging of Lenacapavir for Missed SC Injection

Participants receiving SC LEN may miss their SC injection window (within 26 to 28 weeks of the previous SC LEN dose [Section 5.3]). If a missed dose is anticipated, participants may receive oral bridging until they can receive their next SC injection.

Oral bridging of LEN is supported by antiviral activity, PK, and safety data from a Phase 1b POC study (GS-US-200-4072) and 2 ongoing Phase 2 and 2/3 studies (GS-US-200-4334 and GS-US-200-4625), as well as PK and safety data from 2 Phase 1 studies in healthy volunteers (GS-US-200-4071 and GS-US-200-4333). Phenotypic analyses and PK-pharmacodynamic modeling indicate that a LEN plasma concentration of 15.5 ng/mL, corresponding to IQ of 4 or higher, would provide near maximal antiviral activity (GS-US-200-4072).

The oral bridging dose of LEN is 300 mg administered once weekly starting 26 to 28 weeks after the last LEN SC injection. This oral weekly bridging dose, even when started as late as 28 weeks after the last LEN SC injection, is predicted to immediately maintain the lower bound of the 90% CI of arithmetic mean for LEN C_{trough} above IQ4 (ie, even before reaching steady state) (Figure 1A). As long as the oral weekly bridging is initiated between 26 to 28 weeks after the last LEN SC injection, the PK profile upon resuming SC injection is predicted to be comparable with that of the prior SC dose and within the target range regardless of when SC injection is resumed (Figure 1B).

Lenacapavir has been administered orally at doses up to 1800 mg (Study GS-US-200-4071). Safety data from all completed and ongoing clinical studies indicate that LEN is generally safe and well tolerated at the intended exposures.

Figure 1. Simulated Pharmacokinetic Profile of Oral Weekly Bridging of Lenacapavir (300 mg) (A) Prior to and (B) After Resuming SC Injection

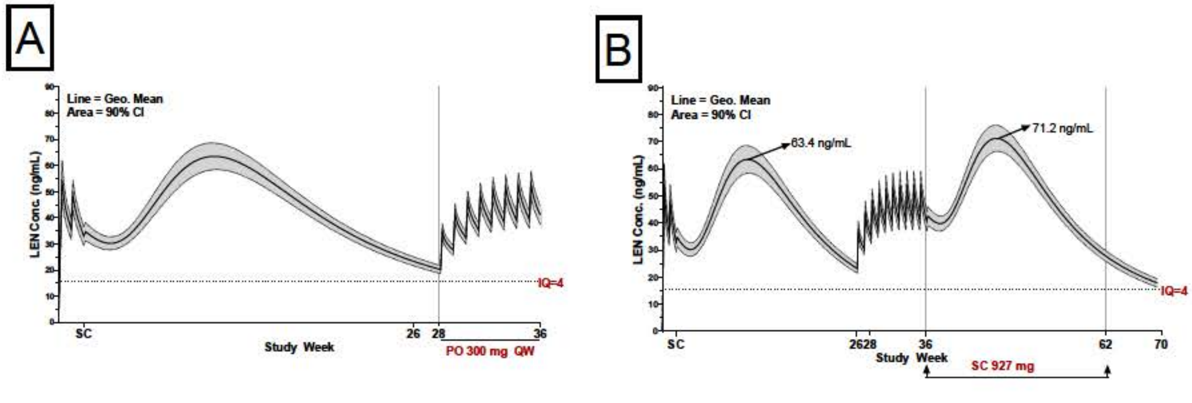

CI = confidence interval; IQ = inhibitory quotient; LEN = lenacapavir (GS-6207); PD = pharmacodynamic; PK = pharmacokinetic; PO = oral; SC = subcutaneous; QW = once weekly
The solid line and the shaded region correspond to the geometric mean and 90% CI, respectively. IQ is calculated as trough concentration/in vitro protein-adjusted EC_{95} ($paEC_{95}$) against wild-type virus. Horizontal dashed lines correspond to target IQ values of 4 based on phenotypic analyses and PK-PD modeling.

1.6. Risk/Benefit Assessment for the Study

Potential risks associated with the study include unknown adverse events (AEs), including ISRs, general risks associated with frequent clinic visits and laboratory blood draws, and the associated pain and discomfort of multiple phlebotomies. Although not specifically evaluated yet, adolescents living with HIV are expected to have similar safety profile as adult PWH; no additional safety monitoring is required for adolescent participants and no dose adjustments are required. Strategies to mitigate any potential risks include close monitoring of laboratory values as well as AEs. Parameters for monitoring of AEs will be well defined and closely followed.

In addition, potential risks to PWH include prolonged exposure to subtherapeutic concentrations of LEN if dosing is stopped which could lead to HIV-1 developing resistance to LEN. Strategies to mitigate any potential risks include initiation of an OBR within 15 days of starting LEN (Cohort 1) or co-initiation of LEN and an OBR (Cohort 2).

People with HIV are eligible for participation in this study if they are HTE with limited therapeutic options from the 4 main classes of ARV agents and inability to construct a fully active ARV regimen. Lenacapavir offers the potential benefit to construct a highly active regimen able to suppress HIV-1 replication, restoring or preserving immunologic function, and averting disease progression to AIDS. Given the above, the benefit-risk balance for this study is considered positive.

An unanticipated event such as a disaster or public health emergency may pose additional risks to study drug availability, the study visit schedule, and adherence to protocol-specified safety monitoring or laboratory assessments. Refer to Appendix 5 for further details on the risks and risk mitigation strategy.

1.7. Compliance

This study will be conducted in compliance with this protocol, Good Clinical Practice (GCP), Good Postmarketing Study Practice (GPSP), and all applicable regulatory requirements.

2. OBJECTIVES

The primary objective of this study is:

- To evaluate the antiviral activity of LEN administered as an add-on to a failing regimen (functional monotherapy) for PWH with MDR as determined by the proportion of participants achieving at least 0.5 $\log_{10}$ reduction from baseline in HIV-1 RNA at the end of the Functional Monotherapy Period.

The secondary objectives of this study are:

- To evaluate the safety and efficacy of LEN in combination with an OBR at Weeks 26 and 52.
- To evaluate the safety and efficacy of LEN in combination with an OBR at Weeks 104 and 156 from the first SC dose of LEN.

CCI [redacted]

[redacted]

[redacted]

[redacted]

[redacted]

3. STUDY DESIGN

3.1. Endpoints

The primary endpoint of this study is:

- The proportion of participants in Cohort 1 achieving ≥ 0.5 log_{10} copies/mL reduction from baseline in HIV-1 RNA at the end of Functional Monotherapy Period.

The secondary endpoints of this study are:

- The proportion of participants in Cohort 1 with plasma HIV-1 RNA < 50 copies/mL and < 200 copies/mL at Weeks 26 and 52 visits based on the United States (US) Food and Drug Administration (FDA)–defined snapshot algorithm.
- The proportion of participants in combined Cohorts 1 and 2 with plasma HIV-1 RNA < 50 copies/mL and < 200 copies/mL at Weeks 104 and 156 from the first SC dose of LEN based on the US FDA–defined snapshot algorithm.

3.2. Study Design

This is a randomized and placebo-controlled multicenter study of LEN in PWH.
Eligible participants will be enrolled in either of 2 cohorts.

Participants who complete a screening visit will return to the clinic between 14 and 30 days after the screening visit, for a Cohort Selection visit. HIV-1 RNA results from this Cohort Selection visit will be used to randomize the participant in Cohort 1 or enroll them in Cohort 2.
Once enrollment in Cohort 1 is complete, the Cohort Selection visit will not be required.

Cohort 1 (n = 36)

Functional Monotherapy Period

Eligible participants with both < 0.5 log_{10} HIV-1 RNA decline compared to the screening visit and HIV-1 RNA ≥ 400 copies/mL at the Cohort Selection visit will be randomized, in a blinded fashion, in a 2:1 ratio to either receive oral LEN or placebo to match LEN for 14 days.
The sponsor, participants, and site staff will be blinded to the treatment assignment.
Functional monotherapy will be assessed while participants continue their failing regimen.

After each participant completes the Functional Monotherapy Period, their treatment assignment will be unblinded.

Maintenance Period

Participants who were randomized to receive oral LEN will receive SC LEN and initiate their OBR on Day 1 SC (14 days after the first dose of oral LEN) (**Cohort 1A**).

Participants who were randomized to receive placebo to match LEN will receive oral LEN and initiate their OBR on Day 15 (**Cohort 1B**). They will receive SC LEN at Day 1 SC (14 days after the first dose of oral LEN) while continuing their OBR.

After the Day 1 SC visit, all Cohort 1 participants will continue with study visits at Weeks 4, 10, 16, 22, 26, 36, and 52 (study visits Week X are identified by the number of weeks that have elapsed since the Day 1 SC visit when LEN is first administered by injection and excludes Oral Bridging Periods).

<u>Cohort 2 (n = 64)</u>

Oral Lead-in Period

Participants will be enrolled into Cohort 2 if Cohort 1 is fully enrolled or if they do not meet the criteria for randomization in Cohort 1 (eg, they had ≥ 0.5 $\log_{10}$ HIV-1 RNA decline compared to the screening visit and/or HIV-1 RNA < 400 copies/mL at the Cohort Selection visit). Participants will be enrolled in Cohort 2 to receive oral LEN for 14 days. Participants will initiate an OBR on Day 1.

Maintenance Period

At Day 1 SC (14 days after the first dose of oral LEN), participants will receive SC LEN and will continue their OBR. After the Day 1 SC visit, participants will continue with study visits at Weeks 4, 10, 16, 22, 26, 36, and 52 (study visits Week X are identified by the number of weeks that have elapsed since the Day 1 SC visit when LEN is first administered by injection and excludes Oral Bridging Periods).

Figure 2. Study Design Schema

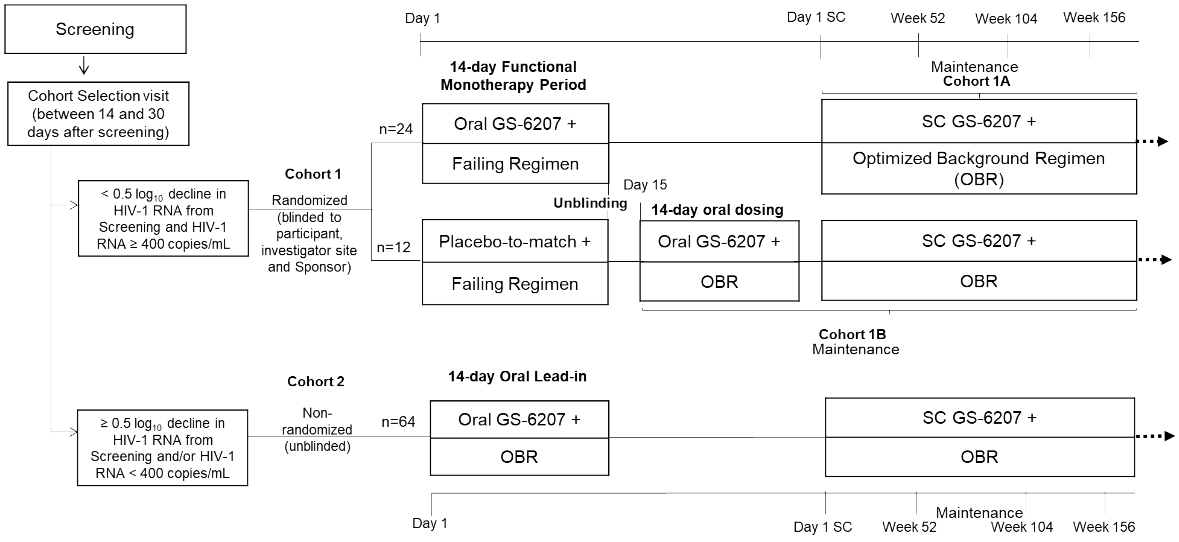

GS-6207 = lenacapavir; HIV-1 = human immunodeficiency virus type 1; OBR = Optimized Background Regimen; RNA = ribonucleic acid; SC = subcutaneous

3.3. Study Treatments

Approximately 100 PWH who meet all eligibility criteria may be enrolled in this study to receive LEN.

<u>Cohort 1:</u>

Functional Monotherapy Period: Participant will receive oral LEN 600 mg, 600 mg, and 300 mg or placebo to match LEN on Days 1, 2, and 8 respectively while continuing their failing regimen; LEN oral tablets can be administered without regards to food.

After each participant completes the Functional Monotherapy Period, their treatment assignment will be unblinded.

Maintenance Period: At Day 1 SC (14 days after the first dose of oral LEN), participants who were randomized to receive oral LEN will receive SC LEN 927 mg and initiate an OBR (as prescribed by the investigator) (**Cohort 1A**). Participants will continue to receive SC LEN 927 mg once every 6 months (26 weeks) ± 2 weeks from last injection.

At Day 15, participants who were randomized to receive placebo to match LEN will receive oral LEN 600 mg and initiate an OBR (as prescribed by the investigator). Participants will receive oral LEN 600 mg and 300 mg at Day 16 and Day 22, respectively. At Day 1 SC (14 days after the first dose of oral LEN), participants will receive SC LEN 927 mg while continuing their OBR (**Cohort 1B**). Participants will continue to receive SC LEN 927 mg once every 6 months (26 weeks) ± 2 weeks from last injection.

<u>Cohort 2:</u>

Oral Lead-in Period:

At Day 1, participants will receive oral LEN 600 mg and initiate an OBR (as prescribed by the investigator). Participants will receive oral LEN 600 mg and 300 mg at Day 2 and Day 8, respectively, while continuing their OBR; LEN oral tablets can be administered without regards to food.

Maintenance Period:

At Day 1 SC, participants will receive SC LEN 927 mg while continuing their OBR. Participants will continue to receive SC LEN 927 mg once every 6 months (26 weeks) ± 2 weeks from last injection.

Cohort 1 and Cohort 2

Participants may require oral bridging if an SC injection of LEN cannot be administered within the protocol visit window. In case of Oral Bridging initiation, participants will receive oral LEN as specified in Section 5.4 and complete Oral Bridging visits. An Oral Bridging visit should be completed every 10 to 12 weeks. Additional visits during the oral weekly administration may be conducted per investigator's discretion. Upon restarting LEN SC injection, an SC dosing visit will restart (eg, Week 52, Week 78), with follow-up visits in between occurring as specified in this protocol.

3.4. Duration of Treatment

Participants will be treated for at least 54 weeks. Following completion of the Functional Monotherapy Period (Cohort 1) or the oral lead-in Period (Cohort 2), participants will continue with study visits at Day 1 SC, Weeks 4, 10, 16, 22, 26, 36, and 52.

At the Week 52 visit, participants will be given the option to attend visits at Week 62, 78, 88, 104, 114, 130 and will continue to alternate between every 10 weeks and every 16 weeks.

At Week 156 and thereafter, upon regulatory and/or institutional review board (IRB)/independent ethics committee (IEC) approval of the protocol and when participants have provided written informed consent of the most current IRB-approved consent form, participants will continue to receive study drug every 26 weeks, and continue to attend visits every 26 weeks (± 2 weeks). Participants will receive SC LEN every 6 months (26 weeks) ± 2 weeks from the last injection starting at the Week 156 visit, while continuing their OBR, until the product becomes accessible to participants through an access program, is commercially available, or until Gilead elects to discontinue the study in the country.

Participants who decide not to receive SC LEN at Week 156 and not to continue the study will complete the study at Week 156 visit. Participants who decide to discontinue SC LEN early and do not wish to continue to attend study visits through Week 156 visit or next scheduled SC dosing visit will return to the clinic for 30-day, 90-day and 180-day follow-up visits after early termination visit. The 180-day follow-up may be conducted via a phone call per the investigator's discretion.

3.5. Biomarker Testing

CCI [redacted]

[redacted]

[redacted]

[redacted]

[redacted].

4. PARTICIPANT POPULATION

4.1. Number of Participants and Participant Selection

Approximately 100 participants may be enrolled in this study. Thirty-six participants will be enrolled in Cohort 1, and up to 64 participants may be enrolled in Cohort 2.

4.2. Inclusion Criteria

Participants must meet all of the following inclusion criteria to be eligible for participation in this study:

1) Willing and able to provide written informed consent (participants ≥ 18 years of age) and assent (participants ≥ 12 and < 18 years of age) prior to performing study procedures. For participants ≥ 12 and < 18 years of age, parent or legal guardian willing and able to provide written informed consent prior to performing study procedures as required by local law.

2) Adult aged ≥ 18 years (at all sites) or adolescent aged ≥ 12 and weighing ≥ 35 kg (at sites in North America and Dominican Republic).

3) Are receiving a stable failing ARV regimen for > 8 weeks before screening and willing to continue the regimen until Day 1. Participants in Cohort 1 must also be willing to continue the failing regimen until completing the Functional Monotherapy Period (Day 1 to Day 14).

4) Have HIV-1 RNA ≥ 400 copies/mL at screening.

5) Have screening or available historical HIV resistance reports showing resistance to ≥ 2 ARV medications from each of ≥ 3 of the 4 main classes of ARV medications (NRTI, nonnucleoside reverse transcriptase inhibitor [NNRTI], protease inhibitor [PI], INSTI). Resistance to FTC or lamivudine (3TC) associated with the presence of the M184V/I reverse transcriptase (RT) mutation cannot be used for the purpose of determining this eligibility criterion.

6) Have ≤ 2 fully active ARV remaining from the 4 main classes that can be effectively combined to form a viable regimen in the opinion of the investigator based on resistance, tolerability, contraindication, safety, drug access, or acceptability to the participant. Refer to Table 4 for list of disallowed ARVs.

7) Able and willing to receive an OBR together with LEN. Participants with an OBR without a fully active agent may be enrolled if the investigator considers that there is a favorable risk-benefit ratio for the participant. With prior approval from Gilead, components of the OBR may be investigational (ie, not yet approved).

8) A negative serum pregnancy test is required for all women at screening.

9) Participants of childbearing potential who engage in heterosexual intercourse must agree to use protocol-specified method(s) of contraception as described in Appendix 4..

10) Lactating women must agree to discontinue nursing before administration of LEN.

4.3. Exclusion Criteria

Participants who meet *any* of the following exclusion criteria are not to be enrolled in this study:

1) An opportunistic illness requiring acute therapy within the 30 days prior to screening.

2) Active, serious infections (other than HIV-1 infection) requiring parenteral antibiotic or antifungal therapy within 30 days before screening.

3) Active tuberculosis infection.

4) Acute hepatitis within 30 days prior to screening visit.

5) Untreated or newly treated (< 3 months prior to screening) hepatitis B virus (HBV) infection. Participants may be enrolled regardless of the HBV serology criteria below if they are receiving treatment with anti-HBV activity and plan to continue the treatment during the study. Hepatitis B infection is defined as screening results showing either or both of:

   a. Positive HBV surface antigen.

   b. Positive HBV core antibody and negative HBV surface antibody. Participants may be enrolled with these findings if they have HBV DNA less than lower limit of quantitation (LLOQ).

6) Hepatitis C virus (HCV) antibody positive and HCV RNA greater than LLOQ.

7) A history of or current clinical decompensated liver cirrhosis (eg, ascites, encephalopathy, or variceal bleeding).

8) Treatment within 3 months prior to screening, or anticipated treatment during the study period with immunosuppressant therapies, hydroxyurea, foscarnet, radiation, or cytotoxic chemotherapeutic agents without prior approval from sponsor prior to randomization. Agents disallowed in Table 4 may not be considered for approval.

9) Active malignancy requiring acute therapy (with the exception of local cutaneous Kaposi's sarcoma).

10) Current alcohol or substance use judged by the investigator to potentially interfere with the participant's study compliance.

11) Clinically significant abnormal electrocardiogram (ECG) at the screening visit.

12) Any of the following laboratory values at screening:

a. Estimated glomerular filtration rate (GFR) ≤ 50 mL/min using Cockcroft-Gault formula for participants ≥ 18 years of age {Cockcroft 1976} and Schwartz Formula for participants < 18 years of age for creatinine clearance (CL_{cr}).

b. Alanine aminotransferase (ALT) > 5 × upper limit of normal (ULN).

c. Direct bilirubin > 1.5 × ULN.

d. Platelets < 50,000/mm^3.

e. Hemoglobin < 8.0 g/dL.

13) Participation or planned participation in any other clinical study (including observational studies) without prior approval from the sponsor throughout the study.

14) Prior use of, or exposure to, LEN.

15) Known hypersensitivity to the study drug, the metabolites, or formulation excipient.

16) Use or planned use of exclusionary medications, refer to Section 5.5.

17) Any other clinical condition or prior therapy that, in the opinion of the investigator, would make the participant unsuitable for the study or unable to comply with dosing requirements.

5. INVESTIGATIONAL MEDICINAL PRODUCTS

5.1. Randomization, Enrollment, Blinding, and Treatment Codes Access

5.1.1. Randomization and Enrollment

Participants will be assigned a screening number using the interactive web response system (IWRS) on the day of the screening visit. Once eligibility has been confirmed and availability of OBR is confirmed, participants will be enrolled to either of the 2 cohorts as following:

- Cohort 1: Randomized in a 2:1 ratio to receive oral LEN or placebo to match oral LEN for 14 days starting on Day 1, while they continue their existing regimen

  OR

- Cohort 2: Enrolled to receive oral LEN together with an OBR.

Randomization or enrollment may occur approximately 3 days prior to Day 1 visit.

Each eligible participant will be assigned a unique participant number using IWRS. Once a participant number has been assigned, it will not be reassigned to another participant.

5.1.2. Blinding

During the Functional Monotherapy Period in Cohort 1, the investigational site(s), participants, and Gilead will remain blinded to treatment assignment and HIV-1 RNA results at Days 2 and 8. As each participant completes the Functional Monotherapy Period, their treatment assignment will be unblinded by investigational site using IWRS to determine their treatment regimen in the Maintenance Period. To mitigate the risks of inadvertently releasing the treatment information to participants who are still receiving functional monotherapy, Gilead staff will not receive the treatment codes from IWRS until all participants in Cohort 1 have completed the Functional Monotherapy Period. In the situation when Gilead staff is able to identify possible treatment assignment of participants still on blinded treatment, based on treatment information (eg, randomization block) from individual participants that have already been unblinded, they will maintain the confidentiality of the unblinded information, and will not communicate the information to blinded sites as specified in Gilead procedural documents.

Furthermore, specified personnel may be unblinded based on their study role. The Pharmacokinetics File Administrator, or designee, in Bioanalytical Operations and/or Clinical Data Management who facilitates the data transfer of PK files between Gilead and vendors will remain unblinded. Individuals in Clinical Packaging and Labeling or Clinical Supply Management who have an Unblinded Inventory Manager role in the IWRS for purposes of study drug inventory management will remain unblinded. Individuals in Gilead Patient Safety (PS) responsible for safety signal detection, investigational new drug (IND) safety reporting, and/or expedited reporting of suspected unexpected serious adverse reactions

(SUSARs) may be unblinded to individual case data and/or group-level summaries. External (ie, contract research organizations [CROs]) biostatisticians and programmers will be unblinded to support safety data review. Quality personnel in Research and Development Quality (R&D Quality) may also be unblinded for purposes of supporting Quality Assurance activities and/or regulatory agency inspections.

5.1.3. Procedures for Breaking Treatment Codes

In the event of a medical emergency where breaking the blind is required to provide medical care to the participant, the investigator may obtain treatment assignment directly from the IWRS system for that participant. Gilead recommends but does not require that the investigator contact the Gilead medical monitor before breaking the blind. Treatment assignment should remain blinded unless that knowledge is necessary to determine participant emergency medical care. The rationale for unblinding must be clearly explained in source documentation and on the electronic case report form (eCRF), along with the date on which the treatment assignment was obtained. The investigator is requested to contact the Gilead medical monitor promptly in case of any treatment unblinding.

All participants will be followed until study completion, unless consent to do so is specially withdrawn by the participant.

5.2. Description and Handling of Lenacapavir Injection, Lenacapavir Tablets, and Placebo to Match Lenacapavir Tablets

5.2.1. Formulation

Lenacapavir Injection, 309 mg/mL, is a clear, yellow to brown solution for SC injection. In addition to the active ingredient (LEN sodium salt), LEN Injection, 309 mg/mL contains the following inactive ingredients: polyethylene glycol 300 and water for injection.

Lenacapavir tablets, 300 mg are capsule-shaped, film-coated beige tablets, debossed with “GSI” on one side of the tablet and “62L” on the other side of the tablet. Each tablet core contains the equivalent of 300 mg LEN free acid in the form of LEN sodium salt. In addition to the active ingredient, LEN tablets, 300 mg contain the following inactive ingredients: microcrystalline cellulose, mannitol, poloxamer 407, copovidone, croscarmellose sodium, magnesium stearate, polyethylene glycol, polyvinyl alcohol, talc, titanium dioxide, iron oxide red, iron oxide black, and iron oxide yellow.

Placebo to match LEN tablets, 300 mg, are capsule-shaped, film-coated beige tablets, debossed with “GSI” on one side of the tablet and “62L” on the other side of the tablet. Placebo to match LEN tablets, 300 mg contain the following inactive ingredients: lactose, microcrystalline cellulose, croscarmellose sodium, magnesium stearate, polyethylene glycol, polyvinyl alcohol, talc and titanium dioxide, iron oxide red, iron oxide black, and iron oxide yellow.

5.2.2. Packaging and Labeling

Lenacapavir injection, 309 mg/mL, is supplied as a sterile solution packaged in a single use, clear vial fitted with a rubber stopper and an aluminum flip-off seal.

Lenacapavir tablets, 300 mg are packaged in white, high-density polyethylene (HDPE) bottles. Each bottle contains 4 or 5 tablets, silica gel desiccant, and polyester packing material. Each bottle is enclosed with a white, continuous thread, child-resistant polypropylene screw cap fitted with an induction-sealed, and aluminum-faced liner.

Placebo to match LEN tablets, 300 mg are packaged in white, HDPE bottles. Each bottle contains 5 tablets, silica gel desiccant, and polyester packing material. Each bottle is enclosed with a white, continuous thread, child-resistant polypropylene screw cap fitted with an induction-sealed, and aluminum-faced liner.

Study drugs to be distributed to centers in the US, European Union (EU) and other participating countries shall be labeled to meet applicable requirements of the US FDA, EU Guideline to Good Manufacturing Practice - Annex 13 (Investigational Medicinal Products), the J-GCP (Ministerial Ordinance on Good Clinical Practice for Drugs) and/or other local regulations.

5.2.3. Storage and Handling

Lenacapavir injection, 309 mg/mL should be stored below 30 °C (86 °F), protected from light. Storage conditions are specified on the label.

Lenacapavir tablets, 300 mg should be stored below 30 °C (86 °F). Storage conditions are specified on the label. Placebo to match LEN tablets, 300 mg should be stored below 30 °C (86 °F). Storage conditions are specified on the label.

Until dispensed to the participants, all study drugs should be stored in a securely locked area, accessible only to authorized site personnel. To ensure the stability and proper identification, study drugs should not be stored in a container other than the container in which they were supplied. Consideration should be given to handling, preparation, and disposal through measures that minimize drug contact with the body. Appropriate precautions should be followed to avoid direct eye contact or exposure when handling.

5.3. Dosage and Administration of Lenacapavir

Oral LEN and LEN injection for SC administration will be provided by Gilead. Participants will be responsible for continuing their pre-existing regimen. The OBR will be prescribed by the investigator, and the participant is responsible for obtaining the OBR prior to their Day 1 visit. Medications disallowed as components of the OBR for PK reasons are provided in Section 5.5. Investigators should confirm the availability of the OBR prior to randomizing or enrolling eligible participants in IWRS.

Oral LEN or placebo to match LEN will be administered on Days 1, 2, and 8. Participants who receive placebo to match LEN on Days 1, 2, and 8 will receive oral LEN on Days 15, 16, and 22. Oral LEN will be administered without regard to food. Oral dosing is presented in Table 1.

Table 1. Oral Dosing of Lenacapavir or Placebo to Match

Study Visits	Treatment
Days 1 or 15[a]	600 mg
Days 2 or 16[a]	600 mg
Days 8 or 22[a]	300 mg

a Days 15, 16 and 22 visits are applicable only to participants who receive placebo to match in the Functional Monotherapy Period at Days 1, 2, and 8.

Lenacapavir 927 mg injection, 309 mg/mL will be administered in the abdomen via SC injections on Day 1 SC, Weeks 26, 52, and every 6 months (26 weeks) ± 2 weeks from last injection thereafter. Lenacapavir injections should be administered at different abdominal sites no more than 15 minutes apart when possible. Each SC LEN dosing should occur 6 months (26 weeks) ± 2 weeks from the last injection.

The entire initial oral lead-in period and all subsequent SC LEN administration will occur on-site (for Oral Bridging Period, see Section 5.4).

Study drug (oral and SC LEN) can be administered without regard to food.

If a participant is not dosed within the protocol visit windows, and the investigator believes that it is in the participant's medical interest to continue to receive LEN, Gilead should be contacted immediately, and the participant may continue to receive LEN with the approval of the Gilead medical monitor. Additional oral LEN doses may be needed prior to subsequent SC dosing (Section 5.4).

For all participants, the date and time of last meal prior to dosing will be collected.

5.4. Dosing and Administration of Oral Weekly Bridging of Lenacapavir

Participants may require oral weekly bridging if an SC injection of LEN cannot be administered for any reason within the protocol visit window.

- If 26 to 28 weeks elapses since the last SC injection and if clinically appropriate to continue LEN, start oral LEN 300 mg (1 tablet) once a week at Oral Bridging visit(s) and continue weekly dosing on the same day of the week.
- If more than 28 weeks elapse since the last SC injection and if clinically appropriate to continue LEN, restart the oral lead-in at the Oral Bridging visits and continue oral weekly dosing on the same day of the week, until SC LEN is administered (Table 2).

Table 2. Dosing Schedule for Oral Weekly Bridging and Resumption of SC Lenacapavir Injection

26 to 28 weeks of elapse since the last SC injection	More than 28 weeks of elapse since the last SC injection	LEN Dose
**Treatment time from oral start**	**Dose**	—
NA	Day 1[a]	Oral 600 mg (2 × 300 mg tablets)
NA	Day 2	Oral 600 mg (2 × 300 mg tablets)
Day 1[a]	Day 8; continue oral weekly LEN on the same day of the week as first dose until LEN SC is administered	Oral 300 mg (1 × 300 mg tablet)
Day 8; continue oral weekly LEN on the same day of the week as first dose until LEN SC is administered	NA	Oral 300 mg (1 × 300 mg tablet) once a week
SC administration restart (within 1 week of the oral weekly 300 mg dose [as early as after Day 1 dosing])	SC administration restart (within 1 week of the oral weekly 300 mg dose [as early as after Day 8 dosing])	SC 927 mg every 6 months[a]

LEN = lenacapavir; NA = not applicable; SC = subcutaneous

a Day 1 oral dosing and SC LEN will be administered on site, otherwise, all other oral dosing will be administered at home, but can be done on site at the investigator's discretion.

- For oral weekly bridging, the administration of the first dose will occur on-site and up to 2 bottles of LEN will be given to the participant to take oral LEN at home; further on-site oral dosing may occur at investigator's discretion. Up to 2 additional bottles may be given to the participant as needed upon completion of previously dispensed bottles.

- Participants may continue receiving oral weekly LEN 300 mg until they can receive their next SC LEN injection.

- If oral weekly LEN administration is expected to be needed beyond 12 consecutive weeks, Gilead should be contacted. Additional oral weekly doses can be provided after assessment unless other guidance is provided by Gilead.

- Monitoring of participants should continue according to the study protocol. Oral Bridging visit should be completed every 10 to 12 weeks. Additional visits during the oral weekly administration may be conducted per investigator's discretion.

- Missed dose recommendations for oral weekly bridging are provided in Table 3.
  The scheduled dosing day of the week should not change due to the missed dose of LEN.

Table 3. Missed Dose Recommendations for Lenacapavir During Oral Weekly Bridging

Number of days since initial missed schedule dose	Recommendation	Example
1 to 6 days<br>(1 missed dose)	Take 1 dose as soon as possible, then resume normal schedule, taking 1 dose on the next scheduled day	Participant forgets to take dose on Monday (scheduled) but remembers before the next scheduled dose day (ie, Tuesday-Sunday). Take 1 dose as soon as possible, then take 1 dose on the following Monday as scheduled.
7 to 14 days<br>(1-2 missed doses)	Take 2 doses as soon as possible, then resume normal schedule on scheduled day. If participant remembers on scheduled dosing day, then take 2 doses only. Never take 3 doses on the same day.	Participant forgets to take dose on Monday (scheduled) but remembers the following Monday. Take 2 doses on the second Monday and resume dosing schedule (1 dose on Mondays).<br>Participant forgets to take dose on 2 consecutive Mondays (scheduled) but remembers a few days later (ie, Tuesday-Sunday following second missed dose) before the third Monday. Take 2 doses as soon as possible, then take 1 dose the next Monday as scheduled.<br>Participant forgets to take dose on 2 consecutive Mondays (scheduled) but remembers on the third Monday. Take 2 doses on the third Monday and resume dosing schedule (1 dose on Mondays). Never take 3 doses on the same day.
More than 14 days<br>(3 or more missed doses)	Assess whether clinically appropriate to continue oral weekly bridging.<br>Consider checking HIV-1 RNA.	Participant forgets to take dose on 3 consecutive Mondays (scheduled). Clinical assessment needed.

HIV-1 = human immunodeficiency virus type 1; RNA = ribonucleic acid

5.5. Prior and Concomitant Medications

Clinical data indicate LEN is a substrate of P-glycoprotein (P-gp) transporters, cytochrome P450 (CYP)3A and uridine diphosphate glucuronosyltransferase 1A1 (UGT1A1) enzymes. Lenacapavir is a moderate inhibitor of CYP3A and a weak inhibitor of breast cancer resistance protein (BCRP), and P-gp. Concomitant use of LEN with some medications or herbal/natural supplements that are inhibitors and inducers of CYP3A, UGT1A1, or P-gp may result in increased or decreased exposure of LEN, respectively.

Concomitant use of LEN with some medications or herbal/natural supplements that are substrates of CYP3A, P-gp, or BCRP may result in increased exposure of these medications.

Representative medications listed in Table 4 and herbal/natural supplements are currently excluded or should be used with caution while participating in this study; this table is not exhaustive. For medications that may be substrates of P-gp, CYP3A, UGT1A1, or BCRP, or those that may be inducers, the investigator should reach out to Gilead for guidance.

Participants should discontinue disallowed concomitant medications 30 days prior to initiation of study drug, unless otherwise specified.

Table 4. List of Representative Medications That are Prohibited or To Be Used With Caution Due to the Potential for Drug-Drug Interaction With Lenacapavir

Medication Class	Disallowed Medications	Use Discouraged and To Be Used With Caution
Anticoagulants	—	Dabigatran etexilate: monitoring and/or dose reduction may be needed for certain populations per prescribing information
Anticonvulsants	Carbamazepine, Oxcarbazepine, Phenobarbital, Phenytoin	—
Antimycobacterials	Rifampin, Rifabutin, Rifapentine[a]	—
Antiretroviral agents	ATV, ATV/co[a], ATV/r, EFV, ETV, NVP, TPV[a]	—
Digoxin	—	Digoxin: Concomitant use of oral LEN may result in increased levels; use with caution and with appropriate monitoring of serum digoxin levels
Ergot derivatives	Ergotamine, Ergonovine, Dihydroergotamine, Methylergonovine, Ergometrine	—
Herbal/Natural Supplements	St. John’s Wort, Echinacea, Milk thistle (eg, silymarin), Chinese herb sho-saiko-to (or Xiao-Shai-Hu-Tang)	—
HMG-CoA Reductase Inhibitors	—	Concentrations of statins may increase with LEN. Start with the lowest dose and titrate to clinical response. For each of the following statins, the maximum allowed dose is:<br>Simvastatin: 10 mg<br>Lovastatin[a]: 20 mg<br><br>Careful monitoring for signs and symptoms of muscle weakness or myopathy, including rhabdomyolysis

Medication Class	Disallowed Medications	Use Discouraged and To Be Used With Caution
Phosphodiesterase-5 Inhibitors	—	Sildenafil, Vardenafil, Tadalafil: It is recommended that a single dose of Sildenafil no more than 25 mg in 48 hours, Vardenafil no more than 2.5 mg in 72 hours, or Tadalafil no more than 10 mg in 72 hours be coadministered.<br>Use of tadalafil for the treatment of pulmonary arterial<br>hypertension is not recommended
Sedatives/Hypnotics	—	Midazolam, Triazolam
Systemic Corticosteroids	Use of all agents for > 7 days is prohibited.	—

ATV = atazanavir; co = cobicistat; EFV = efavirenz; ETV = etravirine; HMG-CoA = 3-hydroxy-3-methylglutaryl coenzyme A; LEN = lenacapavir; NVP = nevirapine; r = ritonavir; TPV = tipranavir

a Not approved in Japan.

Physicians should refer to the package insert for the OBR medications for guidance on concomitant medications.

Investigators should make every effort to maintain the initial OBR. However, if clinically indicated for efficacy, safety, or tolerability reasons, investigators may change the OBR after consulting with Gilead's medical monitor. The reason for any changes to the OBR will be documented in the participant's source documents and the eCRF.

Medications to treat disease conditions **excluded** from the protocol are not listed under this concomitant medication section and are disallowed in the study. Medications for malignancy are not included.

Should participants have a need to initiate treatment with any disallowed concomitant medication, the medical monitor must be consulted prior to initiation of the new medication. In instances where disallowed medication is initiated prior to discussion with the sponsor, the investigator must notify Gilead as soon as they are aware of the use of the medication.

5.6. Accountability for Study Drug

The investigator (or designee, eg, study center pharmacist) is responsible for ensuring adequate accountability of all used and unused study drug. This includes acknowledgment of receipt of each shipment of study drug (quantity and condition). Each study site must keep accountability records that capture:

- The date received and quantity of study drug kits.
- The date, participant number, and the study drug kit number dispensed.
- The date, quantity of used and unused study drug returned, along with the initials of the person recording the information.

5.6.1. Study Drug Return or Disposal for Lenacapavir and Placebo to Match Lenacapavir Tablets

Gilead recommends that used and unused study drug supplies be destroyed at the site. If the site has an appropriate standard operating procedure (SOP) for drug destruction as determined by Gilead, the site may destroy used (empty or partially empty) and unused study drug supplies in accordance with that site's approved SOP. A copy of the site's approved SOP will be obtained for electronic Trial Master File. If study drug is destroyed on site, the investigator must maintain accurate records for all study drugs destroyed. Records must show the identification and quantity of each unit destroyed, the method of destruction, and the person who disposed of the study drug. Upon study completion, copies of the study drug accountability records must be filed at the site. Another copy will be returned to Gilead.

If the site does not have an appropriate SOP for drug destruction, used and unused study drug supplies are to be sent to the designated disposal facility for destruction. The study monitor will provide instructions for return.

The study monitor will review study drug supplies and associated records at periodic intervals.

For both disposal options listed above, the study monitor must first perform drug accountability during an on-site monitoring visit, unless otherwise agreed with the sponsor and documented.

6. STUDY PROCEDURES

The study procedures to be conducted for each participant enrolled in the study are presented in tabular form in Appendix 2. and described in the text that follows.

The investigator must document any deviation from the protocol procedures and notify the Gilead or CRO.

6.1. Participant Enrollment and Treatment Assignment

Entry into screening does not guarantee enrollment into the study. In order to manage the total study enrollment, Gilead, at its sole discretion, may suspend screening and/or enrollment at any site or study-wide at any time.

6.2. Pretreatment Assessments

6.2.1. Screening Assessments

Participants will be screened within 42 days prior to enrollment in the study. Each participant will be assigned a unique screening number using the IWRS.

The following will be performed and documented at screening:

- Obtain written informed consent–related events, available historical genotype/phenotype reports, available HIV-1 treatment history, substance (ie, illicit drug) use and prior medications within 30 days of the screening visit.
- Complete physical examination (urogenital/anorectal examinations will be performed at the discretion of the investigator) including, vital signs, body weight, and height.
- 12-lead ECG performed supine.
- Obtain blood and urine samples as noted in Section 6.5.
- Record any SAEs and all AEs related to protocol-mandated procedures occurring after signing of the consent form.
- Select an OBR based on the screening and/or available historical HIV resistance reports.
    - Availability of selected OBR must be confirmed prior to randomization/enrollment and participant must be able to initiate OBR as specified in the protocol.
- Return to the clinic between 14 and 30 days after the screening visit, for a Cohort Selection visit. HIV-1 RNA results from this visit will be used to determine whether eligible participants will participate in Cohort 1 or Cohort 2.
    - Once enrollment in Cohort 1 is complete, Cohort Selection visit will not be required.

Participants meeting all the inclusion criteria and none of the exclusion criteria will return to the clinic within 42 days after screening for a Day 1 visit. Participants will be instructed to continue their failing regimen.

Participants not meeting one or more inclusion criteria and/or meeting one or more exclusion criteria may be rescreened on a case-by-case basis upon written approval from Gilead medical monitor or study director.

From the time of obtaining informed consent through the first administration of study drug, record all SAEs, as well as any AEs related to protocol-mandated procedures on the AEs eCRF. All other untoward medical occurrences observed during the screening period, including exacerbation or changes in medical history are to be considered medical history. See Section 7 Adverse Events and Toxicity Management for additional details.

6.3. Treatment Assessments

6.3.1. Functional Monotherapy Period and Oral Lead-in Period Assessments

Cohort 1: Eligible participants will be randomized in Cohort 1 if they have a < 0.5 $\log_{10}$ HIV-1 RNA decline compared to the screening visit and HIV-1 RNA ≥ 400 copies/mL at the Cohort Selection visit. Approximately 3 days prior to the Day 1 visit, investigator or designee may randomize the participant to receive oral LEN or placebo to match LEN and obtain a participant number using IWRS. Cohort 1 participants will have a Functional Monotherapy Period. Participants will continue to receive their failing regimen during this period.

Cohort 2: Eligible participants will be enrolled in Cohort 2 if Cohort 1 is fully enrolled or if they do not meet the criteria for randomization in Cohort 1 (eg, they had a ≥ 0.5 $\log_{10}$ HIV-1 RNA decline compared to the screening visit and/or HIV-1 RNA < 400 copies/mL at the Cohort Selection visit). Approximately 3 days prior to the Day 1 visit, investigator or designee may enroll the participant using IWRS and obtain a participant number. Cohort 2 participants will have an oral lead-in period and will initiate an OBR (as prescribed by the investigator) on Day 1.

All participants will complete the following study visits: Days 1, 2, 5 (± 1 day) CCI [redacted]. The following procedures are to be completed at the Day 1 visit, prior to study drug dosing and at Days 2, 5 (± 1 day) and 8, unless otherwise noted.

- Prior to completion of other study procedures, participants ≥ 18 years of age at the Day 1 visit will read the Patient Reported Outcomes, if available, by himself/herself and provide answers directly onto the questionnaires.
  - Symptoms Distress Module, Short Form Health Survey (SF-36), EQ-5D-5L at Day 1.
- Review of AEs and changes in concomitant medications.
- Complete physical examination (urogenital/anorectal examinations will be performed at the discretion of the investigator) (Day 1 only) or symptom-directed physical examination as needed at Days 2 and 8.

- Vital signs and body weight (except at Day 5).
- Obtain blood and urine samples collection as noted in Section 6.5.
- After completion of study assessments, Cohort 1 participants will receive oral LEN 600 mg, 600 mg, and 300 mg or placebo to match on Days 1, 2, and 8 respectively.
  - Cohort 1 participants will continue on their failing regimen.
- After completion of study assessments, Cohort 2 participants will receive oral LEN 600 mg, 600 mg, and 300 mg on Days 1, 2, and 8 respectively.
  - Cohort 2 participants will initiate an OBR (as prescribed by the investigator) at Day 1. Participants will take last dose of their failing regimen prior to Day 1 visit.
- All study drug dosing will occur at the study center without regard to food.
- Document study drug administration.
- After each participant completes the Functional Monotherapy Period, their treatment assignment will be unblinded.

6.3.2. Maintenance Period Assessments

All participants will complete a Maintenance Period. The Gilead medical monitor should be consulted prior to administration of SC LEN in the Maintenance Period if a participant was not administered all doses of oral LEN during the Functional Monotherapy Period in Cohort 1 or the Oral Lead-in Period in Cohort 2.

All participants will complete the following study visits: Day 1 SC (14 days after the first dose of oral LEN), Weeks 4, 10, 16, 22, 26, 36, and 52. At the Week 52 visit, participants will be given an option to continue receive SC LEN 927 mg and to complete study visits at Weeks 62, 78, 88, 104, 114, 130 and will continue to alternate between every 10 weeks and every 16 weeks.

At Week 156 and thereafter, upon regulatory and/or IRB/IEC approval of the protocol and when participants have provided written informed consent of the most current IRB-approved consent form, participants will continue to receive study drug every 26 weeks, and continue to attend visits every 26 weeks (± 2 weeks). Participants will receive SC LEN every 6 months (26 weeks) ± 2 weeks from the last injection starting at the Week 156 visit, while continuing their OBR, until the product becomes accessible to participants through an access program, is commercially available, or until Gilead elects to discontinue the study in the country.

Study visits Week X are identified by the number of weeks that have elapsed since the Day 1 SC visit when LEN is first administered by injection and excludes Oral Bridging Periods.

Cohort 1B participants will complete the following study visits prior to receiving SC LEN: Days 15, 16, 19 (± 1) CCI [redacted]. They will receive oral LEN 600 mg, 600 mg, and 300 mg on Days 15, 16, and 22, respectively.

Participants may require oral bridging if an SC injection of LEN cannot be administered within the protocol visit window. In case of Oral Bridging initiation, participants will receive oral LEN as specified in Section 5.4 and complete Oral Bridging visits. An Oral Bridging visit should be completed every 10 to 12 weeks. Additional visits during the oral weekly administration may be conducted per investigator's discretion. Upon restarting LEN SC injection, an SC dosing visit will restart (eg, Week 52, Week 78), with follow-up visits in between occurring as specified in this protocol.

Week 4 visit through Week 52 visit are to be completed within ± 2 days of the protocol-specified visit date based on the Day 1 SC visit. Following the completion of Week 52 visit, visits are to be completed within ± 6 days of the protocol-specified visit date. Each SC LEN dosing should occur within 26 and 28 weeks of the previous SC LEN dosing.

The following procedures are to be completed at all visits unless otherwise noted.

- Participants ≥ 18 years of age at the Day 1 visit will read the Patient Reported Outcomes, if available, by himself/herself and provide answers directly onto the questionnaires.
  - Participants will complete Symptoms Distress Module, Short Form Health Survey (SF-36), EQ-5D-5L at Weeks 4, 16, 26, and 52 (these questionnaires should be completed prior to the completion of other study procedures).
  - Participants to complete the Numeric Pain Rating Scale at Day 1 SC, Weeks 26 and 52 (this questionnaire should be completed after the participant received their SC LEN injections).
- Review of AEs and changes in concomitant medications.
- Complete physical examination (Day 15 [Cohort 1B only], Day 1 SC, Weeks 26 and 52) (urogenital/anorectal examinations will be performed at the discretion of the investigator) or symptom-directed physical examination as needed (except at Days 5 and 19).
- Obtain vital signs and body weight (except at Days 5 and 19).
- Obtain blood and urine samples as noted in Section 6.5.
- Cohort 1 participants who received oral LEN during randomized phase will receive SC LEN 927 mg and initiate their OBR on Day 1 SC visit (14 days after the first dose of oral LEN) (**Cohort 1A**). Participants will continue to receive SC LEN 927 mg every 6 months (26 weeks) ± 2 weeks from last injection thereafter while continuing their OBR.

- Cohort 1 participants who received placebo to match LEN during randomized phase will receive oral LEN 600 mg and initiate their OBR on the Day 15 visit after completion of Day 15 assessments (**Cohort 1B**). Participants will receive oral LEN 600 mg and 300 mg on Days 16 and 22 respectively while continuing their OBR.
- Cohort 1B participants will receive SC LEN 927 mg at the Day 1 SC visit while continuing on their OBR (14 days after the first dose of oral LEN). Participants will continue to receive SC LEN 927 mg every 6 months (26 weeks) ± 2 weeks from last injection thereafter while continuing their OBR.
- Cohort 2 participants will receive SC LEN 927 mg at the Day 1 SC visit while continuing on their OBR (14 days after the first dose of oral LEN). Participants will continue to receive SC LEN 927 mg every 6 months (26 weeks) ± 2 weeks from last injection thereafter while continuing their OBR.
- SC study drug administration will occur at the study center without regard to food.
- Provide ISR assessment worksheet and instruct the participants to measure and report ISRs following the administration of the SC injections.
- For all participants on oral weekly bridging, the first dose of oral LEN will be administered at the study center without regard to food and up to 2 bottles will be provided to the participant with instructions to take the remaining doses at home once a week (1 × 300 mg LEN) without regard to food on the same day of the week that the first dose was given.
- Document study drug administration.
- Document study drug accountability.
- Participants who meet the criteria for virologic failure will be managed according to the Management of Virologic Failure Section 6.7.

The Week 156 visit and visits every 26 weeks thereafter are to be completed within ± 2 weeks of the protocol-specified visit date. Each SC LEN dosing should occur within 26 weeks ± 2 weeks of the previous SC LEN dosing.

The following procedures are to be completed at all visits unless otherwise noted.

- Review of AEs and changes in concomitant medications.
- Symptom-directed physical examination.
- Obtain vital signs and body weight.
- Obtain blood and urine samples as noted in Section 6.5.
- SC study drug administration will occur at the study center without regard to food.

6.4. Posttreatment Assessments

6.4.1. Early Termination Visit Assessments

6.4.1.1. Early Termination Visit After Day 1 and Prior to Day 1 SC

If a participant discontinues oral LEN or decides not to initiate SC LEN at Day 1 SC, an early termination visit should be performed within 72 hours of decision to discontinue the study drug. Participants in Cohort 1 will be asked to continue to attend study visits until completion of the Functional Monotherapy Period. Participant will be required to complete the 90-day follow-up visit. Refer to Section 6.4.2.

6.4.1.2. Early Termination Visit After Day 1 SC and Prior to Week 52

If a participant decides to discontinue SC LEN prior to Week 52, an early termination visit should be performed within 72 hours of decision to discontinue study drug.

The participant will be asked to continue attending the scheduled study visits through the Week 52 visit.

- If the participant decides to discontinue SC LEN and agrees to continue to attend study visits through Week 52, no follow-up visits are required.
- If the participant decides to discontinue SC LEN and does not agree to continue to attend study visits through Week 52, the 30-day, 90-day, and 180-day follow-up visits are required. Refer to Section 6.4.2. The 180-day follow-up may be conducted via a phone call per the investigator's discretion.

6.4.1.3. Early Termination Visit After Week 52

If a participant continues the study after completing the Week 52 visit, but discontinues SC LEN prior to study completion, an early termination visit should be performed.

- If the participant discontinues LEN (SC and oral) and continues to attend study visits through their next SC dosing visit, all assessments, except SC dosing, will be performed at the last visit. No further follow-up visits are required.
- If the participant discontinues SC LEN and does not continue to attend study visits through the participant's next SC dosing visit, the 30-day, 90-day, and 180-day follow-up visits are required. Refer to Section 6.4.2. The 180-day follow-up may be conducted via a phone call per the investigator's discretion.
- If a participant discontinues oral LEN during the Oral Bridging Period and does not continue to attend study visits through their next SC dosing visit, the participant will be required to complete the 90-day follow-up visit (see Section 6.4.2).

If there are any abnormal laboratory results with a possible or probable causal relationship with the study drug, every attempt should be made to keep the participant in the study and repeat those laboratory tests weekly (or as often as deemed prudent by the investigator) until the abnormality is resolved, returns to baseline, or is otherwise explained. If this is not possible or acceptable to the participant or investigator, the participant may be withdrawn from the study.

If there are any AEs, every attempt should be made to keep the participant in the study and should be followed up until the AE is resolved or stable. Gilead may request that certain AEs be followed beyond the protocol-defined follow-up period. If this is not possible or acceptable to the participant or investigator, the participant may be withdrawn from the study.

The following evaluations should be performed at the early termination visit:

- Review of AEs and changes in concomitant medications.
- Complete physical examination (urogenital/anorectal examinations will be performed at the discretion of the investigator).
- Vital signs measurement (blood pressure, pulse, respiration rate, and temperature).
- Weight.
- Obtain blood and urine samples as noted in Section 6.5.
- Counsel participant regarding the importance of continuing a complete ART regimen in accordance to standard of care, and refer patient to an appropriate HIV treatment facility.

6.4.1.4. Early Termination Visit After Week 156

If a participant continues the study after completing the Week 156 visit, but discontinues SC LEN prior to study completion, an early termination visit should be performed.

- If the participant discontinues LEN (SC) and continues to attend study visits through their next SC dosing visit, all assessments, except SC dosing, will be performed at the last visit. No further follow-up visits are required.
- If the participant discontinues SC LEN and does not continue to attend study visits through the participant's next SC dosing visit, the 30-day, 90-day, and 180-day follow-up visits are required. Refer to Section 6.4.2. The 180-day follow-up may be conducted via a phone call per the investigator's discretion.

If there are any abnormal laboratory results with a possible or probable causal relationship with the study drug, every attempt should be made to keep the participant in the study and repeat those laboratory tests weekly (or as often as deemed prudent by the investigator) until the abnormality is resolved, returns to baseline, or is otherwise explained. If this is not possible or acceptable to the participant or investigator, the participant may be withdrawn from the study.

If there are any AEs, every attempt should be made to keep the participant in the study and should be followed up until the AE is resolved or stable. Gilead may request that certain AEs be followed beyond the protocol-defined follow-up period. If this is not possible or acceptable to the participant or investigator, the participant may be withdrawn from the study.

The following evaluations should be performed at the early termination visit:

- Review of AEs and changes in concomitant medications.
- Symptom-directed physical examination (urogenital/anorectal examinations will be performed at the discretion of the investigator).
- Vital signs measurement (blood pressure, pulse, respiration rate, and temperature).
- Obtain blood and urine samples as noted in Section 6.5.
- Counsel participant regarding the importance of continuing a complete ART regimen in accordance to standard of care, and refer patient to an appropriate HIV treatment facility.

6.4.2. 30-Day, 90-Day, and 180-Day Follow-Up Visits

The assessments below will be completed for participants who are required to complete 30-day, 90-day, and/or 180-day follow-up visits as noted in Section 6.4.1. Follow-up visits will be scheduled based on the date of the early termination visit. No follow-up visits are required for those participants who do not receive SC LEN at the Week 52 visit.

For scheduling the follow-up visits, a ± 6-day window may be used.

The following evaluations are to be completed at the follow-up visits:

- Review of AEs and changes in concomitant medications.
- Symptom-directed physical examination.
- Vital signs measurement (blood pressure, pulse, respiration rate, and temperature), including weight.
- Obtain blood and urine samples as noted in Section 6.5.

At the 30-day, 90-day, or 180-day follow-up visit, if there are any abnormal laboratory results indicating that there is a possible or probable causal relationship with the study drug, every attempt should be made to repeat those laboratory tests weekly (or as often as deemed prudent by the investigator) until the abnormality is resolved, returns to baseline, or is otherwise explained. If this is not possible or acceptable to the participant or investigator, the participant may be withdrawn from the study.

If there are any AEs, every attempt should be made to keep the participant in the study and should be followed up until resolution or until the AE is stable, if possible. Gilead may request that certain AEs be followed beyond the protocol-defined follow-up period.

6.5. Clinical Laboratory Assessments

Blood and urine samples will be collected throughout the study as outlined below and in Study Procedures Table (Appendix 2.).

6.5.1. Blood Samples

Blood sample collection for the following laboratory analyses will be performed at every visit, unless specified:

- Serum pregnancy test for all women (screening visit only).
- Serum follicle-stimulating hormone (FSH) test (FSH test is required for women who are < 54 years old and have stopped menstruating for ≥ 12 months but do not have documentation of ovarian hormonal failure) (screening visit only).
- Chemistry profile: alkaline phosphatase, aspartate aminotransferase, ALT, gamma-glutamyl transferase, total bilirubin, direct and indirect bilirubin, total protein, albumin, lactate dehydrogenase, creatine kinase (CK), bicarbonate, blood urea nitrogen, calcium, chloride, creatinine, glucose, lipase, magnesium, phosphorus, potassium, sodium, uric acid (except at the Cohort Selection, Days 2, 5, 16, and 19 visits).
- Estimated GFR (except at the Cohort Selection, Days 2, 5, 16, and 19 visits) according to:
  - Cockcroft-Gault formula for CL_{cr} for participants ≥ 18 years of age.

    Men: $$\frac{(140 - \text{age in years}) \times (\text{wt in kg})}{72 \times (\text{serum creatinine in mg/dL})} = CL_{cr}\ (\text{mL/min})$$

    Women: $$\frac{(140 - \text{age in years}) \times (\text{wt in kg}) \times 0.85}{72 \times (\text{serum creatinine in mg/dL})} = CL_{cr}\ (\text{mL/min})$$

  - Schwartz Formula for participants < 18 years of age.

    Adolescent boys ≥ 12 years of age: $0.70 \times L/S_{Cr}$ (L is height in cm).

    Adolescent girls ≥ 12 years of age: $0.55 \times L/S_{Cr}$ (L is height in cm).

- Hematology profile: complete blood count with differential and platelet count (except at the Cohort Selection visit, Days 2, 5, 16, and 19 visits).
- CD4+ cell count (except at the Cohort Selection visit, Days 2, 5, 16, and 19 visits).

- Plasma HIV-1 RNA (except at Day 5 and 19).
- HIV-1 genotype and phenotype for protease (PR), RT, integrase (IN), and capsid at screening visit and any subsequent visit with virologic failure (described in Section 6.7).
- HBV serologies (HBV surface antigen, HBV core antibody, HBV surface antibody) (screening only).
- HCV antibody serology (screening only).
- CCI [redacted]

[redacted]

PK Sampling

PK sampling will occur relative to dosing of LEN at the following time points for all participants:

- Day 1: Predose (within 30 minutes of dosing), 1, 2, 4, 6, and 8 hours postdose.
- Day 2 and 8:
  - Predose (within 30 minutes of dosing).
  - A single timed PK sample between 1 and 6 hours postdose.
- Days 5 (if visit occurs): A single anytime PK sample.
- Day 15 (for Cohort 1B participants only): Predose (within 30 minutes of dosing), 1, 2, 4, 6, and 8 hours postdose.
- Day 16 and 22 (for Cohort 1B participants only):
  - Predose (within 30 minutes of dosing).
  - A single timed PK sample between 1 and 6 hours postdose.
- Days 19 (for Cohort 1B participants only, if visit occurs): A single anytime PK sample.
- During the Maintenance Period, at all visits without SC LEN injections, including the Oral Bridging visits, if applicable: A single anytime PK sample will be collected.

- During the Maintenance Period, at all visits with SC LEN injections, including when SC LEN injection is resumed after the Oral Bridging visits, if applicable: A single predose (within 30 minutes of dosing) PK sample will be collected (while participants are continuing to receive study drug).

At Week 156 and thereafter, upon regulatory and/or IRB/IEC approval of the protocol and when participants have provided written informed consent of the most current IRB-approved consent form, participants will continue to receive study drug every 26 weeks, and continue to attend visits every 26 weeks (± 2 weeks). Participants will not be required to provide PK samples for these visits.

6.5.2. Blood and Urine Storage Samples

Any residual blood (PK and virology samples) and urine samples collected at all visits (except the screening and early termination visits) will be frozen and stored. These stored blood and urine samples may be used by the sponsor or its research partners for HIV-1 genotyping/phenotyping assays or their development, for retesting the amount of HIV-1 in the blood, for measurement of antiviral drug levels in the blood, or for future testing to learn more about how the study drug has worked against HIV-1 or clinical laboratory testing to provide additional safety data. No human genetic testing will be performed using these samples without consent of study participants.

At the conclusion of this study, these samples may be retained in storage by Gilead for a period up to 15 years.

6.5.3. Urine Samples

Urine samples will be collected for the following laboratory analyses at every study visit, unless otherwise specified:

- Urinalysis and urine chemistry: including color & clarity, specific gravity, pH, glucose, ketones, bilirubin, urobilinogen, blood, nitrite, leukocyte esterase and microscopic (if microscopic elements are seen), urine protein, albumin, creatinine, phosphate, calcium, magnesium and uric acid (except at Cohort Selection visit, Days 2, 5, 16, and 19 visits).

- Urine pregnancy test for women of childbearing potential (except at screening, Cohort Selection visit, Days 2, 5, 16, and 19 visits).
  - If the test is positive, confirmatory serum test should be performed and study drug dosing should be delayed until results obtained.

- Urine storage for possible additional clinical testing (except at screening, Cohort Selection, Day 5, Day 19, and at 30-, 90-, and 180-day follow-up visits, as applicable).

6.6. Criteria for Discontinuation of Study Treatment

Study drug may be discontinued in the following instances:

- Intercurrent illness that would, in the judgment of the investigator, affect assessments of clinical status to a significant degree. Following resolution of intercurrent illness, the participant may resume study drug at the discretion of the investigator.
- Unacceptable toxicity, or toxicity that, in the judgment of the investigator, compromises the ability to continue study-specific procedures or is considered to not be in the participant's best interest.
- Lack of Efficacy; participants may continue to receive study drug if the investigator believes that there is medical benefit in doing so.
- Participant request to discontinue for any reason.
- Participant noncompliance.
- Pregnancy during the study; refer to Appendix 4.
- Discontinuation of the study at the request of Gilead, a regulatory agency, or an IRB or IEC.

6.7. Management of Virologic Failure

Participants who experience suboptimal virologic response (SVR), virologic rebound (VR), or are viremic at their last visit, as defined below, will be considered to have virologic failure for the purposes of resistance analysis.

6.7.1. Suboptimal Virologic Response

Suboptimal virology response is defined as:

- HIV-1 RNA ≥ 50 copies/mL **and** < 1 $\log_{10}$ HIV-1 RNA reduction from the start of oral LEN (Day 1 for Cohort 1A and Cohort 2 participants, or Day 15 for Cohort 1B participants) at the Week 4 visit.

Following the first instance of SVR at Week 4, participants will be asked to return to the clinic for a scheduled or unscheduled blood draw (2 to 3 weeks after first SVR visit) for confirmation of SVR. A plasma sample from either the first instance or the SVR confirmation visit will be tested for HIV-1 capsid genotypic and phenotypic resistance. In addition, if SVR is confirmed, a plasma sample from the SVR confirmation visit will be tested for HIV-1 PR, RT, and IN genotypic and phenotypic resistance.

6.7.2. Virologic Rebound

Virologic rebound is defined as:

- At any visit, after achieving HIV-1 RNA < 50 copies/mL, a rebound in HIV-1 RNA to ≥ 50 copies/mL, which is subsequently confirmed at the following scheduled or unscheduled visit; OR

- At any visit, a > 1 $\log_{10}$ increase in HIV-1 RNA from the nadir which is subsequently confirmed at the following scheduled or unscheduled visit.

At any visit after achieving HIV-1 RNA < 50 copies/mL, if the HIV-1 RNA is ≥ 50 copies/mL and < 200 copies/mL, a reflex HIV-1 RNA repeat test will be conducted on stored plasma samples, if available. If the repeat result is < 50 copies/mL, no further action is required. If the repeat result is ≥ 50 copies/mL participants will be asked to return to the clinic for a scheduled or unscheduled blood draw (2 to 3 weeks after the date of the original test that resulted in HIV-1 RNA VR) for confirmation of VR.

A plasma sample from either the first instance or the VR confirmation visit will be tested for HIV-1 capsid genotypic and phenotypic resistance. In addition, if VR is confirmed, a plasma sample from the VR confirmation visit (or from the next available visit) will be tested for HIV-1 PR, RT, and IN genotypic and phenotypic resistance.

6.7.3. Viremia at Last Visit

Participants with HIV-1 RNA ≥ 50 copies/mL at their last study visit (eg, not confirmable) will be analyzed for resistance (HIV-1 capsid, PR, RT, and IN resistance testing).

6.8. End of Study

The end of the study will be the last participant's last observation (or visit).

6.9. Poststudy Care

After the participant has completed/terminated their participation in the study, long-term care of the participant will remain the responsibility of their primary treating physician.

7. ADVERSE EVENTS AND TOXICITY MANAGEMENT

7.1. Definitions of Adverse Events and Serious Adverse Events

7.1.1. Adverse Events

An AE is any untoward medical occurrence in a clinical study participant administered a study drug, which does not necessarily have a causal relationship with the treatment. An AE can therefore be any unfavorable and/or unintended sign, symptom, or disease temporally associated with the use of a study drug, whether or not considered related to the study drug. AEs may also include pre- or posttreatment complications that occur as a result of protocol-specified procedures or special situations (Section 7.7). Preexisting events that increase in severity or change in nature during or as a consequence of participation in the clinical study will also be considered AEs.

An AE does not include the following:

- Medical or surgical procedures such as surgery, endoscopy, tooth extraction, and transfusion. The condition that led to the procedure may be an AE and must be reported.
- Preexisting diseases, conditions, or laboratory abnormalities present or detected before the screening visit that do not worsen.
- Situations where an untoward medical occurrence has not occurred (eg, hospitalization for elective surgery, social and/or convenience admissions).
- Overdose without clinical sequelae (Section 7.7.1).
- Any medical condition or clinically significant laboratory abnormality with an onset date before the consent form is signed and not related to a protocol-associated procedure is not an AE. It is considered to be preexisting and should be documented as medical history.

7.1.2. Serious Adverse Events

An SAE is defined as an event that, at any dose, results in the following:

- Death.
- Life-threatening (Note: The term “life-threatening” in the definition of “serious” refers to an event in which the participant was at risk of death at the time of the event; it does not refer to an event that hypothetically might have caused death if it were more severe).
- In-patient hospitalization or prolongation of existing hospitalization.
- Persistent or significant disability/incapacity.

- A congenital anomaly/birth defect.

- A medically important event or reaction: such events may not be immediately life-threatening or result in death or hospitalization but may jeopardize the participant or may require intervention to prevent one of the other outcomes constituting SAEs. Medical and scientific judgment must be exercised to determine whether such an event is a reportable under expedited reporting rules. Examples of medically important events include intensive treatment in an emergency room or at home for allergic bronchospasm; blood dyscrasias or convulsions that do not result in hospitalization; and development of drug dependency or drug abuse.

7.2. Assessment of Adverse Events and Serious Adverse Events

The investigator or qualified subinvestigator is responsible for assessing AEs and SAEs for causality and severity, and for final review and confirmation of accuracy of event information and assessments.

7.2.1. Assessment of Causality for Study Drugs and Procedures

The investigator or qualified subinvestigator is responsible for assessing the relationship to study drug using clinical judgment and the following considerations:

- **No**: Evidence exists that the AE has an etiology other than the study drug. For SAEs, an alternative causality must be provided (eg, preexisting condition, underlying disease, intercurrent illness, or concomitant medication).

- **Yes**: There is reasonable possibility that the event may have been caused by the study drug.

It should be emphasized that ineffective treatment should not be considered as causally related in the context of AE reporting.

The relationship to study procedures (eg, invasive procedures such as venipuncture or biopsy) should be assessed using the following considerations:

- **No:** Evidence exists that the AE has an etiology other than the study procedure.

- **Yes:** The AE occurred as a result of protocol procedures, (eg, venipuncture).

7.2.2. Assessment of Severity

The severity of AEs will be graded using the Division of AIDS (DAIDS) Table for Grading the Severity of Adult and Pediatric Adverse Events, corrected Version 2.1, dated July 2017. For each episode, the highest grade attained should be reported as defined in the grading scale.

The DAIDS scale is available at the following location:
https://rsc.niaid.nih.gov/sites/default/files/daidsgradingcorrectedv21.pdf

7.3. Investigator Requirements and Instructions for Reporting Adverse Events and Serious Adverse Events

Requirements for Collection Prior to Study Drug Initiation:

After informed consent, but prior to initiation of study drug, the following types of events must be reported on the applicable eCRFs: all SAEs and AEs related to protocol-mandated procedures.

7.3.1. Adverse Events

Following initiation of study drug, all AEs, regardless of cause or relationship, throughout the duration of the study, including the protocol-defined follow-up visit, must be reported on the eCRFs as instructed.

All AEs should be followed up until resolution or until the AE is stable, if possible. Gilead may request that certain AEs be followed beyond the protocol-defined follow-up period.

7.3.2. Serious Adverse Events

All SAEs, regardless of cause or relationship, that occurs after the participant first consents to participate in the study (ie, signing the informed consent) and throughout the duration of the study, including the protocol-defined follow-up visit, must be reported on the applicable eCRFs and submitted to Gilead PS as instructed below in this section. This also includes any SAEs resulting from protocol-associated procedures performed after informed consent is signed.
The investigator must report the primary cause of death for any participant who dies during the follow-up period to the sponsor.

Investigators are not obligated to actively seek SAEs after the protocol-defined follow-up period. However, if the investigator learns of any SAEs that occur after the protocol-defined follow-up period has concluded and the event is deemed relevant to the use of study drug, the investigator should promptly document and report the event to Gilead PS.

- All AEs and SAEs will be recorded in the eCRF database within the timelines outlined in the eCRF completion guidelines.

7.3.2.1. Electronic Serious Adverse Event Reporting Process

- Site personnel record all SAE data on the applicable eCRFs and from there transmit the SAE information to Gilead PS within 24 hours of the investigator’s knowledge of the initial event and of any updates from the time of the informed consent form signature throughout the duration of the study, including the protocol-required posttreatment follow-up period. Detailed instructions can be found in the eCRF completion guidelines.

- If it is not possible to record and submit the SAE information electronically, because the eCRF database cannot be accessed or is not available (including at study start), record the SAE on the paper serious AE reporting form as an initial or follow-up, and submit within 24 hours to:

  Gilead Patient Safety: Gilead Patient Safety
  Email: Safety_FC@gilead.com or
  Fax: +1-650-522-5477

  Initial SAE reports need to be submitted using the Initial SAE Report Form. Any follow-up information on a previously reported SAE (including updates to the reported event term) will also be reported to PS and in applicable eCRFs within 24 hours of the investigator's knowledge of the new/updated information. Follow-up will be submitted to PS using the SAE Follow-up Report Form in accordance with the form's Completion Guidelines.

- As soon as it is possible to do so, any SAE reported via paper must be transcribed on the applicable eCRFs according to instructions and within the timelines outlined in the eCRF completion guidelines.

- If an SAE has been reported via a paper form because the eCRF database has been locked, no further action is necessary.

- For fatal or life-threatening events, copies of hospital case reports, autopsy reports, and other documents are also to be submitted by email or fax when requested and applicable. Transmission of such documents should occur without personal participant identification, maintaining the traceability of a document to the participant identifiers.

- Additional information may be requested to ensure the timely completion of accurate safety reports.

- Any medications necessary for treatment of the SAE must be recorded onto the concomitant medication section of the participant’s eCRF and the event description of the SAE form.

7.4. Gilead Reporting Requirements

Depending on relevant local legislation or regulations, including the applicable US FDA CFR, the EU Clinical Trials Directive (2001/20/EC) and relevant updates, and other country-specific legislation or regulations, Gilead may be required to expedite to worldwide regulatory agencies reports of SAEs, which may be in the form of line-listings, serious adverse drug reactions, or SUSARs. In accordance with the EU Clinical Trials Directive (2001/20/EC), Gilead or a specified designee will notify worldwide regulatory agencies and the relevant IEC in concerned Member States of applicable SUSARs as outlined in current regulations.

Assessment of expectedness for SAEs will be determined by Gilead using reference safety information specified in the IB or relevant local label as applicable.

All investigators will receive a safety letter notifying them of relevant SUSAR reports associated with any study drug. The investigator should notify the IRB or IEC of SUSAR reports as soon as is practical, where this is required by local regulatory agencies, and in accordance with the local institutional policy.

7.5. Clinical Laboratory Abnormalities and Other Abnormal Assessments as Adverse Events or Serious Adverse Events

Laboratory abnormalities without clinical significance are not recorded as AEs or SAEs. However, laboratory abnormalities (eg, clinical chemistry, hematology, and urinalysis) that require medical or surgical intervention or lead to study drug interruption, modification, or discontinuation must be recorded as an AE, as well as an SAE, if applicable. In addition, laboratory or other abnormal assessments (eg, ECG, x-rays, vital signs) that are associated with signs and/or symptoms must be recorded as an AE or SAE if they meet the definition of an AE or SAE as described in Sections 7.1.1 and 7.1.2. If the laboratory abnormality is part of a syndrome, record the syndrome or diagnosis (eg, anemia), not the laboratory result (eg, decreased hemoglobin).

Severity should be recorded and graded according to the DAIDS Table for Grading the Severity of Adult and Pediatric Adverse Events, corrected Version 2.1, dated July 2017. For AEs associated with laboratory abnormalities, the event should be graded on the basis of the clinical severity in the context of the underlying conditions; this may or may not be in agreement with the grading of the laboratory abnormality

7.6. Toxicity Management

All clinical and clinically significant laboratory toxicities will be managed according to uniform guidelines detailed in Appendix 3 and as outlined below.

Grade 3 and 4 clinically significant laboratory abnormalities should be confirmed by repeat testing within 3 calendar days of receipt of results and before study drug discontinuation, unless such a delay is not consistent with good medical practice. If repeat testing is not possible within 3 calendar days of receipt of results, it may be completed within 14 calendar days of receipt of results per the investigator's discretion.

The Gilead medical monitor should be consulted prior to study drug discontinuation when medically feasible. Before discontinuation of study drug for AEs or laboratory abnormalities, an assessment of the participants' medical situation including the potential for an alternative ARV regimen and the risk of resistance with subtherapeutic LEN exposure should be made. Although routinely, participants with Grade 3 or higher AEs or laboratory abnormalities that are considered related to the study drug should be discontinued, if alternative HIV therapies are not available, the participant may continue dosing with approval of the medical monitor following discussion with the investigator.

7.6.1. Grades 1 and 2 Laboratory Abnormality or Clinical Event

Continue study drug at the discretion of the investigator.

7.6.2. Grade 3 Laboratory Abnormality or Clinical Event

For a Grade 3 clinically significant laboratory abnormality or clinical event, study drug may be continued if the event is considered to be unrelated to study drug.

For a Grade 3 clinically significant laboratory abnormality or clinical event confirmed by repeat testing, that is considered to be related to study drug, consider withholding study drug until the toxicity returns to Grade 2 or lower.

If a clinically significant laboratory abnormality or clinical event recurs to Grade 3 or higher following re-challenge with study drug and is considered to be related to study drug, consider permanent discontinuation of study drug. The participant should be managed according to local practice.

Recurrence of laboratory abnormalities considered unrelated to study drug may not require permanent discontinuation but requires discussion with the Gilead medical monitor.

7.6.3. Grade 4 Laboratory Abnormality or Clinical Event

For a Grade 4 clinically significant laboratory abnormality or clinical event confirmed by repeat testing, that is considered to be related to study drug, consider permanent discontinuation of study drug. The participant should be managed according to local practice. The participant should be followed as clinically indicated until the laboratory abnormality returns to baseline or is otherwise explained, whichever occurs first. A clinically significant Grade 4 laboratory abnormality that is not confirmed by repeat testing should be managed according to the algorithm for the new toxicity grade.

Study drug may be continued without dose interruption for a clinically nonsignificant Grade 4 laboratory abnormality (eg, Grade 4 CK elevation after strenuous exercise or triglyceride elevation that is nonfasting or that can be medically managed) or a clinical event considered unrelated to study drug.

Treatment-emergent toxicities will be noted by the investigator and brought to the attention of the Gilead medical monitor, and the appropriate course of action will be discussed and decided. Whether or not considered treatment-related, all participants experiencing AEs must be monitored periodically until symptoms subside, any abnormal laboratory values have resolved or returned to baseline levels or they are considered irreversible, or until there is a satisfactory explanation for the changes observed.

Any questions regarding toxicity management should be directed to the Gilead medical monitor.

7.7. Special Situations Reports

7.7.1. Definitions of Special Situations

Special situation reports include all reports of medication error, abuse, misuse, overdose, occupational exposure with AE, AE in an infant following exposure via breastfeeding, product complaints with AE, and pregnancy regardless of an associated AE.

Medication error is any unintentional error in the prescribing, dispensing, preparation for administration or administration of a study drug while the medication is in the control of a health care professional, patient, or consumer. Medication errors may be classified as a medication error without an AE, which includes situations of missed dose; medication error with an AE; intercepted medication error; or potential medication error.

Abuse is defined as persistent or sporadic intentional excessive use of a study drug by a participant.

Misuse is defined as any intentional and inappropriate use of a study drug that is not in accordance with the protocol instructions or the local prescribing information.

An overdose is defined as an accidental or intentional administration of a quantity of a study drug given per administration or cumulatively which is above the maximum recommended dose as per protocol or in the product labeling (as it applies to the daily dose of the participant in question). In cases of a discrepancy in drug accountability, overdose will be established only when it is clear that the participant has taken the excess dose(s). Overdose cannot be established when the participant cannot account for the discrepancy except in cases in which the investigator has reason to suspect that the participant has taken the additional dose(s).

Occupational exposure is defined as exposure to a study drug as a result of one’s professional or nonprofessional occupation.

Product complaint is defined as complaints arising from potential deviations in the manufacture, packaging, or distribution of the study drug.

7.7.2. Instructions for Reporting Special Situations

7.7.2.1. Instructions for Reporting Pregnancies

The investigator should report pregnancies in female study participants and female partners of male study participants that are identified after initiation of study drug and throughout the study, including the protocol-defined follow-up period, to Gilead PS using the pregnancy report form within 24 hours of becoming aware of the pregnancy. Refer to Appendix 4 for further information regarding protocol-defined follow-up period.

If the investigator learns of any pregnancy or pregnancy outcomes that occur after the protocol-defined follow-up period has concluded but within 700 days following the last dose of SC LEN, or within 60 days of last oral dose of LEN, the investigator should promptly document and report the event to Gilead PS.

Refer to Section 7.3 and the eCRF completion guidelines for full instructions on the mechanism of pregnancy reporting.

The pregnancy itself is not considered an AE nor is an induced elective abortion to terminate a pregnancy without medical reasons.

Any premature termination of pregnancy (eg, a spontaneous abortion, an induced therapeutic abortion due to complications or other medical reasons) must be reported within 24 hours as an SAE. The underlying medical reason for this procedure should be recorded as the AE term.

A spontaneous abortion is always considered to be an SAE and will be reported as described in Section 7.3. Furthermore, any SAE occurring as an adverse pregnancy outcome after study must be reported to Gilead PS.

The participant should receive appropriate monitoring and care until the conclusion of the pregnancy. The outcome should be reported to Gilead PS using the pregnancy outcome report form. If the pregnancy/outcome occurs after the study has been completed, including the protocol-defined follow-up period, but within 700 days of the last dose of SC LEN, or within 60 days of last oral dose of LEN, the pregnancy/outcome should be reported directly to Gilead PS. Gilead PS contact information is as follows: email: Safety_FC@gilead.com and fax: +1-650-522-5477.

Pregnancies of female partners of male study participants exposed to Gilead or other study drugs during the study, including the protocol-defined follow-up period, must also be reported and relevant information should be submitted to Gilead PS using the pregnancy and pregnancy outcome forms within 24 hours. If the end of pregnancy occurs after the study has been completed, the outcome should be reported directly to Gilead PS, fax number +1-650-522-5477 or email Safety_FC@gilead.com.

Refer to Appendix 4. for Pregnancy Precautions, Definition for Women of Childbearing Potential, and Contraceptive Requirements.

7.7.2.2. Reporting Other Special Situations

All other special situation reports must be reported on the special situations report form and forwarded to Gilead PS within 24 hours of the investigator becoming aware of the situation. These reports must consist of situations that involve study drug and/or Gilead concomitant medications, but do not apply to non-Gilead concomitant medications.

Special situations involving non-Gilead concomitant medications do not need to be reported on the special situations report form; however, special situations that result in AEs due to a non-Gilead concomitant medication, must be reported as an AE.

Any inappropriate use of concomitant medications prohibited by this protocol should not be reported as “misuse,” but may be more appropriately documented as a protocol deviation.

Refer to Section 7.3 and the eCRF completion guidelines for instructions on special situation reporting.

All clinical sequelae in relation to these special situation reports will be reported as AEs or SAEs at the same time using the AE eCRF and/or the SAE report form. Details of the symptoms and signs, clinical management, and outcome will be reported, when available.

8. STATISTICAL CONSIDERATIONS

8.1. Analysis Objectives and Endpoints

8.1.1. Analysis Objectives

The primary objective of this study is:

- To evaluate the antiviral activity of LEN administered as an add-on to a failing regimen (functional monotherapy) for PWH with MDR as determined by the proportion of participants achieving at least 0.5 $\log_{10}$ reduction from baseline in HIV-1 RNA at the end of the Functional Monotherapy Period.

The secondary objectives of this study are:

- To evaluate the safety and efficacy of LEN in combination with an OBR at Weeks 26 and 52.
- To evaluate the safety and efficacy of LEN in combination with an OBR at Weeks 104 and 156 (from the first SC dose of LEN).

CCI [redacted]

- [redacted]
- [redacted]
- [redacted]
- [redacted].

8.1.2. Primary Endpoint

The proportion of participants in Cohort 1 achieving $\geq$ 0.5 $\log_{10}$ copies/mL reduction from baseline in HIV-1 RNA at the end of Functional Monotherapy Period.

8.1.3. Secondary Endpoints

The secondary endpoints of this study are:

- The proportion of participants in Cohort 1 with plasma HIV-1 RNA < 50 copies/mL and < 200 copies/mL at Weeks 26 and 52 visits based on the US FDA–defined snapshot algorithm.
- The proportion of participants in combined Cohorts 1 and 2 with plasma HIV-1 RNA < 50 copies/mL and < 200 copies/mL at Weeks 104 and 156 from the first SC dose of LEN based on the US FDA–defined snapshot algorithm.

8.2. Planned Analyses

8.2.1. Interim Analysis

Before the final analysis, interim analyses may be conducted, and the analyses may be submitted to regulatory agencies to seek guidance for the overall clinical development program.

8.2.1.1. Dose Escalation Analysis

Not applicable.

8.2.1.2. Planned Internal Analysis

Interim analyses after the primary analysis (see Section 8.2.2) will be performed after all participants in Cohort 1 have completed their Week 52 visit and/or after all participants in Cohort 2 have completed their Week 26 visit or have prematurely discontinued the study drug, outstanding data queries have been resolved or adjudicated as unresolvable, and the data have been cleaned and finalized.

8.2.1.3. Data Monitoring Committee (DMC) Analysis

There will be 1 planned DMC analysis of efficacy and safety. The DMC will convene after all participants in Cohort 1 have completed 14 days of assessment or discontinued the study drug in the Functional Monotherapy Period. Given that all participants have completed the Functional Monotherapy Period, treatment assignment will be unblinded for the DMC analysis.

No formal stopping rules will be used by the DMC for safety outcomes. Rather, a clinical assessment will be made to determine if the nature, frequency, and severity of AEs associated with a study regimen warrant the early termination of the study in the best interest of the participants.

Gilead does not have a prior intent to ask the DMC to consider early termination of the study even if there is an early evidence of favorable efficacy. However, Gilead will stop further enrollment if 50% or more of the participants in the LEN group fail in Cohort 1 to achieve at least 0.5 $\log_{10}$ reduction from baseline in HIV-1 RNA at the end of the Functional Monotherapy Period. The decision whether to continue with the study and the development of LEN will be based on the magnitude of the HIV-1 RNA decline at the end of the Functional Monotherapy Period.

8.2.2. Primary Analysis

The primary analysis of the primary endpoint will be conducted after all participants in Cohort 1 have completed the Week 26 visit or have prematurely discontinued the study drug, outstanding data queries have been resolved or adjudicated as unresolvable, and the data have been cleaned and finalized for the analysis. This analysis of the primary endpoint will serve as the final analysis for this endpoint. The data from this analysis will be used to support the LEN regulatory filing for the indication in the HTE PWH.

8.2.3. Year 2 Analysis

The Year 2 Analysis will be performed after all participants in both cohorts have had HIV-1 RNA data in the Week 104 analysis window (or reached the upper limit of the analysis window with missing HIV-1 RNA data), or have prematurely discontinued the study drug, outstanding data queries have been resolved or adjudicated as unresolvable, and the data have been cleaned and finalized.

8.2.4. Year 3 Analysis

The Year 3 Analysis will be performed after all participants in both cohorts have had HIV-1 RNA data in the Week 156 analysis window (or reached the upper limit of the analysis window with missing HIV-1 RNA data), or have prematurely discontinued the study drug, outstanding data queries have been resolved or adjudicated as unresolvable, and the data have been cleaned and finalized.

8.2.5. Final Analysis

The final analysis will be performed after all participants have completed the study, outstanding data queries have been resolved or adjudicated as unresolvable, and the data have been cleaned and finalized.

8.3. Analysis Conventions

8.3.1. Analysis Sets

8.3.1.1. Efficacy

The primary analysis set for efficacy analysis is defined as Full Analysis Set (FAS). Two FASs are defined for this study: one for the primary efficacy endpoint (referred to as "FAS for the Functional Monotherapy Period analysis") and the other for secondary efficacy endpoints (referred to as "FAS for the All LEN analysis"). The FAS for the Functional Monotherapy Period includes all participants who are randomized and receive at least 1 dose of blinded study drug. In this analysis, participants will be grouped according to the treatment to which they are randomized. The FAS for the All LEN analysis includes all participants who receive at least 1 dose of LEN.

8.3.1.2. Safety

The primary analysis set for safety analyses is defined as safety analysis set, which includes all participants who are randomized/enrolled and receive any dose of study drug. Participants who receive treatment other than that intended will be analyzed according to treatment received. All data collected during treatment will be included in the safety summaries.

8.3.1.3. Pharmacokinetics

The primary analysis set for PK analyses is defined for LEN, which includes all participants who are randomized/enrolled, receive any dose of study drug, and have at least 1 nonmissing postbaseline concentration value for LEN.

8.3.2. Data Handling Conventions

Logarithm (base 10) transformation will be applied to HIV-1 RNA levels for efficacy analysis. Natural logarithm transformation for all PK parameters of LEN will be applied for PK analysis.

For summary statistics, PK concentration values below the limit of quantitation will be treated as zero at predose and one-half of the LLOQ for postdose time points.

Laboratory data that are continuous in nature but are less than the LLOQ or above the upper limit of quantitation will be imputed to the value of the lower or upper limit minus or plus 1 significant digit, respectively (eg, if the result of a continuous laboratory test is < 20, a value of 19 will be assigned; if the result of a continuous laboratory test is < 20.0, a value of 19.9 will be assigned).

8.4. Demographic and Baseline Characteristics Analysis

Demographic and baseline measurements will be summarized using standard descriptive methods by cohort and treatment.

Demographic summaries will include sex, race, ethnicity, age, sexual orientation, and gender identity.

Baseline data will include a summary of body weight, height, and body mass index.

For Cohort 1, the Cochran-Mantel-Haenszel test will be used to compare the 2 treatment groups for categorical data, and the 2-sided Wilcoxon rank sum test will be used to compare the treatment groups for continuous data. No statistical comparisons will be made for Cohort 2.

8.5. Efficacy Analysis

8.5.1. Primary Analysis

The primary efficacy endpoint is the proportion of participants in Cohort 1 achieving $\geq$ 0.5 $\log_{10}$ reduction from baseline in HIV-1 RNA at the end of the Functional Monotherapy Period.
The primary analysis of the efficacy endpoint will be based on the FAS for the Functional Monotherapy Period.

The null hypothesis is that there is no difference in the proportion of participants achieving ≥ 0.5 log_{10} reduction from baseline at the end of the Functional Monotherapy Period (between the LEN group and the Placebo group in Cohort 1); the alternative hypothesis is that there is a difference (LEN − Placebo) in the proportion of participants achieving ≥ 0.5 log_{10} reduction from baseline at the end of the Functional Monotherapy Period between the 2 treatment groups in Cohort 1. For participants with missing HIV-1 RNA values at the end of the Functional Monotherapy Period the value will be imputed using the last observation carried forward method. The difference in proportions between 2 treatment groups will be compared using an unconditional exact method using 2 invert 1-sided tests {Chan 1999} with an alpha level at 0.05 to evaluate superiority. The *P* value and 95% CI for the point estimate of treatment difference in proportions will be estimated and constructed using the above-mentioned method.

8.5.2. Secondary Analyses

The proportion of participants in Cohort 1 with HIV-1 RNA < 50 copies/mL at Week 26 will be summarized using the US FDA–defined snapshot algorithm.

The analysis window at Week 26 is defined as from Study Day 184 to Study Day 232, inclusive, where Study Day is calculated from the first dose of LEN. Virologic outcome will be defined as the following categories:

- **HIV-1 RNA < 50 copies/mL:** this includes participants who have the last available on-treatment HIV-1 RNA < 50 copies/mL in the Week 26 analysis window
- **HIV-1 RNA ≥ 50 copies/mL:** this includes participants
  - Who have the last available on-treatment HIV-1 RNA ≥ 50 copies/mL in the Week 26 analysis window, or
  - Who do not have on-treatment HIV-1 RNA data in the Week 26 analysis window and
    - Who discontinue study drug prior to or in the Week 26 analysis window due to lack of efficacy, or
    - Who discontinue study drug prior to or in the Week 26 analysis window due to reasons other than AE, death, or lack of efficacy and have the last available on-treatment HIV-1 RNA ≥ 50 copies/mL.
- **No Virologic Data in the Week 26 analysis window:** this includes participants who do not have on-treatment HIV-1 RNA data in the Week 26 analysis window because of the following:
  - Discontinuation of study drug prior to or in the Week 26 analysis window due to AE or death (regardless of whether the last available on-treatment HIV-1 RNA < 50 copies/mL or not) or,

- Discontinuation of study drug prior to or in the Week 26 analysis window due to reasons other than AE, death, or lack of efficacy and the last available on-treatment HIV-1 RNA < 50 copies/mL or,
- Missing data during the window but on study drug.

The proportion of participants in Cohort 1 with HIV-1 RNA < 200 copies/mL at Week 26 will also be summarized using the US FDA–defined snapshot algorithm.

In addition, the proportion of participants in Cohort 1 with HIV-1 RNA < 50 copies/mL and < 200 copies/mL at Week 52, and the proportion of participants in combined Cohorts 1 and 2 with HIV-1 RNA < 50 copies/mL and < 200 copies/mL at Weeks 104 and 156 (from the first SC dose of LEN) will also be summarized using the US FDA–defined snapshot algorithm. The analysis window at Week 52 is defined as from Study Day 324 to Study Day 414, inclusive. The analysis window at Week 104 is defined as from Study Day 688 to Study Day 778, inclusive. The analysis window at Week 156 is defined as from Study Day 1052 to Study Day 1142, inclusive. The secondary endpoints will be based on the FAS for the All LEN analysis. Analysis will be based on participants who receive at least 1 dose of SC LEN.

8.6. Safety Analysis

All safety data collected on or after the date that study drug was first dispensed will be summarized by cohort and treatment (according to the study drug received). For participants who discontinue from the oral LEN, only data collected up to 60 days (approximately 5 times the LEN oral dose half-life) after the last dose of the oral tablet will be included. All data collected will be included in data listings.

8.6.1. Extent of Exposure

A participant’s extent of exposure to study drug data will be generated from the study drug administration data. Exposure data will be listed.

8.6.2. Adverse Events

Clinical and laboratory AEs will be coded using the MedDRA. System Organ Class (SOC), High-Level Group Term, High-Level Term, Preferred Term (PT), and Lower-Level Term will be attached to the clinical database.

Events will be summarized on the basis of the date of onset for the event. A treatment-emergent AE will be defined as any AE that begins on or after the date of first dose of study drug. For participants who discontinue from the oral LEN, only AEs collected up to 60 days (approximately 5 times the LEN oral dose half-life) after the last dose date will be considered treatment emergent.

Summaries (number and percentage of participants) of treatment-emergent AEs (TEAEs) (by SOC, and PT) will be provided by treatment group.

8.6.3. Laboratory Evaluations

Selected laboratory data (using units) will be summarized using only observed data. Data and change from baseline at all scheduled time points will be summarized.

Graded laboratory abnormalities will be defined using the grading scheme in the DAIDS Table for Grading the Severity of Adult and Pediatric Adverse Events, corrected Version 2.1, dated July 2017.

Incidence of treatment-emergent laboratory abnormalities, defined as values that increase at least 1 toxicity grade from baseline at any time postbaseline (and up to 60 days after the last dose date if participants discontinue from oral LEN), will be summarized by cohort and treatment group. If baseline data are missing, any graded abnormality (eg, at least a Grade 1) will be considered treatment emergent. The postbaseline maximum toxicity grade will be summarized by laboratory parameter.

8.6.4. Other Safety Evaluations

Vital sign and safety ECG data will be summarized and/or listed as appropriate.

8.7. Adjustments for Multiplicity

The primary efficacy endpoint will be evaluated at the time when all participates in Cohort 1 have completed the Functional Monotherapy Period or discontinued the study drug. There will be no interim analyses before the analysis of the primary efficacy endpoint; therefore, no alpha level adjustment will be applied to the primary efficacy endpoint.

8.8. Pharmacokinetic Analysis

Plasma concentrations of LEN will be summarized by nominal sampling time using descriptive statistics, as appropriate (eg, sample size, arithmetic mean, 90% CIs, geometric mean, %CV, standard deviation, median, minimum, and maximum). The concentrations of the study drug in plasma over time will be plotted in semilogarithmic and linear formats as mean ± standard deviation and median (first quartile [Q1], third quartile [Q3]), respectively.

8.9. Sample Size

A total of 36 participants in Cohort 1 will provide at least 90% power to detect a 60% difference in the proportion of participants achieving a $\geq 0.5 \log_{10}$ reduction from baseline at Day 15 of the Functional Monotherapy Period between the treatment groups (LEN in Cohort 1A and placebo in Cohort 1B).

In this sample size and power computation, it is assumed that 70% and 10% of participants achieve a $\geq 0.5 \log_{10}$ reduction from baseline in HIV-1 RNA in the LEN treatment group (Cohort 1A) and the placebo group (Cohort 1B), respectively (based on data from Trogarzo Phase 3 TMB-301 study {Emu 2018}), and the Fisher exact test is conducted at 2-sided significant level of 0.05.

A total sample size of 36 participants from Cohort 1A and 1B will provide reasonable assessment of safety through at least 24 weeks of LEN treatment in HTE participants.

8.10. Data Monitoring Committee

An external multidisciplinary DMC will review the progress of the study and perform interim reviews of efficacy and safety data, and provide recommendation to Gilead whether the nature, frequency, and severity of adverse effects associated with study treatment warrant the early termination of the study in the best interests of the participants, whether the study should continue as planned, or the study should continue with modifications.

The DMC’s specific activities will be defined by a mutually agreed charter, which will define the DMC’s membership, conduct, and meeting schedule.

While the DMC will be asked to advise Gilead regarding future conduct of the study, including possible early study termination, Gilead retains final decision-making authority on all aspects of the study.

9. RESPONSIBILITIES

9.1. Investigator Responsibilities

9.1.1. Good Clinical Practice

The investigator will ensure that this study is conducted in accordance with International Council for Harmonisation (ICH) E6(R2) addendum to its guideline for GCP and applicable laws and regulations.

9.1.2. Good Postmarketing Study Practices (Japan Only)

The investigator will ensure that this study is conducted in accordance with GPSP after approval of LEN in Japan and adherence to the basic principles of GCP.

9.1.3. Financial Disclosure

The investigator and subinvestigators will provide prompt and accurate documentation of their financial interest or arrangements with Gilead, or proprietary interests in the investigational drug during the course of a clinical study. This documentation must be provided prior to the investigator's (and any subinvestigator's) participation in the study. The investigator and subinvestigator agree to notify Gilead of any change in reportable interests during the study and for 1 year following completion of the study. Study completion is defined as the date when the last participant completes the protocol-defined activities.

9.1.4. Institutional Review Board/Independent Ethics Committee Review and Approval

The investigator (or Gilead as appropriate according to local regulations) will submit this protocol, informed consent form, and any accompanying material to be provided to the participant (such as advertisements, participant information sheets, or descriptions of the study used to obtain informed consent) to an IRB/IEC. The investigator will not begin any study participant activities until approval from the IRB/IEC has been documented and provided as a letter to the investigator.

Before implementation, the investigator will submit to and receive documented approval from the IRB/IEC any modifications made to the protocol or any accompanying material to be provided to the participant after initial IRB/IEC approval, with the exception of those necessary to reduce immediate risk to study participants.

9.1.5. Informed Consent

The investigator is responsible for obtaining written informed consent from each participant after adequate explanation of the aims, methods, objectives, and potential hazards of the study before undertaking any study-related procedures. The investigator must use the most current IRB- or

IEC-approved consent form for documenting written informed consent. Each informed consent (or assent as applicable) will be appropriately signed and dated by the participant or the participant's legally authorized representative and the person conducting the consent discussion, and also by an impartial witness if required by IRB or IEC or local requirements.

A consent form will inform participants about pharmacogenomic testing and/or planned sample retention. CCI [redacted] The results of the tests done on the samples will not be given to the participant or the investigator.

9.1.6. Confidentiality

The investigator must assure that participants' anonymity will be strictly maintained and that their identities are protected from unauthorized parties. Only an identification code and any other unique identifier(s) as allowed by local law (such as year of birth) will be recorded on any form or biological sample submitted to Gilead, or laboratory. Laboratory specimens must be labeled in such a way as to protect participant identity while allowing the results to be recorded to the proper participant. Refer to specific laboratory instructions. NOTE: The investigator must keep a screening log with details for all participants screened and enrolled in the study, in accordance with the site procedures and regulations. Participant data will be processed in accordance with all applicable regulations.

The investigator agrees that all information received from Gilead, including but not limited to the IB, this protocol, eCRF, the study drug, and any other study information, remain the sole and exclusive property of Gilead during the conduct of the study and thereafter. This information is not to be disclosed to any third party (except employees or agents directly involved in the conduct of the study or as required by law) without prior written consent from Gilead.
The investigator further agrees to take all reasonable precautions to prevent the disclosure by any employee or agent of the study site to any third party or otherwise into the public domain.

9.1.7. Study Files and Retention of Records

The investigator must maintain adequate and accurate records to enable the conduct of the study to be fully documented and the study data to be subsequently verified. These documents should be classified into at least the following 2 categories: (1) investigator's study file, and (2) participant clinical source documents.

The investigator's study file will contain the protocol/amendments, eCRFs, and governmental approval with correspondence, informed consent, drug records, staff curriculum vitae and authorization forms, and other appropriate documents and correspondence.

The required source data should include sequential notes containing at least the following information for each participant:

- Participant identification;
- Documentation that participant meets eligibility criteria, ie, medical history, physical examination, and confirmation of diagnosis (to support inclusion and exclusion criteria);
- Documentation of the reason(s) a consented participant is not enrolled;
- Participation in study (including study number);
- Study discussed and date of informed consent;
- Dates of all visits;
- Documentation that protocol-specific procedures were performed;
- Results of efficacy parameters, as required by the protocol;
- Start and end date (including dose regimen) of study drug, including dates of dispensing and return;
- Record of all AEs and other safety parameters (start and end date, and including causality and severity), and documentation that adequate medical care has been provided for any AE;
- Concomitant medication (including start and end date, dose if relevant; dose changes);
- Date of study completion and reason for early discontinuation if it occurs.

All clinical study documents must be retained by the investigator until at least 2 years or according to local laws, whichever is longer, after the last approval of a marketing application in an ICH region (eg, US, Europe, or Japan) and until there are no pending or planned marketing applications in an ICH region; or, if no application is filed or if the application is not approved for such indication, until 2 years after the investigation is discontinued and regulatory authorities have been notified. Investigators may be required to retain documents longer if specified by regulatory requirements, by local regulations, or by an agreement with Gilead. The investigator must notify Gilead before destroying any clinical study records.

Should the investigator wish to assign the study records to another party or move them to another location, Gilead must be notified in advance.

If the investigator cannot provide for this archiving requirement at the study site for any or all of the documents, special arrangements must be made between the investigator and Gilead to store these records securely away from the site so that they can be returned sealed to the investigator in case of an inspection. When source documents are required for the continued care of the participant, appropriate copies should be made for storage away from the site.

9.1.8. Case Report Forms

For each participant consented, an eCRF casebook will be completed by an authorized study staff member whose training for this function is completed in the electronic data capture (EDC) system. The eCRF casebook will only capture the data required per the protocol schedule of events and procedures. The Inclusion/Exclusion Criteria and Enrollment eCRFs should be completed only after all data related to eligibility have been received. Data entry should be performed in accordance with the eCRF Completion Guidelines provided by the sponsor.
After data entry, a study monitor will perform source data verification within the EDC system. System-generated or manual queries will be issued in the EDC system as data discrepancies are identified by the monitor or Gilead staff, who routinely review the data for completeness, correctness, and consistency. The site investigator or site coordinator or other designee is responsible for responding to the queries in a timely manner, within the system, either by confirming the data as correct or updating the original entry, and providing the reason for the update (eg, data entry error). Original entries as well as any changes to data fields will be stored in the audit trail of the system. At a minimum, prior to any interim time points or database lock (as instructed by Gilead), the investigator will use his/her log in credentials to confirm that the forms have been reviewed, and that the entries accurately reflect the information in the source documents. At the conclusion of the study, Gilead will provide the site investigator with a read-only archive copy of the data entered by that site. This archive must be stored in accordance with the records retention requirements outlined in Section 9.1.7.

9.1.9. Investigator Inspections

The investigator will make available all source documents and other records for this study to Gilead's appointed study monitors, to IRBs/IECs, or to regulatory authority or health authority inspectors.

9.1.10. Protocol Compliance

The investigator is responsible for ensuring the study is conducted in accordance with the procedures and evaluations described in this protocol.

9.2. Sponsor Responsibilities

9.2.1. Protocol Modifications

Protocol modifications, except those intended to reduce immediate risk to study participants, may be made only by Gilead. The investigator must submit all protocol modifications to the in accordance with local requirements and receive documented approval before modifications can be implemented.

9.2.2. Study Reports and Publications

A CSR will be prepared and provided to the regulatory agency(ies) when applicable and in accordance with local regulatory requirements. Gilead will ensure that the report meets the standards set out in the ICH Guideline for Structure and Content of Clinical Study Reports (ICH E3). Note that an abbreviated report may be prepared in certain cases. For studies with sites in countries following the Directive 2001/20/EC, a CSR will be submitted within 1 year (6 months for pediatric studies, in accordance with Regulation [EC] No. 1901/2006) after the global end of study (as defined in Section 6.8).

Investigators in this study may communicate, orally present, or publish in scientific journals or other scholarly media in accordance with the Gilead clinical trial agreement. Study results will be made publicly available (including posted to the Clinical Trials Information System and ClinicalTrials.gov) in accordance with local regulatory requirements.

9.3. Joint Investigator/Sponsor Responsibilities

9.3.1. Payment Reporting

Investigators and their study staff may be asked to provide services performed under this protocol, eg, attendance at investigator meetings. If required under the applicable statutory and regulatory requirements, Gilead will capture and disclose to Federal and State agencies any expenses paid or reimbursed for such services, including any clinical study payments, meal, travel expenses or reimbursements, consulting fees, and any other transfer of value.

9.3.2. Access to Information for Monitoring

The monitor is responsible for routine review of the eCRF at regular intervals throughout the study to verify adherence to the protocol and the completeness, consistency, and accuracy of the data being entered on them. The monitor should have access to any participant records needed to verify the entries in the eCRF. The investigator agrees to cooperate with the monitor to ensure that any problems detected through any type of monitoring (central, on site) are resolved.

9.3.3. Access to Information for Auditing or Inspections

Representatives of regulatory authorities or of Gilead may conduct inspections or audits of the clinical study. If the investigator is notified of an inspection by a regulatory authority the investigator agrees to notify the Gilead medical monitor immediately. The investigator agrees to provide to representatives of a regulatory agency or Gilead access to records, facilities, and personnel for the effective conduct of any inspection or audit.

9.3.4. Study Discontinuation

Both Gilead and the investigator reserve the right to terminate the study at any time. Should this be necessary, both parties will arrange discontinuation procedures and notify the participants, appropriate regulatory authorities, IRBs, and IECs. In terminating the study, Gilead and the investigator will assure that adequate consideration is given to the protection of the participants' interests.

9.3.5. Data Protection

Enterprise level technical and organizational controls have been developed at Gilead for the purpose of data protection. This includes user authentication and identification, fine grained access controls, end-to-end data encryption, security monitoring, network segregation, and physical security controls. Users of Gilead systems are provided training for security awareness and privacy.

To prepare for the possibility of a data security breach, Gilead maintains a business continuity and disaster recovery plan and conducts regular disaster recovery testing to ensure that Gilead systems are recoverable if a cyber or data security incident is experienced. Gilead's detailed incident response plan for any cyber or data security incident is based on the following 5 steps: detection, analysis, containment, eradication, and recovery. Gilead's standard clinical trial agreement with study sites also includes data privacy language and arrangements in case of data security breaches as follows:

Gilead and institutions will both act in accordance with the applicable data protection law. Furthermore, the study site and Gilead will cooperate with each other to take the necessary measures in order to comply with the applicable data protection law. Both Gilead and the study site shall implement appropriate technical and organizational measures to meet the requirements of the EU General Data Protection Regulation. If either party becomes aware of a personal data breach related to data processed under this agreement, that party shall promptly notify the other party. In such a case, parties will fully cooperate with each other to remedy the personal data breach and promptly fulfill the (statutory) notification obligations. A personal data breach refers to a personal data breach as described in Article 4, Article 33, and Article 34 of the EU General Data Protection Regulation and applicable national data protection laws.

10. REFERENCES

Chan IS, Zhang Z. Test-based exact confidence intervals for the difference of two binomial proportions. Biometrics 1999;55 (4):1202-9.

Claborn KR, Meier E, Miller MB, Leffingwell TR. A Systematic Review Of Treatment Fatigue Among HIV-Infected Patients Prescribed Antiretroviral Therapy. Psychol Health Med 2015;20 (3):1-11.

Cockcroft DW, Gault MH. Prediction of creatinine clearance from serum creatinine. Nephron 1976;16:31-41.

Department of Health and Human Services (DHHS). Guidelines for the Use of Antiretroviral Agents in HIV-1-Infected Adults and Adolescents. Developed by the HHS Panel on Antiretroviral Guidelines for Adults and Adolescents – A Working Group of the Office of AIDS Research Advisory Council (OARAC). Revised 12 February 2013. Available at: http://aidsinfo.nih.gov/guidelines. Accessed: 2013 February 12. 2013.

Emu B, Fessel J, Schrader S, Kumar P, Richmond G, Win S, et al. Phase 3 Study of Ibalizumab for Multidrug-Resistant HIV-1. N Engl J Med 2018;379 (7):645-54.

Lundgren JD, Clumeck N, Rockstroh J, Ryom L. European AIDS Clinical Society (EACS) Guidelines. Version 7.0 October. 2013:

Thompson MA, Aberg JA, Cahn P, Montaner JS, Rizzardini G, Telenti A, et al. Antiretroviral treatment of adult HIV infection: 2010 recommendations of the International AIDS Society-USA panel. JAMA 2010;304 (3):321-33.

U. S. Department of Health and Human Services, Food and Drug Administration (FDA), Center for Drug Evaluation and Research (CDER). Human Immunodeficiency Virus-1 Infection: Developing Antiretroviral Drugs for Treatment. Guidance for Industry. Silver Spring, MD. November, 2015.

UNAIDS. Fact Sheet 2022. Global HIV statistics. 2022:

Williams I, Churchill D, Anderson J, Boffito M, Bower M, Cairns G, et al. British HIV Association guidelines for the treatment of HIV-1-positive adults with antiretroviral therapy 2012 (Updated November 2013. All changed text is cast in yellow highlight.). HIV Med 2014;15 Suppl 1:1-85.

11. APPENDICES

Appendix 1. Investigator Signature Page

GILEAD SCIENCES, INC.
333 LAKESIDE DRIVE
FOSTER CITY, CA 94404

STUDY ACKNOWLEDGMENT

A Phase 2/3 Study to Evaluate the Safety and Efficacy of Long-Acting Capsid Inhibitor GS-6207 in Combination With an Optimized Background Regimen in Heavily Treatment Experienced People Living With HIV-1 Infection With Multidrug Resistance

GS-US-200-4625 Amendment 5, 22 May 2024

This protocol has been approved by Gilead Sciences, Inc. The following signature documents this approval.

PPD	*[See appended electronic signature]*
Executive Director, Clinical Development	Signature
[See appended electronic signature]	
Date	

INVESTIGATOR STATEMENT

I have read the protocol, including all appendices, and I agree that it contains all necessary details for me and my staff to conduct this study as described. I will conduct this study as outlined herein and will make a reasonable effort to complete the study within the time designated.

I will provide all study personnel under my supervision copies of the protocol and access to all information provided by Gilead Sciences, Inc. I will discuss this material with them to ensure that they are fully informed about the drugs and the study.

Principal Investigator Name (Printed)	Signature
Date	Site Number

Appendix 2. Study Procedures Table

	Screening[a]	Cohort Selection[b]	Day 1[c]	Day 2	Day 5	Day 8	Day 15[d]	Day 16[d]	Day 19[d]	Day 22[d]	Day 1 SC	Weeks 4, 10, 16, 22, 26, 36	Week 52 and Visits Thereafter[e]	Oral Bridging Visits[f]	Week 156 and Visits Thereafter[g]	30, 90, and 180 Day Follow-Up[h]	Early Termination[i]
Written Informed Consent/Assent/Parental Consent	X																
Medical History	X																
Demographic Information	X																
Complete Physical Examination	X		X				X				X	X[j]	X[j]				X
Symptom-Directed Physical Examination				X		X		X		X		X[j]	X[j]	X	X[j]	X	
Vital Signs (include weight)[k]	X		X	X		X	X	X		X	X	X	X	X	X	X	X
12-lead ECG (supine)	X																
Height	X																
Hematology[l], Chemistry[m], Estimated GFR, Urinalysis[n], Urine Chemistry[n], CD4+ Cell Count	X		X			X	X			X	X	X	X	X	X	X	X
Urine Storage Sample			X	X		X	X	X		X	X	X	X	X			X
Serum Pregnancy Test[o]	X																
Serum FSH[p]	X																
Urine Pregnancy Test[o]			X			X	X			X	X	X	X	X	X	X	X
HBV, HCV Testing	X																
HIV-1 Genotyping/Phenotyping	X																
Plasma HIV-1 RNA	X	X	X	X		X	X	X		X	X	X	X	X	X	X	X
Plasma Storage Sample	X	X	X	X		X	X	X		X	X	X	X	X		X	X
Intensive PK Plasma Collection[q]			X				X										

	Screening[a]	Cohort Selection[b]	Day 1[c]	Day 2	Day 5	Day 8	Day 15[d]	Day 16[d]	Day 19[d]	Day 22[d]	Day 1 SC	Weeks 4, 10, 16, 22, 26, 36	Week 52 and Visits Thereafter[e]	Oral Bridging Visits[f]	Week 156 and Visits Thereafter[g]	30, 90, and 180 Day Follow-Up[h]	Early Termination[i]
Single Timed, Single Anytime, and/or Predose PK Plasma Sample[r]				X	X	X		X	X	X	X	X[s,t]	X	X			
CCI [redacted]																	
Begin OBR[w]			X				X				X						
SC LEN Administration[x]											X	X	X		X		
Symptoms Distress Module, SF-36, EQ-5D-5L[y]			X									X	X				
Numeric Pain Scale[z]											X	X	X				
Injection Site Reaction Worksheet[aa]											X	X	X				
Adverse Events/Concomitant Medications	X	X	X	X	X	X	X	X	X	X	X	X	X	X	X[bb]	X	X

AE = adverse event; ALT = alanine aminotransferase; ARV = antiretroviral; AST = aspartate aminotransferase; BUN = blood urea nitrogen; CBC = complete blood count; CD4 = clusters of differentiation 4; CK = creatine kinase; ECG = electrocardiogram; EQ-5D-5L = EuroQol (5 dimensions, 5 levels); FSH = follicle-stimulating hormone; GFR = glomerular filtration rate; GGT = gamma-glutamyl transferase; HBV = hepatitis B virus; HCV = hepatitis C virus; HIV-1 = human immunodeficiency virus type 1; IEC = independent ethics committee; IRB = institutional review board; LDH = lactate dehydrogenase; LEN = lenacapavir; OBR = Optimized Background Regimen; PG = pharmacogenomics; PK = pharmacokinetic(s); RNA = ribonucleic acid; SC = subcutaneous; SF-36 = Short Form-36

a Screening evaluations must be completed within 42 days prior to Day 1.
b Cohort Selection visit to be completed 14 to 30 days after the screening visit until Cohort 1 is fully enrolled.
c Day 1 tests and procedures must be completed prior to study drug administration.
d Days 15, 16, 19, and 22 visits will be completed by Cohort 1B participants only.
e At the Week 52 visit, participants will be given an option to attend visits at Weeks 62, 78, 88, 104, 114, 130, and will continue to alternate between every 10 weeks and every 16 weeks. Participants will receive SC LEN every 6 months (26 weeks) ± 2 weeks from last injection starting at Week 52 visit, while continuing their OBR, until the product becomes accessible to participants through an access program, is commercially available, or until Gilead elects to discontinue the study in the country.
f Only applicable to participants who require oral weekly bridging if an SC injection of LEN cannot be administered for any reason within the protocol visit window.
g At Week 156 and thereafter, upon regulatory and/or IRB/IEC approval of the protocol and when participants have provided written informed consent of the most current IRB-approved consent form, participants will continue to receive study drug every 26 weeks, and continue to attend visits every 26 weeks (± 2 weeks). Participants will receive SC LEN every 6 months (26 weeks) ± 2 weeks from the last injection starting at the Week 156 visit, while continuing their OBR, until the product becomes accessible to participants through an access program, is commercially available, or until Gilead elects to discontinue the study in the country.
h Participants may be required to return to the clinic for a 30-, 90-, and 180-day follow-up visit after early termination visit as noted in Section 6.4.1.
i Early termination visit to be completed, if participant decides to discontinue study drug prior to completing Week 52 visit or prior to study completion. Investigators should counsel participant regarding the importance of continuing a complete ARV therapy in accordance to standard of care, and refer participant to an appropriate HIV treatment facility.

j Complete physical examination to be completed at Weeks 26, 52, and 156, symptom-directed physical examination to be completed at all other visits.

k Vital signs: blood pressure, pulse, respiration rate, and temperature, weight.

l Hematology: CBC with differential and platelet count.

m Chemistries: alkaline phosphatase, AST, ALT, GGT, total bilirubin, direct and indirect bilirubin, total protein, albumin, LDH, CK, bicarbonate, BUN, calcium, chloride, creatinine, glucose, lipase, magnesium, phosphorus, potassium, sodium, uric acid (except at Cohort Selection, Days 2, 5, 16, and 19 visits).

n Urinalysis and Urine Chemistry: including color & clarity, specific gravity, pH, glucose, ketones, bilirubin, urobilinogen, blood, nitrite, leukocyte esterase and microscopic (if microscopic elements are seen), urine protein, albumin, creatinine, phosphate, calcium, magnesium and uric acid (except at Cohort Selection, Days 2, 5, 16, and 19 visits).

o All women will have a serum test performed at screening. Urine pregnancy test will be performed at all subsequent visits for women of childbearing potential (except at Days 2, 5, 16, and 19). Positive urine pregnancy tests will be confirmed with a serum test.

p FSH test is required for women who are < 54 years old and have stopped menstruating for ≥ 12 months but do not have documentation of ovarian hormonal failure.

q Day 1 (for all participants) and Day 15 (for Cohort 1B participants only): 0 (predose, ≤ 30 min before dose), 1, 2, 4, 6, and 8 hours postdose.

r Days 2, 8, 16, and 22: A predose sample (within 30 minutes of dosing) and a single timed PK samples between 1 and 6 hours postdose. Days 5 and 19 (if the visits are completed): a single anytime PK sample.

s During the Maintenance Period, at all visits without SC LEN injections, including the Oral Bridging visits, if applicable: A single anytime PK sample will be collected.

[redacted]

v Cohort 1 participants will be administered oral LEN or placebo to match LEN at Days 1, 2, and 8. Participants who receive placebo to match LEN at Days 1, 2, and 8 will receive oral LEN at Days 15, 16, and 22. Cohort 2 participants will be administered oral LEN at Days 1, 2, and 8. Participants requiring oral bridging will be administered oral LEN per Section 5.4, as applicable.

w Cohort 1 participants will begin an OBR on Day 15 (Cohort 1B) or Day 1 SC (Cohort 1A). Cohort 2 participants will begin an OBR on Day 1. An OBR should be selected based on the screening and/or available historical HIV resistance reports.

x All participants will be administered SC LEN at Day 1 SC (14 days after the first dose of oral LEN) and will continue to receive SC LEN every 6 months (26 weeks) ± 2 weeks from last injection. Each SC LEN dosing should occur 6 months (26 weeks) ± 2 weeks from last injection.

y Participants ≥ 18 years of age at Day 1 visit will complete Symptoms Distress Module, SF-36, EQ-5D-5L at Day 1, Weeks 4, 16, 26, and 52, if available (before completing other study procedures).

z Participants ≥ 18 years of age at Day 1 visit will complete the Numeric Pain Rating Scale at Day 1 SC, Weeks 26 and 52 (after they receive SC LEN injections).

aa Provide injection site reaction assessment worksheet and instruct the participants to measure and report injection site reactions following the administration of the SC injections.

bb For visits after Week 156, it is recommended that a phone contact to the participant is made in between in-clinic visits (ie, every 13 weeks) to assess for any AEs experienced by the participant.

Appendix 3. Management of Clinical and Laboratory Adverse Events

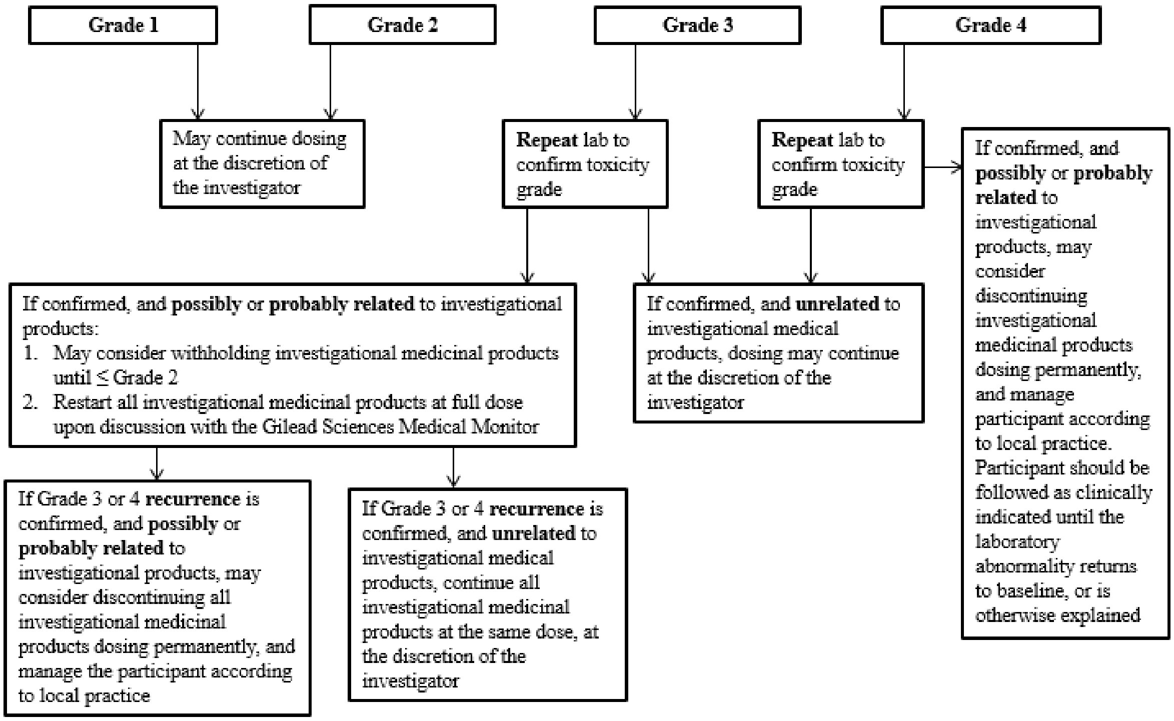

Appendix 4. Pregnancy Precautions, Definition for Women of Childbearing Potential, and Contraceptive Requirements

1) Definitions

a. Definition of Childbearing Potential

For the purposes of this study, a female born participant is considered a woman of childbearing potential following the initiation of puberty (Tanner stage 2) until becoming postmenopausal, unless permanently sterile or with medically documented ovarian failure. For participants deemed by the investigator to have initiated puberty, no documentation of Tanner stage will be required.

Female born participants are considered to be in a postmenopausal state when they are ≥ 54 years of age with cessation of previously occurring menses for ≥ 12 months without an alternative cause. In addition, female born participants < 54 years of age with amenorrhea of ≥ 12 months may also be considered postmenopausal if their follicle-stimulating hormone level is in the postmenopausal range and they are not using hormonal contraception or hormonal replacement therapy.

For the purposes of this study, permanent sterilization is hysterectomy, bilateral oophorectomy, or bilateral salpingectomy in a female born participant of any age. Tubal ligation is not considered permanent sterilization.

b. Definition of Male Fertility

For the purposes of this study, a male born participant is considered fertile after the initiation of puberty unless permanently sterile by bilateral orchidectomy or medical documentation.

2) Contraception Requirements for Female Born Participants

a. Study Drug Effects on Pregnancy and Hormonal Contraception

Nonclinical toxicity studies of lenacapavir (LEN; GS-6207) have demonstrated no adverse effect on fertility or embryo-fetal development. However, there are no clinical studies of LEN in pregnant women. Based on in vitro and in vivo drug-drug interaction liability assessment, a clinically significant drug-drug interaction with LEN and hormonal contraceptives is not expected; an oral contraception drug-drug interaction study was not done.

b. Contraception Requirements for Female Born Participants of Childbearing Potential

The inclusion of female born participants of childbearing potential requires using at least an acceptable effective contraceptive. They must have a negative serum pregnancy test at screening and a negative pregnancy test at the Day 1 visit prior to the dose of study drug. Pregnancy tests will be performed as defined by the Study Procedures Table (Appendix 2). In the event of a delayed menstrual period (over 1 month between menstruations), a pregnancy test must be performed to rule out pregnancy. This is also applicable for female born participants of childbearing potential with infrequent or irregular periods.

Duration of contraception for female born participants of childbearing potential enrolled in this clinical trial should start from screening visit until 60 days after the last dose of oral study drug or 700 days following the last dose of subcutaneous (SC) study drug, whichever is later, as applicable.

Female born participants of childbearing potential must agree to 1 of the following contraceptive methods:

Complete abstinence from intercourse of reproductive potential. Abstinence is an acceptable method of contraception only when it is in line with the participant's preferred and usual lifestyle.

Or

- Consistent and correct use of 1 of the following methods of birth control listed below:
  - Hormonal and nonhormonal intrauterine device (IUD)
  - Bilateral tubal occlusion sterilization (upon medical assessment of surgical success)
  - Vasectomy in the male born partner (upon medical assessment of surgical success)

Or

Female born participants who initiate use of a hormonal contraceptive > 7 days after onset of menses as one of their birth control methods should use additional back-up contraception (eg, condoms) or avoid sexual intercourse for 7 days. Hormonally-based contraceptives and barrier methods permitted for use in this protocol are as follows:

- Hormonal Methods
  - Oral contraceptives (either combined or progesterone only)
  - Injectable progesterone[a]
  - Subdermal contraceptive implant[a]
  - Transdermal contraceptive patch[a]
  - Contraceptive vaginal ring[a]
- Barrier methods
  - Male condom (with or without spermicide)
  - Female condom (with or without spermicide)
  - Diaphragm with spermicide[a]

- Cervical cap with spermicide[a]
- Sponge with spermicide

[a] Not approved in Japan.

Inclusion of methods of contraception in this list of permitted methods does not imply that the method is approved in any country or region. Methods should only be used if locally approved.

Female born participants must also refrain from egg donation and in vitro fertilization during treatment and until the end of contraception requirement.

3) Contraception Requirements for Male Born Participants

No contraception measures are needed.

Condoms should be used for all sexual activity including oral, vaginal, and anal sexual contact to decrease the risk of transmission of HIV and other sexually transmitted diseases.

4) Unacceptable Birth Control Methods

Birth control methods that are unacceptable include periodic abstinence (eg, calendar, ovulation, symptothermal, postovulation methods), withdrawal (coitus interruptus), spermicides only, and lactational amenorrhea method (LAM). A female condom and a male condom should not be used together.

5) Procedures to be Followed in the Event of Pregnancy

Female born participants who are pregnant or suspect they are pregnant will be instructed to notify the investigator at any time during the study and within 700 days following the last dose of SC LEN, or 60 days following the last oral dose of LEN. Male participants whose partner has become pregnant or suspects she is pregnant during the study, including the protocol-defined posttreatment follow-up period, must report the information to the investigator. Female born participants who become pregnant or who suspect that they are pregnant during the study should discontinue study drug immediately. Instructions for reporting pregnancy, partner pregnancy, and pregnancy outcome are outlined in Section 7.7.2.1.

Appendix 5. Disaster and Public Health Emergency Risk Assessment and Mitigation Plan

In the event of an ongoing pandemic (such as COVID-19), potential risks associated with participants being unable to attend study visits have been identified for this study.

These risks can be summarized as follows:

- Completion of study visits which require on-site study drug administration:

  — Oral and SC study drug administration must occur at the study site. Participants may be unable to return to the site, or the site may be unable to accept any participant visits. If a dosing visit is missed, the participant would not be able to complete dosing per protocol.

  Mitigation plan: Prior to initiation of study drug dosing, investigator or designee should ensure investigator or designee and participant availability to complete dosing visits from Day 1 through Day 1 SC visit. If oral or SC dosing (at Day 1 SC and subsequent) is not completed per protocol, sites should inform sponsor immediately upon becoming aware of the issue and obtain sponsor's input for participant's dosing management.

- Study drug shortage at the sites:

  — Delayed or missed study drug shipment to the sites will cause a shortage of study drug at the sites.

  Mitigation plan: The investigator or designee should closely monitor their study drug inventory. Site staff should notify the sponsor or delegate if they foresee shortage in study drug inventory or if there is any interruption in local shipping service. The sponsor will continue to monitor inventory at the study drug depot and study sites. Manual shipments will be triggered, as necessary.

- Participant safety monitoring and follow-up:

  — Participants cannot come to the study site for their scheduled study visits as required per protocol.

  Mitigation plan: For participants who cannot come to the study site for their scheduled study visits as required per protocol, the principal investigator or qualified delegate will conduct a virtual study visit, via phone or video conferencing, to assess the participant within target visit window date whenever possible. During the virtual study visit, the following information at minimum will be reviewed:

  - ■ Confirm if participant has experienced any adverse events (AEs)/serious adverse events (SAEs) and follow-up on any unresolved AE/SAEs.

  - ■ Review current list of concomitant medications and document any new concomitant medications.

■ Review any changes in medical history.

■ Remind participant to continue to administer other antiretrovirals (failing regimen or optimized background regimen).

— Safety blood draws & central laboratory analysis cannot be done.

Mitigation plan: Local labs may be utilized as appropriate to monitor participant safety until the participant can return to the site for their regular follow-up per protocol per principal investigator discretion. Any laboratory assessments conducted at a local laboratory due to the pandemic will be documented accordingly.

— Participants are unable to attend the study visit to sign an updated informed consent form version.

Mitigation plan: The site staff will follow their approved consent process and remain in compliance with local institutional review board (IRB)/independent ethics committee (IEC) and national laws and regulations. Remote consent will be allowed if approved by the local IRB/IEC. The consent process will be documented and confirmed by normal consent procedure at the earliest opportunity.

- Protocol and monitoring compliance:

— Protocol deviations in case scheduled visits cannot occur as planned per protocol.

Mitigation plan: If it is not possible to complete a required procedure at scheduled visit, the visit should be conducted as soon as possible when conditions allow. The situation should be recorded and explained as a protocol deviation. Any missed participant visits or deviation to the protocol due to the pandemic must be reported in the electronic case report form and described in the clinical study report (CSR). Any virtual study visits that are conducted in lieu of clinic visits due to the pandemic will be documented as a protocol deviation related to the pandemic.

— On-site monitoring visit is not feasible.

Monitors may be unable to carry out source data review or source data verification (SDV), study drug accountability, or protocol and Good Clinical Practice/Good Postmarketing Study Practice compliance. This may lead to delays in SDV, an increase in protocol deviations, or under reporting of AEs.

Mitigation plan: The study monitor is to remain in close communication with the site to ensure data entry and query resolution (remote SDV not allowed). The study monitor is to reference the Study Monitoring Plan for guidance on how to conduct a remote monitoring visit. The study staff is to save and document all relevant communication in the study files. The status of sites that cannot accept monitoring visits and/or participants on site, must be tracked centrally and updated on a regular basis.

- Missing data and data integrity:
  - Increased number of missing data due to participants missing visits/assessments. This could have an impact on the analysis and the interpretation of clinical study data.

    Mitigation plan: Implications of a pandemic on methodological aspects for the study will be thoroughly assessed and documented, and relevant actions will be taken as appropriate (ie, modification of the statistical analysis plan) and in compliance with regulatory authorities' guidance. Overall, the CSR will describe the impact of the pandemic on the interpretability of study data.

Virtual visits should be documented in the participant's source documents. For any completed virtual visits, associated data will be entered in electronic data capture and a general comment will be added noting that the visit was completed virtually due to pandemic.

Risks will be assessed continuously, and temporary measures will be implemented to mitigate these risks as part of a mitigation plan, as described above. These measures will be communicated to the relevant stakeholders as appropriate and are intended to provide alternate methods that will ensure the evaluation and assessment of the safety of participants who are enrolled in this study.

Since these potential risks would be considered mitigated with the implementation of these measures, the expected benefit-risk assessment of study drug(s) in study participants remains unchanged.

Appendix 6. Country-Specific Requirements

Not applicable.

Appendix 7. Amendment History

A high-level summary of amendment history is provided in tabular form below. Minor changes such as the correction of typographic errors, grammar, or formatting are not detailed.

Separate summary of change documents for earlier amendments are available upon request.

A separate tracked change (red-lined) document comparing the previous version of the protocol to this amendment will be made available upon the publication of this protocol.

Amendment 5 (22 May 2024)

Rationale for Key Changes Included in Amendment 5	Affected Sections
Sections summarizing data from clinical studies has been updated to align with the most recent data available.	Sections 1.2.2, 1.2.2.1, 1.2.2.2, 1.2.2.3, and 1.4
The rationale for the study was updated to remove text specific to the initiation of protocol amendment 3 in Japan because there are no participants in Japan that remain in the study.	Section 1.3
COVID-19 risks to study conduct were broadened to include unanticipated events (disasters or public health emergencies) to align with the United States Food and Drug Administration's September 2023 Guidance.	Section 1.6, Appendix 5
Visit details for participants continuing to receive study drug after Week 156 have been added.	Synopsis, Sections 3.4, 6.3.2, and 6.5.1, Appendix 2
Text has been added to clarify that samples for optional future research and pharmacogenomic research will no longer be collected for visits at Week 156 and thereafter.	Section 3.5.1
Regulatory Quality and Compliance personnel has been updated to quality personnel in Research and Development Quality (R&D Quality) to align with the new department title.	Section 5.1.2
Information for disallowed medications for systemic corticosteroids has been updated in Table 4 (List of Representative Medications That are Prohibited or To Be Used With Caution Due to the Potential for Drug-Drug Interaction With LEN) to align with preexposure prophylaxis guidelines for systemic corticosteroid use.	Section 5.5
Details for assessments to be completed for visits at Week 156 and thereafter have been added.	Section 6.3.2
A new section has been added describing assessments to be completed if early termination occurs after Week 156.	Section 6.4.1.4
Text describing investigator reporting of primary of cause of death has been added to add clarity to reporting requirements for deaths during the follow-up period.	Section 7.3.2
Text has been updated to add more information regarding timing of reporting of serious adverse events (SAEs) and initial SAE reporting to align with internal processes.	Section 7.3.2.1

Rationale for Key Changes Included in Amendment 5	Affected Sections
Section describing management of injection site reactions (ISRs) that are Grade 3 or higher, or persisting for more than 26 weeks has been removed. The additional assessments and extended monitoring criteria described in this section were added to assess the risk of ISRs in participants who were exposed to subcutaneous lenacapavir administered from borosilicate vial lots. This section is deleted as use of borosilicate vials has been discontinued and adequate safety data to assess the risk of ISRs has been collected from the study to date. Additionally, currently there are only 13 participants remaining on the study.	Section 7.6.4
The section describing the safety monitoring committee has been removed because it is no longer applicable to the study. There are no additional meetings planned for the safety monitoring committee.	Section 7.8
Text describing the availability of study results on ClinicalTrials.gov has been added to align with new International Council for Harmonisation (ICH) guidance.	Section 9.2.2
A new section describing data protection has been added to align with new ICH guidance.	Section 9.3.5
Recommendations for phone contact with participants after Week 156 to follow up on AEs has been added.	Appendix 2
Minor changes to correct typographic errors.	Throughout, as needed

Amendment 4 (18 January 2023)

Rationale for Key Changes Included in Amendment 4	Affected Sections
Long-term data collection were added to support that lenacapavir in combination with optimized background regimen at Weeks 104 and 156 (from the first subcutaneous [SC] dose of GS-6207) have sustained durable virologic responses, clinically meaningful improvement in CD4+, and was well tolerated in people living with human immunodeficiency virus with multidrug resistance and limited options. Secondary objectives and endpoints have been added to reflect this long-term data collection. To reflect the long-term data collection window, Year 2 and 3 analyses have been added to the Week 104 and 156 analysis window.	Synopsis, Study Design Schema, Sections 2, 3, 5.5, 8, 8.2.3, and 8.2.4
Since worldwide marketing authorization applications are upcoming, ongoing or approved for lenacapavir participants willing to continue the study beyond Week 52 visit are given the option to also receive SC GS-6207 until the product becomes commercially available.	Synopsis, Section 3, Appendix 2
Clinical studies that are completed and summarized in the Investigator's Brochure were removed from the Background.	Section 1
Business critical update to the language in Section 9.2.2 has been streamlined to avoid misalignment with the publications language in the clinical trial agreement.	Section 9.2.2
Minor changes to correct typographic errors.	Throughout, as needed

Prot GS-US-200-4625 amd-5

ELECTRONIC SIGNATURES

Signed by	Meaning of Signature	Server Date (dd-MMM-yyyy hh:mm:ss)
PPD	Clinical Development eSigned	23-May-2024 17:59:57
